# The Evolution of Transglutaminases Underlies the Origin and Loss of Cornified Skin Appendages in Vertebrates

**DOI:** 10.1093/molbev/msae100

**Published:** 2024-05-23

**Authors:** Attila Placido Sachslehner, Marta Surbek, Karin Brigit Holthaus, Julia Steinbinder, Bahar Golabi, Claudia Hess, Leopold Eckhart

**Affiliations:** Department of Dermatology, Medical University of Vienna, 1090 Vienna, Austria; Department of Dermatology, Medical University of Vienna, 1090 Vienna, Austria; Department of Dermatology, Medical University of Vienna, 1090 Vienna, Austria; Department of Dermatology, Medical University of Vienna, 1090 Vienna, Austria; Department of Dermatology, Medical University of Vienna, 1090 Vienna, Austria; Clinic for Poultry and Fish Medicine, Department for Farm Animals and Veterinary Public Health, University of Veterinary Medicine Vienna, 1210 Vienna, Austria; Department of Dermatology, Medical University of Vienna, 1090 Vienna, Austria

**Keywords:** transglutaminase, gene family, gene loss, hair, water–land transition

## Abstract

Transglutaminases (TGMs) cross-link proteins by introducing covalent bonds between glutamine and lysine residues. These cross-links are essential for epithelial cornification which enables tetrapods to live on land. Here, we investigated which evolutionary adaptations of vertebrates were associated with specific changes in the family of TGM genes. We determined the catalog of TGMs in the main clades of vertebrates, performed a comprehensive phylogenetic analysis of TGMs, and localized the distribution of selected TGMs in tissues. Our data suggest that TGM1 is the phylogenetically oldest epithelial TGM, with orthologs being expressed in the cornified teeth of the lamprey, a basal vertebrate. Gene duplications led to the origin of *TGM10* in stem vertebrates, the origin of *TGM2* in jawed vertebrates, and an increasing number of epithelium-associated TGM genes in the lineage leading to terrestrial vertebrates. *TGM9* is expressed in the epithelial egg tooth, and its evolutionary origin in stem amniotes coincided with the evolution of embryonic development in eggs that are surrounded by a protective shell. Conversely, viviparous mammals have lost both the epithelial egg tooth and *TGM9*. *TGM3* and *TGM6* evolved as regulators of cornification in hair follicles and underwent pseudogenization upon the evolutionary loss of hair in cetaceans. Taken together, this study reveals the gain and loss of vertebrate TGM genes in association with the evolution of cornified skin appendages and suggests an important role of *TGM9* in the evolution of amniotes.

## Graphical abstract

**Graphical abstract:**
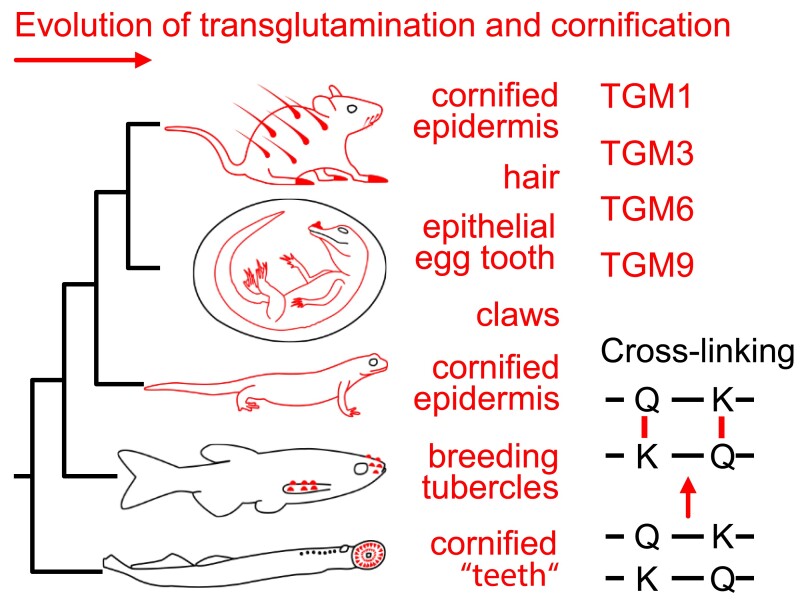

## Introduction

Transglutaminases (TGMs) are calcium-dependent enzymes which introduce covalent bonds (Nε-(γ-glutamyl)lysine bridges) between lysine and glutamine residues of proteins (Greenberg et al. 1991). This process, known as transglutamination, allows the formation of large macromolecular structures which have been implicated in blood coagulation, apoptotic cell death, and the cornification of epithelia (Eckert et al. 2014). In addition, TGMs can catalyze the deamidation of glutamine residues and the linkage of amines to glutamine residues of proteins, known as transamidation (Lorand and Iismaa 2019). TGM genes have been identified and partially characterized in a broad range of metazoans ranging from the fruit fly (Shibata and Kawabata 2018) to humans (Greenberg et al. 1991). However, the evolution of TGMs has remained incompletely understood. In the present study, we utilized comparative genomics to infer the evolutionary history of TGMs in vertebrates, focusing on the evolutionary lineage leading to humans.

Humans have nine TGM genes, namely *TGM1* through *TGM7*, *F13A1*, and *EPB42* with different expression patterns (Eckert et al. 2014). *TGM2* is expressed in a wide variety of cell types, whereas expression of *F13A1* (coagulation factor XIII A chain) is confined to macrophages (Beckers et al. 2017). *EPB42* (erythrocyte membrane protein band 4.2) is expressed as a catalytically inactive TGM family protein in erythrocytes (Korsgren et al. 1990), and *TGM4* is specific for the prostate (Dubbink et al. 1996). Similarly, the expression of *TGM1*, *TGM3*, and *TGM5* is confined to stratified epithelia, such as the epidermis of the skin, skin appendages, and the esophageal epithelium. Epithelial cells, known as keratinocytes, of the epidermis, hair, and nails undergo cornification, that is, a conversion into dead, mechanically stable and interconnected cell remnants, known as corneocytes (Candi et al. 2005; Yamane et al. 2010; Surbek et al. 2023). Protein cross-linking is an essential step of cornification (Eckhart et al. 2013), and the importance of TGMs for cornification is highlighted by the effects of TGM gene mutations in knockout mouse models and human patients. Deletion of *Tgm1* in mice leads to a skin barrier defect and perinatal death due to excessive transepidermal water loss (Matsuki et al. 1998; Kuramoto et al. 2002). Humans can partially compensate inactivating mutations of *TGM1*, but develop severe ichthyosis (Rice et al. 2003; Zhang et al. 2019). Mutations of *TGM3* and *TGM5* cause deformations of hair fibers and acral peeling skin syndrome, respectively (Cassidy et al. 2005; Ü Basmanav et al. 2016). *TGM6* and *TGM7* are the least characterized human TGMs, with contradictory data on expression being available in the literature (Grenard et al. 2001; Thomas et al. 2013) and in the Genotype-Tissue Expression database (https://www.gtexportal.org/, last accessed on 2024 February 9). In addition to orthologs of human TGMs, a gene termed *TGM8* was identified in zebrafish (Deasey et al. 2012).

**Fig. 1.**
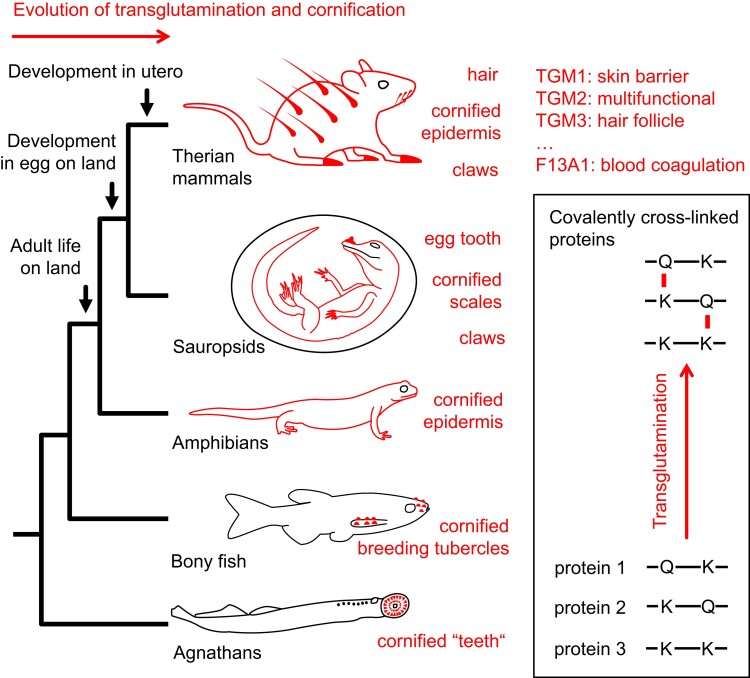
Cornified skin structures evolved in vertebrates and depend on protein cross-linking by transglutamination. Schematic depiction of the cornified integumentary structures (red) in different lineages of vertebrates (left panel). Transglutamination is a mechanism of covalently cross-linking proteins at glutamine (Q) and lysine (K) residues, which is catalyzed by TGMs, including coagulation factor XIIIa (F13A1) (right panel). This study tests the hypothesis that the evolutionary origin and loss of skin appendages, such as the egg tooth, claws, or hair, were associated with specific changes in the TGM gene family. Parts of the figure are modified from Carron et al. 2024.

Cornified integumentary structures are not specific for mammals, but also form in other vertebrates (Wu et al. 2004; Akat et al. 2022; Alibardi 2022). The cornified layer of the epidermis, the stratum corneum, is conserved in all land-dwelling vertebrates (Alibardi 2003; Candi et al. 2005; Akat et al. 2022). The stratum corneum consists of a single layer of dead cells in amphibians and many layers thereof in amniotes (Bereiter-Hahn et al. 1986). Epidermal cornification is diminished or modified at the subcellular level in mammals that have secondarily acquired a fully aquatic lifestyle (cetaceans and sirenians) (Ehrlich et al. 2019; Menon et al. 2022). Hard skin appendages, such as hair, claws, and feathers, are cornified by transglutamination (Sachslehner et al. 2023) and by the formation of disulfide bonds (Strasser et al. 2015; Harland and Plowman 2018; Ehrlich et al. 2020). The cornified epithelial egg tooth, also known as caruncle, is conserved in many but not all amniotes (Mlitz et al. 2021; Fenelon et al. 2023). Various species of teleost fish have cornified breeding tubercles (Fischer et al. 2014), and the most basal vertebrates, the agnathans (lamprey and hagfish), have cornified epithelial teeth (Fig. 1) (Rice et al. 1994; Zaccone et al. 1995; Alibardi and Segalla 2011). The distribution of cornification among vertebrate taxa suggests that TGMs are active in phylogenetically diverse clades. However, it is unknown whether and how the evolution of cornified structures was linked to the evolution of the TGM gene family.

Here, we determined the repertoire of TGM genes in phylogenetically and phenotypically diverse vertebrates and reconstructed the evolutionary history of TGMs in different lineages. Our results point to key roles of TGM gene innovation and degeneration in the evolution of skin appendages that were critical for evolutionary transitions of vertebrates.

## Results

### The Phylogenetically Basal *TGM1* and *F13A1* Genes are Conserved in Vertebrates, Whereas *TGM4* has been Lost in At Least Four Lineages

**Fig. 2.**
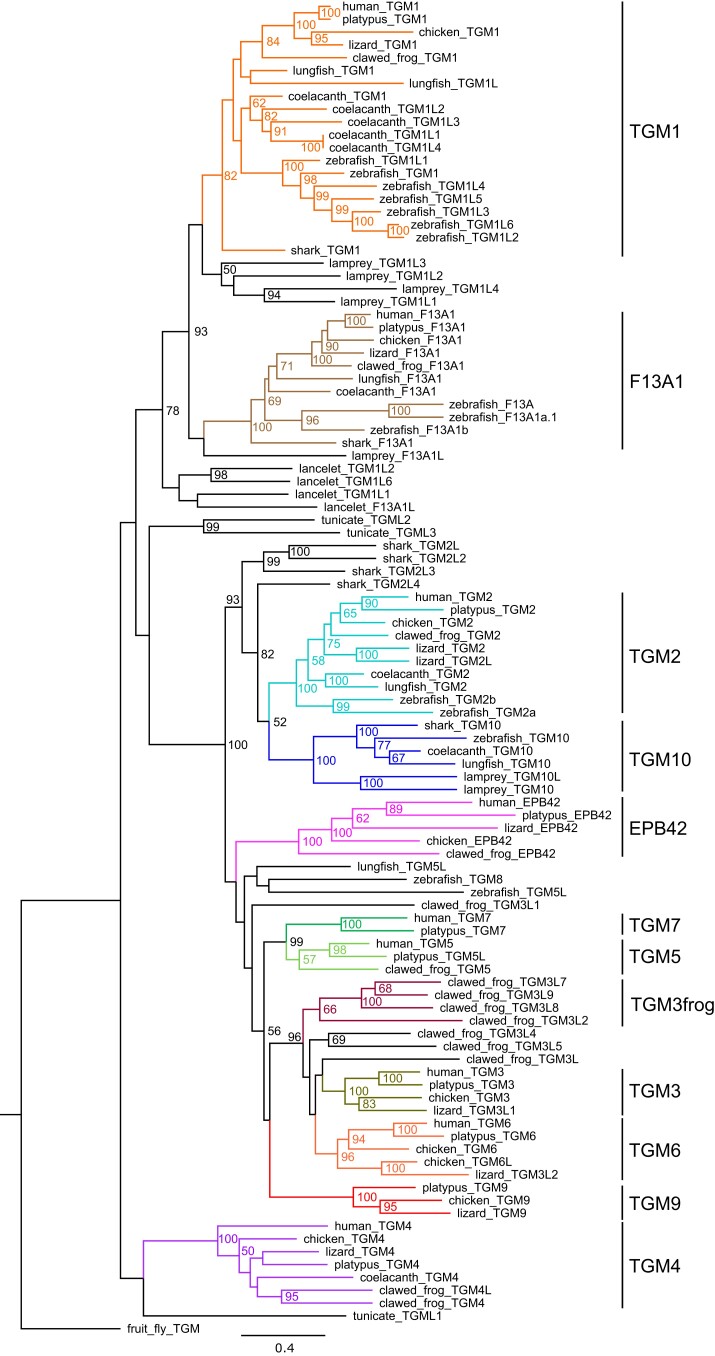
Phylogenetic analysis of TGMs of vertebrates. A phylogenetic tree of TGMs was inferred using the maximum likelihood method. TGMs of species from the main clades of vertebrates were included, with TGMs from a tunicate, the lancelet, and the fruit fly as outgroups. Colors highlight groups of TGMs that are discussed in the text. Bootstrap values above 50 are shown. The scale bar indicates amino acid substitutions per site. Species: Human (*Homo sapiens*), platypus (*Ornithorhynchus anatinus*), chicken (*Gallus gallus*), lizard (*A. carolinensis*), clawed frog (*X. tropicalis*), lungfish (*Protopterus annectens*), coelacanth (*L. chalumnae*), zebrafish (*Danio rerio*), shark (*Carcharodon carcharias*), lamprey (*P. marinus*), tunicate (*Ciona intestinalis*), lancelet (*Branchiostoma floridae*), and fruit fly (*Drosophila melanogaster*).

We identified the complete set of TGM genes in representatives from major groups of vertebrates (Chordata, Olfactores, Vertebrata) and subjected them to a phylogenetic analysis (supplementary table S1 and supplementary fig. S1, Supplementary Material online). TGM genes of a tunicate (Chordata, Olfactores, Tunicata) and lancelet (Chordata, Cephalochordata, Leptocardii) were included for comparison. Besides tunicate TGM genes, which do not cluster together with any TGM of the other species, three distinct clades of TGMs (Fig. 2) were identified: (i) the clade comprising *TGM1* and *F13A1*, (ii) *TGM4*, and (iii) the clade comprising *TGM2* through *TGM8*, *EPB42*, and two genes that we tentatively name *TGM9* and *TGM10*. A comparison of the exon–intron structures showed that, in agreement with a previous report (Phillips et al. 1992), *TGM1* and *F13A1* have an intron that is missing in all other TGM genes (supplementary fig. S2, Supplementary Material online), supporting the hypothesis that *TGM1* and *F13A1* are closely related. *TGM4* does not have a homologous intron but contains another unique intron (supplementary fig. S2, Supplementary Material online).

Homologs of *TGM1* (supplementary figs. S3 and S4 and supplementary table S2, Supplementary Material online) and *F13A1* (supplementary fig. S5 and supplementary table S3, Supplementary Material online) exist in all species investigated, except the tunicates. *TGM1* and *F13A1*-like homologs of the lancelet form the outgroup to the vertebrate *TGM1*/*F13A1* genes (Fig. 2). Sea lamprey, zebrafish, and coelacanth have more than one copy of *TGM1*, indicating lineage-specific gene duplications (supplementary fig. S4, Supplementary Material online). By mass spectrometry-based proteomics, we detected TGM1-like proteins in the cornified epithelial teeth of the sea lamprey (supplementary table S4, Supplementary Material online), suggesting that orthologs of mammalian TGM1 contribute to epithelial cornification in agnathans.

*TGM4* is expressed predominantly in the prostate of humans and mice (Dubbink et al. 1996; Tian et al. 2009). Our comparative genomics and phylogenetic analyses revealed orthologs of *TGM4* in sauropsids, amphibians, the coelacanth, and actinopterygians (ray-finned fish), but not in sharks (supplementary fig. S6 and supplementary table S5, Supplementary Material online), indicating that *TGM4* has originated in stem Osteichthyes (bony fish including tetrapods) and not at the emergence of land vertebrates, as suggested in a previous study (Tian et al. 2009). *TGM4* was independently duplicated in the gray birchir (*Polypterus senegalus*), the diploid frog *Xenopus tropicalis*, and the green anole lizard (*Anolis carolinensis*) and has been lost or pseudogenized in the lungfish (supplementary fig. S6, Supplementary Material online) and at least three groups of mammals, namely marsupials, Paenungulata (a subclade of Afrotheria, comprising elephants and sirenians), and Laurasiatheria (supplementary fig. S6, Supplementary Material online). The origin of *TGM4* in fish and its loss in several taxa of mammals argue against a strict association of *TGM4* with a function in the prostate, because this organ is confined to mammals and conserved among them.

### The Previously Uncharacterized Gene *TGM10* is Present in Fish and has been Lost Upon the Water-to-Land Transition of Tetrapods

According to molecular phylogenetics (Fig. 2), the largest clade of TGMs is subdivided into two subclades, of which the first one comprises *TGM2* and a *TGM2*-like gene (Lisetto et al. 2023) which we tentatively name *TGM10*, and the second subclade comprises the orthologs of human *TGM3*, *TGM5*, *TGM6*, *TGM7*, *EPB42*, fish *TGM8*, and another uncharacterized gene, tentatively named *TGM9*. For further analysis of homology, we performed a synteny analysis of TGM gene loci.

**Fig. 3.**
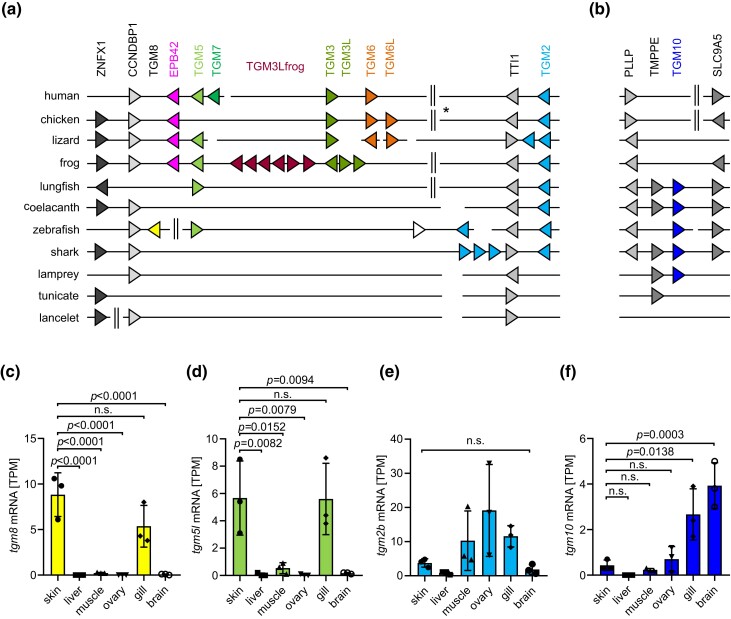
Synteny analysis of *TGM2*-related genes in vertebrates. a) Schematic depiction of gene clusters comprised of *TGM2* and *TGM* genes closely related to *TGM2* (Fig. 2). Genes are shown as triangles pointing in the direction of transcription. Horizontal lines indicate chromosomes or sequence scaffolds. Because of space limitations, not all genes are shown. Vertical double lines indicate gaps in the display where several genes are not shown. An asterisk besides the vertical double lines indicates a complex rearrangement of the left and right segments of the chicken gene locus. b) Locus of the *TGM10* gene in different species. Note that *TGM10* is not present in tunicates and terrestrial vertebrates. c to f) Expression of zebrafish TGMs in tissues. The colors of bars correspond to the colors of the genes in panel (a). RNA-seq data were analyzed for the mRNA levels of *tgm8* (c), *tgm5 l*, which is orthologous to human *TGM5* (d), *tgm2b*, which is one of two orthologs of human *TGM2* (e) and *tgm10* (f). *P*-values were calculated by ANOVA. n.s., not significant. Species: Human (*H. sapiens*), chicken (*Gallus gallus*), lizard (*A. carolinensis*), frog (*X. tropicalis*), lungfish (*P. annectens*), coelacanth (*L. chalumnae*), zebrafish (*D. rerio*), shark (*C. carcharias*), lamprey (*P. marinus*), tunicate (*C. intestinalis*), and lancelet (*B. floridae*).

In most species investigated, the genes *TGM2*, *TGM3*, *TGM5*, *TGM6*, *TGM7*, *EPB42*, and, if present, *TGM8* are clustered at one chromosomal locus (Fig. 3a, supplementary tables S6 and S7, Supplementary Material online). Deviations from this organization are likely due to rearrangements of genes or chromosomal segments. *TGM9* (see the last section of Results) and *TGM10* (Fig. 3b) were found at loci lacking synteny with the aforementioned TGM gene cluster. Importantly, a basal vertebrate, the lamprey, has a *TGM10* gene but lacks TGM genes at the loci corresponding to those of *TGM2* and *TGM9*, suggesting that *TGM10* originated prior to all other TGMs of this clade. Of note, an additional *TGM10*-like gene at another locus of the lamprey genome is likely the product of a lamprey-specific *TGM10* gene duplication (supplementary fig. S7, Supplementary Material online). *TGM10* is conserved in fish including the lungfish but not in tetrapods, indicating that this gene was lost upon the evolutionary transition to life on land.

A cluster of *TGM2* and other TGM genes is located next to *CCNDBP1* (*cyclin D1-binding protein 1*) in jawed vertebrates. In zebrafish, *TGM8* localizes to this cluster, confirming the association in molecular phylogenetics (Fig. 2). Analysis of tissue transcriptomes (supplementary table S8, Supplementary Material online) showed that *TGM8* is predominantly expressed in the skin of the zebrafish, with *TGM1*, *TGM1L1*, *TGM5-like* (*TGM5L*), and *F13A1B* being other skin-enhanced TGMs (Fig. 3, c to f, supplementary fig. S8, Supplementary Material online). A monophyletic subcluster of *TGM3*-like genes (Fig. 2) is present in the frog (Fig. 3a), indicating lineage-specific gene duplications. *EPB42*, which is specifically expressed in erythrocytes (Wang et al. 2020), is present in tetrapods but not in fish, suggesting an evolutionary origin at the time of the water-to-land transition. Residues critical for catalytic TGM activity are conserved in EPB42 orthologs of some but not all amphibians and sauropsids and in none of the mammalian species investigated (supplementary fig. S9, Supplementary Material online), suggesting that EPB42 was originally an active enzyme which lost its activity in distinct lineages, including the one leading to humans.

### Mammalian *TGM6* is Associated with Hair Follicles and has been Lost in Cetaceans

**Fig. 4.**
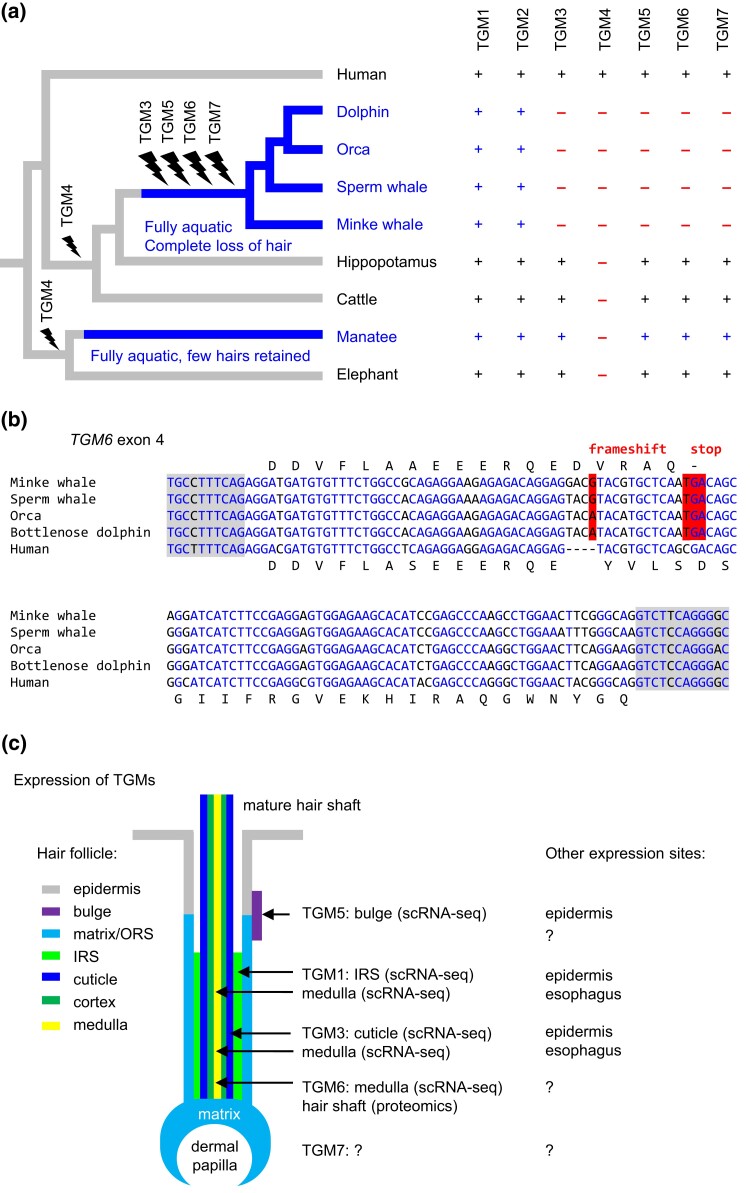
Cetaceans have lost TGM homologs implicated in epithelial cell differentiation within hair follicles. a) Loss of TGM genes (flash symbol) was mapped onto a phylogenetic tree of mammals. The presence (+) or absence (−) of TGM orthologs is indicated for each species. Lineages of fully aquatic mammals are highlighted by blue lines and fonts. b) Nucleotide sequence alignment of exon 4 of human *TGM6* and its homologs in cetaceans. Identical nucleotides in all five species are shown with blue fonts. A nucleotide leading to a frame shift and a premature stop codon are shaded red. Sequences of the flanking introns are shaded gray. Amino acid sequences obtained by translation of the exonic nucleotide sequences are shown for human and minke whale. Note that additional mutations are present in other exons of *TGM6* in cetaceans. c) Schematic depiction of the epithelial compartments of a hair follicles and summary of evidence for expression of individual TGM genes. IRS, inner root sheath; ORS, inner root sheath; sc, single cell.

The *TGM2*-related gene cluster contains at least one gene, namely *TGM3*, that is strongly expressed in epithelial cells of hair follicles (Thibaut et al. 2009; Eckert et al. 2014; Ü Basmanav et al. 2016; Harland and Plowman 2018). The functions of the *TGM3*-related gene *TGM6* (Fig. 2) are not known. We hypothesized that TGMs with a predominant role in hair follicles may have become dispensable in mammals that have entirely lost their hair coat. Thus, we investigated the TGM genes of cetaceans (whales and dolphins), which are the only clade of mammals in which hair has completely disappeared. We found that cetaceans lack protein-coding *TGM3* through *TGM7* genes (Fig. 4a). In contrast to *TGM4*, which is also absent in the closest land-dwelling relatives of cetaceans (Fig. 4a), *TGM3*, *TGM5*, *TGM6*, and *TGM7* have been lost specifically in cetaceans. The loss of TGM5 was reported previously (Sharma et al. 2018). Detailed sequence analysis of *TGM6* showed that, besides other mutations, a frameshift mutation in exon 5 is shared among all cetaceans investigated (Fig. 4b), indicating that *TGM6* underwent pseudogenization in stem cetaceans. By analyzing publicly available gene expression (supplementary tables S9 and S10, Supplementary Material online) and hair proteome data (supplementary tables S11, Supplementary Material online) from mouse studies (Joost et al. 2020; Sukseree et al. 2024), we found evidence for expression of *TGM6* in hair keratinocytes (Fig. 4c, supplementary tables S9 and S11, Supplementary Material online). Of note, *TGM3* and *TGM5* are also expressed in human and mouse tissues other than hair follicles (Fig. 4c, list of “other expression sites”), suggesting that the loss of these genes in cetaceans occurred despite pleiotropic functions of the TGMs. We conclude that the loss of hair follicles, possibly together with modifications of other TGM-associated structures, allowed the pseudogenization of *TGM6* and the contraction of the entire TGM gene repertoire in cetaceans.

### Origin and Loss of *TGM9* Paralleled the Evolution of the Epithelial Egg Tooth in Amniotes and the Evolution of Claws in Squamates

**Fig. 5.**
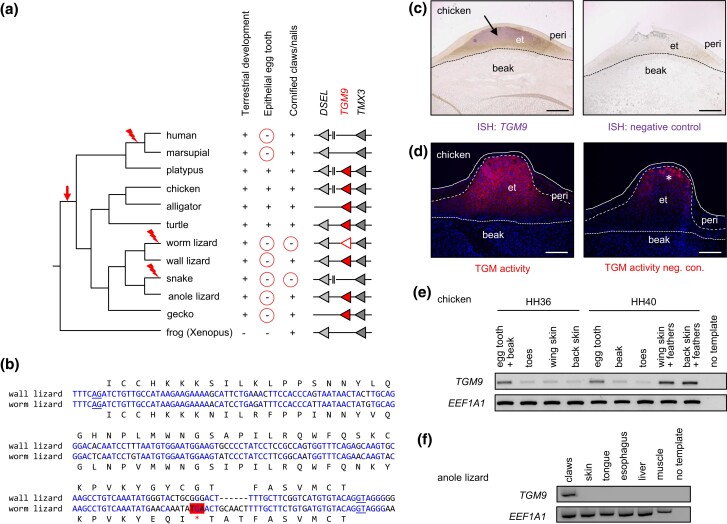
*TGM9* is expressed in the epithelial egg tooth of chicken embryos and in claws of lizards. a) Evolution of *TGM9* in relation to the evolution of cornified skin appendages. The locus of *TGM9* and its neighboring genes was compared between diverse species of tetrapods (right panel). The relationship of species is shown by a phylogenetic tree (left) on which the inferred origin (arrow) and loss of *TGM9* (flash symbol) are indicated. Traits that are correlated with the conservation of *TGM9* are shown for each lineage, whereby “+” and “−” indicate the presence or absence of the respective trait. Red circles highlight the evolutionary loss of traits. Species: human (*H. sapiens*), marsupial (Tasmanian devil, *Sarcophilus harrisii*), platypus (*O. anatinus*), chicken (*Gallus gallus*), alligator (*Alligator sinensis*), turtle (*Mauremys reevesii*), worm lizard (*Rhineura floridana*), wall lizard (*Podarcis muralis*), snake (*Pantherophis guttatus*), anole lizard (*A. carolinensis*), gecko (*Gekko japonicus*), and frog (*X. tropicalis*). b) Nucleotide sequence alignment of exon 6 of the *TGM9* genes of the wall lizard and the legless worm lizard. Splicing signals (underlined) and further intronic sequences are included in the alignment. Sequence identity is highlighted by blue fonts. A premature stop codon is highlighted by red shading. c) mRNA in situ hybridization of *TGM9* in the chicken egg tooth at developmental stage HH36. An arrow points to the strongest signal in the epithelial egg tooth (et). In the negative control reaction (right panel), the antisense probe was replaced by the corresponding sense probe. Scale bars, 200 µm. peri, periderm. d) TGM activity (red) was localized by using a fluorescently labeled (red) substrate on a cryosection of the chicken beak and egg tooth at developmental stage HH36. In the negative control (neg. con.) reaction (right panel), calcium ions were replaced by EDTA. Unspecific signals are indicated by an asterisk. Scale bars, 200 µm. e) RT-PCR analysis of *TGM9* expression in chicken embryos at developmental stages HH36 and HH40. f) RT-PCR analysis of *TGM9* expression in the adult anole lizard (*A. carolinensis*). The house-keeping gene *EEF1A1* was amplified to confirm the integrity of tissue cDNAs in panels e) and f).

Our phylogenetic analysis (Fig. 2) revealed that *TGM9* is most closely related to *TGM3*, *TGM5*, *TGM6*, and *TGM7*. *TGM9* is present in the genomes of turtles, crocodilians, birds, and the platypus, a monotreme mammal, where it is flanked by the conserved genes *TMX3* (*thioredoxin-related transmembrane protein 3*) and *DSEL* (*dermatan sulfate epimerase-like*) (Fig. 5a). The species distribution of *TGM9* orthologs suggests that *TGM9* has originated, presumably by duplication of a *TGM3*/*TGM5*-like gene (Fig. 2), in stem amniotes and that it has been lost or pseudogenized independently in at least three clades of amniotes, namely therian mammals, snakes, and worm lizards (Fig. 5, a and b, supplementary table S12, Supplementary Material online).

We noticed that all of the *TGM9*-negative amniotes lack an epithelial egg tooth, which is a cornified structure that evolved in stem amniotes to facilitate hatching from the egg after completion of embryonic development. To test the hypothesis that the epithelial egg tooth is associated with *TGM9*, we localized *TGM9* mRNA in chicken embryos isolated from eggs (Fig. 5c). *TGM9* mRNA was prominently detected by in situ hybridization in the epithelial cells that give rise to the egg tooth, but only at minute amounts in the beak and the periderm, a transient epithelium that covers the egg tooth and epidermis during development (Fig. 5c). TGM9 co-localized with TGM activity (Fig. 5d). Reverse transcription polymerase chain reaction (RT-PCR) confirmed the expression of *TGM9* in the egg tooth and revealed additional expression of *TGM9* in the wings and back skin where feathers were developing (Fig. 5e). To substantiate these data, we determined the proteome of the epithelial egg tooth and detected the TGM9 protein in three biological replicates there of (PRIDE dataset: PXD048875, accession number TGM9: XP_040519905.1). Reanalysis of a publicly available proteomic dataset showed that TGM9 is also present in developing feathers of the pied flycatcher (*Ficedula hypoleuca*) (protein accession number Ensembl: ENSTGUP00000009578, corresponding to GenBank: XP_030121789.1) (Leskinen et al. 2012). In the anole lizard (*A. carolinensis*), *TGM9* mRNA was detected by RT-PCR in the claws (Fig. 5f), whereas internal organs were *TGM9*-negative.

These data suggest that *TGM9* plays roles of in diverse cornified skin appendages of amniotes and that the loss of the epithelial egg tooth has been a prerequisite for the degeneration of the *TGM9* gene. Additional constraints on the evolution of *TGM9* appear to arise from its roles in claws, so that claw-bearing squamates have retained the *TGM9* gene after the loss the epithelial egg tooth, whereas limbless and therefore also clawless squamates (snakes and worm lizards) have lost *TGM9*.

## Discussion

**Fig. 6.**
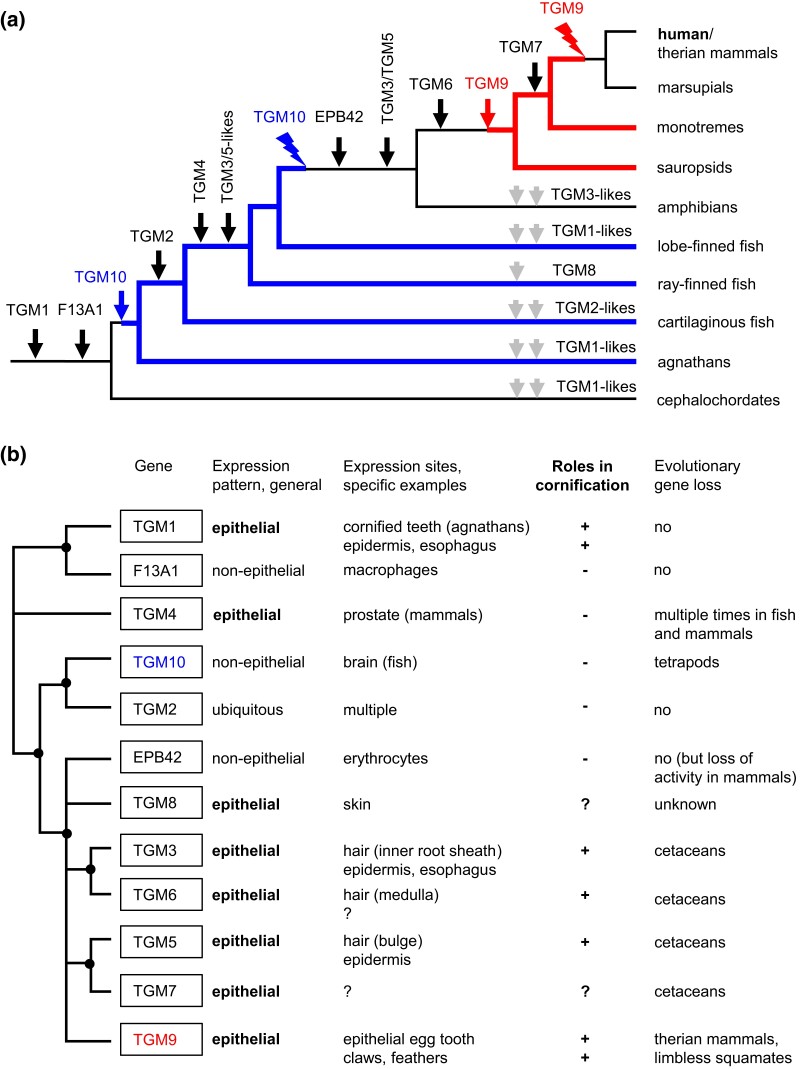
Gene duplications and gene loss have shaped the repertoire of TGMs in humans and other vertebrates. a) The evolution of the TGM gene family is schematically depicted on a phylogenetic tree focusing on the lineage leading to humans. The origin (vertical arrows) and loss (flash symbols) of TGM genes are indicated at positions inferred from the species distribution of TGMs in extant species. The presence of *TGM10* and *TGM9* is indicated by blue and red colors of branches, respectively. b) Summary of functional diversification of TGMs in vertebrates. The phylogenetic relationships of TGMs are indicated by a simplified tree (left side) in which well-supported nodes (Fig. 2) are marked.

The results of this study suggest a new model for the evolution of TGMs in vertebrates (Fig. 6a). *TGM1* and *F13A1* are the phylogenetically basal TGM genes, which evolved from a single ancestral gene prior to the emergence of vertebrates. A subsequent gene duplication in stem vertebrates gave rise to *TGM10* from which the ancestor of *TGM2* evolved at a new locus. Subsequent gene duplications starting from *TGM2* led to the evolution of *TGM3* through *TGM8* and *EPB42* during the diversification of vertebrates. *TGM9* arose from the TGM2-like gene cluster by gene duplication and translocation to a different locus. From the presence or absence of individual TGMs in extant vertebrates, we infer a stepwise diversification of TGMs during the evolution of vertebrates and an important role of gene loss in shaping the human TGM repertoire: (i) *TGM10* was lost after the divergence of tetrapods from other sarcopterygians (lobe-finned fishes) and (ii) *TGM9* was lost after the divergence of therian mammals from prototherians. Moreover, our study indicates that duplications (Fig. 6a) and loss (Fig. 6b) of TGM genes occurred also in other lineages of vertebrates.

By combining the model of gene origin and gene loss (Fig. 6a) with the data on gene expression in tissues of diverse species (Fig. 6b), we conclude that two phylogenetically distinct groups of TGMs (Fig. 2) are expressed predominantly in stratified epithelia and cornified skin appendages: (i) TGM1 and (ii) the group of TGM3, TGM5, TGM6, TGM7, TGM8, and TGM9. Other TGMs are expressed either predominantly in nonepithelial tissues or at similar levels in epithelial and nonepithelial tissue. Thus, the role of TGMs in epithelial cornification was either an ancestral trait of vertebrate TGMs with roles in nonepithelial tissues evolving for F13A1, TGM10, TGM2, and possibly TGM4, or TGM-mediated epithelial cornification evolved at least twice during the diversification of TGMs. Importantly, the evolution of epithelial TGMs was constrained by pleiotropic functions of TGMs, correlating with the expression of individual TGMs at more than one epithelial site, and influenced by redundancy of TGMs due to expression of more than one TGM at particular epithelial sites. We propose that, in general, pleiotropy favors conservation of a gene, whereas redundancy favors gene loss. Our findings of TGM gene losses in cetaceans (Fig. 4) and loss of *TGM9* in therian mammals and legless squamates (Fig. 5) indicate that phenotypic adaptations can be associated with the degeneration of genes even when the genes have pleiotropic roles in other phylogenetic lineages. These data support the concept that the conservative effects of pleiotropy are limited by gene redundancy and parallel adaptations of multiple body sites (Hecker et al. 2017).

*TGM1* is the phylogenetically oldest epithelial TGM (Fig. 6). Previous studies have shown expression of *TGM1* in the epidermis of mammals, birds, and ray-finned fish (Rodriguez Cruz et al. 2017). Here, we added proteomic evidence for the presence of *TGM1* orthologs in the cornified epithelial teeth of the sea lamprey, which diverged from the aforementioned *TGM1*-positive taxa more than 500 million years ago (Kumar et al. 2022). The group of *TGM3*/*TGM5*-like genes evolved more recently than *TGM1* and was not as strictly conserved as *TGM1*. Upon the evolutionary transition to life in water and loss of hair in cetaceans, *TGM3*, *TGM5*, *TGM6*, and *TGM7* were pseudogenized or lost (Fig. 6b). The loss of functional *TGM6* in cetaceans and the evidence for expression of *TGM6* in hair follicles, particularly in the medulla of growing hair fibers (supplementary table S9, Supplementary Material online), suggest that *TGM6* resembles its presumable parental gene, *TGM3*, with regard to a primary function in hair (John et al. 2012). It is worth mentioning that a role of *TGM6* in the central nervous system, as discussed in the literature (Thomas et al. 2013), is not supported by the distribution of *TGM6* mRNA in human tissues, which is limited to the skin (https://www.gtexportal.org/home/gene/TGM6), last accessed on 2024 February 9), and a hypothetical link between mutations of *TGM6* and spinocerebellar ataxia (Wang et al. 2010) was refuted by a recent genetic study (Chen et al. 2020).

Our phylogenetic and synteny analyses define *TGM9* as a gene that evolved in amniotes prior to the divergence of the lineages leading to sauropsids and mammals. Previous studies of chicken keratinocytes cultured in vitro had detected *TGM9* mRNA (Couteaudier et al. 2015; Lachner et al. 2021), which was then named protein—glutamine gamma-glutamyltransferase 5-like according to the GenBank annotation or TG5 (TGM5, ENSGALG00000013762). The data of the current study show that *TGM9* is not orthologous to *TGM5* and, more importantly, reveal expression of *TGM9* in the epithelial egg tooth (Fig. 5, c and e), which is a cornified skin appendage that has played an essential role in the evolutionary transition of tetrapods to fully terrestrial life (Fenelon et al. 2023). The egg tooth must have co-evolved with the egg shell in stem amniotes to facilitate hatching. However, the epithelial egg tooth was lost in specific subgroups of amniotes, namely squamate reptiles, in which the epithelial egg tooth was functionally substituted by a calcified “real” tooth, and in therian mammals, which have replaced embryonic development *in ovo* by development *in utero*. Of note, monotremes have both an epithelial egg tooth (caruncle) and a calcified egg tooth, which, however, differs histologically from the egg tooth of squamates (Fenelon et al. 2023). We could confirm the presence of a complete *TGM9* gene in the platypus (Fig. 5; supplementary table S1, Supplementary Material online), but we found only one exon of *TGM9* in the current genome sequence assembly of the echidna (*Tachyglossus aculeatus*) (GenBank accession number NC_052070.1, nucleotides 28415425 to 28415602). Thus, the evolution of *TGM9* in monotremes requires further investigation. Based on gene expression evidence, we propose that TGM9 contributes to the cornification of the epithelial egg tooth and thereby helps to establish the hardness required for its function in opening the egg shell. The loss of *TGM9* in viviparous mammals supports the primary association of *TGM9* with the epithelial egg tooth, whereas a role of *TGM9* in the claws of squamates is suggested by the conservation of *TGM9* in claw-bearing lizards and geckos (Fig. 5a).

## Conclusion

In conclusion, the evolution of the TGM gene family was linked to important adaptations of the skin epithelium in vertebrates and, indirectly, to major changes in the embryonic development of tetrapods. Future studies will explore the possible coevolution of TGMs with their substrate proteins and the evolution of the mechanisms that regulate the expression of established and newly identified TGMs in vertebrates.

## Materials and Methods

### Comparative Genomics and Sequence Alignments

For the comparative genomic analysis, the genome sequences of human (GCF_000001405.40, International Human Genome Sequencing Consortium), Tasmanian devil (GCF_902635505.1, Stammnitz et al. 2023), platypus (GCF_004115215.2, Zhou et al. 2021), chicken (GCF_016699485.2, Vertebrate Genomes Project), alligator (GCF_000455745.1, Wan et al. 2013), turtle (GCF_016161935.1, Liu et al. 2022), worm lizard (GCF_030035675.1, Vertebrate Genomes Project), wall lizard (GCF_004329235.1, Andrade et al. 2019), corn snake (GCF_029531705.1), green anole lizard (GCF_000090745.1, Alföldi et al. 2011), Japanese gecko (GCF_001447785.1, Kim et al. 2016), frog (GCF_000004195.4, Mitros et al. 2019), lungfish (GCF_019279795.1, Wang et al. 2021), coelacanth (GCF_000225785.1, Amemiya et al. 2013), zebrafish (GCF_000002035.6, Howe et al. 2013), great white shark (GCF_017639515.1, Vertebrate Genomes Project), sea lamprey (GCF_010993605.1, Vertebrate Genomes Project), tunicate (GCF_000224145.3, Satou et al. 2008), and lancelet (GCF_000003815.2, Simakov et al. 2020) were investigated. TGM sequences were collected from NCBI GenBank. The chicken TGM1 homolog was identified in a previous study (Sachslehner, Surbek, et al. 2021). For several genes, the predictions of exon sequences were corrected according to the results of tBLASTn searches with other TGMs as queries (supplementary table S1, Supplementary Material online). *TGM1* and *TGM1L4* of the coelacanth (*Latimeria chalumnae*) lacked large parts of their coding sequences in the annotations available in GenBank. For identification of the complete coding sequences, RNA-seq data of pooled tissues of the coelacanth were downloaded from NCBI GenBank (accession number: SRX112771, run accession: SRR391920) with the prefetch (version 3.0.6) package of SRA Toolkit (https://github.com/ncbi/sra-tools, last accessed on 2024 January 18) and converted to the fastq format with fastq-dump (version 3.0.6) package of SRA Toolkit. A quality check was performed with FastQC (version 0.12.1, https://www.bioinformatics.babraham.ac.uk/projects/fastqc/, last accessed on 2024 January 18). The transcriptome was assembled with Trinity (Grabherr et al. 2011, version 2.15.1), and coding regions were predicted with TransDecoder (version 5.7.1, https://github.com/TransDecoder/TransDecoder, last accessed on 2024 January 18). The complete *TGM1* and *TGM1L4* coding sequences were identified via BLASTp (Altschul et al. 1990, version 2.14.0+). The assembled transcriptome is available at https://doi.org/10.5281/zenodo.10619730.

### Molecular Phylogenetics

Multiple sequence alignments for phylogeny were created and manually trimmed with aliview (Larsson 2014). After trimming, the amino acid sequences contained only the residues that span from the “Transglut_N superfamily” domain to the second “Transglut_C superfamily” domain (supplementary fig. S10, Supplementary Material online). Prottest (version 3.0) (Abascal et al. 2005; Darriba et al. 2011) was used to calculate the amino acid substitution model. All available matrices and models with rate variation among sites were included. The Akaike information criterion was used to assess the likelihood of the predicted models (Akaike 1998). The best suited amino acid substitution model for the TGM phylogeny was LG (Le and Gascuel 2008). Maximum likelihood analysis was performed using PhyML (version 20120412, https://github.com/stephaneguindon/phyml, last accessed on 2024 February 9). Tree topology, branch length, and rate parameters were optimizing according to a published approach (Guindon and Gascuel 2003). Phylogenetic trees were visualized with FigTree (http://tree.bio.ed.ac.uk/software/figtree/, last accessed on 2024 February 2). Phylogenetic trees were edited with Inkscape (version 1.0.0.0; https://inkscape.org/de/, accessed on 2024 January 18).

### Animals and Tissue Samples

Tissue samples were prepared from 21-d-old specific pathogen-free (SPF) chickens and chicken embryos from fertilized SPF eggs (VALO BioMedia, Osterholz-Scharmbeck, Germany) on days 9, 10, and 18 of incubation, corresponding to Hamburger and Hamilton stages 35, 36, and 44, respectively (Hamburger and Hamilton 1951). Samples were obtained from untreated chickens which were maintained in a trial approved by the ethics and animal welfare committee of University of Veterinary Medicine, Vienna, Austria, and the Austrian Federal Ministry of Education, Science and Research (license number BMBWF GZ: GZ-2021-0.842.250). Sea lamprey (*Petromyzon marinus*) tissue was generously provided by the Museum of Natural History Vienna (inventory number: NMW-63577). cDNAs from green anole lizards were available from a previous study (Strasser et al. 2014).

### RNA Extraction and RT-PCR

Tissue samples were placed in RNA-later (Invitrogen) immediately after dissection and stored at 4 °C overnight and subsequently at −80 °C. RNA isolation was performed with Trizol (VWR) from homogenized tissues according to published protocols (Chomczynski 1993). cDNA synthesis was performed with the iScript® cDNA synthesis kit (Quantabio). cDNA was PCR-amplified with Dream Taq DNA polymerase (Thermo Scientific) using the following primers (Microsynth, Switzerland): chicken TGM9 (forward: 5′-AGCGTCCCTATTCTTCAGCA-3′, reverse: 5′-TAGCTTGTCCTTGCCACAGA-3′, product 222 bp, Fig. 5e; forward: 5′-AGTTTTCTGTGACGCTTGGC-3′, reverse: 5′-ACAGAAGAATTCACCCAGGG-3′, product 157 bp, Fig. 5d), chicken eef1a1 (forward: 5′-GCCCCGAAGTTCCTGAAATC-3′, reverse: 5′-GGCCTTGATGACACCAACAG-3′, product 153 bp, Fig. 5, d and e), anole lizard TGM9 (forward: 5′-GAGTGGCAGCAGTCTCAATG-3′, reverse: 5′-GCACCTCCTTCTCCAGATGT-3′, product 400 bp, Fig. 5e), anole lizard eef1a1 (forward: 5′-TTGCCACACTGCCCATATTG-3′, reverse: 5′-CGCTTTCTTGTCAACTGCCT-3′, product 250 bp, Fig. 5e).

### Analysis of Zebrafish RNA-Seq Data

Publicly available zebrafish sequence read archives (SRA) were downloaded from NCBI GenBank (supplementary table S8, Supplementary Material online) with prefetch (version 3.0.10) package of SRA Toolkit (https://github.com/ncbi/sra-tools, last accessed on 2024 January 18) and converted to fastqc files with fastq-dump (version 3.0.10) package of sratoolkit. Quality check was done with FastQC (version 0.12.1, https://www.bioinformatics.babraham.ac.uk/projects/fastqc/, last accessed on 2024 January 18). Expression analysis was performed with Salmon (version 1.10.1, Patro et al. 2017). The reference transcriptome was concatenated with the corresponding genome to create a decoy aware reference index, which avoids mapping of genomic reads, before the calculation of transcripts per million. Statistical significance was calculated with Analysis of Variance (ANOVA).

### Histology, TGM In Situ Activity Labeling

An in situ TGM activity assay combined with immunofluorescence was performed based on a published protocol (Sachslehner et al. 2023). The samples were encircled with a liquid blocker (Daido Sangyo Co. Ltd.) and subsequently incubated with 2% bovine serum albumin (BSA, Sigma-Aldrich) in phosphate-buffered saline (PBS) with 0.05% Tween at room temperature for 30 min. Subsequently, the samples were incubated with 5 µM Alexa-fluor555-cadaverine (Thermo Fisher Scientific) in 0.1 M Tris–HCl pH 7.4 with either 5 mM CaCl_2_ to facilitate TGM activity or 5 mM EDTA to suppress TGM activity (negative control) for 2 h at room temperature under protection from light. The reaction was stopped by incubating the samples in 25 mM EDTA in PBS for 5 min after which the samples were rinsed with PBS. Afterward, the sections were rinsed with PBS and mounted with Permafluor (Thermo Fisher Scientific). Sections were studied with an Olympus BX63 light microscope, and images were taken with an Olympus UC-90 camera using the cellSens Dimensions software (version 1.16).

### Preparation of Riboprobes

A riboprobe template that anneals at the beginning of the coding region of chicken TGM9 was amplified by RT-PCR from chicken skin cDNA using the following primer pair: TGM9 (forward: 5′-CCTGAACTGTCCTTCCAACTGC-3′ and reverse: 5′-GCACAGTGATGAGGTGTTTGG-3′), further ligated into a pGEM-T Easy vector (Promega). Plasmids containing an insert were amplified in *E. coli* (L2001, Promega), and plasmid DNA was isolated with the QIAprep spin miniprep kit 250 (Qiagen). The plasmids were sequenced (Microsynth) using SP6 primers to determine the orientation of the inserts. The plasmids that contained the insert in antisense and sense orientation were linearized via PCR with M13 primers (forward: 5′-CGCCAGGGTTTTCCCAGTCACGAC-3′ and reverse: 5′-CAGGAAACAGCTATGAC-3′). The insert contained a T7 RNA polymerase–binding site and was used as template for in vitro transcription with the DIG RNA labeling mix (Roche). T7 RNA polymerase was used for transcription of antisense and sense probes. RNA probes were precipitated with 4 M LiCl (Sigma-Aldrich) and ethanol overnight at −20 °C, followed by subsequent washing steps with 70% and 100% ethanol and resuspension in RNase-free water. The riboprobes were stored at −80 °C.

### mRNA In Situ Hybridization

Sections were prepared with a Microm HM 335E microtome at a thickness of 5 µm and collected in a water bath set to 42 °C on super-adhesive slides (Menzel). mRNA in situ hybridization was performed on tissue sections of formalin-fixed paraffin-embedded chicken beaks of stage HH36 (Hamburger and Hamilton 1951) based on published protocols (Mlitz et al. 2021; Sachslehner, Zieger, et al. 2021). Briefly, paraffin sections were melted at 58 °C for 1 h, deparaffinized in xylene (Fisher Chemical) for 20 min, and further hydrated in a descending ethanol series. The samples were treated with 20 µg/ml proteinase K for 10 min at 37 °C. Specimens were subsequently incubated for 10 min in 1% triethanolamine (Sigma-Aldrich) in PBS, for 5 min in 1% triethanolamine with 0.15% acetic anhydride (Prolabo), and for 5 min in 1% triethanolamine with 0.3% acetic anhydride to neutralize charged probe binding. Samples were postfixed with 4% paraformaldehyde in PBS for 40 min followed by dehydration in an ascending ethanol series. Samples were dried at room temperature for 1 h and prehybridized in hybridization buffer at 60 °C for 2 h. Corresponding sense and antisense riboprobes were prepared at a concentration of 2 ng/µl and preheated in hybridization buffer (4 M urea, VWR; 5x saline sodium citrate (SSC), Roth; 100 μg/ml heparin, Sigma-Aldrich; 5 mM EDTA, Roth; 1x Denhardt's block reagent, Sigma-Aldrich; 100 μg/ml salmon sperm DNA, Agilent; 5% dextran sulfate, Sigma-Aldrich) at 85 °C for 10 min. Hybridization took place overnight at 60 °C. To remove unbound probes, samples were washed thrice with 4 M urea and 4x SSC, once with a solution containing 4 M urea and 2x SSC, and once with 4 M urea and 1x SSC at 58 °C for 15 min each, followed by one wash with 1x SSC at 37 °C for 15 min. Specimens were incubated for 2 h in 0.1 M maleic acid buffer (MAB, Sigma-Aldrich), pH 7.5 containing 2% BSA to prevent nonspecific antidigoxigenin antibody binding. Afterward, specimens were incubated with an antidigoxigenin antibody conjugated to the alkaline phosphatase enzyme (1:5,000, Roche) in 2% MAB block solution overnight at 4 °C. A buffer suitable for alkaline phosphatase enzyme activity (0.5 M Tris pH 9.5; 0.5 M NaCl) was used, and samples were washed twice with alkaline phosphatase buffer for 15 min at room temperature. The staining was developed with alkaline phosphatase buffer supplemented with 0.05 M MgCl_2_, 3.75 μl/ml 5-bromo-4-chloro-3-indolyl phosphate (Roche), and 5 µl/ml nitroblue tetrazolium (Roche). Sections were mounted with Aquatex (Sigma-Aldrich), and photographs were taken with an Olympus UC-90 camera on an Olympus BX63 light microscope.

### Mass Spectrometry-Based Proteomics

The beaks of stage HH44 chicken embryos were dissected and dried for some minutes at room temperature. The egg tooth became clear after the drying step and was removed from the beak with a pointy scalpel. The epidermis of the upper beak was removed with forceps. Samples were frozen in liquid nitrogen and stored at −80 °C until lysate preparation. Lamprey teeth were obtained from a museum sample that was stored in ethanol at room temperature. The specimens were placed in 200 µl of lysis buffer, made of 30 mM Tris, 7 M urea (VWR), 2 M thiourea (Sigma-Aldrich), and 4% CHAPSO (Pierce). 0.2 M dithiothreitol (DTT) was added to the samples. After incubation at 70 °C for 3 h, the samples were homogenized with a homogenizer (Precellys, VWR) and centrifuged at 18,000 × *g* for 15 min at 4 °C, and the supernatant was collected. The pellet was sonicated with a sonicator (Hielscher Ultrasound Technology) twice for 30 s at an amplitude of 100, and centrifugation was repeated as before. The supernatant was pooled with the one from the initial homogenization and stored at −80 °C until analysis was started. The proteomic analysis and database search were performed like in a previous study (Cruz-Bustos et al. 2023) with the following modification: After protein reduction with 200 mM DTT (37 °C, 30 min), the proteins were alkylated with 500 mM iodoacetamide (Sigma) at 37 °C for 30 min. The database for the lamprey (NCBI_Petromyzon_marinus_tx7757_230919.fasta) and the chicken (NCBI_Gallus_gallus_tx9031_230919.fasta) was downloaded from crap.fasta (https://www.thegpm.org/crap/, last accessed on 2024 February 7).

## Supplementary Material

msae100_Supplementary_Data

## Data Availability

Proteome data were deposited in the PRIDE database under the accession numbers PXD048875 (chicken egg tooth) and PXD048873 (lamprey teeth and skin). The assembled reads of the coelacanth transcriptome (NCBI read archive accession: SRX112771) were deposited at Zenodo (doi: https://doi.org/10.5281/zenodo.10619730).
