# Supplementary material for: The Evolution of Transglutaminases Underlies the Origin and Loss of Cornified Skin Appendages in Vertebrates
Source: Mol Biol Evol. 2024 May 23;41(6):msae100. doi: 10.1093/molbev/msae100 (PMC11152450; doi:10.1093/molbev/msae100)
Supplement: msae100_Supplementary_Data [file msae100_supplementary_data.pdf]

Supplementary Data: Supplementary Figures and Tables

The evolution of transglutaminases underlies the origin and loss of cornified skin appendages in vertebrates

Attila Placido Sachslehner, Marta Surbek, Karin Brigit Holthaus, Julia Steinbinder, Bahar Golabi, Claudia Hess, Leopold Eckhart

Content

Supplementary Figures S1-S10

Supplementary Tables S1-S12

```
>human_TGM1
MMDGPRSDVGRWGGNPLQPPTTPSPEPEPEPDGRSRRGGGRSFWARCCGCCSCRNAADDDWGPEPSDSRGRGSSSGTRRPGSRGSDSRRPVSRG
SGVNAAGDGTIREGMLVVNGVDLLSSRSDQNRREHHTDEYEYDELIVRRGQPFHMLLLLSRTYESSDRITLELLIGNNPEVGKGTHVIIPVGKG
GSGGWKAQVVKASGQNLNLRVHTSPNAIIGKFQFTVRTQSDAGEFQLPFDPRNEIYILFNPWCPEDIVYVDHEDWRQEYVLNESGRIYYGTEAQ
IGERTWNYGQFDHGVLDACLYILDRRGMPYGGRGDPVNVSRVISAMVNSLDDNGVLIGNWSGDYSRGTNPSAWVGSVEILLSYLRTGYSVPYGQ
CWVFAGVTTTVLRCLGLATRTVTNFNSAHDTDTSLTMDIYFDENMKPLEHLNHDSVWNFHVWNDCWMKRPDLPSGFDGWQVVDATPQETSSGIF
CCGPCSVESIKNGLVYMKYDTPFIFAEVNSDKVYWQRQDDGSFKIVYVEEKAIGTLIVTKAISSNMREDITYLYKHPEGSDAERKAVETAAAHG
SKPNVYANRGSAEDVAMQVEAQDAVMGQDLMVSVMLINHSSSRRTVKLHLYLSVTFYTGVSGTIFKETKKEVELAPGASDRVTMPVAYKEYRPH
LVDQGAMLLNVSGHVKESGQVLAKQHTFRLRTPDLSLTLLGAAVVGQECEVQIVFKNPLPVTLTNVVFRLEGSGLQRPKILNVGDIGGNETVTL
RQSFVPVRPGPRQLIASLDSPQLSQVHGVIQVDVAPAPGDGGFFSDAGGDSHLGETIPMASRGGA

>human_TGM2
MAEELVLERCDLELETNGRDHHTADLCREKLVVRRGQPFWLTLHFEGRNYEASVDSLTFSVVTGPAPSQEAGTKARFPLRDAVEEGDWTATVVD
QQDCTLSLQLTTPANAPIGLYRLSLEASTGYQGSSFVLGHFILLFNAWCPADAVYLDSEEERQEYVLTQQGFIYQGSAKFIKNIPWNFGQFEDG
ILDICLILLDVNPKFLKNAGRDCSRRSSPVYVGRVVSGMVNCNDDQGVLLGRWDNNYGDGVSPMSWIGSVDILRRWKNHGCQRVKYGQCWVFAA
VACTVLRCLGIPTRVVTNYNSAHDQNSNLLIEYFRNEFGEIQGDKSEMIWNFHCWVESWMTRPDLQPGYEGWQALDPTPQEKSEGTYCCGPVPV
RAIKEGDLSTKYDAPFVFAEVNADVVDWIQQDDGSVHKSINRSLIVGLKISTKSVGRDEREDITHTYKYPEGSSEEREAFTRANHLNKLAEKEE
TGMAMRIRVGQSMNMGSDFDVFAHITNNTAEEYVCRLLLCARTVSYNGILGPECGTKYLLNLNLEPFSEKSVPLCILYEKYRDCLTESNLIKVR
ALLVEPVINSYLLAERDLYLENPEIKIRILGEPKQKRKLVAEVSLQNPLPVALEGCTFTVEGAGLTEEQKTVEIPDPVEAGEEVKVRMDLLPLH
MGLHKLVVNFESDKLKAVKGFRNVIIGPA

>human_TGM3
MAALGVQSINWQTAFNRQAHHTDKFSSQELILRRGQNFQVLMIMNKGLGSNERLEFIVSTGPYPSESAMTKAVFPLSNGSSGGWSAVLQASNGN
TLTISISSPASAPIGRYTMALQIFSQGGISSVKLGTFILLFNPWLNVDSVFMGNHAEREEYVQEDAGIIFVGSTNRIGMIGWNFGQFEEDILSI
CLSILDRSLNFRRDAATDVASRNDPKYVGRVLSAMINSNDDNGVLAGNWSGTYTGGRDPRSWNGSVEILKNWKKSGFSPVRYGQCWVFAGTLNT
ALRSLGIPSRVITNFNSAHDTDRNLSVDVYYDPMGNPLDKGSDSVWNFHVWNEGWFVRSDLGPSYGGWQVLDATPQERSQGVFQCGPASVIGVR
EGDVQLNFDMPFIFAEVNADRITWLYDNTTGKQWKNSVNSHTIGRYISTKAVGSNARMDVTDKYKYPEGSDQERQVFQKALGKLKPNTPFAATS
SMGLETEEQEPSIIGKLKVAGMLAVGKEVNLVLLLKNLSRDTKTVTVNMTAWTIIYNGTLVHEVWKDSATMSLDPEEEAEHPIKISYAQYEKYL
KSDNMIRITAVCKVPDESEVVVERDIILDNPTLTLEVLNEARVRKPVNVQMLFSNPLDEPVRDCVLMVEGSGLLLGNLKIDVPTLGPKEGSRVR
FDILPSRSGTKQLLADFSCNKFPAIKAMLSIDVAE

>human_TGM4
MMDASKELQVLHIDFLNQDNAVSHHTWEFQTSSPVFRRGQVFHLRLVLNQPLQSYHQLKLEFSTGPNPSIAKHTLVVLDPRTPSDHYNWQATLQ
NESGKEVTVAVTSSPNAILGKYQLNVKTGNHILKSEENILYLLFNPWCKEDMVFMPDEDERKEYILNDTGCHYVGAARSIKCKPWNFGQFEKNV
LDCCISLLTESSLKPTDRRDPVLVCRAMCAMMSFEKGQGVLIGNWTGDYEGGTAPYKWTGSAPILQQYYNTKQAVCFGQCWVFAGILTTVLRAL
GIPARSVTGFDSAHDTERNLTVDTYVNENGEKITSMTHDSVWNFHVWTDAWMKRPDLPKGYDGWQAVDATPQERSQGVFCCGPSPLTAIRKGDI
FIVYDTRFVFSEVNGDRLIWLVKMVNGQEELHVISMETTSIGKNISTKAVGQDRRRDITYEYKYPEGSSEERQVMDHAFLLLSSEREHRRPVKE
NFLHMSVQSDDVLLGNSVNFTVILKRKTAALQNVNILGSFELQLYTGKKMAKLCDLNKTSQIQGQVSEVTLTLDSKTYINSLAILDDEPVIRGF
IIAEIVESKEIMASEVFTSFQYPEFSIELPNTGRIGQLLVCNCIFKNTLAIPLTDVKFSLESLGISSLQTSDHGTVQPGETIQSQIKCTPIKTG
PKKFIVKLSSKQVKEINAQKIVLITK

>human_TGM5
MAQGLEVALTDLQSSRNNVRHHTEEITVDHLLVRRGQAFNLTLYFRNRSFQPGLDNIIFVVETGPLPDLALGTRAVFSLARHHSPSPWIAWLET
NGATSTEVSLCAPPTAAVGRYLLKIHIDSFQGSVTAYQLGEFILLFNPWCPEDAVYLDSEPQRQEYVMNDYGFIYQGSKNWIRPCPWNYGQFED
KIIDICLKLLDKSLHFQTDPATDCALRGSPVYVSRVVCAMINSNDDNGVLNGNWSENYTDGANPAEWTGSVAILKQWNATGCQPVRYGQCWVFA
AVMCTVMRCLGIPTRVITNFDSGHDTDGNLIIDEYYDNTGRILGNKKKDTIWNFHVWNECWMARKDLPPAYGGWQVLDATPQEMSNGVYCCGPA
SVRAIKEGEVDLNYDTPFVFSMVNADCMSWLVQGGKEQKLHQDTSSVGNFISTKSIQSDERDDITENYKYEEGSLQERQVFLKALQKLKARSFH
GSQRGAELQPSRPTSLSQDSPRSLHTPSLRPSDVVQVSLKFKLLDPPNMGQDICFVLLALNMSSQFKDLKVNLSAQSLLHDGSPLSPFWQDTAF
ITLSPKEAKTYPCKISYSQYSQYLSTDKLIRISALGEEKSSPEKILVNKIITLSYPSITINVLGAAVVNQPLSIQVIFSNPLSEQVEDCVLTVE
GSGLFKKQQKVFLGVLKPQHQASIILETVPFKSGQRQIQANMRSNKFKDIKGYRNVYVDFAL

>human_TGM6
MAGIRVTKVDWQRSRNGAAHHTQEYPCPELVVRRGQSFSLTLELSRALDCEEILIFTMETGPRASEALHTKAVFQTSELERGEGWTAAREAQME
KTLTVSLASPPSAVIGRYLLSIRLSSHRKHSNRRLGEFVLLFNPWCAEDDVFLASEEERQEYVLSDSGIIFRGVEKHIRAQGWNYGQFEEDILN
ICLSILDRSPGHQNNPATDVSCRHNPIYVTRVISAMVNSNNDRGVVQGQWQGKYGGGTSPLHWRGSVAILQKWLKGRYKPVKYGQCWVFAGVLC
TVLRCLGIATRVVSNFNSAHDTDQNLSVDKYVDSFGRTLEDLTEDSMWNFHVWNESWFARQDLGPSYNGWQVLDATPQEESEGVFRCGPASVTA
IREGDVHLAHDGPFVFAEVNADYITWLWHEDESRERVYSNTKKIGRCISTKAVGSDSRVDITDLYKYPEGSRKERQVYSKAVNRLFGVEASGRR
IWIRRAGGRCLWRDDLLEPATKPSIAGKFKVLEPPMLGHDLRLALCLANLTSRAQRVRVNLSGATILYTRKPVAEILHESHAVRLGPQEEKRIP
ITISYSKYKEDLTEDKKILLAAMCLVTKGEKLLVEKDITLEDFITIKVLGPAMVGVAVTVEVTVVNPLIERVKDCALMVEGSGLLQEQLSIDVP
TLEPQERASVQFDITPSKSGPRQLQVDLVSPHFPDIKGFVIVHVATAK

>human_TGM7
MDQVATLRLESVDLQSSRNNKEHHTQEMGVKRLTVRRGQPFYLRLSFSRPFQSQNDHITFVAETGPKPSELLGTRATFFLTRVQPGNVWSASDF
TIDSNSLQVSLFTPANAVIGHYTLKIEISQGQGHSVTYPLGTFILLFNPWSPEDDVYLPSEILLQEYIMRDYGFVYKGHERFITSWPWNYGQFE
EDIIDICFEILNKSLYHLKNPAKDCSQRNDVVYVCRVVSAMINSNDDNGVLQGNWGEDYSKGVSPLEWKGSVAILQQWSARGGQPVKYGQCWVF
ASVMCTVMRCLGVPTRVVSNFRSAHNVDRNLTIDTYYDRNAEMLSTQKRDKIWNFHVWNECWMIRKDLPPGYNGWQVLDPTPQQTSSGLFCCGP
ASVKAIREGDVHLAYDTPFVYAEVNADEVIWLLGDGQAQEILAHNTSSIGKEISTKMVGSDQRQSITSSYKYPEGSPEERAVFMKASRKMLGPQ
RASLPFLDLLESGGLRDQPAQLQLHLARIPEWGQDLQLLLRIQRVPDSTHPRGPIGLVVRFCAQALLHGGGTQKPFWRHTVRMNLDFGKETQWP
LLLPYSNYRNKLTDEKLIRVSGIAEVEETGRSMLVLKDICLEPPHLSIEVSERAEVGKALRVHVTLTNTLMVALSSCTMVLEGSGLINGQIAKD
LGTLVAGHTLQIQLDLYPTKAGPRQLQVLISSNEVKEIKGYKDIFVTVAGAP

>human_F13A1
MSETSRTAFGGRRAVPPNNSNAAEDDLPTVELQGVVPRGVNLQEFLNVTSVHLFKERWDTNKVDHHTDKYENNKLIVRRGQSFYVQIDFSRPYD
PRRDLFRVEYVIGRYPQENKGTYIPVPIVSELQSGKWGAKIVMREDRSVRLSIQSSPKCIVGKFRMYVAVWTPYGVLRTSRNPETDTYILFNPW
CEDDAVYLDNEKEREEYVLNDIGVIFYGEVNDIKTRSWSYGQFEDGILDTCLYVMDRAQMDLSGRGNPIKVSRVGSAMVNAKDDEGVLVGSWDN
IYAYGVPPSAWTGSVDILLEYRSSENPVRYGQCWVFAGVFNTFLRCLGIPARIVTNYFSAHDNDANLQMDIFLEEDGNVNSKLTKDSVWNYHCW
NEAWMTRPDLPVGFGGWQAVDSTPQENSDGMYRCGPASVQAIKHGHVCFQFDAPFVFAEVNSDLIYITAKKDGTHVVENVDATHIGKLIVTKQI
GGDGMMDITDTYKFQEGQEEERLALETALMYGAKKPLNTEGVMKSRSNVDMDFEVENAVLGKDFKLSITFRNNSHNRYTITAYLSANITFYTGV
```

```
PKAEFKKETFDVTLEPLSFKKEAVLIQAGEYMGQLLEQASLHFFVTARINETRDVLAKQKSTVLTIPEIIIKVRGTQVVGSDMTVTVEFTNPLK
ETLRNVWVHLDGPGVTRPMKKMFREIRPNSTVQWEEVCRPWV
SGHRKLIASMSSDSLRHVYGELDVQIQRRPSM

>human_EPB42
MGQGEPSQRSTGLAGLYAAPAASPVFIKGSGMDALGIKSCDFQAARNNEEHHTKALSSRRLFVRRGQPFTIILYFRAPVRAFLPALKKVALTAQ
TGEQPSKINRTQATFPISSLGDRKWWSAVVEERDAQSWTISVTTPADAVIGHYSLLLQVSGRKQLLLGQFTLLFNPWNREDAVFLKNEAQRMEY
LLNQNGLIYLGTADCIQAESWDFGQFEGDVIDLSLRLLSKDKQVEKWSQPVHVARVLGALLHFLKEQRVLPTPQTQATQEGALLNKRRGSVPIL
RQWLTGRGRPVYDGQAWVLAAVACTVLRCLGIPARVVTTFASAQGTGGRLLIDEYYNEEGLQNGEGQRGRIWIFQTSTECWMTRPALPQGYDGW
QILHPSAPNGGGVLGSCDLVPVRAVKEGTLGLTPAVSDLFAAINASCVVWKCCEDGTLELTDSNTKYVGNNISTKGVGSDRCEDITQNYKYPEG
SLQEKEVLERVEKEKMEREKDNGIRPPSLETASPLYLLLKAPSSLPLRGDAQISVTLVNHSEQEKAVQLAIGVQAVHYNGVLAAKLWRKKLHLT
LSANLEKIITIGLFFSNFERNPPENTFLRLTAMATHSESNLSCFAQEDIAICRPHLAIKMPEKAEQYQPLTASVSLQNSLDAPMEDCVISILGR
GLIHRERSYRFRSVWPENTMCAKFQFTPTHVGLQRLTVEVDCNMFQNLTNYKSVTVVAPELSA

>platypus_TGM1
MTDDYRSDVGRWARGPWRADGAPSPSPEPTPTSTPDGRSGRSFWARCCGCCSRRSRVDESDWGPEPGPDGSGGPGRAGGSGRAGGPGSRGSGSR
RPGSRRPGSRGGTVNGAGDGTVRDGMLVVTGVDLLSGRSSQNRRDHHTDEFEYDELVLRRGQPFHLSLSLARPYQSSDRITLELLIGSTPEVGK
GTHIIIPVGRGGGGGWSAQVTKASGQHVNLRVNTSPSAIVGKFRFTVRTQSDAGEFQLPFDPRNEIYILFNPWCSDDPVFVNREDWRQEYVLNE
VGRIYYGTEAQIGERTWNYGQFDHGVLDACLYILDRRGMPYAGRGDPITVSRVVSAMVNSLDDNGVLIGNWSGDYSRGTNPSAWVGSVEILLSY
LRTGYSVPYGQCWVFAGVTTTVLRCLGIATRTVTNYNSAHDTDTSLTMDIYFDENMKPLEHLNRDSVWNFHVWNDCWMRRPDLPSGFDGWQVVD
ATPQETSSGIFCCGPCSVESIKNGLVYMKYDTPFIFAEVNSDKVYWQRQSDGSFKIVYVEEKAIGTLISTKAIGSNMREDITHIYKHPEGSDAE
RKAVETATAHGSKPNVYESRDAAEDVGVQVEAEDAVMGQDLAVSVVLTNRGGSQRTVKLHLYLSVTFYTGVTGPVFKDSKKEVALAPGASDRVV
LPVAYSEYRPHLVDQGAMLLNVSGRVLENGQVLAKQHTFRLRTPDLSLTVLGAAVVGQETEVQIVFKNPLPITLTNVVFRLEGSGLQRSKVLNV
GDIGGNETVTLRQTFVPVRPGPRQLVASLDSPQLSQVHGVIQVDVAPAPDGSSFAGARGSSNRSGENIPMVGRGEG

>platypus_TGM2
MTEELQLQRCDLELENNGRCHHTSELSADRLVVRRGQSFSVTLHFEGRSYEEGVDNLTFTVETGPEPSTETGTKARFALSAEEPVGCWSAFLMN
QDDASLSVALCSPPDAPVGQYQLTLETDIGTQGSRFPAGKFILLFNTWCSEDSVYLDDEREREEYVLSQYGIIYQGSHKFINPTHWNFGQFEDG
ILDICLHVLNLTPRPRQDTEGNGFWRQSPIYVSRVVSSMINSNDEDHGVLLGRWKEPYEDGTNPNFWTGSVAILRKWMESGSQQVRYGQCWVFA
AVACTVLRALGIPTRVVTNFNSAHDTDRNLVIDIYYDASGQRLEGQRDSIWNYHCWVESWMTRPDLQPEYNGWQAIDPTPQEKSEGIYWCGPAS
VNAIKEGDLSGGFDVPFVFAEVNADVVHWFQESDGNRRKYSSDPRLVGKNISTKGVGTDHRQDITHCYKYPEGSSEEREAFRKADRQLEEPEPE
RAGLSVRIKGSEHMDKGSDFDVSALVTNGTDGDLSARLLLCARTVSYNGILGPPCGFVDLRDFSLPARNDQTVPLRIRYHDYSPYLTESNLIKV
MALVTEPQSDSYLMAERDIYLKNPDIRIRILGEPKQYEKLVAEVSLKNPLETPLLDCCFTLEGAGLTLGQQVIHLPDPVAGGQEAKGRLALCPQ
QSGLRKLVVDFESNVLKAVKGFRNVIVAPVPK

>platypus_TGM3
MAALQPTNVSWREEANKRSHRTSKFYSSELIVRRGQPFVIALGLNRAVAAGETLTFTAVTGPSPSESARTKAVFQLSGPVARGGWSAVPEARDG
SGLTIAVTSPANSPIGQYKLSVQISSRGKVSSTVLGTFIQLFNPWLQADAVFLNSEAEREEYVLRDAGIIYVGSVNRIGMVGWNYGQFEDDVLN
ICLSILDRSLNYQRDPTTDVARRNDPKYIGRVLSAMVNGNDDAGVLAGNWSGEYTGGRDPRNWNGSVEILKEWKKTGYRPVRYGQCWVFAGALN
TVLRCLGIPARVVTNFNSAHDTDRNLTVDVYYDPTGNPLDRGSDSVWNFHVWNEAWFLRTDLGPAYGGWQVLDATPQERSQGIFQCGPSSVAAI
RAGEVQLDFDGPFVFAEVNADRVTWVYDRSNGTQRQNWLDAYSVGRFISTKAVGVNARLDLTERYKFPEGSAQERAVFKKALSQLRPTMGFSPT
ATEEAGRGQAAQVSGKFKVIGSLEVGQEVNVVLLLQNLTRDLKTVTVNRTAWTIVYNGTLVREIWRDSITASLEPEEEIELPIKLAYAQYDGHL
TADNMIRVTAVCQVTDGGEVVVERDVILDNPTITLEVPDHAKVKTPMNVLVLFANPLAEPVENCVLMVEGSGLMRGNLKIDVPSLRPKQRSRVQ
FEIVPIRSGANQLLIDFSCNKFPAIKAFVNINVSD

>platypus_TGM4
MKETDFLGSKTWAPQDNKNSGVHVTVPPQSVPVSQHLTFGGERGGEGSVEKQEPTTFEGSMVASANAEFQAATMLKTSDETNLRVLQVDFLKTQ
NARLHRTEDYNTPNLVVRRGQAFQLKLVLDRQLEDKDNVELQFCMGKAPRKSNETLVVMKLRGQKELHRWQMALLGVNGKECLLSVSSPVDTAV
GKYFLSVKSGADPVHTPENNTIYFIFNPWCEGDTVFMAGDKERSEYVLNDTGYIYVGSKDKIHARPWNYGQFEEDILDACMFLLDKSNLKPRDR
KNPVIVSRAMSALVNANDDCGVLLANWSGMYKGGTSPLAWTGSTPILQQYFRTQKTVLYGQCWVFAGVLTTVMRSLGVPSRTVTNFASAHDTEQ
NLRVDVYLNEKGQKIEDLTTDSIWNFHVWTDVWMTRPDLPQGYEGWQAVDATPQEISQGVFQCGPAPLKAIRQGDVYLTYDTKFIFAEVNADKV
FWLVKEVNGVSEMTKLREETLVIGRSISTKAVGSNTREDITAQYKFTEGSPEERKAMEKACSYLSCEDGPEAAPTEEANIELKIQAGEAVWLGD
PIRMSVIINNSSARSWTIKLGATCELQLYTGKVVAVLGSVSETVATAGKPVIEVPVHLEASSYVGSLASVDDEVIVQGHVIAAIEETEEKFSEE
VTLTFQYPPLRVEVPETVKIGQNFTCAFIFKNTLSIPLENCKLHVEGLGIFMLETFDQGDVLPGGIFKCRIICTPKKAGKKTIIAKLNSIQVKE
IYTEKMVTVTE

>platypus_TGM5
MAEGLEVILTDFQHSRNSAEHHTEEMGPGRLVVRRGQPFSITLHFGNRGFRPDADRLVFIADTGLKPESLPGTRAVFGLGEPGSPGAWTAAVEA
GNSRALEISLCPPATAAVGRFCLKIHIETTGGPVGAYRLGTFILLFNPWCPEDDVYLSSEPQRQEYIMNDYGFIYQGNKNWICPVPWNYGQFDE
EILDICLTLLDKSLNFQADPVRDFALRGNSVYVSRVVCAMINGNDDGGVLQGNWGEDYRDGVSPSEWNGSVAILRQWHAAGGQPVRYGQCWVFA
AVMCTVMRCLGIPTRVVTNFDSGHDTDRNLIIDEYYDPMGRILEDKKKDSVWNFHVWNECWMARWDLPPGHGGWQLLDATPQETSDGLYCCGPA
SVAAIKEGEVDLPYDTPFAFSMVNADRTAWLVHRGRERKLHQDAHSVGNFLSTKGARSDEREDVTESYKYGEGTVLPCTIPYLRYRGHLSPDKL
IRLSALGEERNGSKKLLVNKIITLALPGITIDVLGPTVVGRPVSVEVRFANPLEEPAGDCVLTLEGGGLFRRQVGVTIGTLGPLRGASVKIQLV
PFKSGRRQIQANLRSDAFKDVKGYKSLEVAPGS

>platypus_TGM6
MAAVKIAEVDWRREANGQSHHTAEFPGPELVVRRGRPFRLALGLSRALDPSEDLVFTVETGPRASEELRTKAVFSTSEEEEEEEEEEAEARWEA
AVEAEEEAGAPGSVGVSISSPADAPVGRYKLSARVSSRGPHRIKKLGQFVLLFNPWAPEDTVHLSSEEEREEYVLNEHGVIFRGVKERIRAQGW
NYGQFEEDILNICLSILDQSPSAQLDPLTDVSRRGDPIYVSRVVSAMVNSNDDKGVVEGQWRGQYGGGTNPLAWRGSVAILRKWYRGRYKPVKF
GQCWVFAGVMCTVLRCLGIATRVVSNFNSAHDTDRNLIVDKYVDSFGRALDDITEDSMWNFHVWNESWFARPDLGPSYNGWQVLDATPQEQSQG
VFRCGPASVAAIREGEVDLAYDGPFVFAEVNADYVTWLWQAEGAAREPVRTDPRAVGARLATKALRGDRPCDLTRCYKHPEGSKKERQVFRKAV
RKLFGVEAAGRRERVRRAGGHVPEPAKKPTVSGKFKLLEPPAVGRDLRLALGLANLTARPQKVKVNLSASTILYTRRPVAEVLHESRAVKLGPR
EEKQIPISIPYSDYMKDLTSDKKILVSAMVLVFKGEKTLLEKDVTLGDFILIKVLGPAVVDEPCTVAVTVSNPLPEAVDDCVLVVEGSGLLAEP
LSIEVPPLEAEEKATVRFDVTPYKSGSRQLQADLVSARFPDIKGFLTVHVDPARRRASSPGPPARPRPL

>platypus_TGM7
MALGRLVVRRGQPFSITLHFGNRGFQPETDRLVFVAETGPRPKRELGTQTRISLSPPDSSGTWGVAWLPETTRSLGVSLAPPADAAIGRYGLKV
EISGSRRVRSHWLGEFILLFNPWSPEDEVFLPSEPQLHEFLLRDHGLIYKGHENWITPSPWNFGQFEGDIVDICLEILDRNLSFLLEPELDCSR
```

RGSVVYVCRVVSAMINSNDDSGVLQGNWGEDYRDGVSPSEWNGSVAILRQWHAAKGQPVKYGQCWVFAAVMCTVMRCLGVPTRVVTNFCSAHSG
DRNLTIDVFYNDAAEMLPGESRDRIWNFHVWNECWMARRDLPPGYGGWQVLDPTPQETSKGVFCCGPASVKAVKEGDIQLPYDTPFVFCEVSGD
EVTWLQEAGEVKEILSHKTHSIGKSISTKMVGAAERQDVTDDYKYPEGSLEERTVYLKATRLLMGGNLPATPMYRLLQTTPGPEPPVRLKLGLA
GSPKWGQAVALQLRASRSWAALPEAGLLFLGLRFSAQAVLHVGGVQAPLWKGKAVLRLAPGEEKDLPVILPYDNYKERLLAEKVVRISCIAHVE
LTGRKLLVVKVISLDIPQLTIKVGGPAVVGQRLDVSIHFTNTQPEALTGCSLALEGSGLVEGLVSAALGTLEPGSSTVVELAIVPLKAGPRQLQ
ALITSNEIKEIKGFEGVSVAPDPTGGDPPSSL

>platypus_TGM9
MEEGIWVAHFDPNCSLNCQSHHTDMMSRDRLILRRGQAFDVFLHFQNRGWDSSKDQITFTVETGPSPCESSGTRTTFSLSETAPLHCWGAVCKA
SRHRSIQVSLLIPANACIGLYSLQAQVPSGQGPGPRTLGEFIVLFNPWCPDDLVYLENPSHREEYILNEHGMVFRGLHKYIVSHPWHFGQFEEN
MVDMCLRVLDMSGNFQRDPSLDCSYRNDPVYVSRVVNSMLCSHSSNSLMKLPRNNDSAQGVNPLAWNGSVPILSQWYGSGCRPVRFGQCSTLAA
VMCTVMRCLGIPSRVVTNFYSTQNASEAFIIDEYFDSTGRSLCGKEHIWRHHCWNESWMVRKDLNESCGEWQYLDPTPMETSGGLVCCGPTCVK
NIREGDLDQDYDGAYVFSRLNAGRASWLRQASEGKAKVHCDARLFGQSISTKGVGTEEREDITHNYKHQPDSIRGREVFYKAYRRIHPKFLSAS
NCQIEKELQALRNPGLDEADTVMKFKLGNSPVYGEDINLFLHLANLSHESRDLRLKLSAEGLTYAGCFMEPFWTDDLIISLKPKAEKKVPLQIM
YSQYGRHLGDHNLLRVVAVSEPGCKGEIMLVDRDILLLKPPVEIKLRGSPRLNVRCTAEIIFTNPLPESLRNCKLTLEGSNLMQKPVTIELGTL
APRHQTQTLVDLIPFRPGLHRLLANFDCHRFSYCKGYANVRVDNSLISLVQGPR

>platypus_F13A1
MAEVAEADRKKERARSALGGRMAVPPNNSNAEEDERPTMELTSLVPRGVNMQDFLNVTDIHLFKDSWDNNKQEHHTDKFINNSLIVRRGQPFCI
QIDFNRPYIPSRDLFRLEFVIGRYPQENKGTYIPVPLVSELQSEKWGAKIILKEDRSLRLSIQSSPHCIVGKFRLYVAIWTPYGIIRTQRNPER
DVYILFNPWCEEDAVYLEDEKEREEYVLNDLGVIFHGEVNNVKSRSWNFGQFEEGILDACLYLMDKAKMELSGRGNPVKISRVGAAMINAKDDE
GVLVGSWDNVYAYGVPPSTWTGSVDILLEYHSTGNPVQYGQCWVFAGVFNTFLRCIGIPARVVTNYFSAHDNNANLQVDIFLNEDGKTNTRISK
DSVWNYHCWNEAWMTRPDLPVGFGGWQAVDGTPQENSDGMYRCGPASVQAIKHGHVCFPFDAPFVFAEVNSDIVYIRVKQDGTHVVESVDATHV
GKFILTKQIGGDGQQDITDQYKFQEGQEEERLALETALMYGAKKPLNTEGLIALRDDTDMNFEVENVVLGRDFKVTITFHNRSPRRYSATAYLS
GNIVFYTGVAQTEFKNHTFEVTLEPLSLKKVEVAVKAGEYMTQLLEQACLHFFLTARINETKKILAKQKSVVLQIPKVNIKVRGEKMIGSDLVV
EAEFVNPLKQTLQNVSVHMGGPGVMRTAKRTFREILPNSTLKWEEICRPWTPGYRKIIASLNCDAIRHVYGELELEIQKKPFV

>platypus_EPB42
MGQALPVVRCDLHASTNNPEHRTDGIGARGLVVRRGRPFQVTLTLSATQPPCSPRIPTFTLVARTGAQAGGAEAVFPVSGHADGTRWSAAVKER
DPRRWVLSVTSPADAGVGRYSLLLQGSGKTRRLLGRFALLFNPWARGDAVFLEGEAQREEYLLNPHGLIFLGTQGGGWPRPWDFGQSDLVDFCL
LLLDVDEEGRSRGDPVHVSRLLGAVRLGEYDTAVNGHTFPASPLAGSRPGTHPGSGFPGRTRPRSSAAMCRGASWASEKGLNLIRERGIPAGSG
TEARREEASAVREPGSVPTLREWVARPPGPAHRSPDWVLAACTCSALRAVGIPARVVTAFGCARDTDGGWTVEEFYDDEGLEAAGAPESRIWAS
RVWTECWMARPDLSSGYGGWQVLDPLATEGGGERSWQDALACCDLTPVRAVKEGAVAVAPGVSRLFASLNSACAVWVRSGAGPPSRASAGAEYL
GNYISTKGVDGDRCEDVTRTYKYPEGSPREAEVLDGVWKERDRSEPRADLFVSLHAPSSFPLDGGAQLKVTVSNRSDEERAVRLTLGAQVLYST
GRLGARLWREERHLTLEGNRDRTFTTGVEFRESGRALENSSSLRLTAVGVDRSAGTCCFAREDVTAYKARATLEIPRTAVQFQPITAVIGIHND
LASSLDNCEVAIAGRGLVHRERIYRLGSVQPRGLLRKQIRLTPTHPGVLRLTARVSCGQFRNLVAFRSVNVAKAEPPA

>chicken_TGM1
MPDADPRLDAGRWAAASFRLRGAPSSDPPPRSRGSFWRRLGGCCGCCGCCRRGKEDWDPPPGEVPGRRPPQALKPGLLVPRGLVVGSRADRIAH
HTAEFCSPQLVVRRGQPFHLRVLLPRPFDPEDDSLCVELLLGPTPQVAKGTHVLIPLGETSATGWTAEEAGEEAEGEEASGSPALRLRLSAPAD
APIGRYRLSVKTRTGAGEFGAPFDDRNDVIVLFNPWCEEDGVYMEQTNDLNEYVLNETGRIFYGTEEQIAERSWNYGQFDAGVLDACLAILDRR
RMPHSARGDPVMVTRVVSAMVNSLDDNGVLVGNWTGDYTQGTNPSAWAGSVDILRSYHRGGAPVRYGQCWVFAGVVTTVLRCLGVPTRTVTNYN
SAHDTDVSLTTDIYFDENMKPLERLNTDSVWNFHVWNDCWMRRPDLPAGYDGWQVVDATPQETSSGLFCCGPCSVTAVKNGEVFLKYDTAFVFA
EVNSDKVYWQRKGNGAFAIVHVEEGAIGRRISTVGPQSAARIDITHLYKHPEGSEAERRAVSTATSHGSRPRSGAAPSRGEVRLSLSSGPAVAG
AELELKVTAHNAAPQPRTVRVRLSVCALRYTGVAAPPFRHEQHRRVVAPGGEEQLCVAVPFSEYSPHVGSQDALRLTAAAAVEETGEVVAKELR
VRLAAPDLSMTLLGPPVVGQEVSVQVLFRNPLPQKLTGAELRMEGAGLSCPASISVGTVAPEQTLRLRQPVVPLRAGRRRLVAAMESAQLGPVH
GELQFDAVPGPDGDSVEGSAATNGNTRRRRRGGGGRRRGGRAGGSTGG

>chicken_TGM2
MAEELVLETCDLQCERNGREHRTAEMGSQQLVVRRGQPFTISLNFAGRGYEEGVDKLAFDVETGPCPVETSGTRSHFTLTDCPEEGTWSAVLQQ
QDGATLCVSLCSPSSARVGRYRLTLEASTGYQGSSFHLGDFILLFNAWHPEDAVYLKEEDERREYVLSQQGLIYMGSRDYITSTPWNFGQFEDE
ILAICLEMLDINPKFLRDQNLDCSRRNDPVYIGRVVSAMVNCNDEDHGVLLGRWDNHYEDGMSPMAWIGSVDILKRWRRLGCQPVKYGQCWVFA
AVACTVMRCLGVPSRVVTNYNSAHDTNGNLVIDRYLSETGMEERRSTDMIWNFHCWVECWMTRPDLAPGYDGWQALDPTPQEKSEGVYCCGPAP
VKAIKEGDLQVQYDIPFVFAEVNADVVYWIVQSDGEKKKSTHSSVVGKNISTKSVGRDSREDITHTYKYPEGSEKEREVFSKAEHEKSSLGEQE
EGLHMRIKLSEGANNGSDFDVFAFISNDTDKERECRLRLCARTASYNGEVGPQCGFKDLLNLSLQPHMEQSVPLRILYEQYGPNLTQDNMIKVV
ALLTEYETGDSVVAIRDVYIQNPEIKIRILGEPMQERKLVAEIRLVNPLAEPLNNCIFVVEGAGLTEGQRIEELEDPVEPQAEAKFRMEFVPRQ
AGLHKLMVDFESDKLTGVKGYRNVIIAPLPK

>chicken_TGM3
MGQAAAQPSTDWHVKENGRDHHTSKFSSKELIVRRGQAFVITFNGVEQPEQTLTFIVETGPKPSKQAKTQATFGISSTVSKDSWSAVLQSTSSH
SVSVSISSPPNAVIGRYKLSVQSTSTGSSSPETLGTFVLLFNPWSSGDNVFMPNKAECEEYVLEEFGIIFAGNNYHINSFGWNFGQFQADILNI
CLSMLDRSLNYRQDPATDVSHRHDPKYLGRVLSAMVNANDDQGVVLGNWSGNYDGGKSPSSWTGSGEILQSWKKSGFKPVRYGQCWVFAAVLTT
VLRCLGIPTRPITNFSSAHDADGNLRVDEFYDASGNHLNEGADSVWNFHVWNESWFSRSDLGPSYSGWQILDATPQEESGGIYQCGPASRNAVK
EGDVDLDYDCPFVFAEVNADCMYWSYDRATRKKTLLFNKSTVIGQLISTKAVGRDDRIDITSDYKYEEGSKKERDIFKKARKKLGLEDKFDPTA
PTPKEIEQKPDISGKFKMAGPLEVGKDLNLILVLKNLQSEVKSVNVNMTAWSTVYTRRPVREIWKDSLSVSLSPEEEKHFPIKISYAEYQQQLT
TDNAIEVTALCHVEGGIQVLVQRHIALDNPTIDIQVLGEAKVNEEMDVEVVFTNPIDIEVKDCVLQVEGSDLVRGILKIDVPPLKAKDISSTKF
KLTPFETGSKHLLVNFSCDKFADIKAFKTVKVID

>chicken_TGM4
MSQDSDLKVTKVDFLKSQNSVQHHTDAYNTSNLVVRRGQPFLLQLTLSRELRAADKLSLHFSIGERPMEPTGTLMSLNPRSTRNVSGWQIAIIK
SSGTECTLSVTSAPNAAVGIYGLMVKTGPNIYKPEKNTVYLLFNPWCEGDIVFLSNEAERKEYVLNDTGYIYVGSAFNIHSKPWNFGQFEESIL
DACMYLLDKSKLKMSSRRDPVVVSRAMSALVNANDDNGVVLGNWSGKYENGTSPMAWIGSVAILQQYYKTKKPVSYGQCWVFSGVLTTVMRCLG
IPARSVSNFNSAHDTDENLRVDVYLNEKGEKLKWMSSDSVWNFHVWNDVWMKRKDLPSGFDGWQAIDATPQEQSQGTFQCGPCPLKAVKEGDVY
LPYDSKFVYAEVNADKVYWRVKEENGRNKYTKLGVESQSIGANISTKAVGQNRREDITWQYKFPEGSAEERASMKRAVSYLQPSGLTPRSRFAA
VPMEVSLRNVSDKDTVQNEVVPKSGVQLEITNEKPLCPGNPIEVTITVKSTVAGSWTVDLASSCQLQSYTGKVHANLGYVKQTVKVEGQSEVHV
PLKIMPDAYMKALATVDDEEHVHVTAIAEIQGTPEKLTKEASLSFEYPPIQVQMPETAKVNNDFTCAFIFKNKLNVPLDNCKLMVEGLGIFKMA
TFDEGDIQPGRIIKSEVICTPTRVGEKKIVARLTSNQVKDISVEKAITVTH

>chicken_TGM6
MAAQKITKVDWHSKLNKAAHHTSDYNSTQIILRRGQAFAITLNFQTTVQPENNFTFIASTGPSPAESQQTKAIFHLSEDAANGWSATQERCEPG
RMSLMIVSPANAIIGRYKLKLQMASGNKTSSALLGQFVLLFNPWCPNDDVYMANEKERQEYVLNDSGIIFQGKEKYIQQEAWNYGQFEEDILDI
SLSILDRSLNHRQDPSTDVSNRNDPIYVCRVISAMVNSNDEKGVVEGKWSGSYRSGTNPLHWSGSVTILRKWYRGRYRPIRYGQCWVFAGVMCT
VLRSLGIPTRVITNFNSAHDSNTNLSIDKYIDVSGKTLDLTEDSIWNFHVWNESWFTRRDLGSFYDGWQVLDATPQEKSKGIYRCGPASTRAIK
EGDVNLDYDSSFVFAAVNADCVTWIQHSNKRKERIYSNTRKIGKFISTKAVGTNTRVDVTNNYKYPEGSSKERQVYKKALKLLAVRSTGRRKRV
TKPRRRFPAARRQNMTATTQKPTVSGKMKLDAPPVIGQDILLTLALQNLTTDFKTVNVKLRASAVLYTRKPKAEILQWSRSVQLGAEEVKEISF
KITYTQYKNALLDDRKILVTAVCEARQGASLLVEKDIVLQDPFLTIEVLGPTVVHKATNVLVTFTNPLSEVVTDCVLRAEGSGLIKEQLNINVA
RMAPMETATAEFEIVPYKSGIRQLQVDLACIHFSNIKGFVMLDVAPAQ

>chicken_TGM6L
MAELKPTHISWQPSVNASAHHTDRYANTELTVRRGQPFTITLYFNRQKYPGESLAFVTEIGPSPSESHRTRAVFNLSEVGASGWSAAQGPSESG
YMTFIISSPANAIIGRYNLILQVNSGNKIFSRFLGQFVLLFNPWCPGDDVYIANENERQEYVLNENGIIFVGNAKYIEARGWYYGQFQDHLLNI
CLTMLDLSLYYRQDPAVDVSRRGDPKYVGRVISSMINGNDNDNGVLLGKWQGSFHSHENPSRWDGSVVILQKWRQDNYKPVQYGQCWVFAGVMC
TVLRCLGIPTRLVSNFNSAHDVDRNLSIDKYYDSSGKSLNISKDSTWDYHVWNESWFIRPDLGPRYNGWQVLDATPQEQSRGLFQCGPASVVAI
KEGDVDLDYDTLFVYTEVNADCNRWIVYNDGTKKRVYCDTEIIGRFISTKAVGSNSRVDITCNYKYPEGSPEERRVYKKALAKIFGSHITEGHT
TSSHERSSEAMRNPGISGKFKLAEPPVFGKDINLILILNNLSTDHKTVKVDISASSVLYTRRAVAEILKANTSVDLGSKQGKHIRLKIPYAYYG
KYLTTDKRIQVTALCEVMHMHGVKLLVEKTIILEDTNIIIKIPRRVVVNKAATLEISYANPLPEPVDRCVLLVTLMNQQVKIHLARLAPRERSR
IYFEFTPRRTGPLQLQVDFSCDKFSHVKGFVTIAVQPA

>chicken_TGM9
MEVIELGDVNLNCPSNCQIHNTHFFGTDRQIIRRGQAFNFYASFHNREWDDSVDQATFTVETGLRPCESNETKCTFPMGRCLDQTCWSASYKTH
QPKCINISVFPPSNACIGRYILNMQITSCGHTYQRCLGDFYVLFNPWCADDPVYLDNQAQREEYILNEHGILYEGVHKHITSRPWHFGQFEDGI
LDICLKILDMGASYHHGSDRDHCWRNDPVHVSMVVNHMISSHTTNSIMKIPENNDYLKGTKPFSWNGSVPILQQWYNGRCRPVRYGYCGSLASV
MCTVMRCLGIPSRVVTNFCFPCSNENPLGINEIFDCTGKNLCGKDKLWRYHCWNESWMARRDLNQCCGDWQCLDPTPLETGRGSACSGPTWVRS
IREGDLDLDYDGHSMFSRLNSNYAGWLSQNNAKKTKVFCDAWPCGQQLITKSVGSEQFEDITGNYKYELGSVKSKEAYYRAYRRIHPGYCNASN
CHIDRELSSLKNPFLSDSGINTRLKMANCPMYGEDVQLHWLLENLRSEPKNLKFNLCAQIITYNGCPMDQFWKDSVTVALGPREVKKIPLCVSY
SQYGPYLSDHNIMKVVAVSDPECGEVLMVSRDIVINRPPVIVKLLSQPRLKVPCTAEISFCNPLQEDMKNCIMTLEGCGLFKEPMTIDLGTLAS
NQQARTIVEFTPYRLGSHRLLANLGCHKFAYCKGCAKAEVCNHVGQNGSLPVNQNGSVPVCENGSLPVNGSGVAPAGDPGLNSTCEYICIPVYN
PNCNPGGPPVFDFVYLPAGHPLCNSIGNPGFNPNFNAIFDPGCDPGFRVICETVPPATCTQMPPTVYSSMPVTTSNPAGPPVPHVVFETGSAPT
HQPGGGGGPMPYSVSVPANNAVSALLTHFMAGSSPTTATQGIPTHLSSHPVCPPVAHAVSSSMPPPAAISVPAASLSHVVCGSAPSPIAQPLCS
SMAPLPPQPLSTPVAHAVCSPAPCPAASPLPRAARSSTPAGLHLAGSPAPRPVGPPAAHSLCSPASRPVGPPAAHSLCSPASISVGATGVRSAS
SPAPCPEDSSATCYTSPPTPQPLAAATARSLCNPASHSISRVVCTPGSRSVCGPQWGPSCNTTTTTRSGSLSVWPPTSRSTWNLTGRSGYIPFS
RGSYNTLSGPPYSSLSRSFNTLSRSAYNTLPRSTSPSAYATLPRSSSRPTGSALTRSGTHTPWSPNRSTLSYFKRKYL

>chicken_F13A1
MSEPTSTDREKKRPPLNGRRALPSNNSNAEENDVPEMDAFGLIPRGFNPKDYLRVVDIHMFKEPNEINKQQHHTDKYYNPKLIVRRGQPFQIQI
DFSRPYDPEKDQIWLEYLIGRYPQPNKGTYIPILIGDVLKPGEWGAKITHRENNSIRLSIMSSATCIIGKFRLYIAIWTPYGIIRTHRNSATDT
YILFNPWCQLDAVYLDDEKEREEYVLNDVGIVFHGDVNEVKLRSWSYGQFEENILDACLFLMDKAELELSGRGNPIKICRVASAMINSKDDNGV
LAGSWDNLYDYGVPPSAWTGSVDILLEYYSSKQPVRYGQCWVFAGVFNTFLRCLGIPARLITNYSSAHDNNANLQLDFFLDDEGQVDNRLTKDS
VWNYHCWNEAWMTRPDLPVGFGGWQAVDGTPQETSDGMYRCGPASVQAIKHGHVCFQFDAPFVYAEVNSDIIYSRMGKNGSQVIEKIDTTHIGK
LIVTKGVGNDDMVDITENYKFQEGSAEERLALETAVMYGVKKQTTQPTTYQPQKDIEMDLQVQKAVLGSDFKVTIILRNKSRNSYTATTYLSGN
IVFYTGVTKSEFKKHSFSAKLEPLLSNTFDVMITSAEYLNDLLDQASFHFFVTARINETGKVLAMQKAVVLEIPTLKIKTKGQMVVDREMSVVV
EFTNPLKQTLENATLRLEGPGVLRTMKKEFRQIPAMSTLIWDVKCIPKRPGLRKLIASLNCDALRHVYGELNIQVQKP

>chicken_EPB42
MGQGLSAKKCDLKIIMNNNNHHTEEISTERLIVRRGQPFVITVNFSSPVHNYLKQLKRIFLSVQTGPQRSKADGTQVKFPISSLGDQKQWSARL
EEQDPYFWTISVNTPANAPIGQYDLFLHASKACRLLGKFILLFNPWCRDDEVFLPNEAQRQEYILNQDGVIYSGTENEILAQPWDFRQFDVGIV
DICFKLLDIGERYQRDQDHTQRKNPIYICRTVAAMMNCNLARILSESGTRECYDGTPPSKWLGSNAILQQWAALQCRPVRYGQCWVFAAVMCSV
LRCLGIPTRVVTGFTWAHNTNSNPNVNEYYEEDGTLLTPDKSARVWTFHVWNECWMARTDLLPEYSGWQALDATCQKKSKGPSFCGPAPVQAIK
EGDVEVDYDVCYFFAAMNAKCKVWIHMADDIFKPASICTKYMGNNISTKSVGSERCEDITHNYKYPEGSLQEKKVLDKVYRKTQTLQAISSRRE
ITSIPTTLEEKVKLFIDFQSKSSLILGQDIPLSIEVFNYSDREKATDLVLGVQSLHYNGVPIMQLWKEKLNFIIKSNEVRTLQVFVPYSQYGKE
LGKNRLLRLTATLRDEDSYIYFAQEEISICDPPLTIEFPDNVLLYQPATVKISLLNPLTEPLEKCVIVVGGQGLIYRQRKYRLGTVQPKSTQDL
NISFTPTESGPRRLTAHLTCLQIQNLKSYKTINVAAA

>lizard_TGM1
MPAEDPRADTGRWANVSYRVRQPNDESEPTPRRKKRNWFRKCCSCCSGQSDAGDWGPAPGEVPGARRTDAVIREGMLVLSGIDLMCSPSSSNRR
SHHTNEYEYENLIIRRGQPFDMKLQFRQPYDPDDHRICLEFLVGPNPQVAKKTHILVPLGSPLSDLSWSAELRDTGTNTMTIRVNTSPEAVIGK
YQFSVKTRSKAGEYQAPFDPRYEIYILFNPWCPDDPVYLDKTSSLDEYVLNESGRIYYGTETQIGERTWNYAQFDHGILDACLFMLDQRGMPHA
SRGDPIMVSRVVSAMVNSLDDNGVLVGNWNGDYSRGTNPSAWVGSRDILLKYLKTGYPVLYGQCWVFAGVVTTVLRCLGIATRTVTNYNSAHDT
DVSLTMDIYFDENMKPLEHLNADSVWNFHVWNDCWMKRPDLPSGFDGWQVVDATPQESSSGIFCCGPCSVEAIKNGLVYMKYDASFCFAEVNSD
KVYWQRQSDGSFKIVYVEEKAIGHLISTKSVGSNQRQDITAIYKHPEGSPEERKAVETAAKHGTKAHIYAEKELGQDISVTVDTDEAYTGQDVS
LRVTLKNRSSMPRNVSLNLFVAVMYYTGVTGKRFKEERRQVQVPAGGNQDVPMVISYPEYKKHLVDQGAMKLSISGKVAETGQVIAKEHTFRLR
TPDLTLTLLGPAIIGCETQVQIVFKNPLGVTLTNAIFHMEGSGLSTPNTMTVGNIGPHQTVTLRQTFTPLRAGQRQLVASLDSPQLSQVHGVLT
VNVTQGPPPGPSASPASNRGSPARRRGSPAVSRASPAPTRASPAPARGSPANNRGSPAARRTGRTGRPV

>lizard_TGM2
MAEALQLETWDLLYEHNNEDHRTADAGFQRLLVRRGQSFIVTLHFSGRSFDEAVDTLTFHVETGPCPIETSGTKSSFPLSCSLEETVWSSAVEG
QDGSSLTLFVFPPPDARIGRYRLTVDVSTEGQGSSFDLGEFVLLFNPWSPDDTVYMESEEACVEYVLTQHGHIYQGSKDFIYSIPWNFGQFEEG
IVDICLQLLDTNPKFLRNQDKDCSRRNNPVYVSRVISAMVNCNDDQGILFGRWDNKYDDGVSPMAWSGSVDILQRWQKFGCQPVRYGQCWVFAA
VACTALVCLGHPPGRVVPNYNSAPQHPWEPWSIEQYLEQSGKLQQGDRELIWNYHCWVEAWMARPDLSEGYDGWQVLDPTPQEKSEGVFCCGPT
PVKAVKEGDLHLKYDVRFVFAEVNADVAYLMLQKDMSRKKTTITTTVGKNISTKSVGRDSKEDITHHYKYPEDSEEERAVFEKAQLHKDPPPAE
EGLKVKIKASEGMNNGCDFDVFALLTNNTAEEHRCRLMFGARTLSYNGALGPECGSKDLLNITLEPHAERTVPLRILYEKYGHCLTQDNMIKVM
ALLVDLDTQEAVLGVRNIYVKNPDIKVRVLGEPMQKRKLVAELTLTNPLPTPLTGCVFTVEGAGLTDRQKVQEIDSPISPGEEAKVRVDFVPRQ
SGLRKLVVDFESDKLKGVKGYRNVIIAPLPK

>lizard_TGM2L

```
MEDKSEKYVKKKKVHQTTLIGTAFHLEIAVLTEEEHVDQVSGGCWDSDAKIPLSTFSLALSVLQLAAWDLLCEHNNEDHRTSDAGSQRLLVXQP
FLVTLHFSGRPFDKAVDKLTFHVETGPCPNETSGTKASFPVSCFLEKTAWSSAVENQDRSSLTMFIFPPPDAQIGRYHLNLEVSTKGQGSSYYI
GEFILLFNPWCKEDTVYMESEEARIEYVLTQHGQIFNRNKYCIGSIPWMYGQFEKGIMDICLKMLDTSLNFLQDQDKDCSRHNSPVYVSRVVCA
MLSQSTGYICFSRQYFHCWAESWMGRPDLPEGYGGWQVLDPTPQEKSGDIYCCGPAPVKAIKEGDVHLKYDVPFVFAEVNADMVCYLRQYGMPW
KVISIDTSKTGMNISTKSVGRDTREDITHLYKYPEGSKEERAVFAKARHKVQDQVQCKDSLPLKETLKVRIKVSEGINNGCDFDVFAVIKNNTA
GKHWCELKIGTRIVSYNGALGPECRSKDNLGITLEPYEEKAIPFQILYKKYGQRLTQDNMIRVTSLLLCQDTQEYFVGMRNIYIKNPDIKIQIL
GEPMLNRRLVAELTLTNPLPAPLTDCVFTVQGAGLTGGQKVQKIDSPVGPGEEAKVKVDFVPFLSGPRKLVVNFESNKLKGVKGYCNINVAPIP
K

>lizard_TGM3L1
MTAKIDWKLKDNGIAHRTDRYSGSELAVRRGQPFGVSLTYGGNPPAVSSLTFTVETGSTAALQTKTRVAFGVTGSPPNNNWGAVQTAPAPGTMS
FSIFSPVNAGIGRYRFGIRTGGSSAPSSLLGTFVLLFNPWLQGDEVFMPNNAEREEYVLSESGVVFMGSSNSISPRGWDFGQFQPDILDICLSI
LDRSLNHRRDAATDLRRRNDPKYVGRVLSAMVNSNDDNGVVLGNWSGNYSGGENPGSWSGSVRILQQWKSSGFRPVRFGQCWVFAGVLTTVLRC
FGFPARMISNFNSAHDTDQSLTVDVYYDPAGNPLNMDSDSIWNFHVWNEAWFARSDLGSTYNGWQILDATPQERSSGIFQCGPASLVAIKEGDV
DLDYDCPFVYAETNADRVTWTYDTATGQKKKIYSETKSVGQFTSTKAVGSFARKDVTNDYKYPEGSTKERDVFNKARGKLNLSTLEATSRVPDA
PKPDVSGKFKVKSPPEVGKDVELVLLLTNLASAARTLTANMTAWSIVYTGKVIHEVWKDSLALTLGPKEEKAYPIKISYEEYQKHLTTDNMIRA
TAVCHFKDGNDAVVEQDIALENPTITLKVPGQAKVGQAVKVEVVFTNPLAEVVSSCVLLAEGSDLLEKAIRKEVSTVKGKESARITFEITPKKK
GTKQLVTNFSCDKFKDINTFQVIKVVD

>lizard_TGM3L2
MAELTPTYMNWHGSSNGQAHRTSRFSASEPVFRRGQAFHITVYMSQATQGGEAFSFVAETGQSPSESQGTRASFSASGVGGSWGASLEGREGNQ
LTFSLTSPASAPIGRWKLSLRVGQGDARLLGQFVLLFNPWCSADLVYLGDEDERQEYVLNENGIIFVGNAKYIEARGWFYGQFQKSILDLCLLL
MDLSLYHRKDPGGDSSRRGDPRYVARVVSSMVNGNDNDNGVLEGKWSEEFAHHENPSRWDGSVAILWKWAKDRYRPVQYGQCWVFAGVAATALR
CLGIPTRLVTNFNSAHDSDHNLAIDKYYDPSGKSLKIGQDSVWDYHVWNEGWFVRGDLGGSYSGWQVIDATPQERSQGLYQCGPASVMAVKQGQ
VRLNYDTAFVYSEVNADINCWVVYPNGTRKRGHSDTTSIGVNMSTKAVGSSARVDVTGNYKFPEGSSEERAVNRRALAELSGSHAAEEEVAEEA
SSRAGATAEEGVPDTSPPSPASPSSDLISQPGLFGRFRLARPPVLGGDVALVLSLANLREEPTDVTVNLSVATALYTRRTVREVLKEATTFRLQ
GKEERQLPLRITYGHYGAALTDDRKLLVTALCDVPGGVKLLVEKAITLEGPDIRIKVPHRVVASVPTTVEIGYGNPLPVSVDQCVLLVTLMGHA
VKINVAALSPGEQSSIFFEFTPRSSGAMQLHVDFSCDRFQHVKAFTLMDVAEA

>lizard_TGM3L3*
MVNSNDDKGVLMGNWSGNYSGGENPGSWSGSVKILQQWKSSGFRPVRYGQCWVFAGVLTTVLRCLGFPARMITNFNSAHDTNTNLRVDKYYSPD
GSYLEYRTNDSVWNFHVWNEAWFTRSDLGAKYNGWQILDATPQELSSNIFQCGPASLVAIKEGDVDLGYDCPFVFAEVNADEVTWILDKDTQSV
KKAATDIRSVGKYLSTKAVGSFARLDVTDAYKYSEGSSEERETFQKARDKLNMNLMAMPSMAGPAMAMPGMTISTIAMPVMDIPALLPDPPKPR
VTGKFEVKSRPEVGQDVELVLQLTNLASAASTLTANMNAWTIVYTGKPIREVWKDSLALTLGPQEEKAFPIKISYADYQKHLTTDNVIRATAVC
QVKEGADTVVLKNISLENPTVTLKMPNQAKVGEVLKVEMVFTNPLAEEISSCLLLAEGSDLLQDEIRKEVPPVKGKETVRVCFEVTPKKKGAKQ
LLANFSCDKFKDIKAFQVITVVD

>lizard_TGM4
MSQAGGPLKAVGVDFLRKENVCLHHTYDYDNCSLVARRGQLFSMKLSFNRVLNDNDNVILQLSTGDKPMESTGTLMRLNTRPKQSEKPMESAGS
LVRLNTRSKQRNQSWHANICETNGKECLIAVTSPADAIIGKYVVKVDTGASIYSCNEHFYLLFNPWCEADSVFLPDDDERTEYVLNDTGYIYIG
STKSIRGRPWNFGQFEKDILDCCMYLLDKSQLKPNARKDPVIISRTMSALVNSQDDRGVLYASWSGKYTSGTSPLAWTGSVPILQQYYKTQKSV
LFGQCWVFSGVLTTVMRCLGIPARSVTNFASAHDTEENLKVDVFLNEKGEKLNNLTKDSVWNFHVWNDVWMKRPDLPKGFDGWQAIDATPQEVS
QGIYQCGPTPISAIKKGEVYLPYDSKFVFAEVNADKVYWVVKNEGGEEKYIKLREETKVIGKSISTKAVGKNVREDITDQYKYPEESSEERKAV
ETACCYLQSCNLGTLEDPSAQDSVKSDLKLGTEGDQALWPGQPIELNIVVRNDSLGTWTINFAASCQLESYTGNVEASLATVKQTIKTEGKPVI
EIPLKIAADAYMKTLMVVEDELLVKINIIADVQETNEKFMEELYFNFEYPPLKVEMAESAKVNEDFTCAFIFKNTLSISLENCKLHVEGLGLFT
METFDQGDLAPGRIFKCKIVCAPRKTGLKKIVAKLTSNQIRGITTEKMICITK

>lizard_TGM5*
MVSNRDSAWKEVTISLAAQPLRHNGRPGAACALQRRHLTLGPKEEKELSWRIPFERYGPVLADGRQLHVTAVAEEGSSWHKALAEKTVTVATSA
LRVKALSPAVLNQSFPLQVAFCNPLSEAVGRCLLTVEGSGLLKGQMQIELGCLAPRAETSVTFHLTPFKAGPRQLHVSLTGSQFAPIKGHKRLQ
VAPAPPTGWRVRRGP

>lizard_TGM9
MEALEVKNVDLNCAENSISHSTHLFNVNKLIVRRGQPFGFYLHFQNREWDDENDKITFTVETGPKPCESLGTKSIFPLGGCPDHNHWNASFKLQ
NERCLCVDIFSPANACIGCYSLTMCLVSCGHKHSQNVGDFYMLFNPWCSDDAVYLDSQAHRGEYVLNEDGIIFKGLPKHITTHPWHFGQFQDDI
LDICLKILDTNTNFLRDPGMDCSCRNDPVYISRVLNTMIGCHKKKSVLKLPSNNNYLQGPNPSLWNGSVPILRQWYHSKCKPIRYGDCGTFASV
LCTVMRCLGIPSRVITGYYCPLNTVNPLVVQEVFDYTGKNINGKEHVWIYHCWNESCMIRKDLNQSVSEWQYLDPTPVETSKGSVCSGPIWVKS
IKDGDVDTDSDGHRVFCMLNTRNTAWVSQGRGKKTKLYCDTWPCGQCISTKGVGRDMREDITDVYKHELGSLQEKKAFYKACKKINPQYLNAPN
SEIEKDILSNRNRSLKDIGVIMKFKMANCPVYGQNVQINWVLENLCDEIKDRKFNLSAQGMMQNGSSLDQLWKENMHVTLGPKEVKKILIDIPY
DSYSSHLCDDNIMRVAAVSMPEPKGEIMMVERDILINSLPIDIKILDHPKLNVPCCTEITFSNPLKEDLKNCVLHLEGFGLIGEPITTELGTLD
AGHKAQTCVEFTPCRYGKHQFVASISCHKFCNCKGYSNVDMGSPPTTSGEGGA

>lizard_F13A1
MSMRICCHGDGEYPAFGARRGRGVPRQPKRQRGCRCTTEHLEAAIGLRALALSRPSRLCTEVSPHSWLGVECIKSIGCLLGLSGSRCRYGTDVA
KIQCFLSLCVFEDVLEVLEVRTFNREHEINKKEHHTDKYSNHKLIVRRGQPYHIQISFNRPYNPEKDTFWIEYLIGRYPQQAKGTYIKVQLVEK
LQSKEWGAKITHSDGQFVALNIMPAANCVVGKFRMYIAISTPIGILRTRRNPATDTYILFNPWCTEDLVYLDDEKQRQEYVLNDLGVIFYGDPE
NIRSRSWNYGQFEEGVLDACLYVMDRAELDLSGRWNPLKISRVGSAMINAKDDEGVIVGSWSNVYDYGVAPSAWTGSVDILLEYHSSREPVRYG
QCWVFAGVFNTFLRCLGIPARVVTNFCSAHDNDANLQMDVFVDENGKVDSKLTKDSIWNYHCWNEAWMSRPELPVGFGGWQAVDSTPQENSDGM
YRCGPASVQAIKHGHVCFPFDTPFVFAEVNSDVVYSKAMQNGVKLVQHVDKTQIGRLIVTKEEGTDKMKIITEQYKFQEGTEEERLALETALMY
GVKKSIPQDVTSTLEVNMDFVVEDPTLGSDFNVTITFQNNTQSRYTATSYLSGNIVFYTGVSKNEFKNHSFNVTLEPMKAQTVEVLIKSSEYMS
QLLEQASLHFFVTARVNETQKILAQQKSVALKIPQLLLKVPGEKVVGKDMAVIVEFTNPLKEDLNNVWVRLDGPGLLKPTSKLFREVPRNSTLT
WEEKCLPKRAGLRKLIASLNCDALRHVYGELDINIQSAV

>lizard_EPB42
MGQALQVERCDLRVAENHEAHRTGALSGGRLVVRRGQPFALRLRLSGPVSEKQRKSLLGTTAASLLVTRTGAAGGALEQAFPIGRRGSDWEAVL
EAHDDLEWTLSVTPPADAPIGLYALTLQLPSGTLAPPSQHPLGHLALLFNPWSPGDSVFLGNEAQRQEYVLSEEGTIFWGLEEAPQGIPWDFGQ
FSEGFLDMSLALLDISLLRMPAQDACDQLRSPLQVCRLVSAMLNGEKPFPVLEGCWSGEYGNGTPPTKWPGSGPILRQWLSGRCRPVRYGQSWT
FAAVACSVLRSLGIPTRVVSAFAWAQGTEGSLHVDESFDESGATIPGDSDARIWLCHAWNECWMAREDLGQEYSGWQALDTTPKKGLQSGLLCC
```

GGPAPVRAIKEGRLDLPSGVGPFFAAINSTCTAWVCKASGESEKATSEVKFLGNCISTKAMGSERCEDLTQHYKFPEGSLQERAVLMKVCQDTE
PPTLFPPLDETEETPLAAWFECESSLALGEDVQVSLRVANWTGKERPLRVVLGAQPLQGNGETLAQFWKEEFMVTLPDGHEKVLSTTLPFVQYS
PALRKGSLLLKLTALLREIPSDSDAGQSRRAPLLAWQEVLLRPPEVTLQIPKSLTQFQQTEATIQLHNHLPEPLTGCSITLSGRGLIYKERSYR
MGSVPPGGSLRLRLPFTPTLAGDRRLTARLDAPGFQHHHSCASVTRVTAP

>clawed_frog_TGM3L
MRSDPQVIACDWYYAVNEKAHHTDVYESDDLVLRRGQPFKLTLMLNRPLQAEENILFIFETGPSPSETSKTKVVFPLFRAENLQSWGAILTSIK
STSITVTINSHSDAVIGRYILSVAVQCRGSDKPLPHQIGALNLLFNPWLQGDAVFMAEEDQRQEYVMNEQGVIFQGSDEDITSINWEYNQFERN
ILDICLVILDRSQNYKKDPALDVSQRNDPLYVCRVLSAMLNSKGDDGVLEENWSDDYINGASPSSWNGSMNILKSWYYSKFKPVKYGQCWVYAG
MLCTVLRSLGIPTRVITHFNSGQDKNANLFIDLQYKSQGSRQNDQEDQLWNFHVWNEAYFKRRDLGKSYNGWQVIDSTPLKRSDGVYQCGPAPL
TAIKEGDINLNYDVKYMFASVNADVACWIYYRNGTKKQVSNNAKETGKFISTKAIGSNDRVDVTNNYKYAKGSEKEIKVFEKALKLSKGSFVGR
TREKPRGGNILFGRCTLDQIATFGQDFNLILSLKNLTPDSINVTVYINTSAILYTGRQRHTIWTGGKFLSLGPNLEKRFSIPVKYGQYSKHMTD
NGVICMTALCEVEGTEERILVERNVSLVKPPLSITLPDKAIINQESNANIVIVNPLLETLNSCILWVEGYGLTDKILKKEVPSLKPGQSSEHNF
VITPMKTGVRTLLVNFSCDKIQNMKGSGKILITYSQEVSDSP
VPT

>clawed_frog_TGM1
MARCEERKKSFWERLCPCCCTERSQYEPDNDMRPVNRPDGGSRDNRPDGGIGDNRPDGGSRDNRPDGGSRDNRPDGGSRDNRPDGGSRDNRPDG
GSRDNRPSVNPPSPSLTPSIGGPLQVQNVDLLKQRGGETRRAHHTDEFEYDELIVRRGQPFKICVTFQRAFNPKSDRICVVLQMANAQPITLSQ
VNEFDESGWACQVAEVNGCKSNIWLNTSAQAPIGLYQMIVKTNSGEGPSNLRVYILFNAWCRMDSVFMDNEAWRQEYVLNEIGRIYYGTQNQIG
ERSWNYGQFDKGVLDACLYLLDCGRIAPGSRGDPINVTRVISAMVNSMDDNGVVAGSWSGDYADGVNPVAWVGSVDILLRYHQSGSSVKYGQCW
VFGGVTTTVLRCLGIPGRTITNFASAHDADGNLTFDIYFDENMKPIEDKNRDSVWNYHVWNDCWMTRPDLPAGYSGWQAIDSTPQETSNGIYCC
GPCPLLAIKNGLTNIKYDAPFIFAEVNSDKVCHQRMPNGQFKRVLVEERAVGHCISTKAVGSFARDDITYLYKHPEGSTEERNSVHTAARYSNK
PLAAVDNEETSDVSMSVESQDGVITGSDINVRVLLKNNSNSRRCVSLSLTVGVMYYNGICKESFKNETRDTTLNPGEGKAVGMLISYAEYSKHL
VDQGAMMLTVSGIVNETGQKLAKQHTFRTRTPDLVIKVRGDAIVGQQIVAEVIFKNPLNTTLHNAVFHVEGPGLQRPKVIKYGNIGPLQTVCVS
ERFTPRRPGPRQFIASLESNELSQVHGTTEVVVQPC

>clawed_frog_TGM2
MAEELYLESFDLDCSGNNRSHRTAEATCERLIVRRGQPFQITLNFSPRGYEEGVDKLSLNAVTGPCPSEESGTSNHIPVSDALQDGAWSAAITS
TDGGTLILSITSPPDARIGYYNLSLETSTEYQGSSFQLGSFTLLFNPWCPEDSVYLETEEERKEYVLCQHGIIFQGTKDSVEHVPWNFGQFEDE
ILDITLQVLDTSPKFLNDSNRDCSRRNDPVYISRVISAMVNCNDDRGVLFGRWDNKYDDGISPMFWMGSVAILRRWRKFSCQAVKYGQCWVYAA
VACTVLRCLGIPARVITNYNSAHDTNSNLLIEQYLDEHGKRQPKQKEIIWNYHCWTEAWMTRPDLGEAYNGWQVVDPTPQEKSEGTYCCGPTPV
KAVKEGDLNLKYDVPFVFAEVNADVVYFVQQNDGSVKKTQFISLVGQKISTKAIGKDEREDITLNYKYPEGSEDERRVFEKANKQSEAEEERVE
TPSDFTIKIKVSEGMNKGSDFDVFAVITNNREEEKQCRLMFCARTCSYTGEVGPECGMKDLLNLTLTPQEEKRVPLRILYEKYGPTMTENNTIK
LVAMLYDYSSKEIILAVRDIHVKNPSIKIKVLGEPKQKRKLVAEISLKNPLAEPLTGCCFTVEGAGLTAEQLVKTLDCPVEPGQEAKVRVDLMP
QLAGKLSLVVDFESDLLKAVKGYRNIIIAPLPK

>clawed_frog_TGM3L1
MTFPPELSGIDFDLLKNVQEHRTQGIRGMGLVVRRGWPFTMKMQFRYLGQDAKLENLNLIAQIGPYPSPHTGTHIYFPVSRLRDNRSWSAEMEG
ENNGVLAVKVQTPATAIIGRYSLSLEGSYRGMPVSHILGNVVLLFNPWCPEDDVFLNDEAMRQEYVMNEHGTMYQGTKDFIKNIPWNYGQFEDG
VGETCLRILDMSPSCLKDPATDCSKRGDPIFICRVVSAMINSNDDRGVIESSWDDLVRGGVEPSSWNGSVAILRQWHTSGCRPVKYGQCWVFAG
VLCTVMRFLGIPTRPVTNFESAHDTNCTLTVDEYYDETGKQLETLGDTIWNFHVWNECWMARKDLRSGYDGWQIVDSTPQEISGGTYCCGPAPV
KAVKEGDMDVSYDVPFVYAEVNGDVIHWVVNEKGIKQGQVDTIAIGKNFTTKRVGSNDKEDVTSHYKHAEGSPQERTVFAKATSAVKSKRPFSA
KESGMASDFEVSLKLAETPMIGQPIVLYCRILNKSFAAKKLSVNMSAQAMQYNGTPLDQFWRKKFDVDVFPNQATDGQFPIHPLQYQQFIRPGN
SIRVTVLATDLQSKQFRFAAQNVVLKEPTIDIKIYGTPQFNRPLNVQLSFQNPFNEVLTNCVMSAEGAGLLPNGPAEIYMGNIYSLRAGSVGLV
CVPIKRGKLQLEVSFSCNKVQHIKGSATIMVAAF

>clawed_frog_TGM3L2
MAGFKVLSADLQLDANKSAHNTSAYISRELIVRRAKAFGIGLNFNTSVNAEDRLQFTAGLITSSASVSLLEFNFSDSRYPAINSWGAQRVATGR
NSVTVNFFTPPDAVIGRYFLSLLTSEGRTRFASFVLLFNPWAQADVVYLSDDAERQEYVLSEFGLIYLASNPNPIAWNFGQFQENILNVSLALL
DSTSSFRSNAAEDVRKRNNPVHVCRVLSSILNSMNNNGVIEGNWSGEYFDGTDPYVWNGSTEILRSWYGQRQPVKYGQCWVFAGTSCTVSRSLG
LPCRVITNYQSAHDTDHNLSIEQYFNTRGEAVPRSEDSIWNFHCWNESWFLRLDLGDFYSGWQVWDSTPQEKSDGLYQLGPTSQRAVKEGEVDL
LFDTPFVLAEVDADVIIYIVQDDGTVTKAGKRENDVGQLICTKAVGQKAMNDVTLEYKYKDGSSKERETYEKARSKIRGSEFEAMSRALVSSPP
PPEVTGTITVAGTPTVGDDINVTLTLKNMTSDNKTVTVNLSAAAIVYNHAVRKPILSNSAAVQLGPNEAKGVPVQIKYSQYEKQLTSDKMIYVT
AVYKVEGKPEKFVEANIVLLNPTLQVKALKTAIFDSPMVVEVTFKNPLSTPITGCVLKVEGSGLTKDVIEKNMGQLEPGQKTSVKVEFIPYMVG
DKELLVNLACDKFKDIKGHLSIKVQSSSSRLRACCF

>clawed_frog_TGM3L4
MEESNRKGHHTEMYNSPELILRRGQSFWITLDFDRPIQEWESIVFTAQTGPLNAKFYNINVEFPLSNSWSSGRWSAVLESAPGNSLRIIMSSPA
NAVIGRYNLTVQICIMGNTSTYSLGKFILLFNPWCLDDEVYMANEDERNEYILNDNGIIFIGSDKHIASLAWNFGQFESNILNICLDMLDRSLN
YRNDPAADCSKRNSPMYVGRVISAMINSNDDYGVLEGKWEKEFSDGVDPNSWTGSVEILLKWQKEAYQPVKYGQCWVFAAVMCTALRCLGIPTR
VITNFNSAHNTDGNLCVDLHYDNDGKFMEISDDSIWNFHVWDESWFLRKDLGQFYGGWQVLDSTPQEQSQNVYRCGPTSVNAVKEGDVHLPYDT
PFVYSEVNADRVTWVCHKDGRKEQAHSDTKAVGQFISTKAVGSNERVDITHCYKYPEGSPKEREVFDKANRKLSNYRPPTDKPNDVNNTRDRRA
TNEVLMDNAENSRPRSNTLPSRPTANTLRPSRPTPNVPLSRPMSNSVASIPSAVDWPSRPRTNTMYSRGIANGFSSRSTERLYNPRTPPLNTFP
ERPPPLNTFPERPPPLNTFPERPPPLNAFPERPSLDGPDIAGKFKLLGPLAVGDDINLLLSLRNLTPYHRQVIVNLSASCILYTGRRINDIFQD
QKSLIINPSQEGHISLQIPYSLYGNFLTDGNMIQMVAVCELPFRKKVVITKELVLDNPPISIKPLEMAVLNRKMIVEVRFTNPLSFVVKDCTVA
VEGAGLIDRQLTAVVPYIKPKQNIKFKVELTPFRSGTKQVIVHVKCRYFSIKGHHLLNVVSL

>clawed_frog_TGM3L5
MEESSNMAEHRTNYYSSSDLILRRAQAFRIMLYFNRPLREKDKVEFTAATGPDPQEADDTMCIFPLFGSQSKASWTAEVDSIDSNCVTAIITSS
ADAVIGRYKLQLYTTSSKKKSYFKLREFVLLFNPWAEDDVVYMEDENERCEYVLNDHGIIYFGHEEMIDEQGWDFGQFEENILDISLQILDRSL
NYQDDPVLDCSQRYDPGYVGRVLTAMINSFDDDGVLEGRWTGKFTGGVDPQHWIGSVEILMRWYRGGYKPVKYGQCWVFAAVMCTVLRCLGIPT
RVITNFASAHDKDGNLGIDSIYSSSGRNMSKDTMWNFHVWNESWFRRNDLGSAYRGWQVLDATPQELSEGTYCCGPASVHAVKEGDVDKDYNVP
FVFAEVNADRNTWVYYAKDVKEKVYTDSKSVGKHMSTKSVGGNERVEITNNYKYPEGTEKERQVYLKARKKLLDMGVLKDENLGRRFIGKKRYK
KRGRASESADDSEEPTELGIIGKFQLVAPPKFGDDVNLILSLRNSGQKSEALKVKLSSSAIKYTGRPMSEIFSDQTSVTLGSMKEKQIPINIAA
SQYEEELTKDHLIEVVALCELKSKKKMLVRRVVSIEKPPLLIQVLSYPVVDELCELQVSFKNPLSVPLTDGILLLGGSGLIRKQIKRRVPKLGP
KAEGSIVLEITPYRFGTQQLVVDFISKHFSAIKGFKRIEVAEDHLEDISEEIIVIEDD

>clawed_frog_TGM3L7
MTSLVLKSIDLQQKSNAAAHNTSDYVSKVPIVRRGLPMKVTLAFSRALQSTESLALVMETGPSPSASTNTRIVMPVSSSRRNDTWSATLESSSA
GTMTVSINIPVNASIGPYKITAQITSAGRTSTNDVGRCSVLFNAWASGDEAYMSNDAERKEYVLNETGLVFYGSAGFPSSSAWDYGHFEDGVVD
ICFKLLQESPEYKADPATHISKCNDPVYISRILSAMVNSQDDRGVIVGNWSGDYSGGENPSSWSGSVALLQRWSQSGPVRYGQCWVYAGVLCTT
LRCLGLAARLITNFESAHDTNNNLIIEQYLDAYGRSIGSPDSVWNFHAWVECWFRRKDLGSTYDGWQILDATPQEPSGGSYRLGPTSQKAVKLG
DVNLPFDGPFVLAEVNADEIYYGRRNDGTFSILYTDTRKVGQYISTKAVGNFSRQDVTDQYKFPEGSKEERDSLHKAQGLTARSDRVMLASRRL
NATAARVVEDKPPIQEAPKPEIIGTFKLSGELQVGQDFTVTLNLKNPTSSKKQVNAKWTLTAIVYNRTPVKEILSDSQSVTLAPNEEKVIEITV
LYPQYEKALTPDNMIRASAVCVEENGGSLLVDKVITLKNPPLQITAPERVTWGKSATLGIVFTNPISEDIKNCVVTLQGSGILKKTLKVPLPDL
KANQRIRAEAEVTPYRNGKRVVIAGFSCDKFSDVKGYQTVDVMAA

>clawed_frog_TGM3L8
MSALQLKSINLQKATNVANHHTSGFDSTVFALRRGQPITVNLNFNRVFYTGESVAIDVETGPSPSVPKNTKAVMPVSKYANPQTWSASSGQSTG
ATMPVIINIPVNAIPGRYKMTAHLARSGRTTASYRLPEFFVLFNPWNQDDQVYMSNEAERNEYVLNEYGVIFRGNANNISRLAWDYAQFEGDML
DICFLMLDSCLEHKTDPITDISNRYDPIYVGRVLSAIVNINDDNGVLYGRWDNDYSDGVSPTSWSGSARILRSWRDNGPVKYGQCWVFAGVLCT
VLRCLGIPARVISNFESAHDGNSNLLIEEYFDEKGTQLGSPDSVWNFHCWNEAWFTRGDLGDNYGGWQILDSTPQEPSGGIYCLGPTSLKAVKQ
GDVTLDYDTTFVFGEVNSDRKRFIKYSDGRPLQNVYTDTASVGQFISTKAVGSNNRQDVTNGYKDPEGSGKERETFNKARTTLLSLGIATMAAR
SVQEEPKGPKPEITGSFKLSGQPQIGDDVVVIFNIKNPTSARKNIKLKFTVTAIVYNRAVSKEILTNNQPVTLAANKEESVNLTVEYSKYQGAL
TPDNMIQVVAVCEEENGAALLTDTVITLKNPPMQLKVPDKAAVNKALSVEVIFQNTIGDRLKNCLITLEGSGLIKDEVKVPVPDLKPSEKYSTK
VVITPYKSGERNLSANLSADKLSDVKANLTVKVDAA

>clawed_frog_TGM3L9
MALQLKSVDLQQKSNAAAHNTSDFITKSLIVRRGQTFTIQLDCSRDLKDGERLDLCVETGPSPTKDNNTQAVMQVSSSGTSSSTSWSATLGSRS
GGTLQVIISIPVNAVIGRYQMTAQLTASTTSSFPVGDFIVLFNPWASGDEVYMSNDEERREYVLNETGFIFLSSGRITWDYGQFEEDILDICLL
LLDRSTEYRRNPVSAVAKRYNPIYVGRVLSAMVNSNDDNGVLVGNWTADMSGGEAPSSWSGSGTILRRWSQNGPVKFGQCWVFAGVLCTVLRCL
GLPARVITNIESAHDTNRNLVIEEYYNEDGTNIPSPDSVWNFHAWNEVWFVRKDLGSTYDGWQILDATPQELSEGIYCLGPTSQRAVKLGDVNL
NFDGVFVFSEVNADKKTYVKYKDGRTVLVHTDTTSVGQTISTKAVGSSSRVDVTNDYKHTEGSSEEREVYFKAQRQVAESSTGITALSAASSVK
KVAYAPKSQIIVTLKMSAQPQVGQDVTAMLNLKNASSGHRKVKVNWTATVIVYNRTPVKEILKESQAVSLTVNEEKAIPLKILYSQYKDTITTN
NMIHIVAVCHEEKGGCLLVNKIIILKHPPLELKVPFIL

>clawed_frog_TGM4
MALKTEKVDFLKTENTSQHNTNDYDNPNLILRRGQEFHMKITFDRELTANDKVTLQFTTGSLALPSNGTLVLIDVGPVIQTNQWSALIRQRNNK
EYLVAVSSPATAIVGKYMLSIITGKGIVYPLADCTIYLLCNPWCKDDTVYMPSEDGRKEYVLKDTGYIYVGIATKITAKPWNFGQYEAEVLDCC
MFLLDHGQLKPEHRRDPVILTRKLSALVNSNDDRGVLTGNWSGNYSGGFSPTSWTGSSVILQKYYKSKRSVMYGQCWVFSGILTTVLRCLGIPA
RSVTNFNSAHDTGGNLKVDVYLNEKGEILEDLCSDSVWNFHVWNDAWMKRPDLPKGYDGWQAVDATPQELSQGVYECGPCSLAAVKNGDVYLPY
DGKFVFAEINADRICWLVKDKQGDEPPIQIRQEKSCIGECISTKTVNMNVREDITVQYKHPEGSPEERETFQKACSFLNSGACLVSPEPPPYPP
AGTKLQIQGDKELIPGNPLSFTVSIENETNEAKSLDITIGCQLQAYTGKVIASVASIKQSVQVPGKKVAYIPVTVASEQYMKSVIMVEDEAIFR
INAITENKETQEKTSDSMAIAFTYPPIKVEMPETAKINEDFSCTFTFKNTLSIQLDKCQLHVEGLSMFKLETFDEGDIKPGGIFRSKIICAPRR
PGEKKIVAKLISSQIKGISVEKTIIIIN

>clawed_frog_TGM4L
MTLRIISLDLLKTENRSHHHTSTYTNTSLILRRGQEFKFKITFSEELTSDIPIELHFSTGTTPLLSNGSLVLIKVNAESTKKQWRAKICESNGN
EHVVEVCSPANAIVGIYSLSILTGSTACFYSTDHIYLLYNPWCEDDDVYMPNEDERQEYVINNTGYIFRGSYKNIKAKPWNFGQFEDNVLDCCM
YLMDIGGLKPSSRRNPVIISRKFSAMINSNDDNGVLTGDWSGNYPDGTAPVAWTGSSAILKKYYKEKKPVLYGQCWVFSGVVTTVMRCIGIPAR
CVTCYSAAHDTEGNLRVDAYYNESGEPLSHMNKDSVWNFHVWNEVWMKRTDLPQGYDGWQVIDATPQERYQGIFQCGPCSVTAIKKGHVYLPYE
GSFVFAEVNADKTNWMVHEYNIKGPHTLLKEERSSIGKFISTKKVNKSLREDITSQYKFDEGTEEEREAYQRACSYLNADNPSSCERSAPAVQG
IKVKIHRGHELYPGNPLNALVEVTNITDGDKTVDVIISCQLQTYTGRITANLTSARKTVEIKKKQVASIPVTIEADLYMKSVMQVDDELAIRVN
VITEIQELRERSFKSRIIHFHYPPIEMEVPETVKTGQQITCRFLFDNTLNIKLENCEMNVEALDIFHLQRVAEWDLNPGEALSFRIKCRPKITG
EKKIVADFISTQIKGITMEKIIHVTN

>clawed_frog_TGM5
MTAEIAYSDFQIKRNNKAHHTDETGDRRLFIRRGQPFFITVHFKTRGFQPGQDNVIFLVETDDDVFMGNEAERQEYVINDYGFVYQGNHNWIIP
CAWNFGQFEDDIVDISLKILDKNLNYIQDSFKDLKNRNNPVYISRVICAMINSNDDNGVLQGNWSGDYTNGVSPSAWNGSILILRQWYKSDCQP
VKYGQCWVFAAVMCTVMRCLGIPARVVTNFDSAHDTDQNLLIDEYYDTTGKKLSKETKDSIWNFHVWCECWMARRDLPPGYGGWQVLDPTPQEA
SNGIFVCGPASVKAIREGDIHLNYDAPFVFAMVNADCVSWIVSTKGKEKYHCDPHLVGTQISTKRAGSDEREDLTHQYKYEEGINETAEERKAF
EKAISRLHKGGAIPNINESEHMNGANGTSANGITPSPDESSRYTPQNENSNLNSPLREALLLLKFKLTESPQLGETISLVLLAGNMVSTAKTLK
LSLSAQAMKHDGKPAQQFWRDSMYVELGPHEAQTNSLWIVIELPKSLCKVTIPFIPFQLTQENEEGNLSPMFFYTEKWLLFKIPYAKYGPSLED
NHLIRFIAVGEQNITWEKVLVEKDVNLALPAVVINFLELAVVDKTCKAELSFSNPINEAIHDCQILIQGSGLLKKQVKLNMGTMKPGQKAVVAF
EFVPWKVGYKQLQVNISSNRFKGLKGFKSVVVKP

>clawed_frog_TGM7*
MSSSFPLSTATYISGAFKVTGERVVGKDVTVTLTITNLTCNSKSIKVNITAFSILYTNKEMHQLLNETRTLCLKGKEEKEVPLTIKYEQYEDLL
TADNMIEVHAVCSSEKTNEKLVVETNIALENPKFEIQLVGKAIINKSTTVQIIFKNPLNKDLTDIVVIVEGSGLIKEPITIQGGSVEAKDSITI
PVTFTPYKEGEKYVLADFTCNKFKNIKGHLQINVQAE

>clawed_frog_F13A1
MSEAKKEKKKSSVPGHTGRSSLPPNVSNADEDEVPTMETFGVIPRGVSMKDYLEVQNIHLFKQTNEKNKREHHTDRYYNNKLIIRRGQTFDIQI
DFNRNYNPEKDQFWIEYVIGRYPQQSKGTYIPIPIVDKLEAGKWGAQVTRNEYNSVSVTILSAPDCIVGKFRMYVAVLTPYGIMRTSRNSETDT
YILFNPWCKEDSVYMDDEREIEEYVMNDVGIIFHGEVSNIKKRSWEYGQFEEQILDACLNLMDRAQLDLSGRGNPIKISRVGSAVINSKDDDGV
VAGNWKNDYSFGVSPSAWTGSVDILLEYHSSGLPVKYGQCWVFAGVFNTFLRCLGIPARLITNYFSAHDNDANLRTDVYLDEDGKTNTTLTKDS
IWNYHCWNEAWMTRSDLPVGFGGWQAVDATPQETSDGMFRCGPASVQAIKHGHACFQFDTPFIFSEVNSDIVYSRAMKDGSKVIEHIDKTHVGQ
KILTKQIGGDDPQDITDLYKFSEGTEEERLALEAALMYGVKKEIRDEDQQQFAPDTDMDFQADNAVFGSDFKVTLTFKNWSANLYTVSAFLTGH
IVFYTGVLKSEFKNKNVEVTLDPSSSKSVEVLIKAHEYLSKLVEQASLHFFVTARINETKKIMAKQKIVILKVPELHIKVLGEKMVGKEMTIVI
EFTNPLKKALQNVALRLGGPGLMRTRAKVFGEIPMNSSLTWEQKCIPQRAGTRKLIATLDCETIRHVYGELELEVLDNPL

>clawed_frog_EPB42
MGIINMHDLPFVTCDLQVSKNNKNHHTIDLSKENRLFLRRGQEFTITLRFHSPTRLHYKQLEGITLTTKTGPNPSKKNGTKNSFPISSLSDNKF
WSAKVVDAQQGLWTLSITTPASAIIGNYTLSLKSKKPTSLSQDLGRFMLLFNPWCKDDPVFLHNEGQRQEYVLNEDGIIYMGTESCIQQHPWHF
GQFEYEIADVCIKLLDMNPKYQKVPKNEYLNRNDPIYVSRVLGDMISRKDEEDRVAFMVENRRPSYGLISSVPILQKWFQNEFKTVYGHHWVFA

AVLCTVLRCLGIPTRLVTNYNSAYDTNRTLQKEIYYDEKGARIHRARSDSIWNFHVWNECWMERRDLSPEYNGWQVLDATAQLKSSEALCVSGP
APMRAIKEGHVDLNYNVDLIFSKLVTDNMVWVRNPKCFSKVLCWARHVGDSISTKSVGSDMQEDLVHHYKYPKGSAEETEVLRIVTQTMLKNHQ
VKDSKETSLSPATVSISSQNLQMYGEDIHFKVTVANVSWEERDFKMLVGAQPVHDHGFTQAQFWKEEFAFHFKPYEGCNLSIHLDHSSYEACLL
DNNLLRITALVKDPTCENDNVALAEQDMTICKPCLSVQAPRVSLQYQPMTAMIHFTNPSEKTLLECVLRASGKGLLHSERQYRCGNVAPRGTLR
YPITFTPTQVGPRRLYVQLECSIFRNIIGFHQFEVLPANIQEWSNEHWQVFQKASESTRAVGHMDPVLSLDIQLEESVFYGQDVTIKVQLSNHS
KTKKDVCLILYAQYVHRNGNFCPNFWKQEHTISLQAHEEKTVFTCIIPSEYGEFQWESNLIRLTGLAKDVTSMISASRHVTLYKPNLCIQMQTE
ALQYQQITSIISITNPIQETLEDCVLTVSGESLIYRERLYSCKNIDPGSTEAFKIRFAPTQTGPLKFQVRFDCKQFCDVRRSQEVGVLPCTNPF
LSG

>lungfish_TGM1
MSYANGDATNSRRIGRQQSSASASLAMRQNSVTPSRKKKDAWWRSRWCCPCLRARSSEEEVLDKLTDGKKDEEGDKEEVELNDMMLIVKKIDLM
KKKKEENKITHHTNEYDYDELIVRRGQPFDMKLQFNRPYNPETDQIFLELQTGKHYPQVTKGTLIIVPLVEEHNRMEWGAKVTQASGNHVTLSV
NSSPEAVIGKFQLSVRTRGPGGELKTPSAPENSIYILFNPWCEKDSVFLDNEEWRKEYVLNETGMIYYGTEFQIGARTWNFAQFSKGVLDACLY
VLEKGGMPASGSKDSVSTARVISAMVNSPDDDGVLEGNWSGDYSDGTSPTSWVGSEDILLQYHKTRKPVCYGQCWVFSGVTTTVLRCLGIPSRS
VTNFSSAHDTDVSLTMDIFFDENMKSLEDMNYDSVWNFHVWNDCWMARPDLPPGYGGWQAVDATPQETSSGTYCCGPASILAIRNGLVFLKHDT
PFIFAEVNSDKIYWQRQADGTFKKLFIEKKAIGHKISTKAVASNEREDITSLYKHPEGSEEERIAVETASKYGNKPSIYIDSDMPSDVGIEVQT
DEKTTMGSDVVLCVGLTNKSNEQRFVTLFINTAMMYYTGVLKESFRKEEKAVQLSPYEEARVEMVIKYAEYHEELIDQAAMMFTISGRVQETGQ
LLATQQTFRLRTPDLEIIPIGGAVVGQEMRAKISFVNPLPKTLTGVYFRVEGPGLHKPNIVQVGDVARGATVTITQTFVPVKPGPRKLIASLDS
RQLTQVHGVAEVDVKE

>lungfish_TGM1L
MLSVKSINFLKKPNEANRLSHHTEKYVTEDLPVRRGNNFQIKIECNLSFDVKTDHFQLEFVHESKNPALKTERPIVVYIKEQLDKKNWGAMVVG
SSGNYVTLSVYSPPNAPIGKFILRVKSKRSPGDFSTYSDPKTHICILFNPWCPDDTVFMNNEQWRQEYVLNEFGIIYRGSVNQNSSMKWSYAQF
AKSIMDACYYTVDRAGLTLSERKDPVVVARKMSAMVNALDDEGILVGNWSSDYSGGTPPTLWIGSEAILLQYYKTGRPVRYAQCWVFAAVVTTI
LRSLGILCRTITNFPSAHDTEKNLTIDVYLDEEFKPLSEMNNDSIWNFHVWNEIWICRPDLPAGFNEWQAIDATPQEKSDGIYCCGPAPVKAIK
EGKIDILYDSDFICSEVNIDKVYKQKQKNGTFSSVYVERNVAGIKILTKAVGSNGTQDLTNQYKYPKDSKEGMSAIASATHRLVQPRPATESTI
PNSVEITACTDGTTDIGSPISFHVKLHNKSQEQYTGILHIHASMMQYNGAIQDPFRIDENKEVVLEASQETDIEMAIEYEEYRDYLTEQACMMF
TVGGQVLETRQPLVLQSTLSLRKPGLVIKTVGVVAVGKESKAEFIFKNNFPFCLTDVLIRIAGPGLFLPIVLNVGTIGEESNSVTGYTFIPTQA
GPRKLLASLHSNELTDVTGSVDIDVVP

>lungfish_TGM2
MASVLVMERCDLECETNNKRHRTSDFGIHCLVVRRGQAFRITVYYKGRGFQQHTDTLTFTAQTGPCPNEVSGTKSQFPLTNSLNEEAWSAAVEQ
NDNSALTLKICSPPDARIGRYTLNMEATTAGKGVSIPLGQFILLFNPWCSGDSVFMDDEAKLREYVLTQDGLIYTGTDKRINSMPWNFGQFESG
ILDICLDLLDKSKTFQNDKDKDCSRRNNPVYISRIVSAMVNCNDDDGILMGRWDNNYGDGISPLQWMGSVKILRNWQNQSCQPVRYGQCWVFGA
VACTVLRCLGIPTRVVTNFSSAHDTNGNLIIERYLDETGKEIKKSKDSIWNFHVWDECWMARPDLGKGYDGWQAVDPTPQERSEGVYCCGPAPV
QAIREGDIKVKYDLPFVFAEVNADVIYWMQRRDGKDEKVVHSSVIGKSISTKSVGSDAREDITYHYKYPEGSEEERRVFEKADLQNKLAQKPVT
DLEVKIKVVEAMKYGCDFNVSAVIVNKTSSDRVCRLMFCARTISYNGEIGNECGMKDLLNLEIPANQEKSVPLRVLYNKYCTTMTEDNLIKLMA
ILFEYKSKDIMLATRSIVIDNPDIKIKILGEPGQFKKVGAEVTLKNPLPVPLTNCSFTIEGAGLTEGQVCENIRDVDKNQEAKAKLYFTPKQSG
MRKLVVEFNSNKLQNVKGYRNVIIAPVRR

>lungfish_TGM5L
MSSALLVKSVDLQFKENNRQHRTEEISKKKLIVRRGELFIITVNFHTRGYEEGRDNITLIAETGPRATEEAKTKAVMTVGASVKPNMWNTTVKN
SHDHTLTLSVSSPANACIGKYTFQMQLSSDGNKTSYNMGEFILLFNPWCKEDEVSMTDEILTQEYVMNEQGTLFFGSTDYISSSPWDYGQFQED
VVDICLKLLDLNPKYLKSPSKDCARRGDPIYVSRVVSAMVNSNDDKGVVEGRWHGDYSDGVSPGTWSGSVPILRKWYKSGYKQVKYGQCWVFAA
VLCTVMRCLGVPTRVVTNFSSAHDTEGNLTIDEYYDLKGKKLDRSYDSIWNYHVWDECWMKRPDLEEIYSGWQVVDSTPQETSEGVYCCGPAPV
LAIKEGHTHLKFDLPFTFSEVNADVIRWISYPDGRTKKASSDTKLVGQYISTKTVGSDEREDITKKYKYDEGTTQEREAVARALRNIRNRSRYE
SARADDRLHEDSEEPNLPATKIDVSIKVDKTPVRGQNIDMLVNVTNKSPAKKHLMLFISAQCMFYDGRPGTRFWKKEADIQLASNEGKDFPYQI
LYSEYDEYLSDSRLIKIAATVTEQETTQTFLAEKDIFLSNPTISIQAPDEALKYEPTTAEVLFSNPLPEPLNSCVLTVSGSGLIYDEVKIDGRQ
MKPGVRGILRVEFTPFKTGMRRLQVVFECDKFKDVKGYKDIKILPSSEEEEDFSNFGIYRY

>lungfish_TGM10
MKVKRKRKCSDIVMMHSFFKHREYCIFDTVDLLCRSNNGAHHTDEISTKRLLLRRGQPFHISVKCRQNILTKPVLPFHFINVLMSIGKNNGTQI
KLSTMKTETTEWRFSMELAGDELLLTICSPASAIIGRYNLYLMLYDSQNQLQQQKSAGQFYLLFNPWCREDTVYMSEEEKLQEYIMNEHGVLYQ
GMWNDIYEVPWNFGQFEENIVDICFEILNNSLPALSNPVNDTLKRSEAEYISRIVTAMVNSNDDKGVLLGKWDGKYWDGIPPTRWTGSVQILQQ
WSRSGAEKVRYGQCWVFAAVACTVLRCLGIPTRCVTNYCSAHDTDGNLNVDCYLNNELQLIPGSTKDIIWNFHCWVESWMARHDLPPGNDGWQI
LDPTPQERSDGVYCCGPCPVKAVKQGNVDVKYDTAFVFAEVNADIVYWVLNKDGTRSQTGAFCKEVGKCLITKNAYGDKREDITQHYKYPEGSK
EEREVYAKAGMKRRLLCSESKDVCLTFKHATVVYGTDFDVYVEICNNSFVDKNVNLTIVAKTVTYNGIILQECHRKTTSFPLKALKVKREVCRI
KYEHYADRISEHNLIRITALLSQNGKDEMILSEKDIVLAVPQLNIKIIGEPVLFQRCMACVSFVNPLPITLLNGIFTIEGAGLTNIQEIKSKPE
EIRPGQEVTVTVPFIPTKAGLRMLLVDFDTNRLKAVKGHVSVLVSEE

>lungfish_F13A1
MSEGTTDPGQKPKHLTNIGRRASPLDISNSEEGSIPEFESFIMLPRGIPPKLDAFLEVLSVDLLRHPTEINKQQHHTDKYDYLNLIVRRGQNFT
MKIKFNREYISNKDQFWVEYLIGRSPRIDNGTYIVAPLTEELKKDKWGAKILSIRDETLTLAIQSAPDCIVGKFRMYIAVMTPFGIRRTPRDPG
TDTYIIFNPWCKDDTVYMENDAEKEECVLNDVGIIYHGEYNNIQHRNWNFGQFESQILDSCLSLMDRAEMPLSSRGNPVKISRVASAMINAKDD
DGVILGNWTGEYLYGLSPSAWTGSQDILLEYYNSHRSVRYGQCWVFAGVFNTFMRCLGIPGRVVTNYVSSHDNDGNLITDIILDEEGKVDKAQA
QDSYWNYHCWNECWMARSDLPEGFGGWQVVDATPQETSDGMYRCGPASVKAIKHGFVYLPFDSPFVYAEVNSDIVYWKRWRNGRKEIVDIVGNQ
IGKLVLTKENGKNAQMDITDQYKFPEGSEEEAIALKAALMYGTKKETSIPQASKDCTLQVVVDSKVPFGSDFNIKMDFGNNSRQTLNIKAYVSG
NIVYYTGVTKREFKSSTFEFNLDSGNYKKETVLIKSKDYMNHIVEQAFLQFITTASIEPSGQIVTSLNVVSLIVPKLSIKVLGEPVTRNEITIL
VEFTNPVKRPLENVNLRIEMPGNMRSKTTKYSYISPNETVSWAVKFVPWRPGLHLCFASLDCDALRQVYGETEINVR

>coelacanth_TGM1
MPAIARETSIYNDVGRRAPVPLAANGTRGKTEAQRAKKSLWRCCPCLGLQRGSTIEVDTDECEGTVPAVEEDPKEMKEFPLTVKNVNLVRGRDE
INKKSHHTDEFEYDELIVRRGQPFQIKLELSRPFNLETDKLFLELQTGLLPQVSKGTLVIIPVVEELEYNEWGVKIVEATDNVLSLLVNSSPQA
VIGRFELTVKLQPAGERENRTEHKPANDIYILFNPWCEADAVFVEDEEWRKEYVLNETGRIYYGTKNQIGARTWNFGQFDKGVLDACLYLLEKG
KMPHSGRGDPISMVRVVSAMVNSQDDRGVLVGNWSGDYTGGSAPTEWVGSVDILLQFHRTGEPVNYGQCWVFSGVTTTVLRCLGIPGRSVTNFA
SAHDTDVSLTTDVYLDENLEPLEHMNFDSVWNFHVWNDCWMARLDLPPGYGGWQAIDATPQETSAGTYCCGPASLQAIKSGLVYLKCDAPFIFA
EVNSDRIYWQRQHNGTFQKVLVQKNAVGHQISTKAVGLDEREDITHLYKYPEDSEEERIAVETACQYGSKPTVYLNSAVMEDVALDIQTQEDIQ
MGSDVTVRVLVENCSSEHRSISLFLKAAVVYYTGVYKNSFKQDREEVLLSPAEGKELLVVLSYSQYQEYLVDQAAMMLTVSGRVVETGQVLAKQ

HNFRLRTPDLQIMPYGEAIVGGQMKAEIVFLNPLPKILKNVTFRIEGPGLQKPKKVQVGDVGRHATVTLKETFVPTKPGPRKLIASLHCRELTQ
VHGVAEVIVLSQ

>coelacanth_TGM1L1
MTPTSVNKLGEKDEEMPQGRNPSLICNTISRGAKALSDDNTIKEESLLEVRNINLLKKIHENNKIFHHTDEFESDYLIVRRGQPFEIKVELSRP
FDAQTDMLLLELQIGPKPRLKKGTLVIVQVVKEHNPKEWGMKTFWETEQTLTLSVNSSPEAVIGRYQLTVRTLTRKEEFRTKHNPDNDIYILFN
PWCEADIVYMENEEWRKEYVLNEVGTLYYGTSEAIKSKMWNFGQFEKGILEACFNLLEKGRLPQYVWGNPITLVRFISAMVNSQDDDGVVIGNW
TGDYSEGTAPTAWAGSVDILHQYHQTGKPVKFGQCWVFSAITTTVLRCLGIPTRSLTNFDSAHDTDVSLTMDIYIDEALEPIEEMNLDSTWNFH
VWNECWMARPDLPPGYGGWQAVDATPQEMSVGKFCCGPASVRAIKNGLVYLNWDSPFIFAEVNCDIVYWKKKKDGSGIFRPIFKEESAIGHFIS
TKAVGSNKREDITHLYKYPEGSEEERISVETACQHGTKPKTYLEGEKVKDVVINVQTEKNLQMGSDFTVWLRVQNCSTEQRGISLFTQVAAIYY
TGVYKSCFKKKREEVQLSASEVRELELVIKYSEYDPHLESQDNMLFTVLGRVAETRQVIAKQHKFCLCTPDLQIRVLGEAIVGKEMKAEIVFIN
PLPKILKNVQLHIEGPGLQTPKIVTIGDVGSHAKVTHTESLVPVRPGMRTLIANLDCPQLSQVHGVAEILVKTEAH

>coelacanth_TGM1L2
MLSTKSKAKDDPGRRNLGCSSNTQKEKPKLVQEAASLQENKEDEVNGAAEQKENLLKVKNIDLLKKPEEINKKSHHTDEYEYDELIIRRGQPFQ
VKLEFSRPFNPETDKLFLELKLGSQPQVGKGTHVIVKLVEKHIPSEWGAKIIEASGNTLTLSINSSPQTMIGRFELTVKTVTEGGAFKMKYNRD
NDIYFLFNPWCEADTVYMEDEEWRKEYVLNDTGRLYYGTENSIGSRTWCFAQFEKGILEACFYLLDKGGMPDSGRGDPISVVRVISAMVNSQDD
DGVLVGSWSGVYWDGRRPTSWTGSSEILLHYHDSYNPVRYGQCWVFSGVATTVLRCLGIPGRSVTNFSSAHDSDTSLEIDYYIDEDHCALDHLN
NDSIWNFHVWNECWMARPDLPQGYGGWQVVDATPQETSAGTYCCGPASVRAIKNGLVYLKYDAPFIFAEVNSDKVYWIRDSNGDFKRYYSARSV
VGHNISTKAVGSEERLDITNLYKYPEGSEEERLSVKTACKFGNKSKMSIEKEVEEDVVVEVEITGNDLIGSNIIARATVTNRGNNVCNICLFMR
AAMTFYTGVYKNEIKNMKEEVTLEPAEVKEVKMTIKYDEYDDYLEDQACIMFTLMGLVSETKQIITKQRDYRLRTPDLMLKVHGEVVKGKESKV
VISFVNPLPKVLKNVVIHLEGPGLQKRKTISVGEVAKQGLMTITEIFIPSKAGCRKLIANLDCHQLTQVHGVVEVEVINKPAEV

>coelacanth_TGM1L3
MSGSRFEEKKRALDSNVQTLEEVQPIILKRVRRDSGPSAVGENILTVKSINLLKGKEEINKKSHHTDEFEYDELIVRRGQSFHIKLELSRPFNP
EADKLLLEMHLGPKPQFGKGTLAIVRLVQELDPKSWSMKIVKVSDRVLTLKVNSSPEAPIGRYQLIVKSLTLNEEFRTKHNPDDDIYILFNPWC
KADTVYMEDEERRKEYVLNDTGKLYRGMQRWIGASDWNFGQFDQGILEACFFLLDKGKMPHAGRGDPISMVRVVSAMINSADDDGVLVGNWSGD
YSDGITPMAWGGSVEILLGYYSTGGEPVSFGQCWVFSGLTTTVLRCFGIPTRSVTNFDSAHDTDVSLSMDTYYDEDMEEIRDLNSDSVWQPVAT
YLYVHTNHFLFSKGTYCCGPASLRAIKNGLVFLKYDAPFIFAEVNCDRIYWQRKADGTFEKFDVVKNAVGHKISTKAVGSDEREDITHLYKYQE
GSEEERISVQTACQYGTKPNLYRKDDAANDVTADIKAEDKIQIGSDVMLKISLGNRSMDKRTVSLFVQSSAILYTGVNKGTFKREQEEIVLGPC
EVKEMVKMLKFEEYDDHLVEHGSFMFTILGRVKETKQVFVKQDHACLSSSSLKIMILGQAEVDREVQAKFEFTNSQTRRIRNVQLRIEGPGLQK
PKTIYIGDIRGNQTMSHTEMIIPSKPGARKLIATLDSHQLITEVQGFANVYVKKPDQCV

>coelacanth_TGM1L4
MTPTSVNKLGEKDEEMPQGRNPSLICNTISRGAKALSDDNTIKEESLLEVRNINLLKKIHENNKIFHHTDEFESDYLIVRRGQPFEIKVELSRP
FDAQTDMLLLELQIGPKPRLKKGTLVIVQVVKEHNPKEWGMKTFWETEQTLTLSVNSSPEAVIGRYQLTVRTLTRKEEFRTKHNPDNDIYILFN
PWCEADIVYMENEEWRKEYVLNEVGTLYYGTSEAIKSKMWNFGQFEKGILEACFNLLEKGRLPQYVWGNPITLVRFISAMVNSQDDDGVVIGNW
TGDYSEGTAPTAWAGSVDILHQYHQTGKPVKFGQCWVFSAITTTVLRCLGIPTRSLTNFDSAHDTDVSLTMDIYIDEALEPIEEMNLDSTWNFH
VWNECWMARPDLPPGYGGWQAVDATPQEMSVGKFCCGPASVRAIKNGLVYLNWDSPFIFAEVNCDIVYWKKKKDGSGIFRPIFKEESAIGHFIS
TKAVGSNKREDITHLYKYPEGSEEERISVETACQHGTKPKTYLEGEKVKDVVINVQTEKNLQMGSDFTVWLRVQNCSTEQRGISLFTQVAAIYY
TGVYKSCFKKKREEVQLSASEVRELELVIKYSEYDPHLESQDNMLFTVLGRVAETRQVIAKQHKFCLCTPDLQIRVLGEAIVGKEMKAEIVFIN
PLPKILKNVQLHIEGPGLQTPKIVTIGDVGSHAKVTHTESLVPVRPGMRTLIANLDCPQLSQVHGVAEILVKTEAH

>coelacanth_TGM2
PCPVETSGTRAKFTLSNSVTEDWSAAIICNNGNVLSLSVQSPANARIGRYFLTMESQDSSIDLGEFILLFNPWCPGDSVFMTDERKLKEYVLTQ
DGIIYQGTVEYITSTPWNFGQFEGGILDVCLLLLDTNPKFFKNSDKDCSRRNDPVYITRVVSAMVNCNDDKGVLYGKWDGEYDGGISPLHWTGS
VQILRNWMTAGCLPVRYGQCWVFAAVACTVLRCLGIPTRVITNYYSAHDTNSNLIIERYIDEKGMSVNKSKDSIWNFHCWVESWMTRSDLKPDY
DGWQVVDPTPQEKSEGVYCCGPAPVKAIKEGEVALKYDVPFVFAEVNADVVFWIKHPNGKQEKAVYPSQVGKSISTKSIGSDTREDVTHHYKFR
DGSPEEREVFLKADHQHKLTQKPQTDLDLTIKVSDGMNNGCDFDVFAVITNRTSVEHFCRLMFCARTVSYNGVIGNECGMKDLLNVTIPANQEK
RIPLRVLYSKYGDSLTQDNLIKLMALLFEYQTKDLLLAVRDIYIDNPKIKIKILGEPAQNRKLAAEITLHNPLSVPLTDCLFTVEGAGLTDGQQ
IQQAVGPVDPGQEAKAKVYFTPRQSGLRKLVVDFDSNKLRNVKGYRNVIISPAPK

>coelacanth_TGM4
MSWQAEDALRIEDVDFLQKKNGKHHHTDEFESPHLIVRRGQEFKMKLKLDREFRKKDKVYFHLALGQGKVQSSESLIVIELDSADSSQLWRATV
CQAKGRKCVVQLSTPPDATVGEYHLWVKTDEQYTFSPDDNRIYILFNPWCQDDAVFMREGPERREYVLNDTGYIYVGSVQHISARSWNFGQFEE
DVLDCCMYLLNKALPEAAERRDPVKVARKMSALVNSNDDWGILYGNWSGSYDDGTPPLTWTGSTAILQQYYKTRKPVKYGQCWVFSGVLTTVMR
SLGIPARSVTNFVSAHDTEGNLKVDIYVDKNGEYLDDMITDSIWNFHVWNDVWMKRPDLPEGYDGWQALDSTPQEKSKGVFQCGPSPLPAIKSG
EVHLEYDTRFVYAEVNADKIVWLVKHPSTAREKRIKLRKNAKAVGKHISTKAVGRNEREDITTQYKFHEGSRMERKNMKAACTEQPAVALPLVS
TPVVQLGIRGDSSIPVGEPIALTITLTNTTSDSKAVSLTAACQLQTYAGKTVASIGNLKRDTEVGGDQCTEVPMKVEVDAYMKHLVSSKDDLLI
HVSVVSKTGDGSEIDTEDATLIFQYPSIVAEMPATAKIGETVVCTFTFKNQLSIPLDNCKLHVEGLGLFKLETLNQGGIEPGRIFRSKIIFNPT
KTGERKIVAKVSSVQINGIAVEKTITVTE

>coelacanth_TGM10
MTAPDNLLGVVDLHCNYNNTAHHTDEIDSERLVVRRGQPFTITVECRSGLFHPGRDQLAIVLDIGQKPSTDNGTRIKISSSDAKAEKWRFAMQA
DQYELQLTLYSPADAPVGQYTILLLVHSDSKLIQKKAAGQFYLLFNPWCKDDTVYLPDKEMLNEYIMNENGQLYQGSWNDIYPVSWNFGQFEKD
VVDICFEILDNSLPALNNPVADILKRKDPIYVSRIVSAMVNANDDKGVLLGRWDGNYFDGIPPTRWNGSVQILRQWSKSGAKKVRYGQCWVFAG
LACTVLRCLGIPTRCITNYYSAHDTDGNLKVDKCFNEDLDVLPGKRKDMIWNFHCWVESWMTRQDLPSGYDGWQVLDPTPQERSDGIYCCGPCP
IKAIKEGNINVKYDASFVFAEVNADVTCYIVKKDGTKSETAVYCHQVGKCISTKSIFGDNREDITEQYKYPEGSSKEREVYAKAGLKNRLVSSN
TKDVLIAIKHTRAVHGSDFDIFIEVCNNSSVDKDVGVTIVAKTVTYNGIILKDCHKKITSFSLMASNAKKEVFRLKYEHYGEHLSEHNLIRITA
LLLQKGKNELILTERDIALAMPQLTVKILGEPMVSRKLKAHIKFVNPLPITLTDGIFTVEGAGLTDLQEIKCPGKIKPGEEVTVDVSFIPTKTG
LRKLLVDFDTSRLRDVKGYASVIIKG

>coelacanth_F13A1
MSESSDAEGRKPKAVSHLGRSSNPPDISNAAEDTVPEFETFGGFSPRGLPPLQDYLEVWRVNVLKNPEEINKKEHRTDKFICRNLVVRRGQPFQ
MKITFNRAYNPETDVVWLDILIGRYPDVRKSTWIQVRLNQELEKGKWGTKVVESIDNNVTLSVTSSSDCIVGKFRMYIAVYTSFGIRRTARDRD
TDIYVLFNPWSKEVINSRDDDGVIVGNWTDNYSYGLAPTAWTGSGDILLQYLSSGASVCYGQCWVFAAVLNTFLRCLGIPGRVVTNFYSAHDND
GNLVMDVVLDEDGKTNKKLTKDTVWNFHCWNECWMARSDLPAGFGGWQVIDATPQETSEGTYRCGPASVHAVKHGHVFFPYDAPFVFAEVNSDI
VYWKRMKDGTRTVVKIDSCHIGKMILTKEVGSDERKDITELYKFPEGSNEERLALESAMKYGAKKEKTTSPFPVDVYMEVYTEKNVTISNDVKL

TLEFRNNSQEGRAINVFIVGYIVFYTGVKKDAFMDQTLSVVVEPGYFKNVDVHVKAKDYMSHLVEQASLHFIVTARVNETTQILTAQCVVPLRS
FKLNLKIDGKPELGNQMSVTVEFQNPLKKPLENVSVRLEGVGLMRTKIKEYSTIPMNESIKWTEMFVPAKPGLRKMIASLDCVALRQVYGEIQI
MIN

>zebrafish_TGM1
MEKTEQKWSCGAWFRQCCCSCRKSHKNTNSTPHAANTTAEKTETPATGSLLEVRSVNLLKSSKEQNRLEHHTERYRSENLIIRRGQTFQMQIEL
SRAFDPKTDKLHLDLKLGDLPDVSKGTHVVVPLVEELQDNCWEAKIVEQKGRLIKLSVNSLPTAPIGKYKLGVATSCPVGESMSPYNPDNDIYM
LFNPWCEEDSVYMDSEKERKEYVLNDMGIIYYGTDSQIGYRTWNFGQFDKSILPACLFLLERSGAPASGWGDPVNVVRLLSAMINAPDDNGVLV
GNWSGTYDDGTAPTFWSGSSDILRQYYNNGGTPVRYGQCWVFSGVTTSVMRCLGIPTRSITNFESAHDTDASLTTDVYLDENHELIEELCSDSV
WNFHVWNDCWMARPDLPAGLGGWQVVDATPQETSQGAFRCGPASVAAVRNGLVYLKHDTPFVFAEVNSDKVYWQRTADGSFTPVQIKKKAVGHC
ISTKAVGSDEREDVTHLYKYPEDSEEERIAVETAVQHGSKAGLYEISSVNDVSIEISMDGEDPQLGSDANLAITVKNSSSEMRTFQLSAQVAVT
YYTGVYKGTVRKDQISIELKPNEAETVEWTLLYDHYKDHLVDQAMLMLILTGRVNETKQVLVNQFHFRLRTPDLAIKTEGDAVVGKELKASITF
RNPLKQTLKNVKFRIEGLGLQHVREISHGNIESLATVTLTETFIPLEAGQHKLLAALDSRQLPQVHGVANITVKAN

>zebrafish_TGM1L1
MPGERQSIRDNSAVGRFHGVSARNGEAVAEKPSESKTKKPESGCRRWLRKACPCCLRRQSNSYDLTSEADGVVAGNEEIEQPASPVSVSVETNV
DDLSLVVRSVDLLSRKTDRNKKEHHTDQYSGDQLIIRRGQTFQIELELSRPFNPNTDKLHLELKTGALPLVSKGTHIIIPLVEELQDERWEAKI
VEQNLNRAKLSINSSVNAVIGKYTLTVVTQCLKTNESTTHDPEKDIYMLFNPWCEDDAVFMEGERELNEYVLNDTGRIYYGTEKQIGARTWNFG
QFDEGILEACLFVLDNSEVPPSGRGDPVNVVRVISAMINSPDDRGVLEGNWSGNYTGGTSPTAWSGSVEILKQYHREGGTPVKYGQCWVFSGVT
TTVLRCLGIPARSVTNFQSAHDTDVSLTTDVYFDEDMEPIHHLNSDSVWNFHVWNDCWLARPDLPPGFGGWQAVDSTPQETSHGTFRCGPASLA
AVRSGQVYLKYDVPFVFAEVNSDKIYWQRNLDGTFSQIHSEKKAVGHCISTKAVGSDERVDITDVYKYPEGSEEERIAVETACRYGSKPDVYSS
AMAEDVQVEVRMEGEGPRMGGDAQLKIVVKNMSSQPRRTTLHSQAAVMYYTGVLKDTVKKDKLSVELMPQEEKVIEWTLPYTQYQNQLVDQAAL
MLTLSGRVSETQQVLANQTSFRLRTPDLQIEPIGEAYVGKEASAKISFTNPLPCTLRNVVVRVEGLGLRDLHPIKVGDVGKHGKVMVTEHFIPS
IAGERKLVASLDCKQLTQVHGVADIIVHESQ

>zebrafish_TGM1L2
MPFSCSPYNNNLCGRFTPIKLMADDCRRGACYSPYANSWYNPCASSCGKINRCATPCADPCGYPYGYSYGYPYGSNCGYNYGYPYGYNCGYNYG
YPYANRCVNPCTTTTPYVKSCVNPCLDTCVNPCVNPCATPCSTYSYRDYESRQVVTRECIDDCTTKRVCATDEALLRVSKVDLLSCRTGPNRME
HRTHFFHEEKLIVRRGQCFNMWIDLCRPFNPTCDKLHLELRLGHIPSIRDGTYVIVPIVDEFKKDCWGARIVERCQNRLKLCVNSLPTSCVGRY
QLSVVTHCSAGRFCLPYVPENDIYMLFNPWCKEDCVYLHEETERAEYVLNDIGKIYYGTKHQIGCKSWNFGQFEEGILPACFYVLEKSCAPCSG
WGNPINISRVVSDMVIAKKDCGVLMGNWSNCYTDGIAPTSWCGSSAILRQYYKCGGAPVRYGQSLAFAGVTNTMLRCLGIPTRPVSNFCSAHDT
DMCLTSDVYLDEKFQLIDHMNANPIWNYHVWNEAWMTRPDLPTGFGGWQAIDSTPQLTHQGFFRCGPTSVAAIRSGQTFLKHDVPFLFAEVNND
KVYWQRKCDGTFGVVHVEKDVVGHCISTKAVGSDQRLDITNLYKHPCGSEDRCTALETALRHGCRRINYPLPCAEDVVCEVNLKGDGPCVGRDA
VVCINLRNKCNQPRSVTLYSHAAAMYYTGVRRTYLKRDQTCIELKPSECKPLEWTLSYDEYKEHLVDHAPLMLNLFGHVAQTKQILATQYNFRL
RTPDLVLAPACDAVVGQEVAVKVTFQNPLSCVLRNAAFRFVGLGLQHPRIINYGDIAGHATVSLTEKFIPMCHGPQKLLASFDCPQLTQVHGFT
NMVVKQH

>zebrafish_TGM1L3
MPIVNGGLNNFCNIGRFPAVKLQDCFWKGSGYVPVYSNPCVKPCVDPCWNPCVKPVVDPCGNPCVKPVVDHCGKPVISPCGKPIVDPCGKPVVD
PCGKPIFDPCGKPVVDLCGKPVVGPCVVPLPKPCVNPCDVRPHCDYDCKQVFLKDCADDCNAARCCEDLVLQVRCVDLMKCWKSQNRQEHHTNG
FRGDHLIVRRGQCFQMWVELSRPFNPKCDQLHLELKLGNVPSIPNGTLVIVPLVEEFKKNRWEAKIVEKCQNRIKLSVYSLPTACIGRYSLTIV
TCGPKGRATSSCNPSNDIYMLFNPWCKDDAVYLDEDAQRTEYVLNDTGKIFYGTKHQIASRTWHFGQFDEGVLAACLFVLEKCGGACSGWGDPV
NVARVVSAMVNANDDSGVLMGNWSNCYADGTAPTAWCGSSAILKQYHKCGGVPVKYGQSLAFAGVTNTLLRCFGIPARPVTNFCSAHDTDVSMT
VDIYLDENYDLIDSLNRDSIWTFHVWNEAWMARSDLPAGFGGWQAIDATPQETSQGVFRCGPTSVAAIRSGQVFLKYDAPFIFAEVNSDVVFWQ
RKACGTFAVVHVDKNAVGHCISTKAVGSDKRVDITNHYKHPEGSEEERRAVETALRHGSKKCAYPLPCAEDVICDITMKGDGVCVGKDAVLCIA
LKNKCSSTRSVTLHSQLSAAYYTGIHKSLVKKDQTCFELKATETKVLEWSLKYEDYKNHLVDHSTMMVTVAGRVTQTQQIVAKRFNFRLPTPGL
AISPGCDCVVGKEVPVKITFQNPLPCVLKNAIFRIEGLGLKHCRSINYGDIAGLATVNLTEKFIPKCHGPHKLLASLDCPQLTQVHGFTDVVVK
EK

>zebrafish_TGM1L4
MSSSSTPARPAPSAATASPSPSAPAPAAVSAASISPSASASDSVLAVRSVDLLRLRDGLNRRQHHTDGFSSERLIVRRGQSFQITVELSRAFKP
RADSLQLQLKLDSAVSNSSGLLISVPLVEDLEDRRWEMKIVEQKENRVRLVVNTLPSASIGCYKLTVVSFSPRGKLLFPCTPDDVYLLFNPWCE
DDPVYLDNEAERKEYVLNTMGRIYYGTEQQIGTRTWNFAQFEQNILEACLFLLERGRVAVTEWRDPVIISRMVSALVNSNDDRGVLMGNWSESF
EGGTAPTAWSGSGDILRQYYSSKGSPVRFAQCWVYAGVTCTVLRCLGIPTRCVTNFSSAHDTDLSLTTDIYLDEKLEMLKDKTSDSIWNFHVWN
ESWMRRGDLPAGYGGWQVVDATPQEQSQGSYRCGPTPVSAVRSGQVNLRFDTPFVFAEVNSDKIYWQRNADGSFRQVSVEKNSIGQKTSTKAVG
SDTRVDITHLYKYPEGSEEERIAVESASRFGSRPTLYPSPSGTDVSLEVQMSGAGPRIGEDVQLSIVLKNSSSAQRSASLLYEALVMYYTGVLK
QSLKKDRITLELQPRETKTIPWTLQYKEYKEQLVDQGALMLTLTGRVSQTKQVLATQFNFRLRTPDLVLTPLQDAVVGKEMSVRISFQNPLSQV
LKNVLFRIEGLGMQSVRKISYGDVARLGTVSLIEKFTPTVSGSQKLLASMDCRQLTQVHGVADITVKAK

>zebrafish_TGM1L5
MNLVFVPAVLDAVLQVRSVDLIKTRKGQNRQEHHTDAFFSNHLIIRRGQCFQMTIELSRPLIPNKDQLYLELRLGNVVPAHRDSFVSVPIVSEF
KKNAWEAKIIEQAKTTIKLSVYSLPTACIGQYKLTVVTNCPAGKATSPYTPDNDIYMLFNPWCKDDPVYLKDEAERNEYVLNDMGKMYYGTEQQ
IGTRTWNFGQFDEGVLEACFFVLEKSGSPCSGWGDPINVVRVISALVNSNDDQGVLIGNWQNSYEGGLSPTAWSGSSAILKQYHKSGGTPVKFG
QCWVFAGVTNTMLRCFGVPTRPVSNFSSAHDTDVSLTTDVYLDEKLEEIKDLNRDSIWNFHVWNESWMTRPDLPAGFGGWQVVDATPQETSQGV
FRCGPTSVAAVRSGQVYLKYDTPFVFAEVNSDKVFWQRQSNGSFTVIKVDENAVGHCISTKAVGSDQRVDITHLYKHPEGSSEERSAVEAACSF
GSKRSIYLPRSSSNTDVTVDVVMEDSGACLGQDAVLFIVLKNRSSSARTVDLQSRVEAVDYTEHHKAFLRKDQTRAQLKPHEIQSLEWILQYEE
YKEELEGQTSLLLSLSGRITETKQTLVKHFTFRLRTPDLVLTPVGDAVVGQELKVKLKFQNPLVSVLRNVIFRMEGLGLQHVKTIHYGDITGGA
TVRLTEIFVPKRSGPQKLLATLDCPQLTQVHGVANILVKHR

>zebrafish_TGM1L6
MMPFVNNPCKSCTTGRFPVFKHETDNSCKGACYPTVYTHPYASHYSSHYTHPCVNPCANTHINPWANVHTNPCANVHTSPCTKPCINPCATSWA
TPHTNPCSFVTPCHDYKTKEVIIKDYKDESISKQVSGGNVDLLRVIKIDLLKCKTGPNRQEHHTHFFHDEQLIVRRGQSFNMWVDLCRPFNPAS
DKLHLELRLGHIPSIRDGTYVIVPIVEEFKKDCWGAKIVEHGQNRIKLCVNSLSTACVGRYQLSVVTQCTAGKFTLPYVPEHDIYMLFNPWCKE
DSVYLSDQAERTEYVLNDMGKIYYGTKQQIGCKTWNFGQFEDGILPACFYVLEKSGTPCSGWGNPINISRVVSEMINANKDRGVLIANWSNYYV
DGTAPTLWSSSSAILKQYHKCGGVPVKYGQSLAFAGVTNTMLRCLGIPARPITNFCSAHDTDVSLTTDVYLDEKFQLIDQMNRDSIWNYHVWNE
AWMTRPDLPTGFGGWQAIDSTPQQTHQGFFRCGPTSVAAIRSGQTFLKHDVPFLFAEVNSDKVYWQRKNNGTFGVVHVEKDVVGHCISTKAVGS
DQRVDITNLYKHPCGSEDRLTALETALRHGCKRLTHPLPIPEDVICEVNLKGDGPLVGKDAVLSINLKNKCNQPRSITLYSHAAAMYYTGVRRT

YLKRDQTSIELKASESKLLEWTLSYDEYKEHLVDHAPLMLNLFGHVAQTKQILATQYNFRLRTPDLVLAPVCDAVVGQEVAVKVTFQNPLSCVL
KNSVFRFVGLGLKQARVINYGDIAGHATVSLTEKFIPMCHGPQKLLASFDCPQLTQVHGFTNMVVKQH

>zebrafish_TGM2a
MERVVEIGSIDLACEVNNTNHHTHLNGVDRLIVRRGQTFTIHLHLKEGTHFQNGDNIKFIAQTGPIPSVEAQTKARFSLSKVISRLSWSATAET
HNSTVSLSICAHTNAPVGRYTLILDQGDGVILGEFVLLFNPWGKLDSVYLANEAEREEYVLSQDGLIYRGTPKRITVLPWTFGQFEHGILDICL
QILDESPNYISDAALDCSERKNAVYVTRVLSAMINSLGDKGVVVGNWSDDYEDGVKPTVWKDSCSILRQWSNEGCRAVRYGQCWVFAAVACTVS
RALGIPCRVITNFGSARDSNGDLIMERFYNEFDENIADDSIWNYHVWVENWMTRPDLALGYEGWQASDPTPQHRSDGVFCCGPASVRAIKEGEL
TFKFDVPFVYAEVNADVVEYIKLRDGRVFKMGGSTTEIGKSISTKAVGRDEREDITHNYKYPEGSEEERKVFKKANHHNKLAQAGEEPGLHIKI
RVTPDMQIGSDFDVYAEIKNNTMVTKSCRVMFYAQAVSYNGTLGETCGLGDFTEMSLASSDGGKVTLRLEYAEYSKAITQDRMIKLVGLLIDAE
TREFYRAKKTIVLDAPEIIVNILGVPKVGRNLVADLALQNPLPEPLENCVFTIHGANLTDGKPITHEVGTIGPKEFATAKVEFAPKLPGQRKLI
IDFASDKLHNIETYENLVIYE

>zebrafish_TGM2b
MALDIGSWDLACKFNNTDHHTELNGTDRLIVRRGQAFTINLQLNSGSYQPGYSQINITAETGPDPQQQYGTRAVFSLSSEVDSSCWSAAVSSPP
GESVCLSICAAPDAPIGHYTLTLDERIQIQFILLFNPWCPLDVVYMDNEEKLAEYVLAQDGIIFRGDAGYPVPLAWNFGQFEEGILDACFRILD
MNPKHRRNPAKDCSGRRNVIYVTRVLSAMINSNDQDSGVLEGCWRDTFDGGVSPMSWGGSVQILRTWDRSSCLPVRYGQCWVFAAVACTVARAV
GIPCRVVTNYYSAHDTNSNLLIERYVNEKGEVDHSSTRDMIWNYHCWVESWMGRSDLPPGFDGWQASDPTPQEKSEGVFCCGPVPVRAIKEGEL
TFKYDAPFVFAEVNADLVYFLKSKDGSTRKINYDQKVGQKISTKSVGRDEREDITHLYKYPEGSADERRVFEKANHQNKLLQEKQNTGLNITIK
LSSGVRKGCDFDVFAIVTNGTAEEKKCRLVFASRAVSYNGVIGRECGFKDLLNVELPPGGERKVPLRLNYSKYCNNLTEDNLIRLGALLIDYST
RDAIMAMRDIVLDDPEIKIRILGEPKENRKLAAELTIQNPLPEALQSCCFTIEGANLTGGDSITHTLDSSIEPGQEAKAKIYFTPTQSGLRKLL
VDFNSDKLGHVRGYRNVIIGK

>zebrafish_TGM5L
MDEFKFVSVNLQPVQNQLQHRTDGLSSGSLVVRRGQPFTVTINYAGRPYDPLREKLQFIFILGPLSVEVPVSSYGQPSSSKWSAVLERGLPNPS
GSRALTVSLSSPASSSIGVYTLQLTVQTRSSTKTHLLGQFTLLANPWCQADSVFLQSEDLRNEYVRSDFGLLFKGTPGNVVSRPWSFGQYEKGL
LDICMKLLQLSPQSQTDMKRDLLNRSSPIYIGRVISAMVNCQDDKGVLMGNWSGDYSDGVNPSVWSGSADILRMWSETQFSPVKYAQCWVFAAV
MCTVMRALGIPTRVVTNFNSAHDTNGNMVIEEYYTEMGEKLSIGRDSIWNFHVWVESWMKRPDLGQTYDGWQVLDPTPQEESAGMYRCGPAAVK
AIHEQKVDAAYDVPFVYAEVNADVRTVIIRDRKILGVSVDKERVGALICTKRPGAMTMLDITSEYKTEMVLSSAYAFSADVRGGEAAPARVAPQ
GLAVSLQLLKTPIIGESIAFSIIITNQASIPKLLRAHANAQNKEYHRNPFGSFWEKHHELKIGPSETVTINQEISFNEYKLKQVLEDYLVNLAV
VVEDVKSQERVLASEEFNIHSPALSIQIQNENVIVNSPQVAMVTFTNPFDVAVTGELTVAGSGLLEEKIQIRVSMQPREAVRRPVRFIPQMTGA
KMLYACLVLINLPTVLHGFKTLSVQAA

>zebrafish_TGM8
MCSSSSSNTPDQGVRVDLECVKNNTDHHTHEITQERLIIRRGQAFSLKISAENIQQNHISITAETGPGVSEVKHTLFSFSTQSSTNPAQVCSRS
ESSVSLALVFPSDAPVGRYSVCVKHGSSSCMNTLTLLFNPWCKDDHVYLPSEAERQEYIMSEQGVLYKGVDEYITSMNWDYGQFEEDIVDICLK
LLDLNPKCLKDPQEDYSARCNPVYVSRVVSAMINCNDDQGVLAGQWGDSYTGGVPPSRWSSSVEILRRWVKYNCSPVKYGQCWVFAAVMCTVLR
CLGIPCRVVTNYQSAHDTDRNLVVDEYFSDYGVRPKNSQDSVWNFHVWVEAWMRRPDLTEDTLYDGWQVLDPTPQEKSSGTYCCGPAPVMAILE
GHTEVKYDVPFVFAEVNADRVCWLLMTDGSRKKIMSDSRSVGQNLSTKAVGSSSRLDITDLYKHGEGSAQERAVYTEAVHRLTDLQIPESPPVS
VQMRLSLDVPPLNGADVPLKLFLKGSRAQCVCVNISAQVMRYTGAPAAAVWSHSTDVQLQDTEEKALSFTLPYSAYGHRMLENNCIKVSAIARE
KNNPKDTYLTEKNIILHTPNLSITVSGSPLLDSEMTATVQFENPLSSTLQNCSISLSGSGLLKSTEKSSTVQLGPGQRIRLQVSFTPYRLGLKK
LMATFNSATFKDVQASADVDVRSA

>zebrafish_TGM10
MASYNAILSDVDLQCYENNHAHRTEEMDVERLLVRRGQPFSVVLQCTEQIPQLPDHQINLILHLGKNNEVVLKVSDSEQDPGKWWFSQRNAQGE
VMLTLHSPADAPVGLYSMTVVLLSADGEIQEQTSPQTFYLLFNPWCKDDCVYLPSEEMLQEYILNENGILYQGAWDDITTVPWNFGQFEKDVVD
ICFDVLDNSPAALKNSEMDIFNRASPVYVSRTITAMVNANDDRGVVSGRWDGEYSDGVAPTRWTGSVPILRRWSEDGGQKVRYGQCWVFTGVAC
TVLRCLGIPTRCITNYSSAHDTDANIAVDYLVNDQLESVSEGRKDTIWNYHCWVESWMKREDLPEGYDGWQVLDPTPQERSDGIFCCGPCPVRA
VKEGEVGLKYDTPFVFSEVNADLIVWIVHPDGERSEVSQNSKIIGRKISTKSVYGDFREDITANYKYPEGSMKEREVYKKAGRQVGQKKDGPGQ
LELFIKHAPAIHGTDFDVIIEVYNAGREDTDAKLTVTSNAITYNSIHRGECQRKTTSLTVPAYKAHKEVLRLQYDHYGACVSEHHMIRVTALLQ
PNDQDNLILQETNIPLKMPALHVKIIGNAIVSRKLTAHISFTNPLPINLQGGVFTVEGAGLTEAREIKTHGKIESGQTVTVKFSFKPTRAGLRK
LLVDFDSDRLRDVKGEASVIVRTRMRHVNAVPEI

>zebrafish_F13A
MNMMSLKNQPAWRWGARAIIDKSNSLESNPPPHEKFPTVSPRGPTDEAIRAASLSVLSIDMRVTENKNVHHTDTYKGSNLIVRRNTEFTIILKL
DRAFNEQQHQVELEFLIGSSPDENKGTYIIVSIGKEKQDSSWKSRVVAMHGNSVMVGITPDAKCIIGRFRIFAVVVSVLGKERTQRNPDTDFYV
LFNPWNSEDEVYMDKEEDRQEYVMNEVGTIYNGVHNNITTRSWNFGQFEEGVLDACLTVMDAGNVPLLFRGNATEVVRQASALMNSQGDNGVLV
GSWSGDYSSGTAPTAWTGSPEILLKYASEGCVPVCFAQCWVFAGTLNTFVRCLGIPGRVVTNYCSAHDNTGNLKTDIVLDEDGRMDKSRTRDSV
WNYHCWNEVFMKRLDLPDQYSGWQVVDCTPQETSDGLFRCGPTSVNAIKEGELSYPFDARFVFAELNSDVIYHKSNKYGKTKIIHVDTSYVGKQ
LVTKKHDSNDYMDITSSYKYSEASLKERQVMQMAERRGVPSRKYLTLPEAGVEIQIQTNTIKIGDDFRLTMNIKNKSSKACTVIATVTGCVVFY
TGITGSDFKLENKKASVQASKTEPLTIDIKAVDYTPHLVEQANLLFVVYGIVEETETPLTTMRVINLQLPELTIKMSGSPRVGSDLLVSVEFTN
PYNFPLLKVELRLDGPGLIQTKVKHYSQILPGASVNYTVSIVPRAHGKKVLMACLDCSALRQVTNQLEFEVLKKELVTQGTIRFRS

>zebrafish_F13A1a.1
MALPSHLPWPWGSQRARIAFAISNALEVSLPECESLPGVMPRGPLPQTLSVQSLDMHITENKQAHNTSMYKNSSLIVRRNKEFLIDILFDRPFD
ETQDTVQLEFMIGSVPDENKGTYITVSFGSVKTESSWRGRLLEKQGNSIRVGITPDVQSIIGRFSTFAVVVNETGKRRTEKNSATDFYVLFNPW
DPSDQVYMPNEAERQEYVMNDVGTIYNGEINDISFRSWNYGQFEEGVLDACLFILDSGKMPLMYRGNATEVARQASALMNSIDDNGVLAGNWTG
DYSSGTAPTAWTGSAEILLKYASKGGAPVCFAQCWVFAGTLNTFFRALGIPARVITNYCSAHDNGGNIKANIMLNPDGSVNRKKTRDSIWNYHC
WNEVFMKRFDLPDQYSGWQVADCTPQETSDGLYRCGPTSVKCIKEGDLSYSFDSRFVFAEVNSDVVFHQFDKYGNSKIVHVDTTYVGQLIVTKR
PNTNGYIDITLNYKYPKGSAEDKRVMQLAERRGIPKRKYSPLPDAGVQIDIKAETIKIGENFTLTMNIKNQTSQTSTVSLTVTGCAMYYTGLTS
STFKLENYSSTVDAWQTTPVTMKVQAAEYMSFLVEQSNLLFVVHGQVNETGKSVSAMRVINLRPPELMMKVTGVPQVGRDLMVSVSFQNPYNFT
LKNVQLRLDGAGLTPTKVKSYDQVAPGGSVQYTDTITPYSPGRKVLIGCLDCIPLSQITNQLEINVVN

>zebrafish_F13A1b
MADQVAPENPTPAAAPTVRPKQRVSHRGRSAGGRASSNQQEGKVEEFEPFMLMPRGPPPLTEYLDIFDVDVLKQPNEVNKQAHHTHLYSSNFLI
VRRAQEFQIKITFNRPYKPAEDKFAVEFVIGPVPQYSKGTYIPVFPTAKRQSVWSGRVIESSENVVTMGITPSAECIVGKYMTYIGVETPYGIR
RTRRDPNTDIYILFNPWSPADPVFLDDEEEREECVMNELGIIYHGAYDDVSERAWNYGQFEFGVLDACLFVMDKADMPLSNRGDVVKVTRVASA
MLNSRDDDGVLVGNWSGDYMYGVPPTSWTGSVEILLDYANSSGTPVCYAQCWVYAAVFNTFLRCLGIPSRVVTNFFSAHDNDGNLKMDIILDEN

GKLDRNRTKDSIWNYHCWNECYMARPDLPSGFGGWQVVDATPQETSDGMFRCGPASVAAIKHGQICYPFDAPFVFAEVNSDVVFYRRRKDGILE
VVKVNQTHVGRMVLTKAVLHSGRRDITNQYKFPEGSPEERRVLEKAEEFGCQREKSSPALSDVDVEIHSLDVNVGENFDVTLQFTNRSDQRRTA
DVYITGTVVYYTGVPSGEVVFKTPKVKLEPMQSKEEKVLVRSEDYMNKLVEQRNIHFIATGKIKETGQIITAMKVIAMHHPKLTVKVTGSPRVS
EEMYVSVEFTNPFKFSLENVDLRVEGPGVLPFKYKQYSVIAPGTSITWTEAFSPRRAGSTKVFAKLDCAALRQVYGETELTVQE

>shark_TGM1
MPSSHVPLPSSEVGRRPEAAIAPTVPTDAERRAGVEHGAKKSGWQRCCPWLSSCCPCGRKSSKSYDVADAEVEEEAGETTDGGKDDRLEVTNLD
LLKGSQEVNQQRHHTAEFECTELVVRRGQPFEIRLRFNRPYDPQSDTVRLELRTGDNPQPAKGTHVTLAPVEQPERGQWGAEVLDRTGGGRGLS
LSVHSPPGCPVGRYRLSARTQDPAGPGKWLEEAVYILFNPWCPDDVVFVDEEKMRNEYVLNETGRIYYGTEKQIGARTWNFGQFSKGILEACLF
MLDRSKMPVAGRGDAVSIVRVISAMINSQDDCGVLVGNWSGQYTGGTAPTAWIGSGDILLQYHRTGEPVQYGQCWVFSGVVTTVLRCLGIAARS
VTNFASAHDTDVSLTTDVFLDEEMKPLDHMNYDSIWNFHVWNDCWMARPDLPAGFGGWQAIDATPQETSSGQYCCGPASVEAIRNGMVYLKYDA
PFIFAEVNSDKVFWQRQTDGTFQKVLVLKKAVGHQMSTKAVGSDERDDITESYKYTEGTDEERIAVETACRHGSRPDTYADQYTASDVEVTVHT
DDGIIMGSDFTVTVGVANTGSECRSLTLFVQAIVMYYTGVAKGSLKKDKRDVLLEPHEKKEVKLVFHNEDYLEFLVDQAAMMFTVTGRVKETGQ
AIVNQHTFRLRTPDLMITPLGDAQVGKPMKVEIALTNPLPKSLKNVTLRIEGPGLQNPGKVNIGDVPRHASITVTETLVPLKPGCRKLIASLDC
QQLTQVHGVAEVLVQES

>shark_TGM2L
MLAEPGKPTAECVVQDVSSVDFHCEKNNPDHRTAEISAKRLIVRRGQPFHITVQFKRNQYNPDVDRFKLVAQTGPSPSETSGTKILFSLSDSIN
KRRWNAVAACSSRSRLSLIIHSAPNAKIGRHTLALQKITSDQTVIYTVGEFVVLLNPWCSEDEVFLNDAGQLNEYILNEQGIIFTGCSEYIQHL
PWNFGQVNCNDDNGILFGKWDAPYTDGVYPGKWSGSVAILRQWNNSGCQPVCYGQCWVFAAVACTGVYCCGPAPVKAIKEGETDICYDVPFIFA
EVNADCVISVYSEKGKKMKVDTDMRHVGQRISTKCVGSDDREDITNNYKYPEGSEEERRIFELADKRRVPLKPGKRLQLHLMTNDPNYNGTNVK
VSAVISNKNSMKRVYTLKINAMKKKYSRSSRGKCIQMYQQEITVAPSEDKTMEVELSYTEYGELLDKYNLIRFTALAIDGETNESVFTLKDICL
INPNITIQVRGAPVLSQEVMTEICFENILPVALTNCVFTLEGAGLIDGQMEIRIGALNPGEAITKEVSFIPKKMGLKKLTVEFDSDNLKDVKGY
INIDIQEEEK

>shark_TGM2L2
MSNMSMSVDPVPSNAKCQVDFQFEKNNKEHRTDKISTKRLIVRRGQSFSIKVNFTDGFNPNDNKLKMIFETGPDPKKLNGTKVEVPFTKSINLK
RWSGIITSSTSNKLCIAISPSPRAKIGYHRLILEHAYKSDVQYHLGNFVVLFNPWCSEDEVFLNSDLQRDEYVMNETGIIYVGSSDYIHDVPWN
FGQFEEDILDICLKLLDKTPKYLKNPNKALRRRGFPVYIARIVSAMVNCNDDTGILYGSWSPPYSDGVYPGKWNGSVAILRKWHNSDCQAVRYG
QCWVFAAVTCTVLRCLGIPTRVVTNFDSAHDTDANLTIDEYYNVEAENLGESADSIWNFHVWVESWMARNDLSPGYDGWQAVDATPQEESDGIY
CCGPASVNAIKEGEMDMQYDIPFVFAEVNAHCVDWLVFNSGEKMKMKVNESRVGHKISTKRCGSEEREDITSNYKYPDGSVQEADVFEKANRMQ
NIPTPEKTLSLSIVTELPIYNGKPIAVSMVVSNNTSEQKVYNLRFWAKKRKYNAVTENQCIKKHEQEITIAPNTEKKIPLKVDYKEYGLFPDMY
NLMKLISVVTDVSSKSSAFAMKDVSLINPPLIIKMLNFSAVVNKKVHIEISFQNSFPETLKNCVMTLEGAGLIEGEKEIKFPNIAPNEEAKVKC
DFVPYKSGMKKLLVDFDCDKLRDLKGSMNIIVQE

>shark_TGM2L3
MNVDRLLVCTTVTEGALKRTDRLIERSLTTLAEMNVAVDFQCEKNNQEHRTAEIDTTRLIVRRGQPFHITLQSYTDEYIDDDTTALSAETGPKP
STTSGTKVLFALNSFSTNGWIGKVTYNTGTRLTLDISSSPNAKIGRYSLSLLAIKGGLVSSCKLGEFILLFNPWCTEDEVFLDSEEQREEYVLN
EDGIVFMGDNHCIQSRSWYFGQFEKKVIDICLMLLDMNLKCLKCPGRDYVRRNDPVYISRVVTAMVNCHDDNGILEGKWDGPYTGGVLPWNWNG
SVAILHRWYNRGYQRVQFGQCWVFAAVACTVLRCLGIPTRVVTNFNSAHDSNGNLTIDTLYDECGRKYGRQSESIWNFHVWIESWMARNDLRPG
YDGWQALDPTPQEKSEGIFCCGPAPVNAIKEGAVDMKYDVPFVFAEVNADQISWIMHRDGRKEKIHVETQHVGQNISTKSCGRNGRDDVTHSYK
YPEGSAKERAIVSEADLTNRLCSQPENKLHVHVKTEKSINNGSDTQVLITISNRSSTNMVCKLNFNAHMKSYDGKLMRQITEKNLEQIAVQANE
DETVALEVAYSHYGDYLEHHHLIKLTALAFDMVTKESAMAMKDLLVINPDIAVQIHGDPIVHKPLTAEICFTNPLRVPLNRCVFTVEGVNLIYG
MEQFNIDEIKPNQTMTINVEIIPKKAGLRKLMVNFDCDRMKDVKGYKNFTVQCFNL

>shark_TGM2L4
MAQALAVAGTDFQCEVNNKAHRTADFGSNRLIVRRGDRFTVTVHFAGRGYQGAEDQISVIVETGLAPSVTSGTKAQFPLSNSLDESKWNAALVS
SAGNQLSLSICSPANAKIGHYTLKLCTTQGQSTPFDLGKFILLFNPWCSDDAVFLDFENQRKEYVLNDQGLIYQGTKKLISHIAWNFGQFEDGI
VDICLKLLDNSSNCLKNQEEDCSQRHDPVYISRIVAAMVNCNDDKGILQGNWGPDYSCGVPPTMWNGSITILRRWNKLGCQPVRFGQCWVFAAV
ACTVLRCLGIPTRPITNFNSAHDTEQNLRIDSFIDENGKISKKSKDSVWNFHCWIESWMTRPDLKPGYDGWQVIDPTPQEKSEGIYCCGPASVK
AIKSGDIDQKFDSPFVFAEVNADYVSWLLCKDGSKKQIEVNHRLVGQNISTKAVGSDEREDVTHNYKYAEGSEEEREAFTKADMKNKLTQEPEK
KFFLKLKAEKVNLNGADFEVSAVLSNQTSAVKNCRLILCAKTILYNGQSIQECSWKDLAKLTIRPHEEKTETLQVHYSNYGQSLTEHNQILIVA
LAMEYEAGELVVTRKVITLQNPDLHIKIIGEPVQYRDLTAEIYFTNPMPVNLCNGTFLVEGAGLTDEQKVPCPVQSIKPGQEVKVRVKFTPQKP
GLRKLAVDFDCNKLKDVKGFKNVIVRPANK

>shark_TGM10
MTTNELYLGAVDLRCEANNTEHHTIEIDKDRLLVRRGQEFRLHVEFKHRAFVEGEDQLAILLDTGPAPSEADGTRIKVLNSGVKWDKWTFRLQC
TPGHIHLAVHSPANACTGYYQIYLILYPTGGESIQRITAGDFHLLFNPWCKEDAVYLPDEDLLQEYILNENGLLYHGSYANIYTLPWNFGQLEK
DVIDICFLILDNSLSALKNPLADAPRRNDPVYISRTVTAMVNANDDKGVLLGRWDGNYSDGIAPTRWTGSLPILRLWTSSGADKVRYGQCWVFA
AVACTVLRCLGIPSRCVTNYSSAHDTDGNLKVDQYYSSEDYSRVPSKKKDMVWNYHCWLEAWMTRPDLPPGYDGWQALDPTPQERSDGIFCCGP
CPVKAIKEGHVDMKYDAVFIFAEVNADVVYWLVNKDGSKKELGVKQHQVGKQIITKSAYTDEREDLTHDYKYPEGSAKEREVYNKAGMKIRSTN
IQQKDLKISIKYAQPILGSDFDVYFSIVNHGFMDKDIHLTLTATTVTYNGFILTEFSKRSTTFMLKAATVQKEVLRLKYKDYGEHLSEHNLIRL
TAMLTIEGTSEVSLKECNVALNLPQLTVKVVGEPILYRELTVQIKFVNPLPITLTGGIFSVEGTGLTDLKEIKSPTAAIHPGQEVVVNASFKPT
KTGLQKLMVDFDSNRLRDVKGSTNIIVRRA

>shark_F13A1
MSASSSDDSNSRRPLASYVGRRITPSNDSNAGENEVLDFEYFGLTPRGPPNLEDFLEVWNVDVHPNPDDVNKKQHHTELYDCRNLIVRRGQPFQ
ITITFNRAYDSGKDKLWVEFLIGQYPDITKQTYAPVYIQEQLEKGKWGAMVTSTRTNTLSLSIVSPPHCIVGRFRMYLAVMTPYGIRRTARDSE
TDIYVIFNPWCREDAVYLDNDRENEEYVLNDAGRIYYGKFTEIISRPWIFGQFERGILDSCIYILDRARMPLQTRSCPIKVSRVASAMINSKDD
GGVLVGCWDGIYTNGVAPTAWNSSVEILLQYFETQLPVCYGQCWVFAAVFNTVLRCLGIPARLVTNFSSAHDNNANLTTDIILDENGKKDMNLT
KDSIWNYHCWNECWMSRYDLPPGYDGWQVVDATPQETSEGMFRCGPASVNAIKHGQVYFPYDAPFIYAEVNSDVVYWNRQKDGSLIKGNVKTDE
VGMLILTQEIGSDGRKDITDQYKYPEGSQADRAAQGTAIQYGIKKEEAVAETAVDATLNVHVPEKILLGTTFDVGIELQNNSSEKQHMTLHLNG
CVVFYTGVPKIKIKDKTIKAMVEPHQVYQTQVKIKSKDYEDHLVEQSILHFLVSGHVAETEQTLAAQKIVTLQIPQLNLRAEGPAVYGRETIII
IEFTNPLKKPLEEVILRVDGLDTLKPKLKIFSSIPESATLTTKELFVPWRRGTRKVIASLDCKALRQVVGELELNVI

>lamprey_TGM1L1
MADREELGRQYAEGRRHRLSSVCPSPVGSGQFPGIGFPPGRTAPSKGHSLRRLCCCCLPARRAPGGEDDHTAPLSSAKNQHAAEEEGAEEETEL
LGERLAVLRVESAGKEEAAREHRTGGFAQPQLVLRRGAPFRLRLKLSRAVQRHHDSIVLEFSTGLSPQLRKGTLIQVPLRWELPPEQQEGAGGQ

RDGGAEGQRQEGGGEEGGGWSAVVEREEERALHVLVRSCPRSVVARYEVAACAHSETAVEEVRSKREPLSVYILFNPWCREDTVYMESEEERQE
YVLRETGLIYYGTKEQISARPWVFGQFGKGILDTCLHLLDRANMPLYGRNDPVNVARVVCGIVNSQDDSGVLEGNWSGDYTEGTSPAAWTGSAE
ILLGYHKGARPAPVRFGQCWVFSGVTTTVMRCLGIPCRSVTNFCSAHDTDVSLTTDVYVDEKMKPIDDLNTDSIWNFHVWNECWMARPDLPMGY
GGWQVIDTTPQETSLGFYRCGPASVNAVRDGQVQHKYDAPFVFAEVNSDRVYWMKQPGGEFSVLSVDKQAVGHCISTKAVGSHEREDITGLYKH
PEGSKEERIAVETACSQGSKANIYKVPGTAQDVRLLVKPPVDEVALGRDFAVTLELANLDADETRTVVLFCAAHGMHYTGVLRGRVKQHSWEVQ
LAPGEECTLSLPVSASEYLQSLVDQCAMLFTVTGRVGETGQTLATQRSFRVNFPALGVTVDDRDAVRVGNKALVKVSFTNPLPQPLRAVTLRLE
AAGLLEPTLIQHGDVPGGGSVYRTVAVVPRVAGVATLLATLDSHQLITVHGELELLVKA

>lamprey_TGM1L2
MWEDFGDFHAEAEGSGARRGKTSAPRSFAPIDTVLTAVEDTTGSSSSEVAGGRPRTTGPPGAGGVPLLGVWAVELHGEQEGRTRLEHRTERYET
PLPVLRRGLPFPVTITFSRPFEPRSDRLQLELYIGKYPRIDRGTYIPIPLAEPGEEVEPGTWKAKVTGSEGARLALLVHTSPKCIVGKYRFHVA
TLSAGGLYRSSRDPNTDLYFIFNPWCPEDSVYMESEAEREEYVLNDIGRIFHGTRDQIVSRVWNFGQYEYGVLDAALSVLDCGRLPLPGRDNPV
TVARVASAAVNSQDDNGVVEGCWVDNYTGGVAPTAWNGSAEILLDFKMSRRPVKYGQCWVFAGVATSVLRCLGIPSRPVTNYCSAHDRDASMSY
DVYLDESLAPRDDLNKDSIWNYHVWSECWMARPDLPLGYGGWQIVDATPQENSEGIFRCGPSPVNAVKSGIVYLPYDTKFVFAEVNSDKVYWKV
GLDGELSPVDVERRAVGHCISTKAIGSDNREDITHAYKYPEGSDEERVSVEMACRYGTKPSLLAEALGSRPNHDVVLEVTAPLDGALTMGRDVA
LGVRLKNHSVDGEERHVSLLLHCDALFYTGVVRTAVKRQRFDLDLPPGAEHQVVLQVRRGEYLGMLVDQGGLMLTATGRVLETSQPLVAQRAFH
LTLAPLRITVLGEVRSGWDFMAEVSFTNPLPTVLQGVTFRLEATGLQQNKVIRHGDIRVGETVTVRERLTPTRPGLRKLAGSMVCRQLTQVLGD
TDLNVY

>lamprey_TGM1L3
MLGHGNLETAQPAATRSNVVGRFAGAPAAPLADGPGGHPNGAPPGTPGTPPGPPPPRSRLGRCCDWFFCCRCGSRAAGGPGGGRGGPGVAGEVE
MKPREPPATPGGPGVPPMPSADEDPAGEPTPVDPVTGVSRLEALWVDAGGRGGGGPGAWNRARHRSAEYAAGHVVTRRGGPVLLHAAFSRTPTD
GVDRVSVELEIGSHPQLNKGTHLIFPVETRAAGEGDVAASSSSSAAASSSSTASYDDDTDCTSGAWWASGGLTEAGGANGRNRALWLRVRADCL
VGKWRLFVSTQGPAGSHRSARREESDIYIIFNAWCPEDAVFMEVEAWRREYVLADTGRIYYGTEQQIGSRPWNYGQFEKDVLEASVMIMDRASL
PLAGRGCPVKVVRIVSAMVNSLDDDGVLEGNWSGDYGGGTAPTAWNGSVDILQQFARSRSPVRYGQCWVFSGVTTTVLRSVGIPTRSVTNFASA
HDTDTSLTTDVYLDDDLQPIAELNTDSVWNFHVWNDCWMTRPDLPEGLGGWQAVDATPQETSSGVFCCGPASVAAVRNGLVYLTHDTPFVFAEV
NSDRIFWLRSADGTVKRLDVERRAIGHCISTKAVGSDTREDITHLYKFPEDTEEERLAVETACLHGSRPDTYRGSPSSPDVTLAISTHERVVMG
HDFDVSVTVSSTAVSERQVSVFVRGAVMHYTGVTRGEAIKQLKLDVTLQPGEERVLTVTVRQGEYLERLVDQSAVMFLVSARVNPGNAILTRQH
CFRLCTPKLLVTCADECRVGEPLTVEVLFENPLPHLLRDVSFRLEGPGLQSPRVIKHGNVSRGGVVRLRETLLPERPGQRKLLVSLHCTQLSQV
HGETDVLVSP

>lamprey_TGM1L4
MPRDTVGERLAVLRVESAGKEEAAREHRTSGFAQPQLVLRRGAPFRLRLQLSRAVQRHHDSIVLEFSTETAGPPVSVPVGPRLREETWSATLSE
ETSGTRGGRGGQGVEIEVTVQSPPHCPVGQYRLAVAVSPAGGRPVRTEPGVCPDVIILFNPWCRGGSSCVLIKNRLESILTNTYLRDPVEDGTR
DAVFMEPEEHKTEFVLNETGRLFYGTAQQIGTRAWNYGQFTSGVLDASLHLLSSMAWEQRGDPVAVVRTVSALVNANDESGVLEGNWSERNDYS
GGTAPAAWTGSPDILRQYHRSGRPVKYGQCWVFAAVTTTVLRALGIPCRTVTNFSSAHDTDGNLVTDIFLDEKMRPDRSHTKDSIWNFHSWSDA
WMKRPDLPSGYDGWQVVDATPQEASAGVFRCGPAPVMAVKKGDIDLKYDTAFVFAEVNSDRVFWRRRAGGERERISVETDSIGQKISTKAPGSD
ARLDITAEYKPSEGNRELGQQEGSSYGTRGNKGVVTEAIVVAMSACSEEERRVVTHALSLLKRGGAERGPREVETGGSGPPQGDNASGAGGPSG
VERGRDAADIGGALDVRSVLNPDGHLIASLGGDPIGAPAGGDPPPPEVTLAVRVLEETALGRELTAEVRVASASARPRVHVRVRVRGHGATYTG
ANLGGFEEEEALLELGPHEERRVLVRVPASEYLHLAVDHFALVLVACARVLDTGAEVARVHVARVRSPRLRVHVPEGAHVGRDLISELCFTNPV
ARALHRVRVRVEGAGVHTPGTILVGYGAVVTSSRS

>lamprey_TGM10
MEGTGHHRTSPSESTVLDFLETHPELLESHILGHCTADQVNRWLCKLQGGGGGGLASSPSKSRSSLLARRGRPFIIDVHLGDSYRFEPDRDELS
LSLRLGPNPQESDRTLILLSSNGAANGDGGGWSLDVESLAAEEEEEAAIEVPEPGTTAWEERQSLSLDLSAVAMGGGEAGGHPAGGRLRLSVRS
PADAIVGEYLLSLELIVGVEPLQITGFTVGKVWMVFNPWCPGDAVYMEDEIGVNEYVLNEKGLIYMGSNDYIYSIPWDFSQFADRVVEICFQIL
DNSNSALDDLEKDVPRRADPIHVSRVVSAMINSNDDRGVLNGRWNEHYTDGTNPMHWAGSANILRQWSDSGFEPVRYGQCWVFAATACTVLRAL
GIPSRCVTNYLSAHDTDGNLACDRFFREEDLQQVLKGRNDSVWNFHCWVESWMARPDLPPGNDGWQVIDPTPQERSNGVFCVGPAPVVAVRDGE
VRHPYEAAFVFAEVNADVVNWVVTAGGEKRRANCFTGFVGQNISTKHVGSDEREDITHLYKHPEGSAEEREAFERAGRGARDAAVVLRDLEALI
KLSASAFVGSNVDAHTVVRNRSGRDLTLTLTSAAAALTYTGQCGAECGARSCSVTVPAGQTARETLRVKYRDYGEHLTDQNLLRVTSLLEEPSS
GQLVIMERNITLKRPRITVTIVGEPRQNRKIKARISFTNPLPSKLIHSVFSIDGEGLTSLQKIPEPAAEIGPGETLTVTAELAPSRPGTFLLSV
VFNSDRIRDAHESKRVIVRP

>lamprey_TGM10L
MDPLELESFERLEVANQKAHHLEALTVEKDRLFLRRGQPFQLLVKFRGRLFNPGNDAIALALHTGPSPSQQDGTLLRASSDSAGELSVTAHGSG
HTWSLLEASTPAGAPIGRYTAFLETRLAKGGDSTVRCFPLGEIVLLFNVWCKDDAVYMEDDLLRSEYVLNETGKIYVGSHDYISSVPWNFSQFA
QGLGDICFQILDKSNMALEDLAADTKKRGDPVHVSRVLSAMINCNDDKGVLQGNWSGKYPNGTCPTSWTGSADILRCWHKTQCQAVKYGQCWVF
AGVACTVMRFFGLPTRCVTNFTSAHDKDGNLSCDRFYYESNNKQESHDSVWNFHVWVESWMSRKDIEEGYDGWQVIDPTPQERSEGMFCCGPAP
VRAVRKGQVSLGYETPFVFAEVNADVVMWSVAADGRRTRMSCNTDHVGRCISTKRAGCDEREDITLQYKYPEGSKEERAAYARATCIVRGERPQ
PGVDDKYSRPLVPNGAGERGEPQGEVVQGLLVRTRLSRSAFVGSDMDVHTSLRNPSRRHAAVVRLVLCARAVTYTGMVREICTQRECTVEVPPG
ETVSEPLRIKYKDYGSQLRDQNIIRVTCAVTLPGQETILAFSERNIILETPEIHVMVLGEPVVGRELKVEMSLVNPLPQPLDDGVFSLEGPGLT
SLQQISCSDRVEPEKNVCVSASFCPRKPGLHNLVITFNSNRLRNVHGEARVIVRRA

>lamprey_F13A1
MSSAHGLVPSPTSTSTWTDAGRWSASRIAARAALSSDCTGGAVEAAAAAGRVAMDIRPLKPSWPWHLAGSDFPGNDPGVGRRRHLAVLSVDQLI
AANRARHRTSDYEVDNLVLRRGEPFDLQLKLNRPYDPIRDKIRLELQIGRFPLESRGSLVVAEVSGWNSESRFGATVSRCSGNELTISMNLAPN
CIVGRFEMYVVTESDGQPNRTRRDPNTDTYVLFNPWCREDQVFMSDESWRREYVLNDTGCLYLGTSQQIVTQHWNFGQFERGILDVCLSLLDKG
DLGMSFRGSPIAVSRTTSAILNANDDXGVLMGRWSGNYRNGVPPTAWNGSVDILLRYGRVGRPVRYGQCWVFSGLLTTVLRCLGIPCRSISNFS
SMHDQNANLTMDTYLDQDMWPIDSLNHDSIWNFHVWNEAWMARPDLPGDNGGWQIVDATPQEQSNGLMQCGPAPQVAVKNGHVHIKHDTPFVFA
EVNGDRVYWVWGKDWRFHKVYTDTSVIGKDISTKAVGSMRREDITLQYKFPEGSREERDTMALALAGGIDFVPYSPSTSGDAARRVPSPASPPA
SPAAADDAVAVERRLGPGDLALSFAIGDAAVGSDFAVRVRVANGKGTAATATVTVHVQGDFASYTGASRQRFKRDRATAVLEPGETGELEFPVL
VSDYLLHLSEHGTMNFTASARVEETENVVTKVRAVSLRAPTIDIAAKGEARVDTEMHAELTFKNPLPVCLTDVSFNMEGPSLLIPTIRTFRNIN
AGESVSLSQSFTPTKAGERTLMASLQCKELYIVTGTLELAVSGRAVAADQAL

>tunicate_TGML1
MARGRRGRPRGTGRGRSPSKDALAVKSVDLLKSDNTVQHYTSDYEGSELVVRRGQPFKLLITLSRALKKEEEVEFELRMGGRPMVAHGSLIPLK
VIDPKPDEDVVGFKLLANAGDILTVEIYTSAENTGVGKWLLALRAMEGRKKLPRMTVTDDIIIVFNPWSKFDPVYMENEAWRDEYVLNEEGLQF
YGTSRRHGKMEWVFGQFEAHCMKAAMKLLLMGSLRYKDHKEPVMVARHMSALVNSNDDNGVLVGNWSGDYSGGRSPSFWHGSTAILKQFVKTGK

PVNYGQCWVFSGVLTSVLRCLGIPTRSLTTFDSAHDTGGNLTIDKHYNETGKPLENDDSIWNFHVWNDVWMARPMLPEGNGGWQALDATPQETS
DGKFQCGPMPVSAIKEGDINFDYDGPFIYAEVNAVEKHWRKLKKPQVVDGKMIEYSEIGANTTKVGKLILTKAVNSWAEEDITHSYKYPEGTKE
EALSFKNARKHVKFTYVPKKEVKNLVLFKPDVPASVTFGKDITFPIKVENVSKSSQNVFISVVAKSVQYNGSIVKEILDVDLSDDIAAGKTHTF
NVSLPFNTYKDSTRGENDVKFFMLGGVTGNEDQVFSEQDLVDLEKPDITVKVPASAQVGKQINVKASFTNPLSISLTGCMFTFEGAGIRDETIV
EVSDVKPKAVSSVDVKITPRVVGTRKVIVGFSSKQLEGLRGNAELKVA

>tunicate_TGML2
MSFFGRLKKCQCIGAPEEPKPVEGLQLKTVDCDWMSSENASDHHTTEYSAKYLILRRAQTFTMKMKFQRKFHKTADRVVLELSLGTGSQLMNET
KVRCPLVATLDPTKWGMVITHEEDETFLVTFTVNIPPKALVGKYKSVVEFTSQLESGESVTTRDNEPELAILFNPWSKLDSVYMENEAEREEYC
LNDLGIVYRGSKVRISGKKWNFGQFEENILECSLLLLDKDKRAKEKPNKWIQKRGDPVWISRAVSAMVNAQDDDGVLVGNWSGDYSGGVSPTKW
NGSVEILQQYYNTGKPVSYGQCWVFSGLVTTVLRSLGIPTRSVTNFASAHDTEGSMTIDNYVDESGEEINLGGDSVWNFHVWNECWMKRGDLPT
GYDGWQAVDATPQEISLGLYQTGPAPLTAIKNGEVYLGFETAFVFAEVNSDRTNWIVKQDEGGEYVIETLGSRFPKSVGKYISTKSVGTDDRLD
VTNLYKYTEDSAEEREAFKKAYAFGTLPEYQAGFLSVEEEGKGVSIDFSTAPNIRNGDGFAIVITAQNDSAEKVTVDISAVLHSTLYTGEKRRF
IKRQRFSAIPVDTNSTVSRDFNVMFSDYNGKLVDLNSLRLSAVVKVKETGKMFADSYEFRLDNKEAIDIQVENTLQINKEYQVLVNFSNPLPTR
LTNVVVTLEGPGLSEPLTRKLNRNVVAGGTAQFSFPIQPKKVGKKSILVDVDAKQVKDLKNFIDVEVVSS

>tunicate_TGML3
MGNRSTKEEKDAENAEEVNEFLDEHPRFLGEYIDRNPHLLDNYVIDNVDEETIQKWLDKVSGPAPTEVLQILRVNYKKETNCVDHHTDKYIHNS
LVVRRAATFDLGLVFKNRNYRPAKDDIVLEFTIGSDPTIKNETKIRVPVGDSLQGSKWTCMKINEDEVTKEVTLQVNIPPDAIIGRYKLTVEVA
TELKDGRQKERKVKPDIVVLFNPFKPADPVYMESSVEREEYVLNDTGRIYVGQWYRIGAKDWLFGQFEEGILDIALKLLREHTNAQKNATKSLK
KRASPAYCSRLLSAMVNCNDDNGVLWGRWDGKYEEGVKPTTWSGSVAILKQWNQTKMNPVKYGQCWVFSGLLTTVLRALGIPARSITNFNSAHD
TEYNMTIDKFLTEDGESAEGTGDSIWNFHVWNEGFFRRPDLPKGYDGWQAVDATPQEESSGVMQCGPAPIKAIKNGEIYIGSDTNFVFAEVNAD
RVFWEVNDEGEVTKMVKNDKRHVGRNISTKAVGSDEREDVTLQYKFAEGSEEERVAFERAYAHGRKAPYHEKFVVEEEGNIKIDINPVGDVING
SDVSISVKVTNAKGVDCDATITTVIHTMLNNEERKRLLKRSRGTRKIAAGKDDVESFKFGFGDYGRHLSDENVIRVTTTVRVKETNKLYVDQYD
IQIESPQCLELICADELKVREYQPIRFKITNPLKVAMTSAVFSLQGSGISSGKSFEVPSPIEPGETYTSPEMEVRPYRSSRATTILGDFDCNEI
WNIKARKRVSVNF

>lancelet_TGM1L1
MSIRMPDGTPVVLNSGTESGGDDEMAGRATRAAGRHAPGGGTNSASDRSSRYNRLSELMKGKREAATAHQAQGPGYNRGTYTSHTTTNNSYQPG
SFMAGGAQGGYMAGSTGFSSGYRSAPSGGGFGGYTGAAGGRFRAADVFPSVFGGTGAAGGSGSTYPRAGMGGGFGGGHHSGGLGGGHAGFAPPS
AAGFQGGFSGGGGFGLNDFSSTTDGLFDPHRPQNFGPGGIRMGDPLDFDDLDPLTPVTPDIPTPSFPDHLRRQPASFTETTRTSYFDRFKTSES
SSPKESVTQNGEDHLFISKAYIELERGGRKNLNPMVIQEPPSEDDGPLKLTNIDFLNDENGRAHHTDEFEEHNVVRRGQPFTVELRFEKTYDEG
EDKLKVELHFGDNPLPNKGTLLRMPVGKSLEDGKFSAALDSSEGGYAKVKITTPPDAIVGKYHVVIETMSDGKTFRSRKTDDNTVVVLFNPWVK
DDMTYLDDDAQLGEYVLNEHGYQYYGTSRRIGKRPWNFGQFEPKILDVCLRLLDRSQIKAESRGDAVKVSRIVSKMVNSADDGGVLTGNWSGNY
AGGRSPTAWNGSVEILRQYYERNRPVCYGQCWVFSGVMTTVLRCLGIPARSVTNYDSAHDTDVSMTIDNYMDHNLREIDGGDSVWNFHVWNEAW
MARPDLPEGYGGWQAVDATPQETSDGVYCCGPCPISAVKNGHVYLPYDTKFVFAEVNADKVYWLVDRHKNLRKLRTRKAAIGNKMSTKAVGSNA
RHDLTENYKYPEGSDQERVAVRTAVSHGLKPNTYDDVIEDEDVEFDVHADDEIYIGQNIHVTLSMKNTSSEPRKVTTHLTARAMYYTGVPAHDI
GELEKDVFIPPNGEASVEMTFTPREYLDMLVDQAIVKIHAMAHVDDTNQVYSGQDDFRLMSPDLTVKAPTKMTLGEQVTAEIEFTNPLDVTLSM
VEFHIEGPGLQKPKKISHTPIKPGETVKVTERMTPRKVGKKTIMASFTSNKLTQVTGELDVVVS

>lancelet_TGM1L2
MPRRSRRQAAVRASSRLRDYYSGVTPGVPSRRRHASEDSTYSGGDSNVHKPTARNTGRFQVLNPDKKRQNKVIKHLEKPVVPPEKDEQLKVTNI
DFREIGNRLAHHTDEFEKQVLIVRRGQLFDIGLDLNRPYSQAKDKIILEFRIGKYPKPSKGTLIKVTLGKEQLTTDKWGAKVQQTKGNFVGISV
MAPANAIVGKFDFYVQTEHEGTKFRTAKDDKNAVIVLFNPWCRDDQVYIDDKAKLNEYVMNETGFIYVGNKRKIRGRPWNFGQFDDPVLDASLY
LLDRARMAYTSRWNPINITRVLTAQINSLDDDGVLVGNWSGDYDDGVEPWVWNGSVKILEQFLKTKESVQYGQCWVFSGVTTSVLRCLGIPARS
VTNFGSAHDTDSNLTVDYHFDDKDEPIEDLNNDSIWNFHVWNECWMARPDLPAGYGGWQAFDSTPQETSEGQYCCGPASLNAIKNGHVYYGYDT
KFIFAEVNADRIYWRVPEDGDDWEQLRVEKYSVGCHISTKMVGSDEREDITHLYKHPEGTELERVAVREAVEHGNRPGTYVDRSIPEDVTFSID
TDDDVDIGEDFSVRVKLTNQSDQERYVTMYLTAQAMYYTGVSLGKFWNQRFTVRLTPNGDGEVKGRIDADLYVPRLVDQGGIKFFLMGHVRETQ
HLLNIVHGEVEGRIDADLYVPRLVDQGGIKFFLMDGEVEGRIDADLYVPRLVDQGGIKFFLMGHVRETQQSFAGQHDFRLGVPDLTLKVSDKDR
IINIGQEVHVNVSFKNPINTALTKSAFYFEGPGLTADDKVIHHKDIGPRETATAVVKLKPKKKGKRKLMVSFTSKQLQQVCGECVLDIV

>lancelet_TGM1L3*
MPMLVEKPQSADDDATGADGPLRVTEITFLKEENGPAHHTDEFVENCIVRRGQPFSADLVFDKDFNKENDKLVIELRYGVNPLPNKGTLLRLLV
GNNLKEGEWSAAVEKAEGQNVKVRITSPADAIIGRYVVVAETTSNGKKWRSARTIISYFVVLFNPWCKDDRTYLPDDAKLEEYVLNEHGYQYFG
GKDNISKRPWNFGQFEPGILEVCLWLLYRSGLTVNSKRTAVWVSRAMSKMVNSNDDKGVLVGKPFGPYLDGTAPVAWNGSVEILLQWFQERQPV
KYAECLTFSGVMTTVLRCLGIPARSITNFNSGVDVDYSMTIDSYLDDQGVPKPSSDFFW

>lancelet_TGM1L4*
MIDVEAWQVWQDDLQQNNRSHLARPNLSHSSAHLQTGRTMAERPRGFAFDTGRRVRRPYANFPMIIESPWWPPLPPVGPVEPPIVPPPPVGPVE
PPPIVTPPPKPTDRPLTLGNFIFHVNENGPAHHAEEFLENNVVRRGQPFSVGLLFDREVDLEKDKLLIEFQYGNNPLPSKDTLLRLPVGDALKE
GEWSAAVEKADGQNVKVSITSPANAIVGRYHVVVETELAGERSRSRKVDIRNVVVLFNPWCKDDLTYLDDNTKLEEYVLNEYGCQFFGNYYRIG
KRGWNFGQFEPGIPDVVLLLLEKSGLGPQSRGSAVSVSRAMSKLVNSADENGVLEGNWSGDYSWGTPPTGWNGSVDILRQWGTERRPVRYGQCW
VFSGVLTTVLRCLGIPARSITNFQSAHDTDVSMTIDNYFNKLGYPVDSEDSVW

>lancelet_TGM1L5*
MAYDDVIKKEDVEFYVNTYAKIKMGEDIQITLYMKNTSSEPRHVTAHLTAKASYYTGTPAAKIGELEQDVTIEPNGEGFVEVKYTPKEYLDKLV
EQSIVKLFVMAHVDTTKQVYVGEHSFRLVSPDLVLKGPSEMTMYEQSKVTVEFTNPLDVKLMDAVFRIEGPGLQTPKTIPYPSIGPKKTVTLVE
PMTPRRAGKRTIVASFTSDKLQQVTGEMEVVVKKE

>lancelet_TGM1L6
MGKCVSKNREEKDLQPVNVEPVANARTARSAGNAPGPRRLTLLAENLDQCLQVSDVYFRLSYNRRAHHTEEYESFQLIVRRGQPFQLSLAFDKP
YTEENSQVTLEFHTGDSPKISDGTLARVPVDSPLGGTSWGAKVTDKGLKTIDLLVQAPPNAIVGKYSIVVETILDGHKYRTERDPYSHIYILFN
PWCKDDSVYLNDEEELQEYIMNESGIIYVGNKRQVMERPWNYGQFEMPVLDASLELLDKAKFPPRARWDPIRLVRVVSKMVNSQDEDGVLVGNW
SGEYEDGIRPSAWNGSVEILQQYHVRKEPVCYGQCWVFAGVTTSVLRSLGIPSRSVTNFASAHDTDGNLVVDIHYDEEFTPIDYLNTDSTWNFH
VWNEAWMTRPDLPAGYGGWQAFDATPQETSEGHYKCGPASVHAIKNGHVYYGYDAKFIFAEVNADSISWVCPEDGPMYQVWQQEKGIGVKISTK
AVGKDEREDITDHYKYSEGTEEERLAVEKAVSHGSRPHTYGERPEKADVEFSINAPKEVIVGHDFTASVTLRNHSNQERSVSMFLTAHTMYYTG
VPVTQFHKEKFVVTLQPNGQMTLHAQIEPAQYMSYLVDQGAIKLNLLGRVKENKCTFAGQTDFRFSVPKLNIHIPARERSDFPVGEEFVVELSF
KNPLAVKLTNTEFHVEGPGIQKPKLISYRDVRPLEAARTLVTMKPVRPGSRKIIANFQSKQLHDVQGHLEIQVTEDDVIDE

```
>lancelet_TGM1L7*
MARPDLPPGYGGWQVVDSTPQETSEGVFQCGPCPVVAVKNGQVYLPYDTKFVFAEVNADKVYWKETEWGYYDYLRHEKRSIGFNISTKQVGLNV
REDVTENYKHQEGTDQERAAVRAAVEHGSTPWTYESIITHEDVEFSISADEEVYVGQDVNVTLTIKNTGSESRHVTAHLTAYATYYTGVLGAEL
GELEQDVTIAAGGEDSVKMTFTPREYLDKLVEQAMVKVFVLAHVDTTKQVWAGQDDIRLLSPDLVITAPSEMTVGQQSVVRVEFTNPLDVKLTQ
AVFRIEGPGLQRPKAIYYGTIMPGKQATVTEFMTPMRAGKKTIVASLTSNKLQQVTGEKEVEVKRA

>lancelet_F13A1L
MADKKEYFVKTFTSSKKGAGTDANVFITLTGEKGTTSEVQLKAEGKQDPFERGKMDIFRIWVDKDPGTLTKIRIRHDNKGMFPGWRLDKVSVIE
EGTGVTSVFPCFQCLSKLCGGIDLELPVSKDTKAQQLRINKVDFLREQNAPLHHTDEYDSPLVVVRRGAPFLLKLGFDHGFDRLKDNIVLEFRT
GNTDKPFANKGTEVIVRVGDGPGDHGWEAELSSVKEGEPWAVVSVTPPAQCPVAKWRMSVETISEGLSYETRYDSELVILFNPWCEDDEVYLEG
DDKRGEYVLREFGKVYQSWKVDRNTWMKVFNGKPWNYGQFEPEILEACLGLLDRSGVSVMSRGSPVAVTRVVSQMVNSQDDNGVLEGNWSGDYS
GGVAPWIWNGSVRILQQYHRTKQPVSYGQCWVFSAVTTTVLRCLGIPSRSVTNFSSAHDTDGSLTIDKVVDEYGNLLEDSDSVWNFHVWNEAWM
ARTDLPKGYGGWQALDATPQEKSSGIFCCGPASVNAIKHGEVQYNFDARFIFAEVNADKVHWCQQRDGSCYVAGMEKGTIGRFISTKAVGINDR
EDITENYKFPEGSEEERVAVQRAAQYGSRRSTGQNPYQSATSDVEFSLKDDGSVPINEDADCTITLKNTSSEIRNVTLLYVANIVKYTGVQHRK
LRKEKIEVELKPKSTESVNVAVNPMESMDHMEDKCALKFLFMAHVQETGQTYATERVVDIDFPDLDVKVLGKTDLGQQVAAAITFTNPLDRVLT
NCEFNIEGPGLQKPKTVFFRNIRPGETVKIEERFTPKRAGLKEIVANFSSSELCDITGEAEVQVGLVLHR

>fruit_fly_TGM
LGVLKVDLCLEDNHEEHHTSHFAKEALVVRRGEPFRLKIHFNRDYSPSDAISFIFTVATKPSPGHGTLNALVPHDGDTLEWGAGIESHEGQTLT
VLIKPPSTCPVTEWKLDIDTKLLRSYPLPLPIYVLFNPWCPDDQVYLEDRDQRKEYVMHDTTLIWRGSYNRLRPSVWKIGQFERHVLECSLKVL
GTVRIPPAYRGDPVRVARALSALVNSVDDDGVLLGNWSEDFSGGVAPTKWTGSVEILQQFYKTQKSVKFAQCWNFSGVLTTIARSLGIPSRIIT
CYSSAHDTQASLTVDVFIDANNKKLAETTDSIWNYHVWNELWMQRPDLGVGFDGWQVVDATPQEASDNMYRVGPASVAAVKNGDILRPFDGGFV
FAEVNADKLYWRYNGPQPLKLLRKDTLAIGHLISTKAVLKWEREDITDTYKHAERSEEERSTMLKALKQSRHAFSRYYNDNFNDIEFDMEKDDI
KIGQSFSVVLKVSNKSESRTMATGQISCDAVLYTGVGAVEVKTLGFELELEPKSSDYVRMEVIFEEYYDKLSSQAAFQISAAAKVDTDYDYYAQ
DDFRVRKPDIKFQLGEAAIVQKELDVILRLENPLPIPLHKGVFTVEGPGIEQPLKFKIAEIPVGGTAAATFKYTPPYAGRGTMLAKFTSKELDD
VDGYRHYEIEP
```

Figure S1. Amino acid sequences of transglutaminases used for the phylogenetic analysis. Sequences are provided in FASTA format. The accession numbers of the sequences are provided in Table S1. Proteins marked with an asterisk were excluded from the phylogenetic analysis due to incompleteness of the corresponding gene prediction. Species: Human (*Homo sapiens*), platypus (*Ornithorhynchus anatinus*) chicken (*Gallus gallus*), lizard (*Anolis carolinensis*), frog (*Xenopus tropicalis*), lungfish (*Protopterus annectens*), coelacanth (*Latimeria chalumnae*), zebrafish (*Danio rerio*), shark (*Carcharodon carcharias*), lamprey (*Petromyzon marinus*), tunicate (*Ciona intestinalis*), lancelet (*Branchiostoma floridae*), fruit fly (*Drosophila melanogaster*).

A

```
hTGM1           MMDGPRSDVGRWGGNPLQPPTTPSPEPEPEPDGRSRRGGGRSFWARCCGCCSCRNAADDD
hF13A1          MSETSRTA---FGGRRAVPPNNSNAAEDDLPTVELQGVVPRGVNLQ-------------
hTGM2           MAE---------------------------------------------------------
zTGM10          MASYN-------------------------------------------------------
hTGM4           MMDASK------------------------------------------------------
hEPB42          MGQGEPSQRSTGLAGLYAAPAASPVFIKGSGMD---------------------------
zTGM8           MCSSSSSNTPDQ------------------------------------------------
hTGM3           MA----------------------------------------------------------
hTGM6           MA----------------------------------------------------------
hTGM5           MAQ---------------------------------------------------------
hTGM7           MDQ---------------------------------------------------------
pTGM9           ME----------------------------------------------------------
                *

hTGM1           WGPEPSDSRGRGSSSGTRRPGSRGSDSRRPVSRGSGVNAAGDGTIREGMLVVNGVDLLSS
hF13A1          ----------------------------------------------E-FLNVTSVHLFKE
hTGM2           ----------------------------------------------E--LVLERCDLELE
zTGM10          ----------------------------------------------A---ILSDVDLQCY
hTGM4           ----------------------------------------------E--LQVLHIDFLNQ
hEPB42          ----------------------------------------------A--LGIKSCDFQAA
zTGM8           ----------------------------------------------G-----VRVDLECV
hTGM3           ----------------------------------------------A--LGVQSINWQTA
hTGM6           ----------------------------------------------G--IRVTKVDWQRS
hTGM5           ----------------------------------------------G--LEVALTDLQSS
hTGM7           ----------------------------------------------VATLRLESVDLQSS
pTGM9           ----------------------------------------------E-GIWVAHFDPNCS
                                                                   :

hTGM1           RSDQNRREHHTDEYEYDELIVRRGQPFHMLLLL-S--RTYES-SDRITLELLIGNNPEVG
hF13A1          RWDTNKVDHHTDKYENNKLIVRRGQSFYVQIDF-S--RPYDPRRDLFRVEYVIGRYPQEN
hTGM2           T---NGRDHHTADLCREKLVVRRGQPFWLTLHFEG--RNYEASVDSLTFSVVTGPAPSQE
zTGM10          E---NNHAHRTEEMDVERLLVRRGQPFSVVLQCTE--QIPQLPDHQINLILHLGKNNEVV
hTGM4           D---NAVSHHTWEFQTSSPVFRRGQVFHLRLVL-N--QPLQS-YHQLKLEFSTGPNPSIA
hEPB42          R---NNEEHHTKALSSRRLFVRRGQPFTIILYFRAPVRAFLPALKKVALTAQTGEQPSKI
zTGM8           K---NNTDHHTHEITQERLIIRRGQAFSLKIS--A--ENIQ--QNHISITAETGPGVSEV
hTGM3           F---NRQAHHTDKFSSQELILRRGQNFQVLMIM-N--KGLGS-NERLEFIVSTGPYPSES
hTGM6           R---NGAAHHTQEYPCPELVVRRGQSFSLTLEL-S--RALDC-EEILIFTMETGPRASEA
hTGM5           R---NNVRHHTEEITVDHLLVRRGQAFNLTLYFRN--RSFQPGLDNIIFVVETGPLPDLA
hTGM7           R---NNKEHHTQEMGVKRLTVRRGQPFYLRLSF-S--RPFQSQNDHITFVAETGPKPSEL
pTGM9           L---NCQSHHTDMMSRDRLILRRGQAFDVFLHFQN--RGWDSSKDQITFTVETGPSPCES
                    *   *.*         .**** * : :               . .    *

                             ! intron specific for TGM4
hTGM1           KGTHVIIPVGKGGSG-GWKAQVVKASGQNLNLRVHTSPNAIIGKFQFTVRTQSDAGEFQL
hF13A1          KGTYIPVPIVSELQSGKWGAKIVMREDRSVRLSIQSSPKCIVGKFRMYVAVWTPYGVLRT
hTGM2           AGTKARFPLRDAVEEGDWTATVVDQQDCTLSLQLTTPANAPIGLYRLSLEASTGY--QGS
zTGM10          LK----VSDSEQDPGKWWFSQRNAQGE--VMLTLHSPADAPVGLYSMTVVLLSADGEIQE
hTGM4           KHTLVVLDPRTPSDHYNWQATLQNESGKEVTVAVTSSPNAILGKYQLNVKT----GNHIL
hEPB42          NRTQATFPISSLGDRKWWSAVVEERDAQSWTISVTTPADAVIGHYSLLLQV------SGR
zTGM8           KHTLFSFSTQSSTNP----AQVCSRSESSVSLALVFPSDAPVGRYSVCVK-------HGS
hTGM3           AMTKAVFPLSNGSSG-GWSAVLQASNGNTLTISISSPASAPIGRYTMALQIFSQGG-ISS
hTGM6           LHTKAVFQTSELERGEGWTAAREAQMEKTLTVSLASPPSAVIGRYLLSIRLSSHRK-HSN
hTGM5           LGTRAVFSLARHHSPSPWIAWLETNGATSTEVSLCAPPTAAVGRYLLKIHIDSFQG-SVT
hTGM7           LGTRATFFLTRVQPGNVWSASDFTIDSNSLQVSLFTPANAVIGHYTLKIEISQGQG-HSV
pTGM9           SGTRTTFFSLSETAPLHCWGAVCKASRHRSIQVSLLIPANACIGLYSLQAQVPSGQG-PGP
                      .            :           : :  .. . :* : .

hTGM1           PFDPRNEIYILFNPWCPEDIVYVDHEDWRQEYVLNESGRIYYGTEAQIGERTWNYGQFDH
hF13A1          SRNPETDTYILFNPWCEDDAVYLDNEKEREEYVLNDIGVIFYGEVNDIKTRSWSYGQFED
hTGM2           SFVL-GHFILLFNAWCPADAVYLDSEEERQEYVLTQQGFIYQGSAKFIKNIPWNFGQFED
zTGM10          QTSP-QTFYLLFNPWCKDDCVYLPSEEMLQEYILNENGILYQGAWDDITTVPWNFGQFEK
hTGM4           KSEE-NILYLLFNPWCKEDMVFMPDEDERKEYILNDTGCHYVGAARSIKCKPWNFGQFEK
hEPB42          KQLLLGQFTLLFNPWNREDAVFLKNEAQRMEYLLNQNGLIYLGTADCIQAESWDFGQFEG
zTGM8           SSCM-NTLTLLFNPWCKDDHVYLPSEAERQEYIMSEQGVLYKGVDEYITSMNWDYGQFEE
hTGM3           V-KL-GTFILLFNPWLNVDSVFMGNHAEREEYVQEDAGIIFVGSTNRIGMIGWNFGQFEE
hTGM6           R-RL-GEFVLLFNPWCAEDDVFLASEEERQEYVLSDSGIIFRGVEKHIRAQGWNYGQFEE
hTGM5           AYQL-GEFILLFNPWCPEDAVYLDSEPQRQEYVMNDYGFIYQGSKNWIRPCPWNYGQFED
hTGM7           TYPL-GTFILLFNPWSPEDDVYLPSEILLQEYIMRDYGFVYKGHERFITSWPWNYGQFEE
pTGM9           R-TL-GEFIVLFNPWCPDDLVYLENPSHREEYILNEHGMVFRGLHKYIVSHPWHFGQFEE
                         :***.*   * *::       **:  : *  : *    *    * :***:
```

```
hTGM1          GVLDACLYILDRR-------GMPYGGRGDPVNVSRVISAMVNSLDDNGVLIGNWSGDYSR
hF13A1         GILDTCLYVMDRA-------QMDLSGRGNPIKVSRVGSAMVNAKDDEGVLVGSWDNIYAY
hTGM2          GILDICLILLDVNPKFLKNAGRDCSRRSSPVYVGRVVSGMVNCNDDQGVLLGRWDNNYGD
zTGM10         DVVDICFDVLDNSPAALKNSEMDIFNRASPVYVSRTITAMVNANDDRGVVSGRWDGEYSD
hTGM4          NVLDCCISLLTES-------SLKPTDRRDPVLVCRAMCAMMSFEKGQGVLIGNWTGDYEG
hEPB42         DVIDLSLRLLSKD--------KQVEKWSQPVHVARVLGALLHFLKEQRVLPTPQTQATQE
zTGM8          DIVDICLKLLDLNPKCLKDPQEDYSARCNPVYVSRVVSAMINCNDDQGVLAGQWGDSYTG
hTGM3          DILSICLSILDRSLNFRRDAATDVASRNDPKYVGRVLSAMINSNDDNGVLAGNWSGTYTG
hTGM6          DILNICLSILDRSPGHQNNPATDVSCRHNPIYVTRVISAMVNSNNDRGVVQGQWQGKYGG
hTGM5          KIIDICLKLLDKSLHFQTDPATDCALRGSPVYVSRVVCAMINSNDDNGVLNGNWSENYTD
hTGM7          DIIDICFEILNKSLYHLKNPAKDCSQRNDVVYVCRVVSAMINSNDDNGVLQGNWGEDYSK
pTGM9          NMVDMCLRVLDMSGNFQRDPSLDCSYRNDPVYVSRVVNSMLCSHSSNSLMKLPRNNDSAQ
                ::. .: ::                  . .    * *.  .::    .    ::    .

hTGM1          GTNPSAWVGSVEILLSYLRTGYS-VPYGQCWVFAGVTTTVLRCLGLATRTVTNFNSAHDT
hF13A1         GVPPSAWTGSVDILLEYRSSE-NPVRYGQCWVFAGVFNTFLRCLGIPARIVTNYFSAHDN
hTGM2          GVSPMSWIGSVDILRRWKNHGCQRVKYGQCWVFAAVACTVLRCLGIPTRVVTNYNSAHDQ
zTGM10         GVAPTRWTGSVPILRRWSEDGGQKVRYGQCWVFTGVACTVLRCLGIPTRCITNYSSAHDT
hTGM4          GTAPYKWTGSAPILQQYYNTK-QAVCFGQCWVFAGILTTVLRALGIPARSVTGFDSAHDT
hEPB42         GALLNKRRGSVPILRQWLTGRGRPVYDGQAWVLAAVACTVLRCLGIPARVVTTFASAQGT
zTGM8          GVPPSRWSSSVEILRRWVKYNCSPVKYGQCWVFAAVMCTVLRCLGIPCRVVTNYQSAHDT
hTGM3          GRDPRSWNGSVEILKNWKKSGFSPVRYGQCWVFAGTLNTALRSLGIPSRVITNFNSAHDT
hTGM6          GTSPLHWRGSVAILQKWLKGRYKPVKYGQCWVFAGVLCTVLRCLGIATRVVSNFNSAHDT
hTGM5          GANPAEWTGSVAILKQWNATGCQPVRYGQCWVFAAVMCTVMRCLGIPTRVITNFDSGHDT
hTGM7          GVSPLEWKGSVAILQQWSARGGQPVKYGQCWVFASVMCTVMRCLGVPTRVVSNFRSAHNV
pTGM9          GVNPLAWNGSVPILSQWYGSGCRPVRFGQCSTLAAVMCTVMRCLGIPSRVVTNFYSTQNA
               *     . .*. **  :       *  **. .::.   * :*.**:. * :: : * :.

hTGM1          DTSLTMDIYFDENMKPLEHLNHDSVWNFHVWNDCWMKRPDLPSG--FDGWQVVDATPQET
hF13A1         DANLQMDIFLEEDGNVNSKLTKDSVWNYHCWNEAWMTRPDLPVG--FGGWQAVDSTPQEN
hTGM2          NSNLLIEYFRNEFGEIQGDKS-EMIWNFHCWVESWMTRPDLQPG--YEGWQALDPTPQEK
zTGM10         DANIAVDYLVNDQLESVSEGRKDTIWNYHCWVESWMKREDLPEG--YDGWQVLDPTPQER
hTGM4          ERNLTVDTYVNENGEKITSMTHDSVWNFHVWTDAWMKRPDLPKG--YDGWQAVDATPQER
hEPB42         GGRLLIDEYYNEEGLQNGEGQRGRIWIFQTSTECWMTRPALPQG--YDGWQILHPSAPNG
zTGM8          DRNLVVDEYFSDYG-VRPKNSQDSVWNFHVWVEAWMRRPDLTEDTLYDGWQVLDPTPQEK
hTGM3          DRNLSVDVYYDPMGNPLDKGS-DSVWNFHVWNEGWFVRSDLGPS--YGGWQVLDATPQER
hTGM6          DQNLSVDKYVDSFGRTLEDLTEDSMWNFHVWNESWFARQDLGPS--YNGWQVLDATPQEE
hTGM5          DGNLIIDEYYDNTGRILGNKKKDTIWNFHVWNECWMARKDLPPA--YGGWQVLDATPQEM
hTGM7          DRNLTIDTYYDRNAEMLSTQKRDKIWNFHVWNECWMIRKDLPPG--YNGWQVLDPTPQQT
pTGM9          SEAFIIDEYFDSTGRSLC--GKEHIWRHHCWNESWMVRKDLNES--CGEWQYLDPTPMET
                  : ::   .             :* .:   : *: *  *         ** : .:. :

                                           ! intron specific for TGM1 and F13A1
hTGM1          SSGIFCCGPCSVESIKNGLVYMKYDTPFIFAEVNSDKVYWQRQDDGSFK-IVYV---EEK
hF13A1         SDGMYRCGPASVQAIKHGHVCFQFDAPFVFAEVNSDLIYITAKKDGTHV-VENV---DAT
hTGM2          SEGTYCCGPVPVRAIKEGDLSTKYDAPFVFAEVNADVVDWIQQDDGSVH-KSIN---RSL
zTGM10         SDGIFCCGPCPVRAVKEGEVGLKYDTPFVFSEVNADLIVWIVHPDGERS-EVSQ---NSK
hTGM4          SQGVFCCGPSPLTAIRKGDIFIVYDTRFVFSEVNGDRLIWLVKMVNGQE-ELHVISMETT
hEPB42         GGVLGSCDLVPVRAVKEGTLGLTPAVSDLFAAINASCVVWKCCEDGTLE-LTDS---NTK
zTGM8          SSGTYCCGPAPVMAILEGHTEVKYDVPFVFAEVNADRVCWLLMTDGSRK-KIMS---DSR
hTGM3          SQGVFQCGPASVIGVREGDVQLNFDMPFIFAEVNADRITWLYDNTTGKQWKNSV---NSH
hTGM6          SEGVFRCGPASVTAIREGDVHLAHDGPFVFAEVNADYITWLWHEDESRE-RVYS---NTK
hTGM5          SNGVYCCGPASVRAIKEGEVDLNYDTPFVFSMVNADCMSWLVQ--GGKEQKLHQ---DTS
hTGM7          SSGLFCCGPASVKAIREGDVHLAYDTPFVYAEVNADEVIWLLGDGQAQE-ILAH---NTS
pTGM9          SGGLVCCGPTCVKNIREGDLDQDYDGAYVFSRLNAGRASWLRQASEGKA-KVHC---DAR
               .     *.   :  :  *           ::: :*..

hTGM1          AIGTLIVTKAISSNMREDITYLYKHPEGSDAERKAVETAAAHG-----------------
hF13A1         HIGKLIVTKQIGGDGMMDITDTYKFQEGQEEERLALETALMYG-----------------
hTGM2          IVGLKISTKSVGRDEREDITHTYKYPEGSSEEREAFTRANHLN-----------------
zTGM10         IIGRKISTKSVYGDFREDITANYKYPEGSMKEREVYKKAGRQV-----------------
hTGM4          SIGKNISTKAVGQDRRRDITYEYKYPEGSSEERQVMDHAFLLL-----------------
hEPB42         YVGNNISTKGVGSDRCEDITQNYKYPEGSLQEKEVLERVEKEK-----------------
zTGM8          SVGQNLSTKAVGSSSRLDITDLYKHGEGSAQERAVYTEAVHRL-----------------
hTGM3          TIGRYISTKAVGSNARMDVTDKYKYPEGSDQERQVFQKALGKL----------------K
hTGM6          KIGRCISTKAVGSDSRVDITDLYKYPEGSRKERQVYSKAVNRL-----FGVEASGRRIWI
hTGM5          SVGNFISTKSIQSDERDDITENYKYEEGSLQERQVFLKALQKLKARSFHGSQRGAELQPS
hTGM7          SIGKEISTKMVGSDQRQSITSSYKYPEGSPEERAVFMKASRKM-----------------
pTGM9          LFGQSISTKGVGTEEREDITHNYKHQPDSIRGREVFYKAYRRI--------------HPK
                .*  : ** :  .   .:*  **.  ..    . .   .
```

B

	hEPB42	hTGM4	hTGM2	zTGM10	pTGM9	zTGM8	hTGM3	hTGM6	hTGM5	hTGM7	hF13A1	hTGM1
1: hEPB42	100.00	28.23	33.73	30.95	29.20	30.85	30.71	31.17	33.38	32.70	24.89	26.27
2: hTGM4	28.23	100.00	32.98	32.93	28.76	36.06	35.49	36.04	34.67	36.98	31.70	37.74
3: hTGM2	33.73	32.98	100.00	42.77	37.83	41.59	37.96	41.74	42.46	41.52	37.54	36.76
4: zTGM10	30.95	32.93	42.77	100.00	33.09	40.39	35.17	38.28	38.73	38.21	34.37	36.18
5: pTGM9	29.20	28.76	37.83	33.09	100.00	36.43	37.10	36.12	43.30	38.43	30.01	31.39
6: zTGM8	30.85	36.06	41.59	40.39	36.43	100.00	39.31	43.91	42.96	41.67	36.62	35.87
7: hTGM3	30.71	35.49	37.96	35.17	37.10	39.31	100.00	50.87	45.44	40.64	34.07	36.40
8: hTGM6	31.17	36.04	41.74	38.28	36.12	43.91	50.87	100.00	47.02	45.66	35.10	39.41
9: hTGM5	33.38	34.67	42.46	38.73	43.30	42.96	45.44	47.02	100.00	49.79	35.34	37.91
10: hTGM7	32.70	36.98	41.52	38.21	38.43	41.67	40.64	45.66	49.79	100.00	31.10	37.28
11: hF13A1	24.89	31.70	37.54	34.37	30.01	36.62	34.07	35.10	35.34	31.10	100.00	41.27
12: hTGM1	26.27	37.74	36.76	36.18	31.39	35.87	36.40	39.41	37.91	37.28	41.27	100.00

C

>hTGM1 NP_000350.1 protein-glutamine gamma-glutamyltransferase K [Homo sapiens]
MMDGPRSDVGRWGGNPLQPPTTPSPEPEPEPDGRSRRGGGRSFWARCCGCCSCRNAADDDWGPEPSDSRGRGSSSGTRRPGSRGSDSRRPVSRGSGVNAAGDG
TIREGMLVVNGVDLLSSRSDQNRREHHTDEYEYDELIVRRGQPFHMLLLLSRTYESSDRITLELLIGNNPEVGKGTHVIIPVGKGGSGGWKAQVVKASGQNLN
LRVHTSPNAIIGKFQFTVRTQSDAGEFQLPFDPRNEIYILFNPWCPEDIVYVDHEDWRQEYVLNESGRIYYGTEAQIGERTWNYGQFDHGVLDACLYILDRRG
MPYGGRGDPVNVSRVISAMVNSLDDNGVLIGNWSGDYSRGTNPSAWVGSVEILLSYLRTGYSVPYGQCWVFAGVTTTVLRCLGLATRTVTNFNSAHDTDTSLT
MDIYFDENMKPLEHLNHDSVWNFHVWNDCWMKRPDLPSGFDGWQVVDATPQETSSGIFCCGPCSVESIKNGLVYMKYDTPFIFAEVNSDKVYWQRQDDGSFKI
VYVEEKAIGTLIVTKAISSNMREDITYLYKHPEGSDAERKAVETAAAHGSKPNVYANRGSAEDVAMQVEAQDAVMGQDLMVSVMLINHSSSRRTVKLHLYLSV
TFYTGVSGTIFKETKKEVELAPGASDRVTMPVAYKEYRPHLVDQGAMLLNVSGHVKESGQVLAKQHTFRLRTPDLSLTLLGAAVVGQECEVQIVFKNPLPVTL
TNVVFRLEGSGLQRPKILNVGDIGGNETVTLRQSFVPVRPGPRQLIASLDSPQLSQVHGVIQVDVAPAPGDGGFFSDAGGDSHLGETIPMASRGGA

>hTGM2 NP_001310245.1 protein-glutamine gamma-glutamyltransferase 2 isoform a [Homo sapiens]
MAEELVLERCDLELETNGRDHHTADLCREKLVVRRGQPFWLTLHFEGRNYEASVDSLTFSVVTGPAPSQEAGTKARFPLRDAVEEGDWTATVVDQQDCTLSLQ
LTTPANAPIGLYRLSLEASTGYQGSSFVLGHFILLFNAWCPADAVYLDSEEERQEYVLTQQGFIYQGSAKFIKNIPWNFGQFEDGILDICLILLDVNPKFLKN
AGRDCSRRSSPVYVGRVVSGMVNCNDDQGVLLGRWDNNYGDGVSPMSWIGSVDILRRWKNHGCQRVKYGQCWVFAAVACTVLRCLGIPTRVVTNYNSAHDQNS
NLLIEYFRNEFGEIQGDKSEMIWNFHCWVESWMTRPDLQPGYEGWQALDPTPQEKSEGTYCCGPVPVRAIKEGDLSTKYDAPFVFAEVNADVVDWIQQDDGSV
HKSINRSLIVGLKISTKSVGRDEREDITHTYKYPEGSSEEREAFTRANHLNKLAEKEETGMAMRIRVGQSMNMGSDFDVFAHITNNTAEEYVCRLLLCARTVS
YNGILGPECGTKYLLNLNLEPFSEKSVPLCILYEKYRDCLTESNLIKVRALLVEPVINSYLLAERDLYLENPEIKIRILGEPKQKRKLVAEVSLQNPLPVALE
GCTFTVEGAGLTEEQKTVEIPDPVEAGEEVKVRMDLLPLHMGLHKLVVNFESDKLKAVKGFRNVIIGPA

>hTGM3 NP_003236.3 protein-glutamine gamma-glutamyltransferase E [Homo sapiens]
MAALGVQSINWQTAFNRQAHHTDKFSSQELILRRGQNFQVLMIMNKGLGSNERLEFIVSTGPYPSESAMTKAVFPLSNGSSGGWSAVLQASNGNTLTISISSP
ASAPIGRYTMALQIFSQGGISSVKLGTFILLFNPWLNVDSVFMGNHAEREEYVQEDAGIIFVGSTNRIGMIGWNFGQFEEDILSICLSILDRSLNFRRDAATD
VASRNDPKYVGRVLSAMINSNDDNGVLAGNWSGTYTGGRDPRSWNGSVEILKNWKKSGFSPVRYGQCWVFAGTLNTALRSLGIPSRVITNFNSAHDTDRNLSV
DVYYDPMGNPLDKGSDSVWNFHVWNEGWFVRSDLGPSYGGWQVLDATPQERSQGVFQCGPASVIGVREGDVQLNFDMPFIFAEVNADRITWLYDNTTGKQWKN
SVNSHTIGRYISTKAVGSNARMDVTDKYKYPEGSDQERQVFQKALGKLKPNTPFAATSSMGLETEEQEPSIIGKLKVAGMLAVGKEVNLVLLLKNLSRDTKTV
TVNMTAWTIIYNGTLVHEVWKDSATMSLDPEEEAEHPIKISYAQYEKYLKSDNMIRITAVCKVPDESEVVVERDIILDNPTLTLEVLNEARVRKPVNVQMLFS
NPLDEPVRDCVLMVEGSGLLLGNLKIDVPTLGPKEGSRVRFDILPSRSGTKQLLADFSCNKFPAIKAMLSIDVAE

>hTGM4 NP_003232.2 protein-glutamine gamma-glutamyltransferase 4 [Homo sapiens]
MMDASKELQVLHIDFLNQDNAVSHHTWEFQTSSPVFRRGQVFHLRLVLNQPLQSYHQLKLEFSTGPNPSIAKHTLVVLDPRTPSDHYNWQATLQNESGKEVTV
AVTSSPNAILGKYQLNVKTGNHILKSEENILYLLFNPWCKEDMVFMPDEDERKEYILNDTGCHYVGAARSIKCKPWNFGQFEKNVLDCCISLLTESSLKPTDR
RDPVLVCRAMCAMMSFEKGQGVLIGNWTGDYEGGTAPYKWTGSAPILQQYYNTKQAVCFGQCWVFAGILTTVLRALGIPARSVTGFDSAHDTERNLTVDTYVN
ENGEKITSMTHDSVWNFHVWTDAWMKRPDLPKGYDGWQAVDATPQERSQGVFCCGPSPLTAIRKGDIFIVYDTRFVFSEVNGDRLIWLVKMVNGQEELHVISM
ETTSIGKNISTKAVGQDRRRDITYEYKYPEGSSEERQVMDHAFLLLSSEREHRRPVKENFLHMSVQSDDVLLGNSVNFTVILKRKTAALQNVNILGSFELQLY
TGKKMAKLCDLNKTSQIQGQVSEVTLTLDSKTYINSLAILDDEPVIRGFIIAEIVESKEIMASEVFTSFQYPEFSIELPNTGRIGQLLVCNCIFKNTLAIPLT
DVKFSLESLGISSLQTSDHGTVQPGETIQSQIKCTPIKTGPKKFIVKLSSKQVKEINAQKIVLITK

>hTGM5 NP_963925.2 protein-glutamine gamma-glutamyltransferase 5 isoform 1 [Homo sapiens]
MAQGLEVALTDLQSSRNNVRHHTEEITVDHLLVRRGQAFNLTLYFRNRSFQPGLDNIIFVVETGPLPDLALGTRAVFSLARHHSPSPWIAWLETNGATSTEVS
LCAPPTAAVGRYLLKIHIDSFQGSVTAYQLGEFILLFNPWCPEDAVYLDSEPQRQEYVMNDYGFIYQGSKNWIRPCPWNYGQFEDKIIDICLKLLDKSLHFQT
DPATDCALRGSPVYVSRVVCAMINSNDDNGVLNGNWSENYTDGANPAEWTGSVAILKQWNATGCQPVRYGQCWVFAAVMCTVMRCLGIPTRVITNFDSGHDTD
GNLIIDEYYDNTGRILGNKKKDTIWNFHVWNECWMARKDLPPAYGGWQVLDATPQEMSNGVYCCGPASVRAIKEGEVDLNYDTPFVFSMVNADCMSWLVQGGK
EQKLHQDTSSVGNFISTKSIQSDERDDITENYKYEEGSLQERQVFLKALQKLKARSFHGSQRGAELQPSRPTSLSQDSPRSLHTPSLRPSDVVQVSLKFKLLD
PPNMGQDICFVLLALNMSSQFKDLKVNLSAQSLLHDGSPLSPFWQDTAFITLSPKEAKTYPCKISYSQYSQYLSTDKLIRISALGEEKSSPEKILVNKIITLS
YPSITINVLGAAVVNQPLSIQVIFSNPLSEQVEDCVLTVEGSGLFKKQQKVFLGVLKPQHQASIILETVPFKSGQRQIQANMRSNKFKDIKGYRNVYVDFAL

>hTGM6 NP_945345.2 protein-glutamine gamma-glutamyltransferase 6 isoform 1 [Homo sapiens]
MAGIRVTKVDWQRSRNGAAHHTQEYPCPELVVRRGQSFSLTLELSRALDCEEILIFTMETGPRASEALHTKAVFQTSELERGEGWTAAREAQMEKTLTVSLAS
PPSAVIGRYLLSIRLSSHRKHSNRRLGEFVLLFNPWCAEDDVFLASEEERQEYVLSDSGIIFRGVEKHIRAQGWNYGQFEEDILNICLSILDRSPGHQNNPAT
DVSCRHNPIYVTRVISAMVNSNNDRGVVQGQWQGKYGGGTSPLHWRGSVAILQKWLKGRYKPVKYGQCWVFAGVLCTVLRCLGIATRVVSNFNSAHDTDQNLS
VDKYVDSFGRTLEDLTEDSMWNFHVWNESWFARQDLGPSYNGWQVLDATPQEESEGVFRCGPASVTAIREGDVHLAHDGPFVFAEVNADYITWLWHEDESRER
VYSNTKKIGRCISTKAVGSDSRVDITDLYKYPEGSRKERQVYSKAVNRLFGVEASGRRIWIRRAGGRCLWRDDLLEPATKPSIAGKFKVLEPPMLGHDLRLAL
CLANLTSRAQRVRVNLSGATILYTRKPVAEILHESHAVRLGPQEEKRIPITISYSKYKEDLTEDKKILLAAMCLVTKGEKLLVEKDITLEDFITIKVLGPAMV
GVAVTVEVTVVNPLIERVKDCALMVEGSGLLQEQLSIDVPTLEPQERASVQFDITPSKSGPRQLQVDLVSPHFPDIKGFVIVHVATAK

>hTGM7 NP_443187.1 protein-glutamine gamma-glutamyltransferase Z [Homo sapiens]
MDQVATLRLESVDLQSSRNNKEHHTQEMGVKRLTVRRGQPFYLRLSFSRPFQSQNDHITFVAETGPKPSELLGTRATFFLTRVQPGNVWSASDFTIDSNSLQV
SLFTPANAVIGHYTLKIEISQGQGHSVTYPLGTFILLFNPWSPEDDVYLPSEILLQEYIMRDYGFVYKGHERFITSWPWNYGQFEEDIIDICFEILNKSLYHL
KNPAKDCSQRNDVVYVCRVVSAMINSNDDNGVLQGNWGEDYSKGVSPLEWKGSVAILQQWSARGGQPVKYGQCWVFASVMCTVMRCLGVPTRVVSNFRSAHNV

```
DRNLTIDTYYDRNAEMLSTQKRDKIWNFHVWNECWMIRKDLPPGYNGWQVLDPTPQQTSSGLFCCGPASVKAIREGDVHLAYDTPFVYAEVNADEVIWLLGDG
QAQEILAHNTSSIGKEISTKMVGSDQRQSITSSYKYPEGSPEERAVFMKASRKMLGPQRASLPFLDLLESGGLRDQPAQLQLHLARIPEWGQDLQLLLRIQRV
PDSTHPRGPIGLVVRFCAQALLHGGGTQKPFWRHTVRMNLDFGKETQWPLLLPYSNYRNKLTDEKLIRVSGIAEVEETGRSMLVLKDICLEPPHLSIEVSERA
EVGKALRVHVTLTNTLMVALSSCTMVLEGSGLINGQIAKDLGTLVAGHTLQIQLDLYPTKAGPRQLQVLISSNEVKEIKGYKDIFVTVAGAP

>zTGM8 XP_009301053.1 protein-glutamine gamma-glutamyltransferase 2-like isoform X2 [Danio rerio]
MCSSSSSNTPDQGVRVDLECVKNNTDHHTHEITQERLIIRRGQAFSLKISAENIQQNHISITAETGPGVSEVKHTLFSFSTQSSTNPAQVCSRSESSVSLALV
FPSDAPVGRYSVCVKHGSSSCMNTLTLLFNPWCKDDHVYLPSEAERQEYIMSEQGVLYKGVDEYITSMNWDYGQFEEDIVDICLKLLDLNPKCLKDPQEDYSA
RCNPVYYVSRVVSAMINCNDDQGVLAGQWGDSYTGGVPPSRWSSSVEILRRWVKYNCSPVKYGQCWVFAAVMCTVLRCLGIPCRVVTNYQSAHDTDRNLVVDEY
FSDYGVRPKNSQDSVWNFHVWVEAWMRRPDLTEDTLYDGWQVLDPTPQEKSSGTYCCGPAPVMAILEGHTEVKYDVPFVFAEVNADRVCWLLMTDGSRKKIMS
DSRSVGQNLSTKAVGSSSRLDITDLYKHGEGSAQERAVYTEAVHRLTDLQIPESPPVSVQMRLSLDVPPLNGADVPLKLFLKGSRAQCVCVNISAQVMRYTGA
PAAAVWSHSTDVQLQDTEEKALSFTLPYSAYGHRMLENNCIKVSAIAREKNNPKDTYLTEKNIILHTPNLSITVSGSPLLDSEMTATVQFENPLSSTLQNCSI
SLSGSGLLKSTEKSSTVQLGPGQRIRLQVSFTPYRLGLKKLMATFNSATFKDVQASADVDVRSA

>pTGM9 corrected from XP_028921818.2 TGM5-like [Ornithorhynchus anatinus]
MEEGIWVAHFDPNCSLNCQSHHTDMMSRDRLILRRGQAFDVFLHFQNRGWDSSKDQITFTVETGPSPCESSGTRTTFSLSETAPLHCWGAVCKASRHRSIQVS
LLIPANACIGLYSLQAQVPSGQGPGPRTLGEFIVLFNPWCPDDLVYLENPSHREEYILNEHGMVFRGLHKYIVSHPWHFGQFEENMVDMCLRVLDMSGNFQRD
PSLDCSYRNDPVYVSRVVNSMLCSHSSNSLMKLPRNNDSAQGVNPLAWNGSVPILSQWYGSGCRPVRFGQCSTLAAVMCTVMRCLGIPSRVVTNFYSTQNASE
AFIIDEYFDSTGRSLCGKEHIWRHHCWNESWMVRKDLNESCGEWQYLDPTPMETSGGLVCCGPTCVKNIREGDLDQDYDGAYVFSRLNAGRASWLRQASEGKA
KVHCDARLFGQSISTKGVGTEEREDITHNYKHQPDSIRGREVFYKAYRRIHPKFLSASNCQIEKELQALRNPGLDEADTVMKFKLGNSPVYGEDINLFLHLAN
LSHESRDLRLKLSAEGLTYAGCFMEPFWTDDLIISLKPKAEKKVPLQIMYSQYGRHLGDHNLLRVVAVSEPGCKGEIMLVDRDILLLKPPVEIKLRGSPRLNV
RCTAEIIFTNPLPESLRNCKLTLEGSNLMQKPVTIELGTLAPRHQTQTLVDLIPFRPGLHRLLANFDCHRFSYCKGYANVRVDNSLISLVQGPR

>zTGM10 XP_687398.2 protein-glutamine gamma-glutamyltransferase 2 [Danio rerio]
MASYNAILSDVDLQCYENNHAHRTEEMDVERLLVRRGQPFSVVLQCTEQIPQLPDHQINLILHLGKNNEVVLKVSDSEQDPGKWWFSQRNAQGEVMLTLHSPA
DAPVGLYSMTVVLLSADGEIQEQTSPQTFYLLFNPWCKDDCVYLPSEEMLQEYILNENGILYQGAWDDITTVPWNFGQFEKDVVDICFDVLDNSPAALKNSEM
DIFNRASPVYVSRTITAMVNANDDRGVVSGRWDGEYSDGVAPTRWTGSVPILRRWSEDGGQKVRYGQCWVFTGVACTVLRCLGIPTRCITNYSSAHDTDANIA
VDYLVNDQLESVSEGRKDTIWNYHCWVESWMKREDLPEGYDGWQVLDPTPQERSDGIFCCGPCPVRAVKEGEVGLKYDTPFVFSEVNADLIVWIVHPDGERSE
VSQNSKIIGRKISTKSVYGDFREDITANYKYPEGSMKEREVYKKAGRQVGQKKDGPGQLELFIKHAPAIHGTDFDVIIEVYNAGREDTDAKLTVTSNAITYNS
IHRGECQRKTTSLTVPAYKAHKEVLRLQYDHYGACVSEHHMIRVTALLQPNDQDNLILQETNIPLKMPALHVKIIGNAIVSRKLTAHISFTNPLPINLQGGVF
TVEGAGLTEAREIKTHGKIESGQTVTVKFSFKPTRAGLRKLLVDFDSDRLRDVKGEASVIVRTRMRHVNAVPEI

>hF13A1 NP_000120.2 coagulation factor XIII A chain [Homo sapiens]
MSETSRTAFGGRRAVPPNNSNAAEDDLPTVELQGVVPRGVNLQEFLNVTSVHLFKERWDTNKVDHHTDKYENNKLIVRRGQSFYVQIDFSRPYDPRRDLFRVE
YVIGRYPQENKGTYIPVPIVSELQSGKWGAKIVMREDRSVRLSIQSSPKCIVGKFRMYVAVWTPYGVLRTSRNPETDTYILFNPWCEDDAVYLDNEKEREEYV
LNDIGVIFYGEVNDIKTRSWSYGQFEDGILDTCLYVMDRAQMDLSGRGNPIKVSRVGSAMVNAKDDEGVLVGSWDNIYAYGVPPSAWTGSVDILLEYRSSENP
VRYGQCWVFAGVFNTFLRCLGIPARIVTNYFSAHDNDANLQMDIFLEEDGNVNSKLTKDSVWNYHCWNEAWMTRPDLPVGFGGWQAVDSTPQENSDGMYRCGP
ASVQAIKHGHVCFQFDAPFVFAEVNSDLIYITAKKDGTHVVENVDATHIGKLIVTKQIGGDGMMDITDTYKFQEGQEEERLALETALMYGAKKPLNTEGVMKS
RSNVDMDFEVENAVLGKDFKLSITFRNNSHNRYTITAYLSANITFYTGVPKAEFKKETFDVTLEPLSFKKEAVLIQAGEYMGQLLEQASLHFFVTARINETRD
VLAKQKSTVLTIPEIIIKVRGTQVVGSDMTVTVEFTNPLKETLRNVWVHLDGPGVTRPMKKMFREIRPNSTVQWEEVCRPWVSGHRKLIASMSSDSLRHVYGE
LDVQIQRRPSM

>hEPB42 NP_000110.2 protein 4.2 isoform 1 [Homo sapiens]
MGQGEPSQRSTGLAGLYAAPAASPVFIKGSGMDALGIKSCDFQAARNNEEHHTKALSSRRLFVRRGQPFTIILYFRAPVRAFLPALKKVALTAQTGEQPSKIN
RTQATFPISSLGDRKWWSAVVEERDAQSWTISVTTPADAVIGHYSLLLQVSGRKQLLLGQFTLLFNPWNREDAVFLKNEAQRMEYLLNQNGLIYLGTADCIQA
ESWDFGQFEGDVIDLSLRLLSKDKQVEKWSQPVHVARVLGALLHFLKEQRVLPTPQTQATQEGALLNKRRGSVPILRQWLTGRGRPVYDGQAWVLAAVACTVL
RCLGIPARVVTTFASAQGTGGRLLIDEYYNEEGLQNGEGQRGRIWIFQTSTECWMTRPALPQGYDGWQILHPSAPNGGGVLGSCDLVPVRAVKEGTLGLTPAV
SDLFAAINASCVVWKCCEDGTLELTDSNTKYVGNNISTKGVGSDRCEDITQNYKYPEGSLQEKEVLERVEKEKMEREKDNGIRPPSLETASPLYLLLKAPSSL
PLRGDAQISVTLVNHSEQEKAVQLAIGVQAVHYNGVLAAKLWRKKLHLTLSANLEKIITIGLFFSNFERNPPENTFLRLTAMATHSESNLSCFAQEDIAICRP
HLAIKMPEKAEQYQPLTASVSLQNSLDAPMEDCVISILGRGLIHRERSYRFRSVWPENTMCAKFQFTPTHVGLQRLTVEVDCNMFQNLTNYKSVTVVAPELSA
```

Supplementary Figure S2. Amino acid sequence alignment of transglutaminases and comparison of exon borders. (**A**) The amino acid sequences of TGMs were aligned with MUSCLE, followed by manual adjustment. Green, yellow and blue shadings indicate amino acid residues encoded by triplets at exon borders in splicing phases 0, 1 and 2, respectively. Exclamation marks (!) above the sequences highlight residues corresponding to the borders of introns that are not shared by all TGM genes. The cysteine cluster involved in lipidation and membrane anchorage of TGM1 is underlined. The symbols "*", ":" and "." below the alignment indicate perfect conservation of a residue, conservation of amino acids with highly similar chemical properties and partial conservation of residues with similar properties, respectively. Species abbreviations: h, human; p, platypus; z, zebrafish. (**B**) Percent identity matrix. The matrix was created by Clustal2.1. (**C**) Amino acid sequences used for the alignment. Note that the amino acid sequence of platypus TGM9 is predicted differently (indicated by red fonts) from the prediction in GenBank.

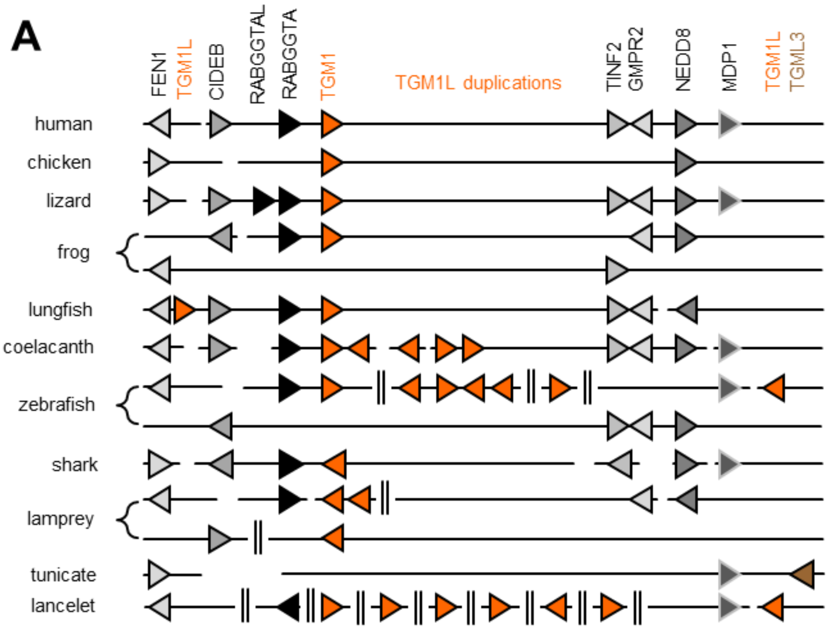

B

Cysteine cluster at N-terminus:

Human TGM1	R--CCGCCS
Platypus TGM1	R--CCGCCS
Lizard TGM1	K--CCSCCS
Chicken TGM1	CCGCCGCCR
Clawed frog TGM1	LCPCC-CTE
Lungfish TGM1	W--CCPCLR
Coelacanth TGM1	R--CCPCLG
Zebrafish TGM1	QC-CCSCRK
Shark TGM1	S--CCPCGR
Lamprey TGM1	L--CCCCLP

Figure S3. Synteny analysis of *TGM1* loci in vertebrates. (**A**) Triangles represent genes which are shown in direction of transcription. *TGM1* is flanked by *RABGGTA* and *TINF2* or *NEDD8* in all investigated vertebrates. A lungfish-specific *TGM1* duplicate is flanked by *FEN1* and *CIDEB*. In coelacanth and zebrafish several species-specific *TGM1* duplicates are located next to the pan-vertebrate *TGM1* ortholog. The lancelet has 7 *TGM1*-like copies. One TGM gene (XP_009861590.1, here called *TGML3*) of the vase tunicate is located next to *MDP1*. *MDP1* is conserved in all chordates in close proximity to the vertebrate *TGM1* locus. Short vertical double lines indicate gaps in the depiction of genes on the same chromosome. (**B**) Amino acid sequence alignment of the cysteine cluster close to the amino-terminus of TGM1 of vertebrates. Species: Human (*Homo sapiens*), chicken (*Gallus gallus*), lizard (*Anolis carolinensis*), frog (*Xenopus tropicalis*), lungfish (*Protopterus annectens*), coelacanth (*Latimeria chalumnae*), zebrafish (*Danio rerio*), shark (*Carcharodon carcharias*), lamprey (*Petromyzon marinus*), tunicate (*Ciona intestinalis*), lancelet (*Branchiostoma floridae*).

```
                    1                                                                                                  100
human_TGM1          ---------------------------------------------------------------MMDG-PRSDVGRWGGNPLQPPTTPSPEPEPEPDGRSR
chicken_TGM1        ---------------------------------------------------------------MPDADPRLDAGRWAAASFRLRGAPSSDPPPRSRGSFW
frog_TGM1           -------------------------------------------------------MARCEERKKSFWERLCPCCCTERSQYEPDNDMRPVNRPDGGSRDN
coelacanth_TGM1     --------------------------------------------------------------------------MPAIARETSI--YNDVGRRAPVPLAA
coelacanth_TGM1L1   ----------------------------------------------------------------------------------------------------
coelacanth_TGM1L4   ----------------------------------------------------------------------------------------------------
coelacanth_TGM1L2   ----------------------------------------------------------------------------------------------------
coelacanth_TGM1L3   ----------------------------------------------------------------------------------------------------
zebrafish_TGM1      ----------------------------------------------------------------------------------------------------
zebrafish_TGM1L1    ----------------------------------------------------------------------MPGERQSIRDNSAVGRFHGVSARNGEAVAE
zebrafish_TGM1L4    ----------------------------------------------------------------------------------------------------
zebrafish_TGM1L2    MPFSCSPYNNNL-CGRFTPIKLMADDCRRGACYSPYANSWYNPCASSCGKINRCATPCADPCGYPYGYSYGYPYGSNCGYNYGYPYGYNCGYNYGYPYAN
Zebrafish_TGM1L6    --------------------------------MMPFVNNPCKSCTTGRFPVFKHETDNSCK-GACYPTVYTHPYASHYSSHYTHPCVNPCANTHINPWAN
zebrafish_TGM1L3    MPIVNGGLNNFCNIGRFPAVKLQDCFWKGSGYVPVYSNPCVKPCVDPCW--NPCVKPVVDPCGNPCVKPVVDHCGKPVISPCGKPIVDPCGKPVVDPCGK
zebrafish_TGM1L5    ----------------------------------------------------------------------------------------------------
lamprey_TGM1L2      ----------------------------------------------------------------------------------------------------
lamprey_TGM1L1      ----------------------------------------------MADREELGRQYAEGRRHRLSSVCPSPVGSGQFPGI-GFPPGRTAPSK--GHSLR
lamprey_TGM1L3      ----------------------------------MLGHGNLETAQPAATRSNVVGRFAGAPAAPLADG-PGGHPNGAPPGTPGTPPGPPPPRSRLGRCCD
lamprey_TGM1L4      ----------------------------------------------------------------------------------------------------

                    101                                                                                                200
human_TGM1          RGGGRSFWARCCGCCSCRNAADDDWGPEPSDSRGRGSSSGTRRPGSRGSDSRRPVSRGSGVNAAGDGTIREGMLVVNGVDLLSSRSDQNRREHHTDEYEY
chicken_TGM1        RRLGG-----CCGCCGCCRRGKEDWDPPPGEVPGR-------RPPQ---------------------ALKPGLLVPRGL-VVGSRAD--RIAHHTAEFCS
frog_TGM1           RPDGGIGDNRPDGGSRDNRPDGGSRDNRPDGGSRDNRPDGGSRDNRPDGGSRDNRPSVNPPSPSLTPSIG-GPLQVQNVDLLKQRGGETRRAHHTDEFEY
coelacanth_TGM1     NGTRGKTEAQRAKKSLW--RCCPCLGLQRGSTIEVDTDECE---GTV-PAVEEDPK-----------EMKEFPLTVKNVNLVRGRDEINKKSHHTDEFEY
coelacanth_TGM1L1   -----------------------MTPTSVNKLGEKDEEMPQGRNPSL-ICNTISRGAKALSDDNTIKE--ESLLEVRNINLLKKIHENNKIFHHTDEFES
coelacanth_TGM1L4   -----------------------MTPTSVNKLGEKDEEMPQGRNPSL-ICNTISRGAKALSDDNTIKE--ESLLEVRNINLLKKIHENNKIFHHTDEFES
coelacanth_TGM1L2   -----------------MLSTKSKAKDDPGRRNLGCSSNTQKEKPKL-VQEAASLQENKEDEVNGAAEQKENLLKVKNIDLLKKPEEINKKSHHTDEYEY
coelacanth_TGM1L3   ----------------------------MSGSRFEEKKRALDSNVQT-LEEVQPIILKRVRRDSGPSAVGENILTVKSINLLKGKEEINKKSHHTDEFEY
zebrafish_TGM1      ----MEKTEQKWSCGAWFRQCCCSC--RKSHKNTNSTPHAA---NTT-AEKTETP-------------ATGSLLEVRSVNLLKSSKEQNRLEHHTERYRS
zebrafish_TGM1L1    KPSESKTKKPESGCRRWLRKACPCC-LRRQSNSYDLTSEAD---GVV-AGNEEIEQPASPVSVSVETNVDDLSLVVRSVDLLSRKTDRNKKEHHTDQYSG
zebrafish_TGM1L4    -------------------------MSSSSTPARPAPSAAT---ASP-SPSAPAPAAVSAASISPSASASDSVLAVRSVDLLRLRDGLNRRQHHTDGFSS
zebrafish_TGM1L2    RCVNPCTT--TTPYVKSCVNPCLDTCVNPCVNPCATPCSTYSYRDYE-SRQVVTRECIDDCTTKRVCATDEALLRVSKVDLLSCRTGPNRMEHRTHFFHE
Zebrafish_TGM1L6    VHTNPCANVHTSPCTKPCINPCATSWATPHTNPCSFVTPCH---DYK-TKEVIIKDYKDESISKQVSGGNVDLLRVIKIDLLKCKTGPNRQEHHTHFFHD
zebrafish_TGM1L3    PIFDPCGKPVVDLCGKPVVGPCVVPLPKPCVNPCDVRPHC----DYD-CKQVFLKDCADDCNAARCC--EDLVLQVRCVDLMKCWKSQNRQEHHTNGFRG
zebrafish_TGM1L5    ------------------------------------------------------------MNLVFVPAVLDAVLQVRSVDLIKTRKGQNRQEHHTDAFFS
lamprey_TGM1L2      ------MWEDFGDFHAEAEGSGARRGKTSAPRSFAPIDTVLTAVEDTTGSSSSEVAGGRPRTTGPPGAGGVPLLGVWAVELHGEQEGRTRLEHRTERYET
lamprey_TGM1L1      RLCCCCLPARRA--PGG----------------EDDHTAPLSSAKNQHAAEEEGAEEETELLG----ERLAVLRVESAGKEEAA------REHRTGGFAQ
lamprey_TGM1L3      WFFCCRCGSRAAGGPGGGRGGPGVAGEVEMKPREPPATPGGPGVPPMPSADEDPAGEPTPVDPVTGVSRLEALWVDAGGRGGGGPGAWNRARHRSAEYAA
lamprey_TGM1L4      ------------------------------------------------------------------MPRDTVGERLAVLRVESAGKEEAAREHRTSGFAQ

                    201                                                                                                300
human_TGM1          DELIVRRGQPFHMLLLLSRTYE-SSDRITLELLIGNNPEVGKGTHVIIP--VG---KGGSGGWKAQVV--KASGQN------------------------
chicken_TGM1        PQLVVRRGQPFHLRVLLPRPFDPEDDSLCVELLLGPTPQVAKGTHVLIP--LG---ETSATGWTAEEAGEEAEGEEASGSPA------------------
frog_TGM1           DELIVRRGQPFKICVTFQRAFNPKSDRICVVLQMANAQPI-----TLSQ--VN---EFDESGWACQVA--EVNGCK------------------------
coelacanth_TGM1     DELIVRRGQPFQIKLELSRPFNLETDKLFLELQTGLLPQVSKGTLVIIPV-VE---ELEYNEWGVKIVEATDNV--------------------------
coelacanth_TGM1L1   DYLIVRRGQPFEIKVELSRPFDAQTDMLLLELQIGPKPRLKKGTLVIVQV-VK---EHNPKEWGMKTFWETEQT--------------------------
coelacanth_TGM1L4   DYLIVRRGQPFEIKVELSRPFDAQTDMLLLELQIGPKPRLKKGTLVIVQV-VK---EHNPKEWGMKTFWETEQT--------------------------
coelacanth_TGM1L2   DELIIRRGQPFQVKLEFSRPFNPETDKLFLELKLGSQPQVGKGTHVIVKL-VE---KHIPSEWGAKIIEASGNT--------------------------
coelacanth_TGM1L3   DELIVRRGQSFHIKLELSRPFNPEADKLLLEMHLGPKPQFGKGTLAIVRL-VQ---ELDPKSWSMKIVKVSDRV--------------------------
zebrafish_TGM1      ENLIIRRGQTFQMQIELSRAFDPKTDKLHLDLKLGDLPDVSKGTHVVVPL-VE---ELQDNCWEAKIVEQKGRL--------------------------
zebrafish_TGM1L1    DQLIIRRGQTFQIELELSRPFNPNTDKLHLELKTGALPLVSKGTHIIIPL-VE---ELQDERWEAKIVEQNLNR--------------------------
zebrafish_TGM1L4    ERLIVRRGQSFQITVELSRAFKPRADSLQLQLKLDSAVSNSSGLLISVPL-VE---DLEDRRWEMKIVEQKENR--------------------------
zebrafish_TGM1L2    EKLIVRRGQCFNMWIDLCRPFNPTCDKLHLELRLGHIPSIRDGTYVIVPI-VD---EFKKDCWGARIVERCQNR--------------------------
Zebrafish_TGM1L6    EQLIVRRGQSFNMWVDLCRPFNPASDKLHLELRLGHIPSIRDGTYVIVPI-VE---EFKKDCWGAKIVEHGQNR--------------------------
zebrafish_TGM1L3    DHLIVRRGQCFQMWVELSRPFNPKCDQLHLELKLGNVPSIPNGTLVIVPL-VE---EFKKNRWEAKIVEKCQNR--------------------------
zebrafish_TGM1L5    NHLIIRRGQCFQMTIELSRPLIPNKDQLYLELRLGNVVPAHRDSFVSVPI-VS---EFKKNAWEAKIIEQAKTT--------------------------
lamprey_TGM1L2      PLPVLRRGLPFPVTITFSRPFEPRSDRLQLELYIGKYPRIDRGTYIPIPL-AEPGEEVEPGTWKAKVTGSEGAR--------------------------
lamprey_TGM1L1      PQLVLRRGAPFRLRLKLSRAVQRHHDSIVLEFSTGLSPQLRKGTLIQVPLRWELPPEQQEGAGGQRDGGAEGQRQEGGGEE----GGGWSAVVEREEERA
lamprey_TGM1L3      GHVVTRRGGPVLLHAAFSRTPTDGVDRVSVELEIGSHPQLNKGTHLIFPVETRAAGEGDVAASSSSSAAASSSSTASYDDDTDCTSGAWWASGGLTEAGG
lamprey_TGM1L4      PQLVLRRGAPFRLRLQLSRAVQRHHDSIVLEFSTETA-----GPPVSVPVGPR----LREETWSATLSEETSGTRGGRGGQ----------------GVE

                    301                                                                                                400
human_TGM1          LNLRVHTSPNAIIGKFQFTV----RTQS-DAGEFQLPFDPRNEIYILFNPWCP-----------------------------EDIVYVDHEDWRQEYVLN
chicken_TGM1        LRLRLSAPADAPIGRYRLSV----KTRT-GAGEFGAPFDDRNDVIVLFNPWCE-----------------------------EDGVYMEQTNDLNEYVLN
frog_TGM1           SNIWLNTSAQAPIGLYQMIV----KTNS-GEGPSNL------RVYILFNAWCR-----------------------------MDSVFMDNEAWRQEYVLN
coelacanth_TGM1     LSLLVNSSPQAVIGRFELTV----KLQPAGERENRTEHKPANDIYILFNPWCE-----------------------------ADAVFVEDEEWRKEYVLN
coelacanth_TGM1L1   LTLSVNSSPEAVIGRYQLTV----RTLT-RKEEFRTKHNPDNDIYILFNPWCE-----------------------------ADIVYMENEEWRKEYVLN
coelacanth_TGM1L4   LTLSVNSSPEAVIGRYQLTV----RTLT-RKEEFRTKHNPDNDIYILFNPWCE-----------------------------ADIVYMENEEWRKEYVLN
coelacanth_TGM1L2   LTLSINSSPQTMIGRFELTV----KTVT-EGGAFKMKYNRDNDIYFLFNPWCE-----------------------------ADTVYMEDEEWRKEYVLN
coelacanth_TGM1L3   LTLKVNSSPEAPIGRYQLIV----KSLT-LNEEFRTKHNPDDDIYILFNPWCK-----------------------------ADTVYMEDEERRKEYVLN
zebrafish_TGM1      IKLSVNSLPTAPIGKYKLGV----ATSC-PVGESMSPYNPDNDIYMLFNPWCE-----------------------------EDSVYMDSEKERKEYVLN
zebrafish_TGM1L1    AKLSINSSVNAVIGKYTLTV----VTQCLKTNESTT-HDPEKDIYMLFNPWCE-----------------------------DDAVFMEGERELNEYVLN
zebrafish_TGM1L4    VRLVVNTLPSASIGCYKLTV----VSFS-PRGKLLFPCTPD-DVYLLFNPWCE-----------------------------DDPVYLDNEAERKEYVLN
zebrafish_TGM1L2    LKLCVNSLPTSCVGRYQLSV----VTHC-SAGRFCLPYVPENDIYMLFNPWCK-----------------------------EDCVYLHEETERAEYVLN
Zebrafish_TGM1L6    IKLCVNSLSTACVGRYQLSV----VTQC-TAGKFTLPYVPEHDIYMLFNPWCK-----------------------------EDSVYLSDQAERTEYVLN
zebrafish_TGM1L3    IKLSVYSLPTACIGRYSLTI----VTCG-PKGRATSSCNPSNDIYMLFNPWCK-----------------------------DDAVYLDEDAQRTEYVLN
zebrafish_TGM1L5    IKLSVYSLPTACIGQYKLTV----VTNC-PAGKATSPYTPDNDIYMLFNPWCK-----------------------------DDPVYLKDEAERNEYVLN
lamprey_TGM1L2      LALLVHTSPKCIVGKYRFHV----ATLS-AGGLYRSSRDPNTDLYFIFNPWCP-----------------------------EDSVYMESEAEREEYVLN
lamprey_TGM1L1      LHVLVRSCPRSVVARYEVAA----CAHSETAVEEVRSKREPLSVYILFNPWCR-----------------------------EDTVYMESEEERQEYVLR
lamprey_TGM1L3      ANGRNRALWLRVRADCLVGKWRLFVSTQGPAGSHRSARREESDIYIIFNAWCP-----------------------------EDAVFMEVEAWRREYVLA
lamprey_TGM1L4      IEVTVQSPPHCPVGQYRLAV----AVSPAGGRPVRTEPGVCPDVIILFNPWCRGGSSCVLIKNRLESILTNTYLRDPVEDGTRDAVFMEPEEHKTEFVLN

                    401                                                                                                500
human_TGM1          ESGRIYYGTEAQIGERTWNYGQFDHGVLDACLYILDRRGMPYGGRGDPVNVSRVISAMVNSLDDNGVLIGNWS--GDYSRGTNPSAWVGSVEILLSYLRT
chicken_TGM1        ETGRIFYGTEEQIAERSWNYGQFDAGVLDACLAILDRRRMPHSARGDPVMVTRVVSAMVNSLDDNGVLVGNWT--GDYTQGTNPSAWAGSVDILRSYHRG
frog_TGM1           EIGRIYYGTQNQIGERSWNYGQFDKGVLDACLYLLDCGRIAPGSRGDPINVTRVISAMVNSMDDNGVVAGSWS--GDYADGVNPVAWVGSVDILLRYHQS
coelacanth_TGM1     ETGRIYYGTKNQIGARTWNFGQFDKGVLDACLYLLEKGKMPHSGRGDPISMVRVVSAMVNSQDDRGVLVGNWS--GDYTGGSAPTEWVGSVDILLQFHRT
coelacanth_TGM1L1   EVGTLYYGTSEAIKSKMWNFGQFEKGILEACFNLLEKGRLPQYVWGNPITLVRFISAMVNSQDDDGVVIGNWT--GDYSEGTAPTAWAGSVDILHQYHQT
coelacanth_TGM1L4   EVGTLYYGTSEAIKSKMWNFGQFEKGILEACFNLLEKGRLPQYVWGNPITLVRFISAMVNSQDDDGVVIGNWT--GDYSEGTAPTAWAGSVDILHQYHQT
coelacanth_TGM1L2   DTGRLYYGTENSIGSRTWCFAQFEKGILEACFYLLDKGGMPDSGRGDPISVVRVISAMVNSQDDDGVLVGSWS--GVYWDGRRPTSWTGSSEILLHYHDS
coelacanth_TGM1L3   DTGKLYRGMQRWIGASDWNFGQFDQGILEACFFLLDKGKMPHAGRGDPISMVRVVSAMINSADDDGVLVGNWS--GDYSDGITPMAWGGSVEILLGYYST
zebrafish_TGM1      DMGIIYYGTDSQIGYRTWNFGQFDKSILPACLFLLERSGAPASGWGDPVNVVRLLSAMINAPDDNGVLVGNWS--GTYDDGTAPTFWSGSSDILRQYYNN
zebrafish_TGM1L1    DTGRIYYGTEKQIGARTWNFGQFDEGILEACLFVLDNSEVPPSGRGDPVNVVRVISAMINSPDDRGVLEGNWS--GNYTGGTSPTAWSGSVEILKQYHRE
zebrafish_TGM1L4    TMGRIYYGTEQQIGTRTWNFAQFEQNILEACLFLLERGRVAVTEWRDPVIISRMVSALVNSNDDRGVLMGNWS--ESFEGGTAPTAWSGSGDILRQYYSS
zebrafish_TGM1L2    DIGKIYYGTKHQIGCKSWNFGQFEEGILPACFYVLEKSCAPCSGWGNPINISRVVSDMVIAKKDCGVLMGNWS--NCYTDGIAPTSWCGSSAILRQYYKC
zebrafish_TGM1L6    DMGKIYYGTKQQIGCKTWNFGQFEDGILPACFYVLEKSGTPCSGWGNPINISRVVSEMINANKDRGVLIANWS--NYYVDGTAPTLWSSSSAILKQYHKC
zebrafish_TGM1L3    DTGKIFYGTKHQIASRTWHFGQFDEGVLAACLFVLEKCGGACSGWGDPVNVARVVSAMVNANDDSGVLMGNWS--NCYADGTAPTAWCGSSAILKQYHKC
zebrafish_TGM1L5    DMGKMYYGTEQQIGTRTWNFGQFDEGVLEACFFVLEKSGSPCSGWGDPINVVRVISALVNSNDDQGVLIGNWQ--NSYEGGLSPTAWSGSSAILKQYHKS
lamprey_TGM1L2      DIGRIFHGTRDQIVSRVWNFGQYEYGVLDAALSVLDCGRLPLPGRDNPVTVARVASAAVNSQDDNGVVEGCWV--DNYTGGVAPTAWNGSAEILLDFKMS
lamprey_TGM1L1      ETGLIYYGTKEQISARPWVFGQFGKGILDTCLHLLDRANMPLYGRNDPVNVARVVCGIVNSQDDSGVLEGNWS--GDYTEGTSPAAWTGSAEILLGYHKG
lamprey_TGM1L3      DTGRIYYGTEQQIGSRPWNYGQFEKDVLEASVMIMDRASLPLAGRGCPVKVVRIVSAMVNSLDDDGVLEGNWS--GDYGGGTAPTAWNGSVDILQQFARS
lamprey_TGM1L4      ETGRLFYGTAQQIGTRAWNYGQFTSGVLDASLHLL--SSMAWEQRGDPVAVVRTVSALVNANDESGVLEGNWSERNDYSGGTAPAAWTGSPDILRQYHRS

                    501                                                                                                600
human_TGM1          G--YSVPYGQCWVFAGVTTTVLRCLGLATRTVTNFNSAHDTDTSLTMDIYFDENMKPLEHLNHDSVWNFHVWNDCWMKRPDLPSGFDGWQVVDATPQETS
chicken_TGM1        G--APVRYGQCWVFAGVVTTVLRCLGVPTRTVTNYNSAHDTDVSLTTDIYFDENMKPLERLNTDSVWNFHVWNDCWMRRPDLPAGYDGWQVVDATPQETS
frog_TGM1           G--SSVKYGQCWVFGGVTTTVLRCLGIPGRTITNFASAHDADGNLTFDIYFDENMKPIEDKNRDSVWNYHVWNDCWMTRPDLPAGYSGWQAIDSTPQETS
coelacanth_TGM1     G--EPVNYGQCWVFSGVTTTVLRCLGIPGRSVTNFASAHDTDVSLTTDVYLDENLEPLEHMNFDSVWNFHVWNDCWMARLDLPPGYGGWQAIDATPQETS
coelacanth_TGM1L1   G--KPVKFGQCWVFSAITTTVLRCLGIPTRSLTNFDSAHDTDVSLTMDIYIDEALEPIEEMNLDSTWNFHVWNECWMARPDLPPGYGGWQAVDATPQEMS
coelacanth_TGM1L4   G--KPVKFGQCWVFSAITTTVLRCLGIPTRSLTNFDSAHDTDVSLTMDIYIDEALEPIEEMNLDSTWNFHVWNECWMARPDLPPGYGGWQAVDATPQEMS
coelacanth_TGM1L2   Y--NPVRYGQCWVFSGVATTVLRCLGIPGRSVTNFSSAHDSDTSLEIDYYIDEDHCALDHLNNDSIWNFHVWNECWMARPDLPQGYGGWQVVDATPQETS
coelacanth_TGM1L3   GG-EPVSFGQCWVFSGLTTTVLRCFGIPTRSVTNFDSAHDTDVSLSMDTYYDEDMEEIRDLNSDSVWXXXXXXXXXXXXXXXXQPVATYLYVHTNHFLFS
zebrafish_TGM1      GG-TPVRYGQCWVFSGVTTSVMRCLGIPTRSITNFESAHDTDASLTTDVYLDENHELIEELCSDSVWNFHVWNDCWMARPDLPAGLGGWQVVDATPQETS
zebrafish_TGM1L1    GG-TPVKYGQCWVFSGVTTTVLRCLGIPARSVTNFQSAHDTDVSLTTDVYFDEDMEPIHHLNSDSVWNFHVWNDCWLARPDLPPGFGGWQAVDSTPQETS
zebrafish_TGM1L4    KG-SPVRFAQCWVYAGVTCTVLRCLGIPTRCVTNFSSAHDTDLSLTTDIYLDEKLEMLKDKTSDSIWNFHVWNESWMRRGDLPAGYGGWQVVDATPQEQS
zebrafish_TGM1L2    GG-APVRYGQSLAFAGVTNTMLRCLGIPTRPVSNFCSAHDTDMCLTSDVYLDEKFQLIDHMNANPIWNYHVWNEAWMTRPDLPTGFGGWQAIDSTPQLTH
zebrafish_TGM1L6    GG-VPVKYGQSLAFAGVTNTMLRCLGIPARPITNFCSAHDTDVSLTTDVYLDEKFQLIDQMNRDSIWNYHVWNEAWMTRPDLPTGFGGWQAIDSTPQQTH
zebrafish_TGM1L3    GG-VPVKYGQSLAFAGVTNTLLRCFGIPARPVTNFCSAHDTDVSMTVDIYLDENYDLIDSLNRDSIWTFHVWNEAWMARSDLPAGFGGWQAIDATPQETS
zebrafish_TGM1L5    GG-TPVKFGQCWVFAGVTNTMLRCFGVPTRPVSNFSSAHDTDVSLTTDVYLDEKLEEIKDLNRDSIWNFHVWNESWMTRPDLPAGFGGWQVVDATPQETS
lamprey_TGM1L2      R--RPVKYGQCWVFAGVATSVLRCLGIPSRPVTNYCSAHDRDASMSYDVYLDESLAPRDDLNKDSIWNYHVWSECWMARPDLPLGYGGWQIVDATPQENS
lamprey_TGM1L1      ARPAPVRFGQCWVFSGVTTTVMRCLGIPCRSVTNFCSAHDTDVSLTTDVYVDEKMKPIDDLNTDSIWNFHVWNECWMARPDLPMGYGGWQVIDTTPQETS
lamprey_TGM1L3      R--SPVRYGQCWVFSGVTTTVLRSVGIPTRSVTNFASAHDTDTSLTTDVYLDDDLQPIAELNTDSVWNFHVWNDCWMTRPDLPEGLGGWQAVDATPQETS
lamprey_TGM1L4      G--RPVKYGQCWVFAAVTTTVLRALGIPCRTVTNFSSAHDTDGNLVTDIFLDEKMRPDRSHTKDSIWNFHSWSDAWMKRPDLPSGYDGWQVVDATPQEAS
```

```
                   601                                                                                              700
human_TGM1         SGIFCCGPCSVESIKNGLVYMKYDTPFIFAEVNSDKVYWQRQDD--GSFKIVYVEEKAIGTLIVTKAISSNMREDITYLYKHPEGSDA--ERKAVETAAA
chicken_TGM1       SGLFCCGPCSVTAVKNGEVFLKYDTAFVFAEVNSDKVYWQRKGN--GAFAIVHVEEGAIGRRISTVGPQSAARIDITHLYKHPEGSEA--ERRAVSTATS
frog_TGM1          NGIYCCGPCPLLAIKNGLTNIKYDAPFIFAEVNSDKVCHQRMPN--GQFKRVLVEERAVGHCISTKAVGSFARDDITYLYKHPEGSTE--ERNSVHTAAR
coelacanth_TGM1    AGTYCCGPASLQAIKSGLVYLKCDAPFIFAEVNSDRIYWQRQHN--GTFQKVLVQKNAVGHQISTKAVGLDEREDITHLYKYPEDSEE--ERIAVETACQ
coelacanth_TGM1L1  VGKFCCGPASVRAIKNGLVYLNWDSPFIFAEVNCDIVYWKKKKDGSGIFRPIFKEESAIGHFISTKAVGSNKREDITHLYKYPEGSEE--ERISVETACQ
coelacanth_TGM1L4  VGKFCCGPASVRAIKNGLVYLNWDSPFIFAEVNCDIVYWKKKKDGSGIFRPIFKEESAIGHFISTKAVGSNKREDITHLYKYPEGSEE--ERISVETACQ
coelacanth_TGM1L2  AGTYCCGPASVRAIKNGLVYLKYDAPFIFAEVNSDKVYWIR--DSNGDFKRYYSARSVVGHNISTKAVGSEERLDITNLYKYPEGSEE--ERLSVKTACK
coelacanth_TGM1L3  KGTYCCGPASLRAIKNGLVFLKYDAPFIFAEVNCDRIYWQRKAD--GTFEKFDVVKNAVGHKISTKAVGSDEREDITHLYKYQEGSEE--ERISVQTACQ
zebrafish_TGM1     QGAFRCGPASVAAVRNGLVYLKHDTPFVFAEVNSDKVYWQRTAD--GSFTPVQIKKKAVGHCISTKAVGSDEREDVTHLYKYPEDSEE--ERIAVETAVQ
zebrafish_TGM1L1   HGTFRCGPASLAAVRSGQVYLKYDVPFVFAEVNSDKIYWQRNLD--GTFSQIHSEKKAVGHCISTKAVGSDERVDITDVYKYPEGSEE--ERIAVETACR
zebrafish_TGM1L4   QGSYRCGPTPVSAVRSGQVNLRFDTPFVFAEVNSDKIYWQRNAD--GSFRQVSVEKNSIGQKTSTKAVGSDTRVDITHLYKYPEGSEE--ERIAVESASR
zebrafish_TGM1L2   QGFFRCGPTSVAAIRSGQTFLKHDVPFLFAEVNNDKVYWQRKCD--GTFGVVHVEKDVVGHCISTKAVGSDQRLDITNLYKHPCGSED--RCTALETALR
zebrafish_TGM1L6   QGFFRCGPTSVAAIRSGQTFLKHDVPFLFAEVNSDKVYWQRKNN--GTFGVVHVEKDVVGHCISTKAVGSDQRVDITNLYKHPCGSED--RLTALETALR
zebrafish_TGM1L3   QGVFRCGPTSVAAIRSGQVFLKYDAPFIFAEVNSDVVFWQRKAC--GTFAVVHVDKNAVGHCISTKAVGSDKRVDITNHYKHPEGSEE--ERRAVETALR
zebrafish_TGM1L5   QGVFRCGPTSVAAVRSGQVYLKYDTPFVFAEVNSDKVFWQRQSN--GSFTVIKVDENAVGHCISTKAVGSDQRVDITHLYKHPEGSSE--ERSAVEAACS
lamprey_TGM1L2     EGIFRCGPSPVNAVKSGIVYLPYDTKFVFAEVNSDKVYWKVGLD--GELSPVDVERRAVGHCISTKAIGSDNREDITHAYKYPEGSDE--ERVSVEMACR
lamprey_TGM1L1     LGFYRCGPASVNAVRDGQVQHKYDAPFVFAEVNSDRVYWMKQPG--GEFSVLSVDKQAVGHCISTKAVGSHEREDITGLYKHPEGSKE--ERIAVETACS
lamprey_TGM1L3     SGVFCCGPASVAAVRNGLVYLTHDTPFVFAEVNSDRIFWLRSAD--GTVKRLDVERRAIGHCISTKAVGSDTREDITHLYKFPEDTEE--ERLAVETACL
lamprey_TGM1L4     AGVFRCGPAPVMAVKKGDIDLKYDTAFVFAEVNSDRVFWRRRAG--GERERISVETDSIGQKISTKAPGSDARLDITAEYKPSEGNRELGQQEGSSYGTR

                   701                                                                                              800
human_TGM1         HGSKPNVY---ANRGSAEDVAMQVEAQ-DAVM--GQDLMVSVMLINHS--SSRRTVKLHLYLSVTFYTGVSGTI-FKETKKEVELAPGASDRVTM-----
chicken_TGM1       HGSRPRSG---AAPSRGE-VRLSLSSG-PAVA--GAELELKVTAHNAA--PQPRTVRVRLSVCALRYTGVAAPP-FRHEQHRRVVAPGGEEQLCV-----
frog_TGM1          YSNKPLAA---VDNEETSDVSMSVESQ-DGVI-TGSDINVRVLLKNNS--NSRRCVSLSLTVGVMYYNGICKES-FKNETRDTTLNPGEGKAVGM-----
coelacanth_TGM1    YGSKPTVY---LNSAVMEDVALDIQTQ-EDIQ-MGSDVTVRVLVENCS--SEHRSISLFLKAAVVYYTGVYKNS-FKQDREEVLLSPAEGKELLV-----
coelacanth_TGM1L1  HGTKPKTY---LEGEKVKDVVINVQTE-KNLQ-MGSDFTVWLRVQNCS--TEQRGISLFTQVAAIYYTGVYKSC-FKKKREEVQLSASEVRELEL-----
coelacanth_TGM1L4  HGTKPKTY---LEGEKVKDVVINVQTE-KNLQ-MGSDFTVWLRVQNCS--TEQRGISLFTQVAAIYYTGVYKSC-FKKKREEVQLSASEVRELEL-----
coelacanth_TGM1L2  FGNKSKMS---IEKEVEEDVVVEVEIT-GNDL-IGSNIIARATVTNRG--NNVCNICLFMRAAMTFYTGVYKNE-IKNMKEEVTLEPAEVKEVKM-----
coelacanth_TGM1L3  YGTKPNLY---RKDDAANDVTADIKAE-DKIQ-IGSDVMLKISLGNRS--MDKRTVSLFVQSSAILYTGVNKGT-FKREQEEIVLGPCEVKEMVK-----
zebrafish_TGM1     HGSKAGLY---EISSV-NDVSIEISMDGEDPQ-LGSDANLAITVKNSS--SEMRTFQLSAQVAVTYYTGVYKGT-VRKDQISIELKPNEAETVEW-----
zebrafish_TGM1L1   YGSKPDVY----SSAMAEDVQVEVRMEGEGPR-MGGDAQLKIVVKNMS--SQPRRTTLHSQAAVMYYTGVLKDT-VKKDKLSVELMPQEEKVIEW-----
zebrafish_TGM1L4   FGSRPTLY----PSPSGTDVSLEVQMSGAGPR-IGEDVQLSIVLKNSS--SAQRSASLLYEALVMYYTGVLKQS-LKKDRITLELQPRETKTIPW-----
zebrafish_TGM1L2   HGCRRINYP--LPCAE--DVVCEVNLKGDGPC-VGRDAVVCINLRNKC--NQPRSVTLYSHAAAMYYTGVRRTY-LKRDQTCIELKPSECKPLEW-----
zebrafish_TGM1L6   HGCKRLTHP--LPIPE--DVICEVNLKGDGPL-VGKDAVLSINLKNKC--NQPRSITLYSHAAAMYYTGVRRTY-LKRDQTSIELKASESKLLEW-----
zebrafish_TGM1L3   HGSKKCAYP--LPCAE--DVICDITMKGDGVC-VGKDAVLCIALKNKC--SSTRSVTLHSQLSAAYYTGIHKSL-VKKDQTCFELKATETKVLEW-----
zebrafish_TGM1L5   FGSKRSIYL--PRSSSNTDVTVDVVMEDSGAC-LGQDAVLFIVLKNRS--SSARTVDLQSRVEAVDYTEHHKAF-LRKDQTRAQLKPHEIQSLEW-----
lamprey_TGM1L2     YGTKPSLLAEALGSRPNHDVVLEVTAPLDGALTMGRDVALGVRLKNHSVDGEERHVSLLLHCDALFYTGVVRTA-VKRQRFDLDLPPGAEHQVVL-----
lamprey_TGM1L1     QGSKANIY---KVPGTAQDVRLLVKPPVDEVA-LGRDFAVTLELANLDA-DETRTVVLFCAAHGMHYTGVLRGR-VKQHSWEVQLAPGEECTLSL-----
lamprey_TGM1L3     HGSRPDTY---RGSPSSPDVTLAISTH-ERVV-MGHDFDVSVTVSSTAV-SE-RQVSVFVRGAVMHYTGVTRGEAIKQLKLDVTLQPGEERVLTV-----
lamprey_TGM1L4     GNKGVVTEAIVVAMSACSEEERRVVTHALSLLKRGGAERGPREVETGGSGPPQGDNASGAGGPSGVERGRDAADIGGALDVRSVLNPDGHLIASLGGDPI

                   801                                                                                              900
human_TGM1         -PVAYKEYRPHLVDQGAMLLNVSGHVKESGQVLAKQHTFRLRTPDLSLTLLGA--AVVGQEC---EVQIVFKNPLPVTLTNVVFRLEGSGLQRPKILNVG
chicken_TGM1       -AVPFSEYSPHVGSQDALRLTAAAAVEETGEVVAKELRVRLAAPDLSMTLLGP--PVVGQEV---SVQVLFRNPLPQKLTGAELRMEGAGLSCPASISVG
frog_TGM1          -LISYAEYSKHLVDQGAMMLTVSGIVNETGQKLAKQHTFRTRTPDLVIKVRGD--AIVGQQI---VAEVIFKNPLNTTLHNAVFHVEGPGLQRPKVIKYG
coelacanth_TGM1    -VLSYSQYQEYLVDQAAMMLTVSGRVVETGQVLAKQHNFRLRTPDLQIMPYGE--AIVGGQM---KAEIVFLNPLPKILKNVTFRIEGPGLQKPKKVQVG
coelacanth_TGM1L1  -VIKYSEYDPHLESQDNMLFTVLGRVAETRQVIAKQHKFCLCTPDLQIRVLGE--AIVGKEM---KAEIVFINPLPKILKNVQLHIEGPGLQTPKIVTIG
coelacanth_TGM1L4  -VIKYSEYDPHLESQDNMLFTVLGRVAETRQVIAKQHKFCLCTPDLQIRVLGE--AIVGKEM---KAEIVFINPLPKILKNVQLHIEGPGLQTPKIVTIG
coelacanth_TGM1L2  -TIKYDEYDDYLEDQACIMFTLMGLVSETKQIITKQRDYRLRTPDLMLKVHGE--VVKGKES---KVVISFVNPLPKVLKNVVIHLEGPGLQKRKTISVG
coelacanth_TGM1L3  -MLKFEEYDDHLVEHGSFMFTILGRVKETKQVFVKQDHACLSSSSLKIMILGQ--AEVDREV---QAKFEFTNSQTRRIRNVQLRIEGPGLQKPKTIYIG
zebrafish_TGM1     -TLLYDHYKDHLVDQAMLMLILTGRVNETKQVLVNQFHFRLRTPDLAIKTEGD--AVVGKEL---KASITFRNPLKQTLKNVKFRIEGLGLQHVREISHG
zebrafish_TGM1L1   -TLPYTQYQNQLVDQAALMLTLSGRVSETQQVLANQTSFRLRTPDLQIEPIGE--AYVGKEA---SAKISFTNPLPCTLRNVVVRVEGLGLRDLHPIKVG
zebrafish_TGM1L4   -TLQYKEYKEQLVDQGALMLTLTGRVSQTKQVLATQFNFRLRTPDLVLTPLQD--AVVGKEM---SVRISFQNPLSQVLKNVLFRIEGLGMQSVRKISYG
zebrafish_TGM1L2   -TLSYDEYKEHLVDHAPLMLNLFGHVAQTKQILATQYNFRLRTPDLVLAPACD--AVVGQEV---AVKVTFQNPLSCVLRNAAFRFVGLGLQHPRIINYG
zebrafish_TGM1L6   -TLSYDEYKEHLVDHAPLMLNLFGHVAQTKQILATQYNFRLRTPDLVLAPVCD--AVVGQEV---AVKVTFQNPLSCVLKNSVFRFVGLGLKQARVINYG
zebrafish_TGM1L3   -SLKYEDYKNHLVDHSTMMVTVAGRVTQTQQIVAKRFNFRLPTPGLAISPGCD--CVVGKEV---PVKITFQNPLPCVLKNAIFRIEGLGLKHCRSINYG
zebrafish_TGM1L5   -ILQYEEYKEELEGQTSLLLSLSGRITETKQTLVKHFTFRLRTPDLVLTPVGD--AVVGQEL---KVKLKFQNPLVSVLRNVIFRMEGLGLQHVKTIHYG
lamprey_TGM1L2     -QVRRGEYLGMLVDQGGLMLTATGRVLETSQPLVAQRAFHLTLAPLRITVLGE--VRSGWDF---MAEVSFTNPLPTVLQGVTFRLEATGLQQNKVIRHG
lamprey_TGM1L1     -PVSASEYLQSLVDQCAMLFTVTGRVGETGQTLATQRSFRVNFPALGVTVDDRDAVRVGNKA---LVKVSFTNPLPQPLRAVTLRLEAAGLLEPTLIQHG
lamprey_TGM1L3     -TVRQGEYLERLVDQSAVMFLVSARVNPGNAILTRQHCFRLCTPKLLVTCADE--CRVGEPL---TVEVLFENPLPHLLRDVSFRLEGPGLQSPRVIKHG
lamprey_TGM1L4     GAPAGGDPPPPEVTLAVRVLEETALGRELTAEVRVASASARPRVHVRVRVRGHGATYTGANLGGFEEEEALLELGPHEERRVLVRVPASEYLHLAVDHFA

                   901                                                                                988
human_TGM1         DIGGNETVTLRQSFVPVRPGPRQLIASLDSPQL-SQVHGVIQVDVAPAP-GDG—GFFSDAGGDSHLGETIPMASRGGA----------
chicken_TGM1       TVAPEQTLRLRQPVVPLRAGRRRLVAAMESAQL-GPVHGELQFDAVPGPDGDSVEGSAATNGNTRRRRRGGGGRRRGGRAGGSTGG--
frog_TGM1          NIGPLQTVCVSERFTPRRPGPRQFIASLESNEL-SQVHGTTEVVVQPC----------------------------------------
coelacanth_TGM1    DVGRHATVTLKETFVPTKPGPRKLIASLHCREL-TQVHGVAEVIVLSQ----------------------------------------
coelacanth_TGM1L1  DVGSHAKVTHTESLVPVRPGMRTLIANLDCPQL-SQVHGVAEILVKTEAH--------------------------------------
coelacanth_TGM1L4  DVGSHAKVTHTESLVPVRPGMRTLIANLDCPQL-SQVHGVAEILVKTEAH--------------------------------------
coelacanth_TGM1L2  EVAKQGLMTITEIFIPSKAGCRKLIANLDCHQL-TQVHGVVEVEVINKPAEV------------------------------------
coelacanth_TGM1L3  DIRGNQTMSHTEMIIPSKPGARKLIATLDSHQLITEVQGFANVYVKKPDQCV------------------------------------
zebrafish_TGM1     NIESLATVTLTETFIPLEAGQHKLLAALDSRQL-PQVHGVANITVKAN----------------------------------------
zebrafish_TGM1L1   DVGKHGKVMVTEHFIPSIAGERKLVASLDCKQL-TQVHGVADIIVHESQ---------------------------------------
zebrafish_TGM1L4   DVARLGTVSLIEKFTPTVSGSQKLLASMDCRQL-TQVHGVADITVKAK----------------------------------------
zebrafish_TGM1L2   DIAGHATVSLTEKFIPMCHGPQKLLASFDCPQL-TQVHGFTNMVVKQH----------------------------------------
zebrafish_TGM1L6   DIAGHATVSLTEKFIPMCHGPQKLLASFDCPQL-TQVHGFTNMVVKQH----------------------------------------
zebrafish_TGM1L3   DIAGLATVNLTEKFIPKCHGPHKLLASLDCPQL-TQVHGFTDVVVKEK----------------------------------------
zebrafish_TGM1L5   DITGGATVRLTEIFVPKRSGPQKLLATLDCPQL-TQVHGVANILVKHR----------------------------------------
lamprey_TGM1L2     DIRVGETVTVRERLTPTRPGLRKLAGSMVCRQL-TQVLGDTDLNVY------------------------------------------
lamprey_TGM1L1     DVPGGGSVYRTVAVVPRVAGVATLLATLDSHQL-ITVHGELELLVKA-----------------------------------------
lamprey_TGM1L3     NVSRGGVVRLRETLLPERPGQRKLLVSLHCTQL-SQVHGETDVLVSP-----------------------------------------
lamprey_TGM1L4     LVLVACARVLDTGAEVARVHVARVRSPRLRVHVPEGAHVGRDLISELCFTNPVARALHRVRVRVEGAGVHTPGTILVGYGAVVTSSRS
```

Figure S4. Amino acid sequence alignment of TGM1-like protein generated by lineage-specific gene duplications. *TGM1* underwent several species specific duplications in zebrafish (*Danio rerio*), coelacanth (*Latimeria chalumnae*) and lamprey (*Petromyzon marinus*). A cysteine cluster (thick underline) is present close to the amino-terminus of many but not all TGM1 proteins. All cysteine residues are highlighted by yellow shading. Residues of the catalytic triad are highlighted with red fonts. Note that three of the TGM1 copies in the zebrafish lack one of the critical residues and may therefore be catalytically inactive. Xs indicate unknown amino acid residue at sites corresponding to gaps in the genome sequence. Human (*Homo sapiens*), chicken (*Gallus gallus*) and frog (*Xenopus tropicalis*) TGM1 sequences are included for comparison.

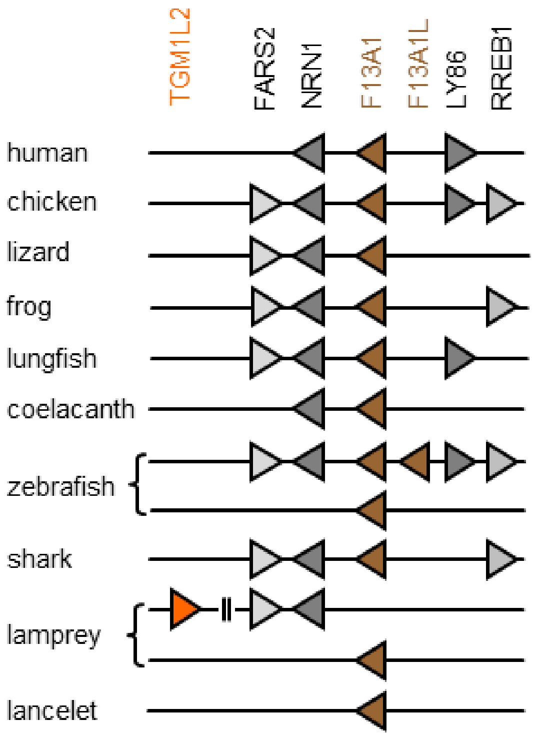

Figure S5. Synteny analysis of *F13A1* loci in vertebrates. Triangles represent genes, which are shown in direction of transcription. *F13A1* is flanked by *NRN1* and *LY68* or *RREB1* in all investigated species, except for lamprey and lancelet. The zebrafish has three *F13A1* paralogs, of which *F13a-like* and *F13A1a.1* are located in tandem at a locus syntenic with that of human *F13A1*. In the lamprey, a gene termed *TGM1L2,* which groups with *TGM1* genes in the phylogenetic analysis (Figure 2), is syntenic with *F13A1*. *F13A1* of the lancelet is not syntenic with TGM genes of vertebrates investigated here. Species: Human (*Homo sapiens*), chicken (*Gallus gallus*), lizard (*Anolis carolinensis*), frog (*Xenopus tropicalis*), lungfish (*Protopterus annectens*), coelacanth (*Latimeria chalumnae*), zebrafish (*Danio rerio*), shark (*Carcharodon carcharias*), lamprey (*Petromyzon marinus*), lancelet (*Branchiostoma floridae*).

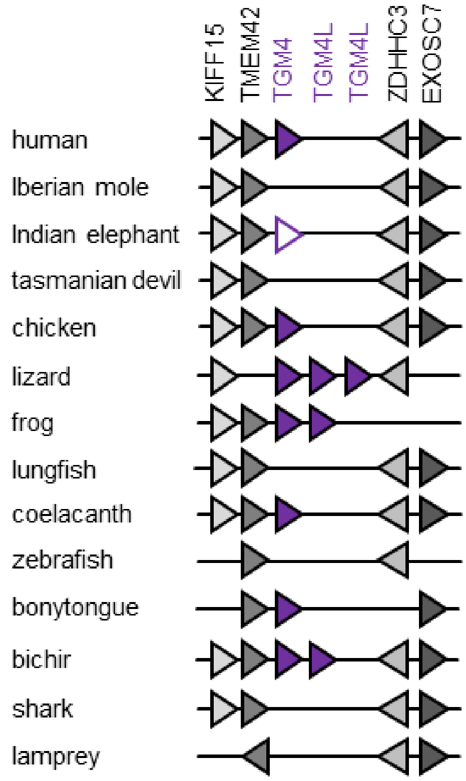

Figure S6. Synteny analysis of *TGM4* loci in vertebrates. Triangles represent genes which are shown in direction of transcription. *TGM4* is flanked by *KIFF15* or *TMEM42* and by *ZDHHC3*. *TGM4* is present in terrestrial vertebrates and coelacanth but not in lungfish, zebrafish, shark and lamprey. Species: Human (*Homo sapiens*), Iberian mole (*Talpa occidentalis*), Indian elephant (*Elephas maximus indicus*), Tasmanian devil (*Sarcophilus harrisii*), chicken (*Gallus gallus*), lizard (*Anolis carolinensis*), frog (*Xenopus tropicalis*), lungfish (*Protopterus annectens*), coelacanth (*Latimeria chalumnae*), zebrafish (*Danio rerio*), Asian bonytongue (*Scleropages formosus*), grey bichir (*Polypterus senegalus*), shark (*Carcharodon carcharias*), lamprey (*Petromyzon marinus*).

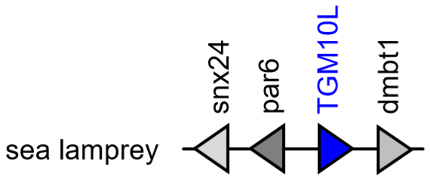

Figure S7. A TGM10-like gene is present in the lamprey (*Petromyzon marinus*). Triangles represent genes which are shown in direction of transcription. On chromosome 63 of the lamprey, a *TGM10-like* (*TGM10L*) gene (GenBank accession number of encoded protein: XP_032833357) is flanked by *snx24* (XP_032833523), *par6* (XP_032833413) and *dmbt1* (XP_032833453)

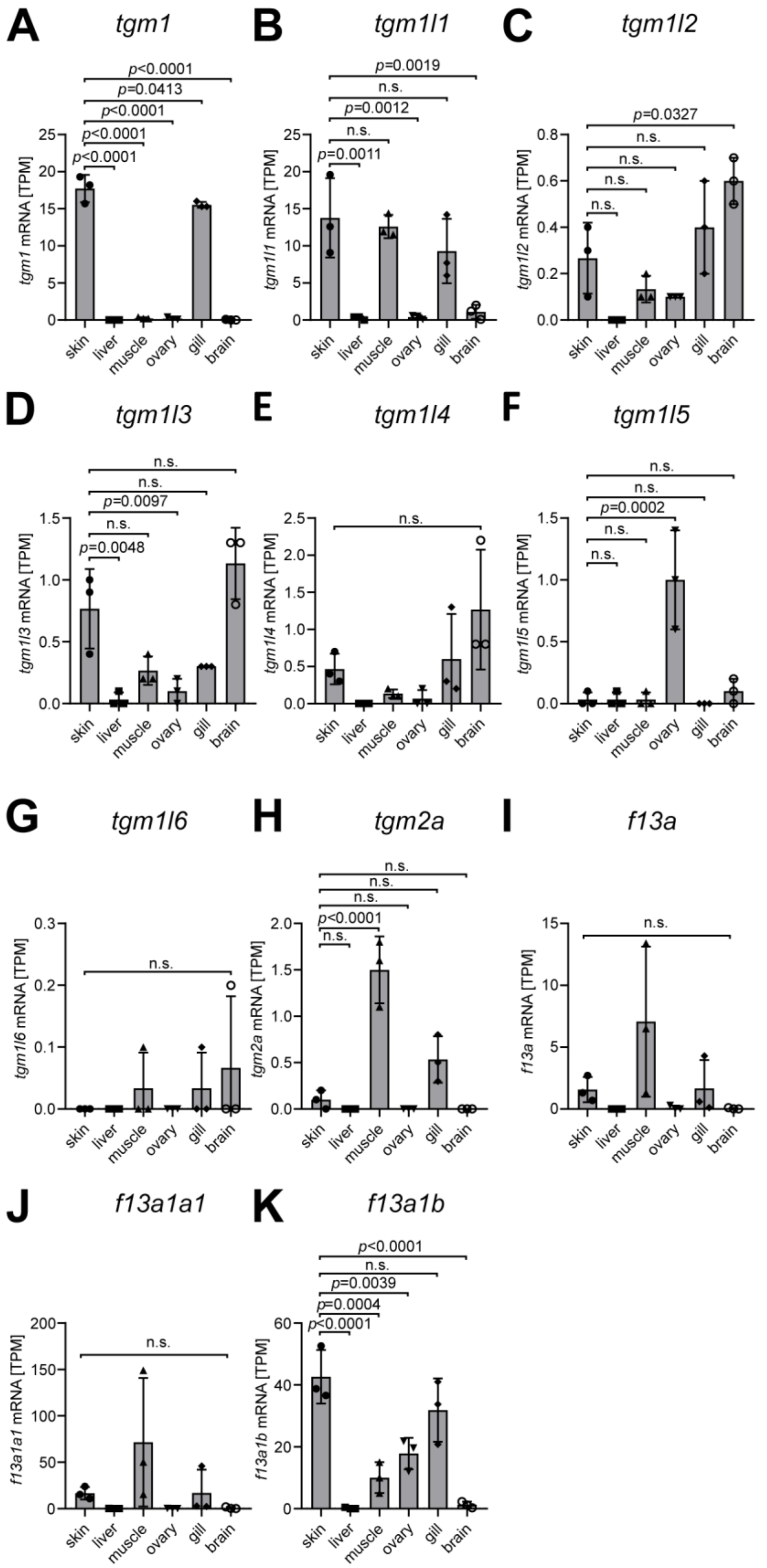

Figure S8. Expression of zebrafish TGM genes. TGM gene expression was studied by analysis of RNA-seq data (Table S8). Three biological replicates of skin, liver, muscle, ovary, gill and brain tissue were investigated for TGM expression. One-way ANOVA was performed to assess statistical significance. P-values of differences between skin and other tissues are shown. n.s., not significant; TPM, transcripts per million.

```
human        EPB42   PVYDGQAWVLAAVACTVLRCLGIPARVVTTFASAQGTGGRLLIDEYYNEEGLQNGEGQRGRIWIFQTSTECWMTRPALPQGYDGWQILHP
platypus     EPB42   PAHRSPDWVLAACTCSALRAVGIPARVVTAFGCARDTDGGWTVEEFYDDEGLEAAGAPESRIWASRVWTECWMARPDLSSGYGGWQVLDP
chicken      EPB42   PVRYGQCWVFAAVMCSVLRCLGIPTRVVTGFTWAHNTNSNPNVNEYYEEDGTLLTPDKSARVWTFHVWNECWMARTDLLPEYSGWQALDA
alligator    EPB42   PVRYGQCWVFAAVACSVLRCLGIPTRVVTSFTWAHNTGGHLSVDEYYSESGDKVACNGKASIWSFHAWNECWMARPDLPPGYDGWQVLDA
lizard       EPB42   PVRYGQSWTFAAVACSVLRSLGIPTRVVSAFAWAQGTEGSLHVDESFDESGATIPGDSDARIWLCHAWNECWMAREDLGQEYSGWQALDT
clawed frog  EPB42   TV-YGHHWVFAAVLCTVLRCLGIPTRLVTNYNSAYDTNRTLQKEIYYDEKGARIHRARSDSIWNFHVWNECWMERRDLSPEYNGWQVLDA
caecilian    EPB42   PVRYGQCWVFAAVMCTVLRCLGIPTRVVTNFTSAHDTGRKLTVDEYYNENGIKIKRNKNGSIWNFHVWNECWMAREDLPPGYDGWQALDA
human        TGM2    RVKYGQCWVFAAVACTVLRCLGIPTRVVTNYNSAHDQNSNLLIEYFRNEFGE-IQGDKSEMIWNFHCWVESWMTRPDLQPGYEGWQALDP
  catalytic triad:         C                                                          H                       D
```

Figure S9. Amino acid sequence alignment of the catalytic center of EPB42 in comparison to TGM2. The sequence of amino acid residues 292-381 of human EPB42 were aligned to the homologous segments of EPB42 of other species and human TGM2. Residues of the the catalytic triad (C, H, D) of TGMs are highlighted by red fonts and yellow shading. Substitutions are shaded grey. Species: Human (*Homo sapiens*), platypus (*Ornithorhynchus anatinus*), chicken (*Gallus gallus*), alligator (*Alligator mississippiensis*), lizard (*Anolis carolinensis*), clawed frog (*Xenopus tropicalis*), caecilian (*Rhinatrema bivittatum*). GenBank accession numbers are shown in Table S6 for human, platypus, chicken, lizard and frog. Caecilian EPB42: XP_029470562.1, alligator EPB42: XP_006271516.2.

```
>clawed_frog_EPB42
LPFVTCDLQVSK---NNKNHHTIDL--SKENRLFLRRGQEFTITLRFHSPTRLHYKQL----
EGITLTTKTG--PNPSKKNGTKNSFPISS-LSDNKFWSAKVVDAQQG--LWTLSITTPA-
SAIIGNYTLSLKSKKPTS------
LSQDLGRFMLLFNPWCKDDPVFLHNEGQRQEYVLNEDGIIYMGTESCIQQHPWHFGQFEYEIADVCIK
LLDMNPKYQKVPKNEYLNRNDPIYVSRVLGDMISRKDEEDRV--------
AFMVENRRPYGLISSVPILQKWFQNEF--KTVYGHHWVFAAVLCTVLRCLGI-
PTRLVTNYNSAYDTN-RTLQKEIYY-DEKGARIHRARSDSIWNFHVWNECWMERRDLSPE--
YNGWQVLDATAQLK-SSEAL--CVSGPAPMRAIKEGHVDLNYNVDLIFSKLVTDNMVWV---RN----
PKC---FSKVLCWARHVGDSISTKSVGSDMQEDLVHHYKYPKGSAEETEVLRIV-TQTMLETSLSP--
-ATVSISSQNLQMYGEDIHFKVTVANVSWEER-DFKMLVGAQPVHDHGFTQA-QF-WKEE-
FAFHFKPYEGCNLSIHLDHSSYE-ACLL---DNN-LLRITALVKD--PTCENDN-
ALAEQDMTICKPC-LSVQAPR-VSLQYQPMTAMIHFTNPSEKTLLECVLRASGKGLLHSER-QYRC-
GNVAPRGT-LRYPITFTPTQVGP-RRLYVQLECSIF-RNI-IGFHQFEV
>platypus_EPB42
LPVVRCDLHAST---NNPEHRTDGI--GARG-LVVRRGRPFQVTLTLSATQPP-CSPRI---
PTFTLVARTG-----AQAGGAEAVFPVSG-HADGTRWSAAVKERDPR--RWVLSVTSPA-
DAGVGRYSLLLQGSGKTR--------
RLLGRFALLFNPWARGDAVFLEGEAQREEYLLNPHGLIFLGTQGGGWPRPWDFGQ--
SDLVDFCLLLLDV--------DEEGRSRGDPVHVSRLLGAVVNGHTFPASPLAGSRP--
GTHPGSGFPASWAGSVPTLREWVARPP-GPAHRSPDWVLAACTCSALRAVGI-
PARVVTAFGCARDTD-GGWTVEEFY-DDEGLEAAGAPESRIWASRVWTECWMARPDLSSG--
YGGWQVLDPLATEG--GDAL--ACCDLTPVRAVKEGAVAVAPGVSRLFASLNSACAVWV---RS-G--
AGP---PSRASAGAEYLGNYISTKGVDGDRCEDVTRTYKYPEGSPREAEVLDGV-WKERDRSEPRA--
-LFVSLHAPSSFPLDGGAQLKVTVSNRSDEER-AVRLTLGAQVLYSTGRLGA-RL-WREE-
RHLTLEGNRDRTFTTGVEFRESG-RALE---NSS-SLRLTAVGVD----RSAGT-
CFAREDVTAYKAR-ATLEIPR-TAVQFQPITAVIGIHNDLASSLDNCEVAIAGRGLVHRER-IYRL-
GSVQPRGL-LRKQIRLTPTHPGV-LRLTARVSCGQF-RNL-VAFRSVNV
>human_EPB42
LGIKSCDFQAAR---NNEEHHTKAL--SSRR-LFVRRGQPFTIILYFRAPVRA-FLPAL---
KKVALTAQTG--EQPSKINRTQATFPISS-LGDRKWWSAVVEERDAQ--SWTISVTTPA-
DAVIGHYSLLLQVSGRKQ--------
LLLGQFTLLFNPWNREDAVFLKNEAQRMEYLLNQNGLIYLGTADCIQAESWDFGQFEGDVIDLSLRLL
SK--------DKQVEKWSQPVHVARVLGALLHFLK-EQRVLPTPQT--
QATQEGALLNKRRGSVPILRQWLTGRG-RPVYDGQAWVLAAVACTVLRCLGI-
PARVVTTFASAQGTG-GRLLIDEYY-NEEGLQNGEGQRGRIWIFQTSTECWMTRPALPQG--
YDGWQILHPSAPNG--GGVL--GSCDLVPVRAVKEGTLGLTPAVSDLFAAINASCVVWK---CC-E--
DGT---LELTDSNTKYVGNNISTKGVGSDRCEDITQNYKYPEGSLQEKEVLERV-EKEKMLETASP--
-LYLLLKAPSSLPLRGDAQISVTLVNHSEQEK-AVQLAIGVQAVHYNGVLAA-KL-WRKK-
LHLTLSANLEKIITIGLFFSNFE-RNPP---ENT-FLRLTAMATH----SESNL-
CFAQEDIAICRPH-LAIKMPE-KAEQYQPLTASVSLQNSLDAPMEDCVISILGRGLIHRER-SYRF-
RSVWPENT-MCAKFQFTPTHVGL-QRLTVEVDCNMF-QNL-TNYKSVTV
>chicken_EPB42
LSAKKCDLKIIM---NNNNHHTEEI--STER-LIVRRGQPFVITVNFSSPVHN-YLKQL---
KRIFLSVQTG--PQRSKADGTQVKFPISS-LGDQKQWSARLEEQDPY--FWTISVNTPA-
NAPIGQYDLFLHASKACR---------
LLGKFILLFNPWCRDDEVFLPNEAQRQEYILNQDGVIYSGTENEILAQPWDFRQFDVGIVDICFKLLD
IGERYQR--DQDHTQRKNPIYICRTVAAMMNCNL--ARILSESGT--
RECYDGTPPSKWLGSNAILQQWAALQC-RPVRYGQCWVFAAVMCSVLRCLGI-
PTRVVTGFTWAHNTN-SNPNVNEYY-EEDGTLLTPDKSARVWTFHVWNECWMARTDLLPE--
YSGWQALDATCQKK--SKGPSFC--GPAPVQAIKEGDVEVDYDVCYFFAAMNAKCKVWI---HM-A--
DDI---FKPASICTKYMGNNISTKSVGSERCEDITHNYKYPEGSLQEKKVLDKV-YRKTQTLEEKV--
-LFIDFQSKSSLILGQDIPLSIEVFNYSDREK-ATDLVLGVQSLHYNGVPIM-QL-WKEK-
LNFIIKSNEVRTLQVFVPYSQYG-KELG---KNR-LLRLTATLRD-----EDSY-
YFAQEEISICDPP-LTIEFPD-NVLLYQPATVKISLLNPLTEPLEKCVIVVGGQGLIYRQR-KYRL-
GTVQPKST-QDLNISFTPTESGP-RRLTAHLTCLQI-QNL-KSYKTINV
>lizard_EPB42
```

LQVERCDLRVAE---NHEAHRTGAL--SGGR-LVVRRGQPFALRLRLS---GPVSEKQR---
KSLLLVTRTG-----AAGGALEQAFPIGR---RGSDWEAVLEAHDDL--EWTLSVTPPA-
DAPIGLYALTLQLPSGTL---
APPSQHPLGHLALLFNPWSPGDSVFLGNEAQRQEYVLSEEGTIFWGLEEAPQGIPWDFGQFSEGFLDM
SLALLDIS-LLRMPAQDACDQLRSPLQVCRLVSAMLNGEK-PFPVLEGCWS--
GEYGNGTPPTKWPGSGPILRQWLSGRC-RPVRYGQSWTFAAVACSVLRSLGI-
PTRVVSAFAWAQGTE-GSLHVDESF-DESGATIPGDSDARIWLCHAWNECWMAREDLGQE--
YSGWQALDTTPKKGLQSGLL--CCGGPAPVRAIKEGRLDLPSGVGPFFAAINSTCTAWV---CK-A--
SGE---SEKATSEVKFLGNCISTKAMGSERCEDLTQHYKFPEGSLQERAVLMKV-CQDET---EET--
-LAAWFECESSLALGEDVQVSLRVANWTGKER-PLRVVLGAQPLQGNGETLA-QF-WKEE-
FMVTLPDGHEKVLSTTLPFVQYS-PALR---KGSLLLKLTALLREIPSDSDAGQ-
LLAWQEVLLRPPE-VTLQIPK-SLTQFQQTEATIQLHNHLPEPLTGCSITLSGRGLIYKER-SYRM-
GSVPPGGS-LRLRLPFTPTLAGD-RRLTARLDAPGF-QHH-HSCASVTR
>zebrafish_TGM5L
FKFVSVNLQPVQ---NQLQHRTDGL--SSGS-LVVRRGQPFTVTINYAG--RP-YDPLR---
EKLQFIFILG--PLS-------VEVPVSS-YGQPSSWSAVLPNPSGS-RALTVSLSSPA-
SSSIGVYTLQLTVQTRSS-----
TKTHLLGQFTLLANPWCQADSVFLQSEDLRNEYVRSDFGLLFKGTPGNVVSRPWSFGQYEKGLLDICM
KLLQLSPQSQTDMKRDLLNRSSPIYIGRVISAMVNCQD-DKGVLMGNWS--
GDYSDGVNPSVWSGSADILRMWSETQF-SPVKYAQCWVFAAVMCTVMRALGI-
PTRVVTNFNSAHDTN-GNMVIEEYY-TEMGEKL-SIGRDSIWNFHVWVESWMKRPDLGQT--
YDGWQVLDPTPQEE--SAGM--YRCGPAAVKAIHEQKVDAAYDVPFVYAEVNADVRTVI---IR----
DRK---ILGVSVDKERVGALICTKRPGAMTMLDITSEYK-TEMVLSSAYAFSAD-VRGGERVAPQG--
-LAVSLQLLKTPIIGESIAFSIIITNQASIPK-LLRAHANAQNKEYHRNPFG-SF-WEKH-
HELKIGPSETVTINQEISFNEYKLKQVL---EDY-LVNLAVVVED----VKSQE-
VLASEEFNIHSPA-LSIQIQN-EVIVNSPQVAMVTFTNPFDVAVTG-ELTVAGSGLLEEKI-QIRV--
SMQPREA-VRRPVRFIPQMTGA-KMLYACLVLINL-PTVLHGFKTLSV
>clawed_frog_TGM3L1
PELSGIDFDLLK---NVQEHRTQGI--RGMG-LVVRRGWPFTMKMQFR------
YLGQDAKLENLNLIAQIG--PYPSPHTGTHIYFPVSR-LRDNRSWSAEMEGENNG--VLAVKVQTPA-
TAIIGRYSLSLEGSYRGM-----
PVSHILGNVVLLFNPWCPEDDVFLNDEAMRQEYVMNEHGTMYQGTKDFIKNIPWNYGQFEDGVGETCL
RILDMSPSCLKDPATDCSKRGDPIFICRVVSAMINSND-DRGVIESSWD--
DLVRGGVEPSSWNGSVAILRQWHTSGC-RPVKYGQCWVFAGVLCTVMRFLGI-
PTRPVTNFESAHDTN-CTLTVDEYY-DETGKQLETL-GDTIWNFHVWNECWMARKDLRSG--
YDGWQIVDSTPQEI--SGGT--YCCGPAPVKAVKEGDMDVSYDVPFVYAEVNGDVIHWV---VN-E--
KGI----KQGQVDTIAIGKNFTTKRVGSNDKEDVTSHYKHAEGSPQERTVFAKA-TSAVKESGMAS--
-FEVSLKLAETPMIGQPIVLYCRILNKSFAAK-KLSVNMSAQAMQYNGTPLD-QF-WRKK-
FDVDVFPNQATDGQFPIHPLQYQ-QFIR---PGN-SIRVTVLATD----LQSKQ-
RFAAQNVVLKEPT-IDIKIYG-TPQFNRPLNVQLSFQNPFNEVLTNCVMSAEGAGLLPNGPAEIYM-
GNIYSLRA-GSVGLVCVPIKRGK-LQLEVSFSCNKV-QHI-KGSATIMV
>platypus_TGM9
IWVAHFDPNCSL---NCQSHHTDMM--SRDR-LILRRGQAFDVFLHFQN--RG-WDSSK---
DQITFTVETG--PSPCESSGTRTTFSLSE-TAPLHCWGAVCKASRHR--SIQVSLLIPA-
NACIGLYSLQAQVPSGQG-----
PGPRTLGEFIVLFNPWCPDDLVYLENPSHREEYILNEHGMVFRGLHKYIVSHPWHFGQFEENMVDMCL
RVLDMSGNFQRDPSLDCSYRNDPVYYVSRVVNSMLCSHS-SNSLMKLPRN--
NDSAQGVNPLAWNGSVPILSQWYGSGC-RPVRFGQCSTLAAVMCTVMRCLGI-
PSRVVTNFYSTQNAS-EAFIIDEYF-DSTGRSLCG--KEHIWRHHCWNESWMVRKDLNES--
CGEWQYLDPTPMET--SGGL--VCCGPTCVKNIREGDLDQDYDGAYVFSRLNAGRASWL---RQAS--
EGK----AKVHCDARLFGQSISTKGVGTEEREDITHNYKHQPDSIRGREVFYKA-YRRIHPGLDEA--
-TVMKFKLGNSPVYGEDINLFLHLANLSHESR-DLRLKLSAEGLTYAGCFME-PF-WTDD-
LIISLKPKAEKKVPLQIMYSQYG-RHLG---DHN-LLRVVAVSEP----GCKGE-
MLVDRDILLLKPP-VEIKLRG-SPRLNVRCTAEIIFTNPLPESLRNCKLTLEGSNLMQKPV-TIEL-
GTLAPRHQ-TQTLVDLIPFRPGL-HRLLANFDCHRF-SYC-KGYANVRV
>chicken_TGM9

```
IELGDVNLNCPS---NCQIHNTHFF--GTDR-QIIRRGQAFNFYASFHN--RE-WDDSV---
DQATFTVETG--LRPCESNETKCTFPMGR-CLDQTCWSASYKTHQPK--CINISVFPPS-
NACIGRYILNMQITSCGH-----
TYQRCLGDFYVLFNPWCADDPVYLDNQAQREEYILNEHGILYEGVHKHITSRPWHFGQFEDGILDICL
KILDMGASYHHGSDRDHCWRNDPVHVSMVVNHMISSHT-TNSIMKIPEN--
NDYLKGTKPFSWNGSVPILQQWYNGRC-RPVRYGYCGSLASVMCTVMRCLGI-
PSRVVTNFCFPCSNE-NPLGINEIF-DCTGKNLCG--KDKLWRYHCWNESWMARRDLNQC--
CGDWQCLDPTPLET--GRGS--ACSGPTWVRSIREGDLDLDYDGHSMFSRLNSNYAGWL---SQ-N--
NAK---KTKVFCDAWPCGQQLITKSVGSEQFEDITGNYKYELGSVKSKEAYYRA-YRRIHPFLSDS--
-INTRLKMANCPMYGEDVQLHWLLENLRSEPK-NLKFNLCAQIITYNGCPMD-QF-WKDS-
VTVALGPREVKKIPLCVSYSQYG-PYLS---DHN-IMKVVAVSDP-----ECGE-
LMVSRDIVINRPP-VIVKLLS-QPRLKVPCTAEISFCNPLQEDMKNCIMTLEGCGLFKEPM-TIDL-
GTLASNQQ-ARTIVEFTPYRLGS-HRLLANLGCHKF-AYC-KGCAKAEV
>lizard_TGM9
LEVKNVDLNCAE---NSISHSTHLF--NVNK-LIVRRGQPFGFYLHFQN--RE-WDDEN---
DKITFTVETG--PKPCESLGTKSIFPLGG-CPDHNHWNASFKLQNER--CLCVDIFSPA-
NACIGCYSLTMCLVSCGH-----
KHSQNVGDFYMLFNPWCSDDAVYLDSQAHRGEYVLNEDGIIFKGLPKHITTHPWHFGQFQDDILDICL
KILDTNTNFLRDPGMDCSCRNDPVYISRVLNTMIGCHK-KKSVLKLPSN--
NNYLQGPNPSLWNGSVPILRQWYHSKC-KPIRYGDCGTFASVLCTVMRCLGI-
PSRVITGYYCPLNTV-NPLVVQEVF-DYTGKNING--KEHVWIYHCWNESCMIRKDLNQS--
VSEWQYLDPTPVET--SKGS--VCSGPIWVKSIKDGDVDTDSDGHRVFCMLNTRNTAWV---SQ-G--
RGK---KTKLYCDTWPCGQCISTKGVGRDMREDITDVYKHELGSLQEKKAFYKA-CKKINRSLKDI--
-VIMKFKMANCPVYGQNVQINWVLENLCDEIK-DRKFNLSAQGMMQNGSSLD-QL-WKEN-
MHVTLGPKEVKKILIDIPYDSYS-SHLC---DDN-IMRVAAVSMP----EPKGE-
MMVERDILINSLP-IDIKILD-HPKLNVPCCTEITFSNPLKEDLKNCVLHLEGFGLIGEPI-TTEL-
GTLDAGHK-AQTCVEFTPCRYGK-HQFVASISCHKF-CNC-KGYSNVDM
>clawed_frog_TGM3L2
FKVLSADLQLDA---NKSAHNTSAY--ISRE-LIVRRAKAFGIGLNFN---TS-VNAE----
DRLQFTAGLI--TSSASVSLLEFNFSDSR-YPAINSWGAQRVATGRN--SVTVNFFTPP-
DAVIGRYFLSLLTSEGRT---------
RFASFVLLFNPWAQADVVYLSDDAERQEYVLSEFGLIYLASNPN--
PIAWNFGQFQENILNVSLALLDSTSSFRSNAAEDVRKRNNPVHVCRVLSSILNSMN-NNGVIEGNWS-
-GEYFDGTDPYVWNGSTEILRSWYGQR--QPVKYGQCWVFAGTSCTVSRSLGL-
PCRVITNYQSAHDTD-HNLSIEQYF-NTRGEAVPRS-EDSIWNFHCWNESWFLRLDLGDF--
YSGWQVWDSTPQEK--SDGL--YQLGPTSQRAVKEGEVDLLFDTPFVLAEVDADVIIYI---VQ-D--
DGT---VTKAGKRENDVGQLICTKAVGQKAMNDVTLEYKYKDGSSKERETYEKA-RSKIRSSPPPP--
-VTGTITVAGTPTVGDDINVTLTLKNMTSDNK-TVTVNLSAAAIVYNHAVRK-PIL-SNS-
AAVQLGPNEAKGVPVQIKYSQYE-KQLT---SDK-MIYVTAVYKV----EGKPK--
FVEANIVLLNPT-LQVKALK-TAIFDSPMVVEVTFKNPLSTPITGCVLKVEGSGLTKDVI-EKNM-
GQLEPGQK-TSVKVEFIPYMVGD-KELLVNLACDKF-KDI-KGHLSIKV
>zebrafish_TGM8
DQGVRVDLECVK---NNTDHHTHEI--TQER-LIIRRGQAFSLKISAENI-QQ---------
NHISITAETG--PGVSEVKHTLFSFSTQS-----STNPAQVCSRSES--SVSLALVFPS-
DAPVGRYSVCVKHGSSSC----------
MNTLTLLFNPWCKDDHVYLPSEAERQEYIMSEQGVLYKGVDEYITSMNWDYGQFEEDIVDICLKLLDL
NPKCLKDPQEDYSARCNPVYVSRVVSAMINCND-DQGVLAGQWG--
DSYTGGVPPSRWSSSVEILRRWVKYNC-SPVKYGQCWVFAAVMCTVLRCLGI-
PCRVVTNYQSAHDTD-RNLVVDEYF-SDYGVRPKNS-
QDSVWNFHVWVEAWMRRPDLTEDTLYDGWQVLDPTPQEK--SSGT--
YCCGPAPVMAILEGHTEVKYDVPFVFAEVNADRVCWL---LM-T--DGS---
RKKIMSDSRSVGQNLSTKAVGSSSRLDITDLYKHGEGSAQERAVYTEA----VHESPPVS---
VQMRLSLDVPPLNGADVPLKLFLKGSRAQ---CVCVNISAQVMRYTGAPAA--AVWSHS-
TDVQLQDTEEKALSFTLPYSAYG-HRML---ENN-CIKVSAIARE----
KNNPKDYLTEKNIILHTPN-LSITVSG-SPLLDSEMTATVQFENPLSSTLQNCSISLSGSGLLKSTE-
KSST-VQLGPGQR-IRLQVSFTPYRLGL-KKLMATFNSATF-KDV-QASADVDV
>clawed_frog_TGM3L
```

```
PQVIACDWYYAV---NEKAHHTDVY--ESDD-LVLRRGQPFKLTLMLN---RP-LQAE----
ENILFIFETG--PSPSETSKTKVVFPLFR-AENLQSWGAILTSIKST--SITVTINSHS-
DAVIGRYILSVAVQCRGS---
DKPLPHQIGALNLLFNPWLQGDAVFMAEEDQRQEYVMNEQGVIFQGSDEDITSINWEYNQFERNILDI
CLVILDRSQNYKKDPALDVSQRNDPLYVCRVLSAMLNSKG-DDGVLEENWS--
DDYINGASPSSWNGSMNILKSWYYSKF-KPVKYGQCWVYAGMLCTVLRSLGI-
PTRVITHFNSGQDKN-ANLFIDLQY-KSQGSRQNDQ-EDQLWNFHVWNEAYFKRRDLGKS--
YNGWQVIDSTPLKR--SDGV--YQCGPAPLTAIKEGDINLNYDVKYMFASVNADVACWI---YY-R--
NGT---KKQVSNNAKETGKFISTKAIGSNDRVDVTNNYKYAKGSEKEIKVFEKA-LKLSKEKPRGG--
ILFGRCTLDQIATFGQDFNLILSLKNLTPDSI-NVTVYINTSAILYTGRQRH-TI-WTGG-
KFLSLGPNLEKRFSIPVKYGQYS-KHMT---DNG-VICMTALCEV----EGTEE-
ILVERNVSLVKPP-LSITLPD-KAIINQESNANIVIVNPLLETLNSCILWVEGYGLTDKIL-KKEV-
PSLKPGQS-SEHNFVITPMKTGV-RTLLVNFSCDKI-QNM-KGSGKILI
>lamprey_TGM10
VNRWLCKLQGGG---GGGLASSPSK--SRSS-LLARRGRPFIIDVHLGDSYR--FEPDR---
DELSLSLRLG--PNPQESDRTLILLSSAN--GDGGGWSLDVEEAAGG--RLRLSVRSPA-
DAIVGEYLLSLELIVGVE--
PLQITGFTVGKVWMVFNPWCPGDAVYMEDEIGVNEYVLNEKGLIYMGSNDYIYSIPWDFSQFADRVVE
ICFQILDNSNSALDDLEKDVPRRADPIHVSRVVSAMINSND-DRGVLNGRWN--
EHYTDGTNPMHWAGSANILRQWSDSGF-EPVRYGQCWVFAATACTVLRALGI-
PSRCVTNYLSAHDTD-GNLACDRFFREEDLQQVLKGRNDSVWNFHCWVESWMARPDLPPG--
NDGWQVIDPTPQER--SNGV--FCVGPAPVVAVRDGEVRHPYEAAFVFAEVNADVVNWV---VT-A--
GGE---KRRANCFTGFVGQNISTKHVGSDEREDITHLYKHPEGSAEEREAFERA--GRGAR-------
-LEALIKLSASAFVGSNVDAHTVVRNRSGRDL-TLTLTSAAAALTYTGQCGA-ECG-ARS-
CSVTVPAGQTARETLRVKYRDYG-EHLT---DQN-LLRVTSLLEE----PSSGQ-
VIMERNITLKRPR-ITVTIVG-EPRQNRKIKARISFTNPLPSKLIHSVFSIDGEGLTSLQK-
IPEPAAEIGPGET-LTVTAELAPSRPGT-FLLSVVFNSDRI-RDA-HESKRVIV
>lamprey_TGM10L
LELESFERLEVA---NQKAHHLEALTVEKDR-LFLRRGQPFQLLVKFRG--RL-FNPGN---
DAIALALHTG--PSPSQQDGTLLRASSDS-AGSGHTWSL-------------LEASTPA-
GAPIGRYTAFLETRLAKG-
GDSTVRCFPLGEIVLLFNVWCKDDAVYMEDDLLRSEYVLNETGKIYVGSHDYISSVPWNFSQFAQGLG
DICFQILDKSNMALEDLAADTKKRGDPVHVSRVLSAMINCND-DKGVLQGNWS--
GKYPNGTCPTSWTGSADILRCWHKTQC-QAVKYGQCWVFAGVACTVMRFFGL-
PTRCVTNFTSAHDKD-GNLSCDRFYYESNNKQES---HDSVWNFHVWVESWMSRKDIEEG--
YDGWQVIDPTPQER--SEGM--FCCGPAPVRAVRKGQVSLGYETPFVFAEVNADVVMWS---VA-A--
DGR---RTRMSCNTDHVGRCISTKRAGCDEREDITLQYKYPEGSKEERAAYARA-TCRGEKYSRPL--
-LLVRTRLSRSAFVGSDMDVHTSLRNPSRRHAAVVRLVLCARAVTYTGMVRE-ICT-QRE-
CTVEVPPGETVSEPLRIKYKDYG-SQLR---DQN-IIRVTCAVTL----
PGQETIAFSERNIILETPE-IHVMVLG-EPVVGRELKVEMSLVNPLPQPLDDGVFSLEGPGLTSLQQ-
ISCS-DRVEPEKN-VCVSASFCPRKPGL-HNLVITFNSNRL-RNV-HGEARVIV
>human_TGM7
LRLESVDLQSSR---NNKEHHTQEM--GVKR-LTVRRGQPFYLRLSFS---RP-FQSQN---
DHITFVAETG--PKPSELLGTRATFFLTR-VQPGNVWSASDFTIDSN--SLQVSLFTPA-
NAVIGHYTLKIEISQGQG----
HSVTYPLGTFILLFNPWSPEDDVYLPSEILLQEYIMRDYGFVYKGHERFITSWPWNYGQFEEDIIDIC
FEILNKSLYHLKNPAKDCSQRNDVVYVCRVVSAMINSND-DNGVLQGNWG--
EDYSKGVSPLEWKGSVAILQQWSARGG-QPVKYGQCWVFASVMCTVMRCLGV-
PTRVVSNFRSAHNVD-RNLTIDTYY-DRNAEMLSTQKRDKIWNFHVWNECWMIRKDLPPG--
YNGWQVLDPTPQQT--SSGL--FCCGPASVKAIREGDVHLAYDTPFVYAEVNADEVIWL---LG----
DGQ--AQEILAHNTSSIGKEISTKMVGSDQRQSITSSYKYPEGSPEERAVFMKA-SRKMLGLRDQP--
-AQLQLHLARIPEWGQDLQLLLRIQRVPDSTH-PLVVRFCAQALLHGGGTQK-PF-WRHT-
VRMNLDFGKETQWPLLLPYSNYR-NKLT---DEK-LIRVSGIAEV----EETGR-
MLVLKDICLEPPH-LSIEVSE-RAEVGKALRVHVTLTNTLMVALSSCTMVLEGSGLINGQI-AKDL-
GTLVAGHT-LQIQLDLYPTKAGP-RQLQVLISSNEV-KEI-KGYKDIFV
>platypus_TGM7
```

LG----------------------------R-LVVRRGQPFSITLHFGN--RG-FQPET---
DRLVFVAETG--PRPKRELGTQTRISLSP-PDSSGTWGVAWLPETTR--SLGVSLAPPA-
DAAIGRYGLKVEISGSRR-----
VRSHWLGEFILLFNPWSPEDEVFLPSEPQLHEFLLRDHGLIYKGHENWITPSPWNFGQFEGDIVDICL
EILDRNLSFLLEPELDCSRRGSVVYVCRVVSAMINSND-DSGVLQGNWG--
EDYRDGVSPSEWNGSVAILRQWHAAKG-QPVKYGQCWVFAAVMCTVMRCLGV-
PTRVVTNFCSAHSGD-RNLTIDVFY-NDAAEMLPGESRDRIWNFHVWNECWMARRDLPPG--
YGGWQVLDPTPQET--SKGV--FCCGPASVKAVKEGDIQLPYDTPFVFCEVSGDEVTWL---QE----
AGE--VKEILSHKTHSIGKSISTKMVGAAERQDVTDDYKYPEGSLEERTVYLKA-TRLLMQTTPGP--
-VRLKLGLAGSPKWGQAVALQLRASRSWAALP-ELGLRFSAQAVLHVGGVQA-PL-WKGK-
AVLRLAPGEEKDLPVILPYDNYK-ERLL---AEK-VVRISCIAHV----ELTGR-
LLVVKVISLDIPQ-LTIKVGG-PAVVGQRLDVSIHFTNTQPEALTGCSLALEGSGLVEGLV-SAAL-
GTLEPGSS-TVVELAIVPLKAGP-RQLQALITSNEI-KEI-KGFEGVSV
>clawed_frog_TGM5
AEIAYSDFQIKR---NNKAHHTDET--GDRR-LFIRRGQPFFITVHFKT--RG-FQPGQ---
DNVIFLVETD--

DDVFMGNEAERQEYVINDYGFVYQGNHNWIIPCAWNFGQFEDDIVDISLKILDKNLNYIQDSFKDLKN
RNNPVYISRVICAMINSND-DNGVLQGNWS--GDYTNGVSPSAWNGSILILRQWYKSDC-
QPVKYGQCWVFAAVMCTVMRCLGI-PARVVTNFDSAHDTD-QNLLIDEYY-
DTTGKKLSKETKDSIWNFHVWCECWMARRDLPPG--YGGWQVLDPTPQEA--SNGI--
FVCGPASVKAIREGDIHLNYDAPFVFAMVNADCVSWI---VS-T--KGK----
EKYHCDPHLVGTQISTKRAGSDEREDLTHQYKYEEGTAEERKAFEKA-ISRLHSPLREA---
LLLKFKLTESPQLGETISLVLLAGNMVSTAK-TLKLSLSAQAMKHDGKPAQ-QF-WRDS-
MYVELGPHEAQTLPCKIPYAKYG-PSLE---DNH-LIRFIAVGEQ----NITWE-
VLVEKDVNLALPA-VVINFLE-LAVVDKTCKAELSFSNPINEAIHDCQILIQGSGLLKKQV-KLNM-
GTMKPGQK-AVVAFEFVPWKVGY-KQLQVNISSNRF-KGL-KGFKSVVV
>human_TGM5
LEVALTDLQSSR---NNVRHHTEEI--TVDH-LLVRRGQAFNLTLYFRN--RS-FQPGL---
DNIIFVVETG--PLPDLALGTRAVFSLAR-HHSPSPWIAWLETNGAT--STEVSLCAPP-
TAAVGRYLLKIHIDSFQG----
SVTAYQLGEFILLFNPWCPEDAVYLDSEPQRQEYVMNDYGFIYQGSKNWIRPCPWNYGQFEDKIIDIC
LKLLDKSLHFQTDPATDCALRGSPVYVSRVVCAMINSND-DNGVLNGNWS--
ENYTDGANPAEWTGSVAILKQWNATGC-QPVRYGQCWVFAAVMCTVMRCLGI-
PTRVITNFDSGHDTD-GNLIIDEYY-DNTGRILGNKKKDTIWNFHVWNECWMARKDLPPA--
YGGWQVLDATPQEM--SNGV--YCCGPASVRAIKEGEVDLNYDTPFVFSMVNADCMSWL---VQ----
GGK---EQKLHQDTSSVGNFISTKSIQSDERDDITENYKYEEGSLQERQVFLKA-LQKLKRPSDVV--
-VSLKFKLLDPPNMGQDICFVLLALNMSSQFK-DLKVNLSAQSLLHDGSPLS-PF-WQDT-
AFITLSPKEAKTYPCKISYSQYS-QYLS---TDK-LIRISALGEE----KSSPE-
ILVNKIITLSYPS-ITINVLG-AAVVNQPLSIQVIFSNPLSEQVEDCVLTVEGSGLFKKQQ-KVFL-
GVLKPQHQ-ASIILETVPFKSGQ-RQIQANMRSNKF-KDI-KGYRNVYV
>platypus_TGM5L
LEVILTDFQHSR---NSAEHHTEEM--GPGR-LVVRRGQPFSITLHFGN--RG-FRPDA---
DRLVFIADTG--LKPESLPGTRAVFGLGE-PGSPGAWTAAVEAGNSR--ALEISLCPPA-
TAAVGRFCLKIHIETTGG----
PVGAYRLGTFILLFNPWCPEDDVYLSSEPQRQEYIMNDYGFIYQGNKNWICPVPWNYGQFDEEILDIC
LTLLDKSLNFQADPVRDFALRGNSVYVSRVVCAMINGND-DGGVLQGNWG--
EDYRDGVSPSEWNGSVAILRQWHAAGG-QPVRYGQCWVFAAVMCTVMRCLGI-
PTRVVTNFDSGHDTD-RNLIIDEYY-DPMGRILEDKKKDSVWNFHVWNECWMARWDLPPG--
HGGWQLLDATPQET--SDGL--YCCGPASVAAIKEGEVDLPYDTPFAFSMVNADRTAWL---VH----
RGR---ERKLHQDAHSVGNFLSTKGARSDEREDVTESYKYGEGTV-----------------------
----
-----LPCTIPYLRYR-GHLS---PDK-LIRLSALGEE----RNGSK-LLVNKIITLALPG-
ITIDVLG-PTVVGRPVSVEVRFANPLEEPAGDCVLTLEGGGLFRRQV-GVTI-GTLGPLRG-
ASVKIQLVPFKSGR-RQIQANLRSDAF-KDV-KGYKSLEV
>clawed_frog_TGM3L8

```
LQLKSINLQKAT---NVANHHTSGF--DSTV-FALRRGQPITVNLNFN---RV-FYTG----
ESVAIDVETG--PSPSVPKNTKAVMPVSK-YANPQTWSASSGQSTGA--TMPVIINIPV-
NAIPGRYKMTAHLARSGR----
TTASYRLPEFFVLFNPWNQDDQVYMSNEAERNEYVLNEYGVIFRGNANNISRLAWDYAQFEGDMLDIC
FLMLDSCLEHKTDPITDISNRYDPIYVGRVLSAIVNIND-DNGVLYGRWD--
NDYSDGVSPTSWSGSARILRSWRDNG---PVKYGQCWVFAGVLCTVLRCLGI-
PARVISNFESAHDGN-SNLLIEEYF-DEKGTQLGS--PDSVWNFHCWNEAWFTRGDLGDN--
YGGWQILDSTPQEP--SGGI--YCLGPTSLKAVKQGDVTLDYDTTFVFGEVNSDRKRFI---KY-S--
DGR--PLQNVYTDTASVGQFISTKAVGSNNRQDVTNGYKDPEGSGKERETFNKA-RTTLLPKGPKP--
-ITGSFKLSGQPQIGDDVVVIFNIKNPTSARK-NIKLKFTVTAIVYNRAVSK-EIL-TNN-
QPVTLAANKEESVNLTVEYSKYQ-GALT---PDN-MIQVVAVCEE-----ENGA-
LLTDTVITLKNPP-MQLKVPD-KAAVNKALSVEVIFQNTIGDRLKNCLITLEGSGLIKDEV-KVPV-
PDLKPSEK-YSTKVVITPYKSGE-RNLSANLSADKL-SDV-KANLTVKV
>clawed_frog_TGM3L7
LVLKSIDLQQKS---NAAAHNTSDY--VSKV-PIVRRGLPMKVTLAFS---RA-LQST----
ESLALVMETG--PSPSASTNTRIVMPVSS-SRRNDTWSATLESSSAG--TMTVSINIPV-
NASIGPYKITAQITSAGR-----
TSTNDVGRCSVLFNAWASGDEAYMSNDAERKEYVLNETGLVFYGSAGFPSSSAWDYGHFEDGVVDICF
KLLQESPEYKADPATHISKCNDPVYISRILSAMVNSQD-DRGVIVGNWS--
GDYSGGENPSSWSGSVALLQRWSQSG---PVRYGQCWVYAGVLCTTLRCLGL-
AARLITNFESAHDTN-NNLIIEQYL-DAYGRSIGS--PDSVWNFHAWVECWFRRKDLGST--
YDGWQILDATPQEP--SGGS--YRLGPTSQKAVKLGDVNLPFDGPFVLAEVNADEIYYG---RR-N--
DGT---FSILYTDTRKVGQYISTKAVGNFSRQDVTDQYKFPEGSKEERDSLHKA-QGLTAPIQEAP--
-IIGTFKLSGELQVGQDFTVTLNLKNPTSSKK-QVNAKWTLTAIVYNRTPVK-EIL-SDS-
QSVTLAPNEEKVIEITVLYPQYE-KALT---PDN-MIRASAVCVE-----ENGG-
LLVDKVITLKNPP-LQITAPE-RVTWGKSATLGIVFTNPISEDIKNCVVTLQGSGILKKTL-KVPL-
PDLKANQR-IRAEAEVTPYRNGK-RVVIAGFSCDKF-SDV-KGYQTVDV
>clawed_frog_TGM3L9
LQLKSVDLQQKS---NAAAHNTSDF--ITKS-LIVRRGQTFTIQLDCS---RD-LKDG----
ERLDLCVETG--PSPTKDNNTQAVMQVSS-SGTSSSWSATLGSRSGG--TLQVIISIPV-
NAVIGRYQMTAQLTASTT------
SSFPVGDFIVLFNPWASGDEVYMSNDEERREYVLNETGFIFLSS----
GRITWDYGQFEEDILDICLLLLDRSTEYRRNPVSAVAKRYNPIYVGRVLSAMVNSND-
DNGVLVGNWT--ADMSGGEAPSSWSGSGTILRRWSQNG---PVKFGQCWVFAGVLCTVLRCLGL-
PARVITNIESAHDTN-RNLVIEEYY-NEDGTNIPS--PDSVWNFHAWNEVWFVRKDLGST--
YDGWQILDATPQEL--SEGI--YCLGPTSQRAVKLGDVNLNFDGVFVFSEVNADKKTYV---KY-K--
DGR---TVLVHTDTTSVGQTISTKAVGSSSRVDVTNDYKHTEGSSEEREVYFKA-QRQVAAYAPKS--
-IIVTLKMSAQPQVGQDVTAMLNLKNASSGHR-KVKVNWTATVIVYNRTPVK-EIL-KES-
QAVSLTVNEEKAIPLKILYSQYK-DTIT---TNN-MIHIVAVCHE-----EKGG-
LLVNKIIILKHPP-LELKVPF-----------------------------------------------
-----------------------------------------------IL
>clawed_frog_TGM3L5
----------SS---NMAEHRTNYY--SSSD-LILRRAQAFRIMLYFN---RP-LREK----
DKVEFTAATG--PDPQEADDTMCIFPLFG-SQSKASWTAEVDSIDSN--CVTAIITSSA-
DAVIGRYKLQLYTTSSKK-----
KSYFKLREFVLLFNPWAEDDVVYMEDENERCEYVLNDHGIIYFGHEEMIDEQGWDFGQFEENILDISL
QILDRSLNYQDDPVLDCSQRYDPGYVGRVLTAMINSFD-DDGVLEGRWT--
GKFTGGVDPQHWIGSVEILMRWYRGGY-KPVKYGQCWVFAAVMCTVLRCLGI-
PTRVITNFASAHDKD-GNLGIDSIY-SSSGRNMS---KDTMWNFHVWNESWFRRNDLGSA--
YRGWQVLDATPQEL--SEGT--YCCGPASVHAVKEGDVDKDYNVPFVFAEVNADRNTWV---YY-A--
KDV---KEKVYTDSKSVGKHMSTKSVGGNERVEITNNYKYPEGTEKERQVYLKA-RKKLLDSEEPT--
-IIGKFQLVAPPKFGDDVNLILSLRNSGQKSE-ALKVKLSSSAIKYTGRPMS-EIF-SDQ-
TSVTLGSMKEKQIPINIAASQYE-EELT---KDH-LIEVVALCEL-----KSKK-
MLVRRVVSIEKPP-LLIQVLS-YPVVDELCELQVSFKNPLSVPLTDGILLLGGSGLIRKQI-KRRV-
PKLGPKAE-GSIVLEITPYRFGT-QQLVVDFISKHF-SAI-KGFKRIEV
>clawed_frog_TGM3L4
```

----------ES---NRKGHHTEMY--NSPE-LILRRGQSFWITLDFD---RP-IQEW----
ESIVFTAQTG--PLNAKFYNINVEFPLSN-SWSSGRWSAVLESAPGN--SLRIIMSSPA-
NAVIGRYNLTVQICIMGN-----
TSTYSLGKFILLFNPWCLDDEVYMANEDERNEYILNDNGIIFIGSDKHIASLAWNFGQFESNILNICL
DMLDRSLNYRNDPAADCSKRNSPMYVGRVISAMINSND-DYGVLEGKWE--
KEFSDGVDPNSWTGSVEILLKWQKEAY-QPVKYGQCWVFAAVMCTALRCLGI-
PTRVITNFNSAHNTD-GNLCVDLHY-DNDGKFM-EISDDSIWNFHVWDESWFLRKDLGQF--
YGGWQVLDSTPQEQ--SQNV--YRCGPTSVNAVKEGDVHLPYDTPFVYSEVNADRVTWV---CH-K--
DGR---KEQAHSDTKAVGQFISTKAVGSNERVDITHCYKYPEGSPKEREVFDKA-NRKLSPSLDGP--
-IAGKFKLLGPLAVGDDINLLLSLRNLTPYHR-QVIVNLSASCILYTGRRIN-DIF-QDQ-
KSLIINPSQEGHISLQIPYSLYG-NFLT---DGN-MIQMVAVCEL-----PFRK-
VVITKELVLDNPP-ISIKPLE-MAVLNRKMIVEVRFTNPLSFVVKDCTVAVEGAGLIDRQL-TAVV-
PYIKPKQN-IKFKVELTPFRSGT-KQVIVHVKCRYF--SI-KGHHLLNV
>human_TGM3
LGVQSINWQTAF---NRQAHHTDKF--SSQE-LILRRGQNFQVLMIMN---KG-LGSN----
ERLEFIVSTG--PYPSESAMTKAVFPLSN--GSSGGWSAVLQASNGN--TLTISISSPA-
SAPIGRYTMALQIFSQGG-----
ISSVKLGTFILLFNPWLNVDSVFMGNHAEREEYVQEDAGIIFVGSTNRIGMIGWNFGQFEEDILSICL
SILDRSLNFRRDAATDVASRNDPKYVGRVLSAMINSND-DNGVLAGNWS--
GTYTGGRDPRSWNGSVEILKNWKKSGF-SPVRYGQCWVFAGTLNTALRSLGI-
PSRVITNFNSAHDTD-RNLSVDVYY-DPMGNPLDKG-SDSVWNFHVWNEGWFVRSDLGPS--
YGGWQVLDATPQER--SQGV--FQCGPASVIGVREGDVQLNFDMPFIFAEVNADRITWL---YDNT--
TGK---QWKNSVNSHTIGRYISTKAVGSNARMDVTDKYKYPEGSDQERQVFQKA-LGKLKTEEQEP--
-IIGKLKVAGMLAVGKEVNLVLLLKNLSRDTK-TVTVNMTAWTIIYNGTLVH-EV-WKDS-
ATMSLDPEEEAEHPIKISYAQYE-KYLK---SDN-MIRITAVCKV----PDESE-
VVVERDIILDNPT-LTLEVLN-EARVRKPVNVQMLFSNPLDEPVRDCVLMVEGSGLLLGNL-KIDV-
PTLGPKEG-SRVRFDILPSRSGT-KQLLADFSCNKF-PAI-KAMLSIDV
>platypus_TGM3
LQPTNVSWREEA---NKRSHRTSKF--YSSE-LIVRRGQPFVIALGLN---RA-VAAG----
ETLTFTAVTG--PSPSESARTKAVFQLSG-PVARGGWSAVPEARDGS--GLTIAVTSPA-
NSPIGQYKLSVQISSRGK-----
VSSTVLGTFIQLFNPWLQADAVFLNSEAEREEYVLRDAGIIYVGSVNRIGMVGWNYGQFEDDVLNICL
SILDRSLNYQRDPTTDVARRNDPKYIGRVLSAMVNGND-DAGVLAGNWS--
GEYTGGRDPRNWNGSVEILKEWKKTGY-RPVRYGQCWVFAGALNTVLRCLGI-
PARVVTNFNSAHDTD-RNLTVDVYY-DPTGNPLDRG-SDSVWNFHVWNEAWFLRTDLGPA--
YGGWQVLDATPQER--SQGI--FQCGPSSVAAIRAGEVQLDFDGPFVFAEVNADRVTWV---YDRS--
NGT---QRQNWLDAYSVGRFISTKAVGVNARLDLTERYKFPEGSAQERAVFKKA-LSQLRGRGQAA--
-VSGKFKVIGSLEVGQEVNVVLLLQNLTRDLK-TVTVNRTAWTIVYNGTLVR-EI-WRDS-
ITASLEPEEEIELPIKLAYAQYD-GHLT---ADN-MIRVTAVCQV----TDGGE-
VVVERDVILDNPT-ITLEVPD-HAKVKTPMNVLVLFANPLAEPVENCVLMVEGSGLMRGNL-KIDV-
PSLRPKQR-SRVQFEIVPIRSGA-NQLLIDFSCNKF-PAI-KAFVNINV
>lizard_TGM3L1
-MTAKIDWKLKD---NGIAHRTDRY--SGSE-LAVRRGQPFGVSLTYGG--NP---PAV---
SSLTFTVETG--STAALQTKTRVAFGVTG-SPPNNNWGAVQTAPAPG--TMSFSIFSPV-
NAGIGRYRFGIRTGGSSA------
PSSLLGTFVLLFNPWLQGDEVFMPNNAEREEYVLSESGVVFMGSSNSISPRGWDFGQFQPDILDICLS
ILDRSLNHRRDAATDLRRRNDPKYVGRVLSAMVNSND-DNGVVLGNWS--
GNYSGGENPGSWSGSVRILQQWKSSGF-RPVRFGQCWVFAGVLTTVLRCFGF-
PARMISNFNSAHDTD-QSLTVDVYY-DPAGNPL-NMDSDSIWNFHVWNEAWFARSDLGST--
YNGWQILDATPQER--SSGI--FQCGPASLVAIKEGDVDLDYDCPFVYAETNADRVTWT---YDTA--
TGQ---KKKIYSETKSVGQFTSTKAVGSFARKDVTNDYKYPEGSTKERDVFNKA-RGKLNPDAPKP--
-VSGKFKVKSPPEVGKDVELVLLLTNLASAAR-TLTANMTAWSIVYTGKVIH-EV-WKDS-
LALTLGPKEEKAYPIKISYEEYQ-KHLT---TDN-MIRATAVCHF----KDGND-
AVVEQDIALENPT-ITLKVPG-QAKVGQAVKVEVVFTNPLAEVVSSCVLLAEGSDLLEKAI-RKEV-
STVKGKES-ARITFEITPKKKGT-KQLVTNFSCDKF-KDI-NTFQVIKV
>chicken_TGM3

AAQPSTDWHVKE---NGRDHHTSKF--SSKE-LIVRRGQAFVITF------NG-VEQPE---
QTLTFIVETG--PKPSKQAKTQATFGISS-TVSKDSWSAVLQSTSSH--SVSVSISSPP-
NAVIGRYKLSVQSTSTGS-----
SSPETLGTFVLLFNPWSSGDNVFMPNKAECEEYVLEEFGIIFAGNNYHINSFGWNFGQFQADILNICL
SMLDRSLNYRQDPATDVSHRHDPKYLGRVLSAMVNAND-DQGVVLGNWS--
GNYDGGKSPSSWTGSGEILQSWKKSGF-KPVRYGQCWVFAAVLTTVLRCLGI-
PTRPITNFSSAHDAD-GNLRVDEFY-DASGNHLNEG-ADSVWNFHVWNESWFSRSDLGPS--
YSGWQILDATPQEE--SGGI--YQCGPASRNAVKEGDVDLDYDCPFVFAEVNADCMYWS---YDRA--
TRK---KTLLFNKSTVIGQLISTKAVGRDDRIDITSDYKYEEGSKKERDIFKKA-RKKLGEIEQKP--
-ISGKFKMAGPLEVGKDLNLILVLKNLQSEVK-SVNVNMTAWSTVYTRRPVR-EI-WKDS-
LSVSLSPEEEKHFPIKISYAEYQ-QQLT---TDN-AIEVTALCHV-----EGGI-
VLVQRHIALDNPT-IDIQVLG-EAKVNEEMDVEVVFTNPIDIEVKDCVLQVEGSDLVRGIL-KIDV-
PPLKAKDI-SSTKFKLTPFETGS-KHLLVNFSCDKF-ADI-KAFKTVKV
>lizard_TGM3L2
LTPTYMNWHGSS---NGQAHRTSRF--SASE-PVFRRGQAFHITVYMS---QA-TQGG----
EAFSFVAETG--QSPSESQGTRASFSASG---VGGSWGASLEGREGN--QLTFSLTSPA-
SAPIGRWKLSLRVGQGDA--------
RLLGQFVLLFNPWCSADLVYLGDEDERQEYVLNENGIIFVGNAKYIEARGWFYGQFQKSILDLCLLLM
DLSLYHRKDPGGDSSRRGDPRYVARVVSSMVNGNDNDNGVLEGKWS--
EEFAHHENPSRWDGSVAILWKWAKDRY-RPVQYGQCWVFAGVAATALRCLGI-
PTRLVTNFNSAHDSD-HNLAIDKYY-DPSGKSL-KIGQDSVWDYHVWNEGWFVRGDLGGS--
YSGWQVIDATPQER--SQGL--YQCGPASVMAVKQGQVRLNYDTAFVYSEVNADINCWV---VY-P--
NGT---RKRGHSDTTSIGVNMSTKAVGSSARVDVTGNYKFPEGSSEERAVNRRA-LAELSDLISQP--
-LFGRFRLARPPVLGGDVALVLSLANLREEPT-DVTVNLSVATALYTRRTVR-EVL-KEA-
TTFRLQGKEERQLPLRITYGHYG-AALT---DDR-KLLVTALCDV----PGGVK-
LLVEKAITLEGPD-IRIKVPH-RVVASVPTTVEIGYGNPLPVSVDQCVLLVT---LMGHAV-KINV-
AALSPGEQ-SSIFFEFTPRSSGA-MQLHVDFSCDRF-QHV-KAFTLMDV
>chicken_TGM6L
LKPTHISWQPSV---NASAHHTDRY--ANTE-LTVRRGQPFTITLYFN---RQ-KYPG----
ESLAFVTEIG--PSPSESHRTRAVFNLSE--VGASGWSAAQGPSESG--YMTFIISSPA-
NAIIGRYNLILQVNSGNK-----
IFSRFLGQFVLLFNPWCPGDDVYIANENERQEYVLNENGIIFVGNAKYIEARGWYYGQFQDHLLNICL
TMLDLSLYYRQDPAVDVSRRGDPKYVGRVISSMINGNDNDNGVLLGKWQ--
GSFHSHENPSRWDGSVVILQKWRQDNY-KPVQYGQCWVFAGVMCTVLRCLGI-
PTRLVSNFNSAHDVD-RNLSIDKYY-DSSGKSL-NISKDSTWDYHVWNESWFIRPDLGPR--
YNGWQVLDATPQEQ--SRGL--FQCGPASVVAIKEGDVDLDYDTLFVYTEVNADCNRWI---VY-N--
DGT---KKRVYCDTEIIGRFISTKAVGSNSRVDITCNYKYPEGSPEERRVYKKA-LAFGSEAMRNP--
-ISGKFKLAEPPVFGKDINLILILNNLSTDHK-TVKVDISASSVLYTRRAVA-EIL-KAN-
TSVDLGSKQGKHIRLKIPYAYYG-KYLT---TDK-RIQVTALCEV----
MHMHGVLLVEKTIILEDTN-IIIKIPR-RVVVNKAATLEISYANPLPEPVDRCVLLVT---LMNQQV-
KIHL-ARLAPRER-SRIYFEFTPRRTGP-LQLQVDFSCDKF-SHV-KGFVTIAV
>chicken_TGM6
QKITKVDWHSKL---NKAAHHTSDY--NSTQ-IILRRGQAFAITLNFQ---TT-VQPE----
NNFTFIASTG--PSPAESQQTKAIFHLSE--DAANGWSATQERCEPG--RMSLMIVSPA-
NAIIGRYKLKLQMASGNK-----
TSSALLGQFVLLFNPWCPNDDVYMANEKERQEYVLNDSGIIFQGKEKYIQQEAWNYGQFEEDILDISL
SILDRSLNHRQDPSTDVSNRNDPIYVCRVISAMVNSND-EKGVVEGKWS--
GSYRSGTNPLHWSGSVTILRKWYRGRY-RPIRYGQCWVFAGVMCTVLRSLGI-
PTRVITNFNSAHDSN-TNLSIDKYI-DVSGKTL-DLTEDSIWNFHVWNESWFTRRDLGSF--
YDGWQVLDATPQEK--SKGI--YRCGPASTRAIKEGDVNLDYDSSFVFAAVNADCVTWI---QH-S--
NKR---KERIYSNTRKIGKFISTKAVGTNTRVDVTNNYKYPEGSSKERQVYKKA-LKLLAATTQKP--
-VSGKMKLDAPPVIGQDILLTLALQNLTTDFK-TVNVKLRASAVLYTRKPKAEILQWSRS---
VQLGAEEVKEISFKITYTQYK-NALL---DDR-KILVTAVCEA-----RQGA-LLVEKDIVLQDPF-
LTIEVLG-PTVVHKATNVLVTFTNPLSEVVTDCVLRAEGSGLIKEQL-NINV-ARMAPMET-
ATAEFEIVPYKSGI-RQLQVDLACIHF-SNI-KGFVMLDV
>human_TGM6

```
IRVTKVDWQRSR---NGAAHHTQEY--PCPE-LVVRRGQSFSLTLELS---RA-LDCE----
EILIFTMETG--PRASEALHTKAVFQTSE-LERGEGWTAAREAQMEK--TLTVSLASPP-
SAVIGRYLLSIRLSSHRK-----
HSNRRLGEFVLLFNPWCAEDDVFLASEEERQEYVLSDSGIIFRGVEKHIRAQGWNYGQFEEDILNICL
SILDRSPGHQNNPATDVSCRHNPIYVTRVISAMVNSNN-DRGVVQGQWQ--
GKYGGGTSPLHWRGSVAILQKWLKGRY-KPVKYGQCWVFAGVLCTVLRCLGI-
ATRVVSNFNSAHDTD-QNLSVDKYV-DSFGRTLEDLTEDSMWNFHVWNESWFARQDLGPS--
YNGWQVLDATPQEE--SEGV--FRCGPASVTAIREGDVHLAHDGPFVFAEVNADYITWL---WH-E--
DES---RERVYSNTKKIGRCISTKAVGSDSRVDITDLYKYPEGSRKERQVYSKA-VNFGVEPATKP--
-IAGKFKVLEPPMLGHDLRLALCLANLTSRAQ-RVRVNLSGATILYTRKPVA-EIL-HES-
HAVRLGPQEEKRIPITISYSKYK-EDLT---EDK-KILLAAMCLV-----TKGE-LLVEKDITLEDF-
-ITIKVLG-PAMVGVAVTVEVTVVNPLIERVKDCALMVEGSGLLQEQL-SIDV-PTLEPQER-
ASVQFDITPSKSGP-RQLQVDLVSPHF-PDI-KGFVIVHV
>platypus_TGM6
VKIAEVDWRREA---NGQSHHTAEF--PGPE-LVVRRGRPFRLALGLS---RA-LDPS----
EDLVFTVETG--PRASEELRTKAVFSTSE-EEEEEEWEAAVEEEAGAPGSVGVSISSPA-
DAPVGRYKLSARVSSRGP-----
HRIKKLGQFVLLFNPWAPEDTVHLSSEEEREEYVLNEHGVIFRGVKERIRAQGWNYGQFEEDILNICL
SILDQSPSAQLDPLTDVSRRGDPIYVSRVVSAMVNSND-DKGVVEGQWR--
GQYGGGTNPLAWRGSVAILRKWYRGRY-KPVKFGQCWVFAGVMCTVLRCLGI-
ATRVVSNFNSAHDTD-RNLIVDKYV-DSFGRALDDITEDSMWNFHVWNESWFARPDLGPS--
YNGWQVLDATPQEQ--SQGV--FRCGPASVAAIREGEVDLAYDGPFVFAEVNADYVTWL---WQ-A--
EGA--AREPVRTDPRAVGARLATKALRGDRPCDLTRCYKHPEGSKKERQVFRKA-VRKLFEPAKKP--
-VSGKFKLLEPPAVGRDLRLALGLANLTARPQ-KVKVNLSASTILYTRRPVA-EVL-HES-
RAVKLGPREEKQIPISIPYSDYM-KDLT---SDK-KILVSAMVLV-----FKGE-TLLEKDVTLGDF-
-ILIKVLG-PAVVDEPCTVAVTVSNPLPEAVDDCVLVVEGSGLLAEPL-SIEV-PPLEAEEK-
ATVRFDVTPYKSGS-RQLQADLVSARF-PDI-KGFLTVHV
>lungfish_TGM5L
LLVKSVDLQFKE---NNRQHRTEEI--SKKK-LIVRRGELFIITVNFHT--RG-YEEGR---
DNITLIAETG--PRATEEAKTKAVMTVGA-SVKPNMWNTTVKNSHDH--TLTLSVSSPA-
NACIGKYTFQMQLSSDGN-----
KTSYNMGEFILLFNPWCKEDEVSMTDEILTQEYVMNEQGTLFFGSTDYISSSPWDYGQFQEDVVDICL
KLLDLNPKYLKSPSKDCARRGDPIYVSRVVSAMVNSND-DKGVVEGRWH--
GDYSDGVSPGTWSGSVPILRKWYKSGY-KQVKYGQCWVFAAVLCTVMRCLGV-
PTRVVTNFSSAHDTE-GNLTIDEYY-DLKGKKLDRS-YDSIWNYHVWDECWMKRPDLEEI--
YSGWQVVDSTPQET--SEGV--YCCGPAPVLAIKEGHTHLKFDLPFTFSEVNADVIRWI---SY-P--
DGR---TKKASSDTKLVGQYISTKTVGSDEREDITKKYKYDEGTTQEREAVARA-LRNIRPNLPAT--
-IDVSIKVDKTPVRGQNIDMLVNVTNKSPAKK-HLMLFISAQCMFYDGRPGT-RF-WKKE-
ADIQLASNEGKDFPYQILYSEYD-EYLS---DSR-LIKIAATVTE----QETTQ-
FLAEKDIFLSNPT-ISIQAPD-EALKYEPTTAEVLFSNPLPEPLNSCVLTVSGSGLIYDEV-KIDG-
RQMKPGVR-GILRVEFTPFKTGM-RRLQVVFECDKF-KDV-KGYKDIKI
>zebrafish_TGM10
AILSDVDLQCYE---NNHAHRTEEM--DVER-LLVRRGQPFSVVLQCT---EQIPQLPD---
HQINLILHLG--------KNNEVVLKVSDSEQDPGKWWFSQRNAQGE---VMLTLHSPA-
DAPVGLYSMTVVLLSADG---
EIQEQTSPQTFYLLFNPWCKDDCVYLPSEEMLQEYILNENGILYQGAWDDITTVPWNFGQFEKDVVDI
CFDVLDNSPAALKNSEMDIFNRASPVYVSRTITAMVNAND-DRGVVSGRWD--
GEYSDGVAPTRWTGSVPILRRWSEDGG-QKVRYGQCWVFTGVACTVLRCLGI-
PTRCITNYSSAHDTD-ANIAVDYLV-NDQLESVSEGRKDTIWNYHCWVESWMKREDLPEG--
YDGWQVLDPTPQER--SDGI--FCCGPCPVRAVKEGEVGLKYDTPFVFSEVNADLIVWI---VH-P--
DGE---RSEVSQNSKIIGRKISTKSVYGDFREDITANYKYPEGSMKEREVYKKA----GRDGPGQL--
-LFIK---HAPAIHGTDFDVIIEVYNAGREDT-DAKLTVTSNAITYNSIHRG-ECQ-RKT-
TSLTVPAYKAHKEVLRLQYDHYG-ACVS---EHH-MIRVTALLQP----NDQDN-
ILQETNIPLKMPA-LHVKIIG-NAIVSRKLTAHISFTNPLPINLQGGVFTVEGAGLTEARE-IKTH-
GKIESGQT-VTVKFSFKPTRAGL-RKLLVDFDSDRL-RDV-KGEASVIV
>shark_TGM10
```

LYLGAVDLRCEA---NNTEHHTIEI--DKDR-LLVRRGQEFRLHVEFKH--RA-FVEGE---
DQLAILLDTG--PAPSEADGTRIKVLNSG--VKWDKWTFRLQCTPGH---IHLAVHSPA-
NACTGYYQIYLILYPTGG---
ESIQRITAGDFHLLFNPWCKEDAVYLPDEDLLQEYILNENGLLYHGSYANIYTLPWNFGQLEKDVIDI
CFLILDNSLSALKNPLADAPRRNDPVYISRTVTAMVNAND-DKGVLLGRWD--
GNYSDGIAPTRWTGSLPILRLWTSSGA-DKVRYGQCWVFAAVACTVLRCLGI-
PSRCVTNYSSAHDTD-GNLKVDQYYSSEDYSRVPSKKKDMVWNYHCWLEAWMTRPDLPPG--
YDGWQALDPTPQER--SDGI--FCCGPCPVKAIKEGHVDMKYDAVFIFAEVNADVVYWL---VN-K--
DGS---KKELGVKQHQVGKQIITKSAYTDEREDLTHDYKYPEGSAKEREVYNKAMKIRSTNIQQKD--
-LKISIKYA-QPILGSDFDVYFSIVNHGFMDK-DIHLTLTATTVTYNGFILT-EFS-KRS-
TTFMLKAATVQKEVLRLKYKDYG-EHLS---EHN-LIRLTAMLTI----
EGTSEVSLKECNVALNLPQ-LTVKVVG-EPILYRELTVQIKFVNPLPITLTGGIFSVEGTGLTDLKE-
IKSPTAAIHPGQE-VVVNASFKPTKTGL-QKLMVDFDSNRL-RDV-KGSTNIIV
>coelacanth_TGM10
LGV--VDLHCNY---NNTAHHTDEI--DSER-LVVRRGQPFTITVECR---SGLFHPGR---
DQLAIVLDIG--QKPSTDNGTRIKISSSD--AKAEKWRFAMQADQYE---LQLTLYSPA-
DAPVGQYTILLLVHSDSK----
LIQKKAAGQFYLLFNPWCKDDTVYLPDKEMLNEYIMNENGQLYQGSWNDIYPVSWNFGQFEKDVVDIC
FEILDNSLPALNNPVADILKRKDPIYVSRIVSAMVNAND-DKGVLLGRWD--
GNYFDGIPPTRWNGSVQILRQWSKSGA-KKVRYGQCWVFAGLACTVLRCLGI-
PTRCITNYYSAHDTD-GNLKVDKCF-NEDLDVLPGKRKDMIWNFHCWVESWMTRQDLPSG--
YDGWQVLDPTPQER--SDGI--YCCGPCPIKAIKEGNINVKYDASFVFAEVNADVTCYI---VK-K--
DGT---KSETAVYCHQVGKCISTKSIFGDNREDITEQYKYPEGSSKEREVYAKAGLKNVSSNTKVL--
-IAIK---HTRAVHGSDFDIFIEVCNNSSVDK-DVGVTIVAKTVTYNGIILK-DCH-KKI-
TSFSLMASNAKKEVFRLKYEHYG-EHLS---EHN-LIRITALLLQ----KGKNE-
ILTERDIALAMPQ-LTVKILG-EPMVSRKLKAHIKFVNPLPITLTDGIFTVEGAGLTDLQE-IKCP-
GKIKPGEE-VTVDVSFIPTKTGL-RKLLVDFDTSRL-RDV-KGYASVII
>lungfish_TGM10
CIFDTVDLLCRS---NNGAHHTDEI--STKR-LLLRRGQPFHISVKCR---
QNILTKPVLPFHFINVLMSIG------KNNGTQIKLSTMK--TETTEWRFSMELAGDE---
LLLTICSPA-SAIIGRYNLYLMLYDSQN---
QLQQQKSAGQFYLLFNPWCREDTVYMSEEEKLQEYIMNEHGVLYQGMWNDIYEVPWNFGQFEENIVDI
CFEILNNSLPALSNPVNDTLKRSEAEYISRIVTAMVNSND-DKGVLLGKWD--
GKYWDGIPPTRWTGSVQILQQWSRSGA-EKVRYGQCWVFAAVACTVLRCLGI-
PTRCVTNYCSAHDTD-GNLNVDCYL-NNELQLIPGSTKDIIWNFHCWVESWMARHDLPPG--
NDGWQILDPTPQER--SDGV--YCCGPCPVKAVKQGNVDVKYDTAFVFAEVNADIVYWV---LN-K--
DGT---RSQTGAFCKEVGKCLITKNAYGDKREDITQHYKYPEGSKEEREVYAKAGMKRLCSESK----
-VCLTFKHA-TVVYGTDFDVYVEICNNSFVDK-NVNLTIVAKTVTYNGIILQ-ECH-RKT-
TSFPLKALKVKREVCRIKYEHYA-DRIS---EHN-LIRITALLSQ----NGKDE-
ILSEKDIVLAVPQ-LNIKIIG-EPVLFQRCMACVSFVNPLPITLLNGIFTIEGAGLTNIQE-
IKSKPEEIRPGQE-VTVTVPFIPTKAGL-RMLLVDFDTNRL-KAV-KGHVSVLV
>shark_TGM2L3
LTTLAVDFQCEK---NNQEHRTAEI--DTTR-LIVRRGQPFHITL------QS-
YTDEYIDDDTTALSAETG--PKPSTTSGTKVLFALNS--FSTNGWIGKVTYNTGT--RLTLDISSSP-
NAKIGRYSLSLLAIKGGL-----
VSSCKLGEFILLFNPWCTEDEVFLDSEEQREEYVLNEDGIVFMGDNHCIQSRSWYFGQFEKKVIDICL
MLLDMNLKCLKCPGRDYVRRNDPVYISRVVTAMVNCHD-DNGILEGKWD--
GPYTGGVLPWNWNGSVAILHRWYNRGY-QRVQFGQCWVFAAVACTVLRCLGI-
PTRVVTNFNSAHDSN-GNLTIDTLY-DECGRKYGRQ-SESIWNFHVWIESWMARNDLRPG--
YDGWQALDPTPQEK--SEGI--FCCGPAPVNAIKEGAVDMKYDVPFVFAEVNADQISWI---MH-R--
DGR---KEKIHVETQHVGQNISTKSCGRNGRDDVTHSYKYPEGSAKERAIVSEALTNRLCSQPENK--
-LHVHVKTEKSINNGSDTQVLITISNRSSTNM-VCKLNFNAHMKSYDGKLMR-QITEKNL-
EQIAVQANEDETVALEVAYSHYG-DYLE---HHH-LIKLTALAFD----
MVTKESAMAMKDLLVINPD-IAVQIHG-DPIVHKPLTAEICFTNPLRVPLNRCVFTVEGVNLIYGME-
QFNI-DEIKPNQT-MTINVEIIPKKAGL-RKLMVNFDCDRM-KDV-KGYKNFTV
>zebrafish_TGM2a

```
VEIGSIDLACEV---NNTNHHTHLN--GVDR-LIVRRGQTFTIHLHLKE--GTHFQNG----
DNIKFIAQTG--PIPSVEAQTKARFSLSK-VISRLSWSATAETHNS---TVSLSICAHT-
NAPVGRYTLILDQGDGVI----------
LGEFVLLFNPWGKLDSVYLANEAEREEYVLSQDGLIYRGTPKRITVLPWTFGQFEHGILDICLQILDE
SPNYISDAALDCSERKNAVYVTRVLSAMINSLG-DKGVVVGNWS--
DDYEDGVKPTVWKDSCSILRQWSNEGC-RAVRYGQCWVFAAVACTVSRALGI-
PCRVITNFGSARDSN-GDLIMERFY-NEFDENIAD---DSIWNYHVWVENWMTRPDLALG--
YEGWQASDPTPQHR--SDGV--FCCGPASVRAIKEGELTFKFDVPFVYAEVNADVVEYI---KL-R--
DGR---VFKMGGSTTEIGKSISTKAVGRDEREDITHNYKYPEGSEEERKVFKKA-NHHNKQAGEEP--
-LHIKIRVTPDMQIGSDFDVYAEIKNNTMVTK-SCRVMFYAQAVSYNGTLGE-TCGLGDF-
TEMSLASSDGGKVTLRLEYAEYS-KAIT---QDR-MIKLVGLLID----AETRE-
YRAKKTIVLDAPE-IIVNILG-VPKVGRNLVADLALQNPLPEPLENCVFTIHGANLTDGKPITHEV-
GTIGPKEF-ATAKVEFAPKLPGQ-RKLIIDFASDKL-HNI-ETYENLVI
>shark_TGM2L4
LAVAGTDFQCEV---NNKAHRTADF--GSNR-LIVRRGDRFTVTVHFAG--RG-YQGAE---
DQISVIVETG--LAPSVTSGTKAQFPLSN-SLDESKWNAALVSSAGN--QLSLSICSPA-
NAKIGHYTLKLCTTQGQS------
TPFDLGKFILLFNPWCSDDAVFLDFENQRKEYVLNDQGLIYQGTKKLISHIAWNFGQFEDGIVDICLK
LLDNSSNCLKNQEEDCSQRHDPVYISRIVAAMVNCND-DKGILQGNWG--
PDYSCGVPPTMWNGSITILRRWNKLGC-QPVRFGQCWVFAAVACTVLRCLGI-
PTRPITNFNSAHDTE-QNLRIDSFI-DENGK-ISKKSKDSVWNFHCWIESWMTRPDLKPG--
YDGWQVIDPTPQEK--SEGI--YCCGPASVKAIKSGDIDQKFDSPFVFAEVNADYVSWL---LC-K--
DGS---KKQIEVNHRLVGQNISTKAVGSDEREDVTHNYKYAEGSEEEREAFTKAMKNKLTQEPEKK--
-FFLKLKAEKVNLNGADFEVSAVLSNQTSAVK-NCRLILCAKTILYNGQSIQ-ECSWKDL-
AKLTIRPHEEKTETLQVHYSNYG-QSLT---EHN-QILIVALAME----
YEAGELVVTRKVITLQNPD-LHIKIIG-
EPVQYRDLTAEIYFTNPMPVNLCNGTFLVEGAGLTDEQKVPCPV-QSIKPGQE-
VKVRVKFTPQKPGL-RKLAVDFDCNKL-KDV-KGFKNVIV
>platypus_TGM2
LQLQRCDLELEN---NGRCHHTSEL--SADR-LVVRRGQSFSVTLHFEG--RS-YEEGV---
DNLTFTVETG--PEPSTETGTKARFALSA-EEPVGCWSAFLMNQDDA--SLSVALCSPP-
DAPVGQYQLTLETDIGTQ-----
GSRFPAGKFILLFNTWCSEDSVYLDDEREREEYVLSQYGIIYQGSHKFINPTHWNFGQFEDGILDICL
HVLNLTPRPRQDTEGNGFWRQSPIYVSRVVSSMINSNDEDHGVLLGRWK--
EPYEDGTNPNFWTGSVAILRKWMESGS-QQVRYGQCWVFAAVACTVLRALGI-
PTRVVTNFNSAHDTD-RNLVIDIYY-DASGQRLEGQ-RDSIWNYHCWVESWMTRPDLQPE--
YNGWQAIDPTPQEK--SEGI--YWCGPASVNAIKEGDLSGGFDVPFVFAEVNADVVHWF---QE-S--
DGN---RRKYSSDPRLVGKNISTKGVGTDHRQDITHCYKYPEGSSEEREAFRKA--DRQLPEPERA--
-LSVRIKGSEHMDKGSDFDVSALVTNGTDGDL-SARLLLCARTVSYNGILGP-PCGFVDL-
RDFSLPARNDQTVPLRIRYHDYS-PYLT---ESN-LIKVMALVTE----PQSDS-
LMAERDIYLKNPD-IRIRILG-EPKQYEKLVAEVSLKNPLETPLLDCCFTLEGAGLTLGQQ-
VIHLPDPVAGGQE-AKGRLALCPQQSGL-RKLVVDFESNVL-KAV-KGFRNVIV
>zebrafish_TGM2b
LDIGSWDLACKF---NNTDHHTELN--GTDR-LIVRRGQAFTINLQLN---SGSYQPGY---
SQINITAETG--PDPQQQYGTRAVFSLSS-EVDSSCWSAAVSSPPGE--SVCLSICAAP-
DAPIGHYTLTLDERIQIQ------------
FILLFNPWCPLDVVYMDNEEKLAEYVLAQDGIIFRGDAGYPVPLAWNFGQFEEGILDACFRILDMNPK
HRRNPAKDCSGRRNVIYVTRVLSAMINSNDQDSGVLEGCWR--
DTFDGGVSPMSWGGSVQILRTWDRSSC-LPVRYGQCWVFAAVACTVARAVGI-
PCRVVTNYYSAHDTN-SNLLIERYV-NEKGEVDHSSTRDMIWNYHCWVESWMGRSDLPPG--
FDGWQASDPTPQEK--SEGV--FCCGPVPVRAIKEGELTFKYDAPFVFAEVNADLVYFL---KS-K--
DGS---TRKINYDQK-VGQKISTKSVGRDEREDITHLYKYPEGSADERRVFEKA-NHQNKQEKQNT--
-LNITIKLSSGVRKGCDFDVFAIVTNGTAEEK-KCRLVFASRAVSYNGVIGR-ECGFKDL-
LNVELPPGGERKVPLRLNYSKYC-NNLT---EDN-LIRLGALLID----YSTRD-
IMAMRDIVLDDPE-IKIRILG-
EPKENRKLAAELTIQNPLPEALQSCCFTIEGANLTGGDSITHTLDSSIEPGQE-
AKAKIYFTPTQSGL-RKLLVDFNSDKL-GHV-RGYRNVII
```

>coelacanth_TGM2

------PCPVETSGTRAKFTLSN--SVTEDWSAAIICNNGN--VLSLSVQSPA-
NARIGRYFLTMESQDSSI---------
DLGEFILLFNPWCPGDSVFMTDERKLKEYVLTQDGIIYQGTVEYITSTPWNFGQFEGGILDVCLLLLD
TNPKFFKNSDKDCSRRNDPVYITRVVSAMVNCND-DKGVLYGKWD--
GEYDGGISPLHWTGSVQILRNWMTAGC-LPVRYGQCWVFAAVACTVLRCLGI-
PTRVITNYYSAHDTN-SNLIIERYI-DEKGMSVNKS-KDSIWNFHCWVESWMTRSDLKPD--
YDGWQVVDPTPQEK--SEGV--YCCGPAPVKAIKEGEVALKYDVPFVFAEVNADVVFWI---KH-P--
NGK----QEKAVYPSQVGKSISTKSIGSDTREDVTHHYKFRDGSPEEREVFLKA--DHQHTQKPQT--
-LDLTIKVSDGMNNGCDFDVFAVITNRTSVEH-FCRLMFCARTVSYNGVIGN-ECGMKDL-
LNVTIPANQEKRIPLRVLYSKYG-DSLT---QDN-LIKLMALLFE----YQTKD-
LLAVRDIYIDNPK-IKIKILG-EPAQNRKLAAEITLHNPLSVPLTDCLFTVEGAGLTDGQQ-
IQQAVGPVDPGQE-AKAKVYFTPRQSGL-RKLVVDFDSNKL-RNV-KGYRNVII
>lungfish_TGM2
LVMERCDLECET---NNKRHRTSDF--GIHC-LVVRRGQAFRITVYYKG--RG-FQQHT---
DTLTFTAQTG--PCPNEVSGTKSQFPLTN-SLNEEAWSAAVEQNDNS--ALTLKICSPP-
DARIGRYTLNMEATTAGK-----
GVSIPLGQFILLFNPWCSGDSVFMDDEAKLREYVLTQDGLIYTGTDKRINSMPWNFGQFESGILDICL
DLLDKSKTFQNDKDKDCSRRNNPVYISRIVSAMVNCND-DDGILMGRWD--
NNYGDGISPLQWMGSVKILRNWQNQSC-QPVRYGQCWVFGAVACTVLRCLGI-
PTRVVTNFSSAHDTN-GNLIIERYL-DETGKEIKKS-KDSIWNFHVWDECWMARPDLGKG--
YDGWQAVDPTPQER--SEGV--YCCGPAPVQAIREGDIKVKYDLPFVFAEVNADVIYWM---QR-R--
DGK----DEKVVHSSVIGKSISTKSVGSDAREDITYHYKYPEGSEEERRVFEKA--DLQNAQKPVT--
-LEVKIKVVEAMKYGCDFNVSAVIVNKTSSDR-VCRLMFCARTISYNGEIGN-ECGMKDL-
LNLEIPANQEKSVPLRVLYNKYC-TTMT---EDN-LIKLMAILFE----YKSKD-
MLATRSIVIDNPD-IKIKILG-EPGQFKKVGAEVTLKNPLPVPLTNCSFTIEGAGLTEGQV-CENI-
RDVDKNQE-AKAKLYFTPKQSGM-RKLVVEFNSNKL-QNV-KGYRNVII
>human_TGM2
LVLERCDLELET---NGRDHHTADL--CREK-LVVRRGQPFWLTLHFEG--RN-YEASV---
DSLTFSVVTG--PAPSQEAGTKARFPLRD-AVEEGDWTATVVDQQDC--TLSLQLTTPA-
NAPIGLYRLSLEASTGYQ-----
GSSFVLGHFILLFNAWCPADAVYLDSEEERQEYVLTQQGFIYQGSAKFIKNIPWNFGQFEDGILDICL
ILLDVNPKFLKNAGRDCSRRSSPVYVGRVVSGMVNCND-DQGVLLGRWD--
NNYGDGVSPMSWIGSVDILRRWKNHGC-QRVKYGQCWVFAAVACTVLRCLGI-
PTRVVTNYNSAHDQN-SNLLIEYFR-NEFGEIQGDK-SEMIWNFHCWVESWMTRPDLQPG--
YEGWQALDPTPQEK--SEGT--YCCGPVPVRAIKEGDLSTKYDAPFVFAEVNADVVDWI---QQ-D--
DGS---VHKSINRSLIVGLKISTKSVGRDEREDITHTYKYPEGSSEEREAFTRA--NHLNAEKEET--
-MAMRIRVGQSMNMGSDFDVFAHITNNTAEEY-VCRLLLCARTVSYNGILGP-ECGTKYL-
LNLNLEPFSEKSVPLCILYEKYR-DCLT---ESN-LIKVRALLVE----PVINS-
LLAERDLYLENPE-IKIRILG-EPKQKRKLVAEVSLQNPLPVALEGCTFTVEGAGLTEEQK-
TVEIPDPVEAGEE-VKVRMDLLPLHMGL-HKLVVNFESDKL-KAV-KGFRNVII
>chicken_TGM2
LVLETCDLQCER---NGREHRTAEM--GSQQ-LVVRRGQPFTISLNFAG--RG-YEEGV---
DKLAFDVETG--PCPVETSGTRSHFTLTD-CPEEGTWSAVLQQQDGA--TLCVSLCSPS-
SARVGRYRLTLEASTGYQ-----
GSSFHLGDFILLFNAWHPEDAVYLKEEDERREYVLSQQGLIYMGSRDYITSTPWNFGQFEDEILAICL
EMLDINPKFLRDQNLDCSRRNDPVYIGRVVSAMVNCNDEDHGVLLGRWD--
NHYEDGMSPMAWIGSVDILKRWRRLGC-QPVKYGQCWVFAAVACTVMRCLGV-
PSRVVTNYNSAHDTN-GNLVIDRYL-SETGMEERRS-TDMIWNFHCWVECWMTRPDLAPG--
YDGWQALDPTPQEK--SEGV--YCCGPAPVKAIKEGDLQVQYDIPFVFAEVNADVVYWI---VQ-S--
DGE----KKKSTHSSVVGKNISTKSVGRDSREDITHTYKYPEGSEKEREVFSKA--EHEKLGEQEE--
-LHMRIKLSEGANNGSDFDVFAFISNDTDKER-ECRLRLCARTASYNGEVGP-QCGFKDL-
LNLSLQPHMEQSVPLRILYEQYG-PNLT---QDN-MIKVVALLTE----YETGD-
VVAIRDVYIQNPE-IKIRILG-EPMQERKLVAEIRLVNPLAEPLNNCIFVVEGAGLTEGQR-
IEELEDPVEPQAE-AKFRMEFVPRQAGL-HKLMVDFESDKL-TGV-KGYRNVII
>clawed_frog_TGM2

```
LYLESFDLDCSG---NNRSHRTAEA--TCER-LIVRRGQPFQITLNFSP--RG-YEEGV---
DKLSLNAVTG--PCPSEESGTSNHIPVSD-ALQDGAWSAAITSTDGG--TLILSITSPP-
DARIGYYNLSLETSTEYQ-----
GSSFQLGSFTLLFNPWCPEDSVYLETEEERKEYVLCQHGIIFQGTKDSVEHVPWNFGQFEDEILDITL
QVLDTSPKFLNDSNRDCSRRNDPVYISRVISAMVNCND-DRGVLFGRWD--
NKYDDGISPMFWMGSVAILRRWRKFSC-QAVKYGQCWVYAAVACTVLRCLGI-
PARVITNYNSAHDTN-SNLLIEQYL-DEHGKRQPKQ-KEIIWNYHCWTEAWMTRPDLGEA--
YNGWQVVDPTPQEK--SEGT--YCCGPTPVKAVKEGDLNLKYDVPFVFAEVNADVVYFV---QQ-N--
DGS----VKKTQFISLVGQKISTKAIGKDEREDITLNYKYPEGSEDERRVFEKA----NKVETPSD--
-FTIKIKVSEGMNKGSDFDVFAVITNNREEEK-QCRLMFCARTCSYTGEVGP-ECGMKDL-
LNLTLTPQEEKRVPLRILYEKYG-PTMT---ENN-TIKLVAMLYD----YSSKE-
ILAVRDIHVKNPS-IKIKVLG-EPKQKRKLVAEISLKNPLAEPLTGCCFTVEGAGLTAEQL-
VKTLDCPVEPGQE-AKVRVDLMPQLAGK-LSLVVDFESDLL-KAV-KGYRNIII
>lizard_TGM2L
LQLAAWDLLCEH---NNEDHRTSDA--GSQR-LLVX--QPFLVTLHFSG--RP-FDKAV---
DKLTFHVETG--PCPNETSGTKASFPVSC-FLEKTAWSSAVENQDRS--SLTMFIFPPP-
DAQIGRYHLNLEVSTKGQ-----
GSSYYIGEFILLFNPWCKEDTVYMESEEARIEYVLTQHGQIFNRNKYCIGSIPWMYGQFEKGIMDICL
KMLDTSLNFLQDQDKDCSRHNSPVYYVSRVVCAMLSQ-------------------------------
--------------------------STGYICFSR-----------------------QY--------
-----------FHCWAESWMGRPDLPEG--YGGWQVLDPTPQEK--SGDI--
YCCGPAPVKAIKEGDVHLKYDVPFVFAEVNADMVCYL---RQ----YGM--
PWKVISIDTSKTGMNISTKSVGRDTREDITHLYKYPEGSKEERAVFAKA-RHKVQLPLKET---
LKVRIKVSEGINNGCDFDVFAVIKNNTAGKH-WCELKIGTRIVSYNGALGP-ECRSKDN-
LGITLEPYEEKAIPFQILYKKYG-QRLT---QDN-MIRVTSLLLC----QDTQE-
FVGMRNIYIKNPD-IKIQILG-EPMLNRRLVAELTLTNPLPAPLTDCVFTVQGAGLTGGQK-
VQKIDSPVGPGEE-AKVKVDFVPFLSGP-RKLVVNFESNKL-KGV-KGYCNINV
>lizard_TGM2
LQLETWDLLYEH---NNEDHRTADA--GFQR-LLVRRGQSFIVTLHFSG--RS-FDEAV---
DTLTFHVETG--PCPIETSGTKSSFPLSC-SLEETVWSSAVEGQDGS--SLTLFVFPPP-
DARIGRYRLTVDVSTEGQ-----
GSSFDLGEFVLLFNPWSPDDTVYMESEEACVEYVLTQHGHIYQGSKDFIYSIPWNFGQFEEGIVDICL
QLLDTNPKFLRNQDKDCSRRNNPVYYVSRVISAMVNCND-DQGILFGRWD--
NKYDDGVSPMAWSGSVDILQRWQKFGC-
QPVRYGQCWVFAAVACTALVCLGHPPGRVVPNYNSAPQHPWEPWSIEQYL-EQSGK-
LQQGDRELIWNYHCWVEAWMARPDLSEG--YDGWQVLDPTPQEK--SEGV--
FCCGPTPVKAVKEGDLHLKYDVRFVFAEVNADVAYLM---LQ-K--DMS---RKKTTI-
TTTVGKNISTKSVGRDSKEDITHHYKYPEDSEEERAVFEKA---QLHPPPAEE---
LKVKIKASEGMNNGCDFDVFALLTNNTAEEH-RCRLMFGARTLSYNGALGP-ECGSKDL-
LNITLEPHAERTVPLRILYEKYG-HCLT---QDN-MIKVMALLVD----LDTQE-
VLGVRNIYVKNPD-IKVRVLG-EPMQKRKLVAELTLTNPLPTPLTGCVFTVEGAGLTDRQK-
VQEIDSPISPGEE-AKVRVDFVPRQSGL-RKLVVDFESDKL-KGV-KGYRNVII
>shark_TGM2L
QDVSSVDFHCEK---NNPDHRTAEI--SAKR-LIVRRGQPFHITVQFK---RNQYNPDV---
DRFKLVAQTG--PSPSETSGTKILFSLSD-SINKRRWNAVAACSSRS--RLSLIIHSAP-
NAKIGRHTLALQKITSDQ-----
TVIYTVGEFVVLLNPWCSEDEVFLNDAGQLNEYILNEQGIIFTGCSEYIQHLPWNFGQ----------
---------------------------------VNCND-DNGILFGKWD--
APYTDGVYPGKWSGSVAILRQWNNSGC-QPVCYGQCWVFAAVACT-----GV----------------
--------------------------------------------------------------------
-YCCGPAPVKAIKEGETDICYDVPFIFAEVNADCVISV---YS-E--KGK---
KMKVDTDMRHVGQRISTKCVGSDDREDITNNYKYPEGSEEERRIFELADKRRVPLKPGKR---
LQLHLMTNDPNYNGTNVKVSAVISNKNSMKR-VYTLKINAMKKKYSRSSRG-KCIQMYQ-
QEITVAPSEDKTMEVELSYTEYG-ELLD---KYN-LIRFTALAID----
GETNESVFTLKDICLINPN-ITIQVRG-APVLSQEVMTEICFENILPVALTNCVFTLEGAGLIDGQM-
EIRI-GALNPGEA-ITKEVSFIPKKMGL-KKLTVEFDSDNL-KDV-KGYINIDI
>shark_TGM2L2
```

MSVDPVDFQFEK---NNKEHRTDKI--STKR-LIVRRGQSFSIKVNFT---DG-FNPND---
NKLKMIFETG--PDPKKLNGTKVEVPFTK-SINLKRWSGIITSSTSN--KLCIAISPSP-
RAKIGYHRLILEHAYKSD------
VQYHLGNFVVLFNPWCSEDEVFLNSDLQRDEYVMNETGIIYVGSSDYIHDVPWNFGQFEEDILDICLK
LLDKTPKYLKNPNKALRRRGFPVYIARIVSAMVNCND-DTGILYGSWS--
PPYSDGVYPGKWNGSVAILRKWHNSDC-QAVRYGQCWVFAAVTCTVLRCLGI-
PTRVVTNFDSAHDTD-ANLTIDEYY-NVEAENLGES-ADSIWNFHVWVESWMARNDLSPG--
YDGWQAVDATPQEE--SDGI--YCCGPASVNAIKEGEMDMQYDIPFVFAEVNAHCVDWL---VF-N--
SGE---KMKMKVNESRVGHKISTKRCGSEEREDITSNYKYPDGSVQEADVFEKANRMQNIPTPEKT--
-LSLSIVTELPIYNGKPIAVSMVVSNNTSEQK-VYNLRFWAKKRKYNAVTEN-QCIKKHE-
QEITIAPNTEKKIPLKVDYKEYG-LFPD---MYN-LMKLISVVTD----
VSSKSSAFAMKDVSLINPP-LIIKMLNFSAVVNKKVHIEISFQNSFPETLKNCVMTLEGAGLIEGEK-
EIKF-PNIAPNEE-AKVKCDFVPYKSGM-KKLLVDFDCDKL-RDL-KGSMNIIV
>fruit_fly_TGM
LGVLKVDLCLED---NHEEHHTSHF--AKEA-LVVRRGEPFRLKIHFN---RD-YSPS----
DAISFIFTVA--TKPSPGHGTLNALVPHD--GDTLEWGAGIESHEGQ--TLTVLIKPPS-
TCPVTEWKLDIDTKLLRS-------
YPLPLPIYVLFNPWCPDDQVYLEDRDQRKEYVMHDTTLIWRGSYNRLRPSVWKIGQFERHVLECSLKV
LGTV-------RIPPAYRGDPVRVARALSALVNSVD-DDGVLLGNWS--
EDFSGGVAPTKWTGSVEILQQFYKTQ--KSVKFAQCWNFSGVLTTIARSLGI-
PSRIITCYSSAHDTQ-ASLTVDVFI-DANNKKLAET-TDSIWNYHVWNELWMQRPDLGVG--
FDGWQVVDATPQEA--SDNM--YRVGPASVAAVKNGDILRPFDGGFVFAEVNADKLYWR------Y--
NGP-QPLKLLRKDTLAIGHLISTKAVLKWEREDITDTYKHAERSEEERSTMLKA-LKQSRDNFNDI--
-FDME---KDDIKIGQSFSVVLKVSNKSESRT-MATGQISCDAVLYTGVGAV-EVK-TLG-
FELELEPKSSDYVRMEVIFEEYY-DKLS---SQA-AFQISAAAKV----DTDYD-
YYAQDDFRVRKPD-IKFQLGE-AAIVQKELDVILRLENPLPIPLHKGVFTVEGPGIE-QPL-KFKI-
AEIPVGGT-AAATFKYTPPYAGR-GTMLAKFTSKEL-DDV-DGYRHYEI
>tunicate_TGML1
LAVKSVDLLKSD---NTVQHYTSDY--EGSE-LVVRRGQPFKLLITLS---RA-LKKE----
EEVEFELRMG--GRPMVAHGSLIPLDPKP---DEDVVGFKLLANAGD--
ILTVEIYTSAENTGVGKWLLALRAMEGRK---
KLPRMTVTDDIIIVFNPWSKFDPVYMENEAWRDEYVLNEEGLQFYGTSRRHGKMEWVFGQFEAHCMKA
AMKLLLMG-------SLRYKDHKEPVMVARHMSALVNSND-DNGVLVGNWS--
GDYSGGRSPSFWHGSTAILKQFVKTG--KPVNYGQCWVFSGVLTSVLRCLGI-
PTRSLTTFDSAHDTG-GNLTIDKHY-NETGKPLEN--DDSIWNFHVWNDVWMARPMLPEG--
NGGWQALDATPQET--SDGK--FQCGPMPVSAIKEGDINFDYDGPFIYAEVNAVEKHWRKLKKP-
QVVDGKMIEYSEIGANTTKVGKLILTKAVNSWAEEDITHSYKYPEGTKEEALSFKNA-
RKHVKKEVKNL---VLFKPDVPASVTFGKDITFPIKVENVSKSSQ-NVFISVVAKSVQYNGSIVK-
EIL-DVD-LSDDIAAGKTHTFNVSLPFNTYK-DSTR---GEN-DVKFFMLGGV----
TGNEDQFSEQDLVDLEKPD-ITVKVPA-SAQVGKQINVKASFTNPLSISLTGCMFTFEGAGIR-DET-
IVEV-SDVKPKAV-SSVDVKITPRVVGT-RKVIVGFSSKQL-EGL-RGNAELKV
>tunicate_TGML2
LKTVDCDWMSSE---NASDHHTTEY--SAKY-LILRRAQTFTMKMKFQ---RK-FHKTA---
DRVVLELSLG--TGSQLMNETKVRCPLVA-TLDPTKWGMVITHEEDETFLVTFTVNIPP-
KALVGKYKSVVEFTSQLESGESVTTRDNEPELAILFNPWSKLDSVYMENEAEREEYCLNDLGIVYRGS
KVRISGKKWNFGQFEENILECSLLLLDKDKRAKEKPNKWIQKRGDPVWISRAVSAMVNAQD-
DDGVLVGNWS--GDYSGGVSPTKWNGSVEILQQYYNTG--KPVSYGQCWVFSGLVTTVLRSLGI-
PTRSVTNFASAHDTE-GSMTIDNYV-DESGEEI-NLGGDSVWNFHVWNECWMKRGDLPTG--
YDGWQAVDATPQEI--SLGL--YQTGPAPLTAIKNGEVYLGFETAFVFAEVNSDRTNWI---VK-
QDEGGEYVIETLGSRFPKSVGKYISTKSVGTDDRLDVTNLYKYTEDSAEEREAFKKA-
YAFGTLSVEEE---VSIDFSTAPNIRNGDGFAIVITAQNDSAEKV-TVDISAVLHSTLYTGEKRR-
FIK-RQRFSAIPVDTNSTVSRDFNVMFSDYN-GKLV---DLN-SLRLSAVVKV----KETGK-
FADSYEFRLDNKEAIDIQVEN-TLQINKEYQVLVNFSNPLPTRLTNVVVTLEGPGLS-EPL-
TRKLNRNVVAGGT-AQFSFPIQPKKVGK-KSILVDVDAKQV-KDL-KNFIDVEV
>tunicate_TGML3
LQILRVNYKKET---NCVDHHTDKY--IHNS-LVVRRAATFDLGLVFKN--RN-YRPAK---
DDIVLEFTIG--SDPTIKNETKIRVPVGD-SLQGSKWTCMKIDEVTK--EVTLQVNIPP-

DAIIGRYKLTVEVATELK-
DGRQKERKVKPDIVVLFNPFKPADPVYMESSVEREEYVLNDTGRIYVGQWYRIGAKDWLFGQFEEGIL
DIALKLLREHTNAQKNATKSLKKRASPAYCSRLLSAMVNCND-DNGVLWGRWD--
GKYEEGVKPTTWSGSVAILKQWNQTKM-NPVKYGQCWVFSGLLTTVLRALGI-
PARSITNFNSAHDTE-YNMTIDKFL-TEDGESAEGT-GDSIWNFHVWNEGFFRRPDLPKG--
YDGWQAVDATPQEE--SSGV--MQCGPAPIKAIKNGEIYIGSDTNFVFAEVNADRVFWE---VN-D--
EGE--VTKMVKNDKRHVGRNISTKAVGSDEREDVTLQYKFAEGSEEERVAFERA-YAHGRVVEEEG--
-IKIDINPVGDVINGSDVSISVKVTNAKGVDC-DATITTVIHTMLNNEERKR-LLK-RSR-
GTRKIAAGKDDVESFKFGFGDYG-RHLS---DEN-VIRVTTTVRV----KETNK-
YVDQYDIQIESPQCLELICAD-ELKVREYQPIRFKITNPLKVAMTSAVFSLQGSGIS-SGK-
SFEVPSPIEPGETYTSPEMEVRPYRSSRATTILGDFDCNEI-WNI-KARKRVSV
>human_TGM4
LQVLHIDFLNQD---NAVSHHTWEF--QTSS-PVFRRGQVFHLRLVLN---QP-LQSY----
HQLKLEFSTG--PNPSIAKHTLVVLDPRT-PSDHYNWQATLQNESGK--EVTVAVTSSP-
NAILGKYQLNVKTGNHIL-------
KSEENILYLLFNPWCKEDMVFMPDEDERKEYILNDTGCHYVGAARSIKCKPWNFGQFEKNVLDCCISL
LTES-------SLKPTDRRDPVLVCRAMCAMMSFEK-GQGVLIGNWT--
GDYEGGTAPYKWTGSAPILQQYYNTK--QAVCFGQCWVFAGILTTVLRALGI-
PARSVTGFDSAHDTE-RNLTVDTYV-NENGEKITSMTHDSVWNFHVWTDAWMKRPDLPKG--
YDGWQAVDATPQER--SQGV--FCCGPSPLTAIRKGDIFIVYDTRFVFSEVNGDRLIWL---VKMV--
NGQ-EELHVISMETTSIGKNISTKAVGQDRRRDITYEYKYPEGSSEERQVMDHA-FLLLSPVKENF--
-LHMSVQSDDVL-LGNSVNFTVILKRKTAALQ-NVNILGSFELQLYTGKKMA-KLC-DLN-
KTSQIQG-QVSEVTLTLDSKTYI-NSLAILDDEP-VIRGFIIAEI----VESKE-
MASEVFTSFQYPE-FSIELPN-TGRIGQLLVCNCIFKNTLAIPLTDVKFSLESLGIS-SLQ-TSDH-
GTVQPGET-IQSQIKCTPIKTGP-KKFIVKLSSKQV-KEI-NAQKIVLI
>coelacanth_TGM4
LRIEDVDFLQKK---NGKHHHTDEF--ESPH-LIVRRGQEFKMKLKLD---RE-FRKK----
DKVYFHLALG--QGKVQSSESLIVIELDS-ADSSQLWRATVCQAKGR--KCVVQLSTPP-
DATVGEYHLWVKTDEQYT------
FSPDDNRIYILFNPWCQDDAVFMREGPERREYVLNDTGYIYVGSVQHISARSWNFGQFEEDVLDCCMY
LLNKA-------LPEAAERRDPVKVARKMSALVNSND-DWGILYGNWS--
GSYDDGTPPLTWTGSTAILQQYYKTRK--PVKYGQCWVFSGVLTTVMRSLGI-
PARSVTNFVSAHDTE-GNLKVDIYV-DKNGEYLDDMITDSIWNFHVWNDVWMKRPDLPEG--
YDGWQALDSTPQEK--SKGV--FQCGPSPLPAIKSGEVHLEYDTRFVYAEVNADKIVWL---VKHP--
STAREKRIKLRKNAKAVGKHISTKAVGRNEREDITTQYKFHEGSRMERKNMKAA-CT---PLVSTP--
-VQLGIRGDSSIPVGEPIALTITLTNTTSDSK-AVSLTAACQLQTYAGKTVA-SIG-NLK-
RDTEVGGDQCTEVPMKVEVDAYM-KHLVSSKDDL-LIHVSVVSKT----GDGSE-
DTEDATLIFQYPS-IVAEMPA-TAKIGETVVCTFTFKNQLSIPLDNCKLHVEGLGLF-KLE-TLNQ-
GGIEPGRI-FRSKIIFNPTKTGE-RKIVAKVSSVQI-NGI-AVEKTITV
>clawed_frog_TGM4L
LRIISLDLLKTE---NRSHHHTSTY--TNTS-LILRRGQEFKFKITFS---EE-LTSDI----
PIELHFSTG--TTPLLSNGSLVLIKVNA-ESTKKQWRAKICESNGN--EHVVEVCSPA-
NAIVGIYSLSILTGSTAC-------
FYSTDHIYLLYNPWCEDDDVYMPNEDERQEYVINNTGYIFRGSYKNIKAKPWNFGQFEDNVLDCCMYL
MDIG-------GLKPSSRRNPVIISRKFSAMINSND-DNGVLTGDWS--
GNYPDGTAPVAWTGSSAILKKYYKEKK--PVLYGQCWVFSGVVTTVMRCIGI-
PARCVTCYSAAHDTE-GNLRVDAYY-NESGEPLSHMNKDSVWNFHVWNEVWMKRTDLPQG--
YDGWQVIDATPQER--YQGI--FQCGPCSVTAIKKGHVYLPYEGSFVFAEVNADKTNWM---
VHEYNIKGP---HTLLKEERSSIGKFISTKKVNKSLREDITSQYKFDEGTEEEREAYQRA-
CSYLNAPAVQG---IKVKIHRGHELYPGNPLNALVEVTNITDGDK-TVDVIISCQLQTYTGRITA-
NLT-SAR-KTVEIKKKQVASIPVTIEADLYM-KSVMQVDDEL-AIRVNVITEI----QELRE-
SFKSRIIHFHYPP-IEMEVPE-TVKTGQQITCRFLFDNTLNIKLENCEMNVEALDIFHLQR-VAEW--
DLNPGEA-LSFRIKCRPKITGE-KKIVADFISTQI-KGI-TMEKIIHV
>clawed_frog_TGM4
LKTEKVDFLKTE---NTSQHNTNDY--DNPN-LILRRGQEFHMKITFD---RE-LTAN----
DKVTLQFTTG--SLALPSNGTLVLIDVGP-VIQTNQWSALIRQRNNK--EYLVAVSSPA-
TAIVGKYMLSIITGKGIV------

YPLADCTIYLLCNPWCKDDTVYMPSEDGRKEYVLKDTGYIYVGIATKITAKPWNFGQYEAEVLDCCMF
LLDHG-------QLKPEHRRDPVILTRKLSALVNSND-DRGVLTGNWS--
GNYSGGFSPTSWTGSSVILQKYYKSKR--SVMYGQCWVFSGILTTVLRCLGI-
PARSVTNFNSAHDTG-GNLKVDVYL-NEKGEILEDLCSDSVWNFHVWNDAWMKRPDLPKG--
YDGWQAVDATPQEL--SQGV--YECGPCSLAAVKNGDVYLPYDGKFVFAEINADRICWL---VK-D-
KQGDE-PPIQIRQEKSCIGECISTKTVNMNVREDITVQYKHPEGSPEERETFQKA-CSFLNPPYPPA-
--TKLQIQGDKELIPGNPLSFTVSIENETNEAK-SLDITIGCQLQAYTGKVIA-SVA-SIK-
QSVQVPGKKVAYIPVTVASEQYM-KSVIMVEDEA-IFRINAITEN----KETQE-
TSDSMAIAFTYPP-IKVEMPE-TAKINEDFSCTFTFKNTLSIQLDKCQLHVEGLSMF-KLE-TFDE-
GDIKPGGI-FRSKIICAPRRPGE-KKIVAKLISSQI-KGI-SVEKTIII
>platypus_TGM4
LRVLQVDFLKTQ---NARLHRTEDY--NTPN-LVVRRGQAFQLKLVLD---RQ-LEDK----
DNVELQFCMG--KAPRKSNETLVVMKLRG-QKELHRWQMALLGVNGK--ECLLSVSSPV-
DTAVGKYFLSVKSGADPV------
HTPENNTIYFIFNPWCEGDTVFMAGDKERSEYVLNDTGYIYVGSKDKIHARPWNYGQFEEDILDACMF
LLDKS-------NLKPRDRKNPVIVSRAMSALVNAND-DCGVLLANWS--
GMYKGGTSPLAWTGSTPILQQYFRTQK--TVLYGQCWVFAGVLTTVMRSLGV-
PSRTVTNFASAHDTE-QNLRVDVYL-NEKGQKIEDLTTDSIWNFHVWTDVWMTRPDLPQG--
YEGWQAVDATPQEI--SQGV--FQCGPAPLKAIRQGDVYLTYDTKFIFAEVNADKVFWL---VKEV--
NGV-SEMTKLREETLVIGRSISTKAVGSNTREDITAQYKFTEGSPEERKAMEKA-CSYLSAPTEEA--
-IELKIQAGEAVWLGDPIRMSVIINNSSARSW-TIKLGATCELQLYTGKVVA-VLG-SVS-
ETVATAGKPVIEVPVHLEASSYV-GSLASVDDEV-IVQGHVIAAI----EETEE-
FSEEVTLTFQYPP-LRVEVPE-TVKIGQNFTCAFIFKNTLSIPLENCKLHVEGLGIF-MLE-TFDQ-
GDVLPGGI-FKCRIICTPKKAGK-KTIIAKLNSIQV-KEI-YTEKMVTV
>chicken_TGM4
LKVTKVDFLKSQ---NSVQHHTDAY--NTSN-LVVRRGQPFLLQLTLS---RE-LRAA----
DKLSLHFSIG--ERPMEPTGTLMSLNPRS-TRNVSGWQIAIIKSSGT--ECTLSVTSAP-
NAAVGIYGLMVKTGPNIY-------
KPEKNTVYLLFNPWCEGDIVFLSNEAERKEYVLNDTGYIYVGSAFNIHSKPWNFGQFEESILDACMYL
LDKS-------KLKMSSRRDPVVVSRAMSALVNAND-DNGVVLGNWS--
GKYENGTSPMAWIGSVAILQQYYKTK--KPVSYGQCWVFSGVLTTVMRCLGI-
PARSVSNFNSAHDTD-ENLRVDVYL-NEKGEKLKWMSSDSVWNFHVWNDVWMKRKDLPSG--
FDGWQAIDATPQEQ--SQGT--FQCGPCPLKAVKEGDVYLPYDSKFVYAEVNADKVYWR---VKEE--
NGR-NKYTKLGVESQSIGANISTKAVGQNRREDITWQYKFPEGSAEERASMKRA-VSYLQEVVPKS--
-VQLEITNEKPLCPGNPIEVTITVKSTVAGSW-TVDLASSCQLQSYTGKVHA-NLGYVKQ--
TVKVEGQSEVHVPLKIMPDAYM-KALATVDDEE-HVHVTAIAEI----QGTPE-LTKEASLSFEYPP-
IQVQMPE-TAKVNNDFTCAFIFKNKLNVPLDNCKLMVEGLGIF-KMA-TFDE-GDIQPGRI-
IKSEVICTPTRVGE-KKIVARLTSNQV-KDI-SVEKAITV
>lizard_TGM4
LKAVGVDFLRKE---NVCLHHTYDY--DNCS-LVARRGQLFSMKLSFN---RV-LNDN----
DNVILQLSTG--DKPMESTGTLMRLPMGS-LQRNQSWHANICETNGK--ECLIAVTSPA-
DAIIGKYVVKVDTGASIY--------
SCNEHFYLLFNPWCEADSVFLPDDDERTEYVLNDTGYIYIGSTKSIRGRPWNFGQFEKDILDCCMYLL
DKS-------QLKPNARKDPVIISRTMSALVNSQD-DRGVLYASWS--
GKYTSGTSPLAWTGSVPILQQYYKTQ--KSVLFGQCWVFSGVLTTVMRCLGI-
PARSVTNFASAHDTE-ENLKVDVFL-NEKGEKLNNLTKDSVWNFHVWNDVWMKRPDLPKG--
FDGWQAIDATPQEV--SQGI--YQCGPTPISAIKKGEVYLPYDSKFVFAEVNADKVYWV---VK-N--
EGGEEKYIKLREETKVIGKSISTKAVGKNVREDITDQYKYPEESSEERKAVETA-CCYLQSAQDSV--
-LKLGTEGDQALWPGQPIELNIVVRNDSLGTW-TINFAASCQLESYTGNVEA-SLA-TVK-
QTIKTEGKPVIEIPLKIAADAYM-KTLMVVEDEL-LVKINIIADV----QETNE-
FMEELYFNFEYPP-LKVEMAE-SAKVNEDFTCAFIFKNTLSISLENCKLHVEGLGLF-TME-TFDQ-
GDLAPGRI-FKCKIVCAPRKTGL-KKIVAKLTSNQI-RGI-TTEKMICI
>zebrafish_F13A
LSVLSIDMRVTE---NKNVHHTDTY--KGSN-LIVRRNTEFTIILKLD---RA-FNEQQ---
HQVELEFLIG--SSPDENKGTYIIVSIGK-EKQDSSWKSRVVAMHGN--SVMVGITPDA-
KCIIGRFRIFAVVVSVLG--
KERTQRNPDTDFYVLFNPWNSEDEVYMDKEEDRQEYVMNEVGTIYNGVHNNITTRSWNFGQFEEGVLD

```
ACLTVMDAG-------NVPLLFRGNATEVVRQASALMNSQG-DNGVLVGSWS--
GDYSSGTAPTAWTGSPEILLKYASEGC-VPVCFAQCWVFAGTLNTFVRCLGI-
PGRVVTNYCSAHDNT-GNLKTDIVL-DEDGRMDKSRTRDSVWNYHCWNEVFMKRLDLPDQ--
YSGWQVVDCTPQET--SDGL--FRCGPTSVNAIKEGELSYPFDARFVFAELNSDVIYHK---SN-K--
YGK---TKIIHVDTSYVGKQLVTKKHDSNDYMDITSSYKYSEASLKERQVMQMA-ERRGVPEAGVE--
-IQIQ---TNTIKIGDDFRLTMNIKNKSSKAC-TVIATVTGCVVFYTGITGS-DFKLENK--
KASVQASKTEPLTIDIKAVDYT-PHLV---EQA-NLLFVVYGIV----EETET-LTTMRVINLQLPE-
LTIKMSG-SPRVGSDLLVSVEFTNPYNFPLLKVELRLDGPGLI-QTK-VKHY-SQILPGAS-
VNYTVSIVPRAHGK-KVLMACLDCSAL-RQV-TNQLEFEV
>zebrafish_F13A1a.1
LSVQSLDMHITE---NKQAHNTSMY--KNSS-LIVRRNKEFLIDILFD---RP-FDETQ---
DTVQLEFMIG--SVPDENKGTYITVSFGS-VKTESSWRGRLLEKQGN--SIRVGITPDV-
QSIIGRFSTFAVVVNETG--
KRRTEKNSATDFYVLFNPWDPSDQVYMPNEAERQEYVMNDVGTIYNGEINDISFRSWNYGQFEEGVLD
ACLFILDSG-------KMPLMYRGNATEVARQASALMNSID-DNGVLAGNWT--
GDYSSGTAPTAWTGSAEILLKYASKGG-APVCFAQCWVFAGTLNTFFRALGI-
PARVITNYCSAHDNG-GNIKANIML-NPDGSVNRKKTRDSIWNYHCWNEVFMKRFDLPDQ--
YSGWQVADCTPQET--SDGL--YRCGPTSVKCIKEGDLSYSFDSRFVFAEVNSDVVFHQ---FD-K--
YGN---SKIVHVDTTYVGQLIVTKRPNTNGYIDITLNYKYPKGSAEDKRVMQLA-ERRGISPLPDA--
-VQIDIKAE-TIKIGENFTLTMNIKNQTSQTS-TVSLTVTGCAMYYTGLTSS-TFK-LEN-
YSSTVDAWQTTPVTMKVQAAEYM-SFLV---EQS-NLLFVVHGQV----NETGK-
VSAMRVINLRPPE-LMMKVTG-VPQVGRDLMVSVSFQNPYNFTLKNVQLRLDGAGLT-PTK-VKSY-
DQVAPGGS-VQYTDTITPYSPGR-KVLIGCLDCIPL-SQI-TNQLEINV
>zebrafish_F13A1b
LDIFDVDVLKQP---NKQAHHTHLY--SSNF-LIVRRAQEFQIKITFN---RP-YKPAE---
DKFAVEFVIG--PVPQYSKGTYIPVFPTA--KRQSVWSGRVIESSEN--VVTMGITPSA-
ECIVGKYMTYIGVETPYG--
IRRTRRDPNTDIYILFNPWSPADPVFLDDEEEREECVMNELGIIYHGAYDDVSERAWNYGQFEFGVLD
ACLFVMDKA-------DMPLSNRGDVVKVTRVASAMLNSRD-DDGVLVGNWS--
GDYMYGVPPTSWTGSVEILLDYANSSG-TPVCYAQCWVYAAVFNTFLRCLGI-
PSRVVTNFFSAHDND-GNLKMDIIL-DENGKLDRNRTKDSIWNYHCWNECYMARPDLPSG--
FGGWQVVDATPQET--SDGM--FRCGPASVAAIKHGQICYPFDAPFVFAEVNSDVVFYR---RR-K--
DGI---LEVVKVNQTHVGRMVLTKAVLHSGRRDITNQYKFPEGSPEERRVLEKA-EEFGCPALSDV--
-VEIH---SLDVNVGENFDVTLQFTNRSDQRR-TADVYITGTVVYYTGVPSG-EVVFKTP--
KVKLEPMQSKEEKVLVRSEDYM-NKLV---EQR-NIHFIATGKI----KETGQ-ITAMKVIAMHHPK-
LTVKVTG-SPRVSEEMYVSVEFTNPFKFSLENVDLRVEGPGVL-PFK-YKQY-SVIAPGTS-
ITWTEAFSPRRAGS-TKVFAKLDCAAL-RQV-YGETELTV
>shark_F13A1
LEVWNVDVHPNPDDVNKKQHHTELY--DCRN-LIVRRGQPFQITITFN---RA-YDSGK---
DKLWVEFLIG--QYPDITKQTYAPVYIQE-QLEKGKWGAMVTSTRTN--TLSLSIVSPP-
HCIVGRFRMYLAVMTPYG--
IRRTARDSETDIYVIFNPWCREDAVYLDNDRENEEYVLNDAGRIYYGKFTEIISRPWIFGQFERGILD
SCIYILDRA-------RMPLQTRSCPIKVSRVASAMINSKD-DGGVLVGCWD--
GIYTNGVAPTAWNSSVEILLQYFETQL--PVCYGQCWVFAAVFNTVLRCLGI-
PARLVTNFSSAHDNN-ANLTTDIIL-DENGKKDMNLTKDSIWNYHCWNECWMSRYDLPPG--
YDGWQVVDATPQET--SEGM--FRCGPASVNAIKHGQVYFPYDAPFIYAEVNSDVVYWN---RQ-K--
DGS---LIKGNVKTDEVGMLILTQEIGSDGRKDITDQYKYPEGSQADRAAQGTA-IQYGIK--AVD--
-ATLNVHVPEKILLGTTFDVGIELQNNSSEKQ-HMTLHLNGCVVFYTGVPKI-KIK-DKT-
IKAMVEPHQVYQTQVKIKSKDYE-DHLV---EQS-ILHFLVSGHV----
AETEQTLAAQKIVTLQIPQ-LNLRAEG-PAVYGRETIIIIEFTNPLKKPLEEVILRVDGLDTL-KPK-
LKIF-SSIPESAT-LTTKELFVPWRRGT-RKVIASLDCKAL-RQV-VGELELNV
>clawed_frog_F13A1
LEVQNIHLFKQT---NKREHHTDRY--YNNK-LIIRRGQTFDIQIDFN---RN-YNPEK---
DQFWIEYVIG--RYPQQSKGTYIPIPIVD-KLEAGKWGAQVTRNEYN--SVSVTILSAP-
DCIVGKFRMYVAVLTPYG--
IMRTSRNSETDTYILFNPWCKEDSVYMDDEREIEEYVMNDVGIIFHGEVSNIKKRSWEYGQFEEQILD
ACLNLMDRA-------QLDLSGRGNPIKISRVGSAVINSKD-DDGVVAGNWK--
```

NDYSFGVSPSAWTGSVDILLEYHSSGL--PVKYGQCWVFAGVFNTFLRCLGI-
PARLITNYFSAHDND-ANLRTDVYL-DEDGKTNTTLTKDSIWNYHCWNEAWMTRSDLPVG--
FGGWQAVDATPQET--SDGM--FRCGPASVQAIKHGHACFQFDTPFIFSEVNSDIVYSR---AM-K--
DGS---KVIEHIDKTHVGQKILTKQIGGDDPQDITDLYKFSEGTEEERLALEAA-LMYGVQQQFAP--
-TDMDFQAD-NAVFGSDFKVTLTFKNWSANLY-TVSAFLTGHIVFYTGVLKS-EFK-NKN-
VEVTLDPSSSKSVEVLIKAHEYL-SKLV---EQA-SLHFFVTARI----NETKK-
MAKQKIVILKVPE-LHIKVLG-EKMVGKEMTIVIEFTNPLKKALQNVALRLGGPGLM-RTR-AKVF-
GEIPMNSS-LTWEQKCIPQRAGT-RKLIATLDCETI-RHV-YGELELEV
>chicken_F13A1
LRVVDIHMFKEP---NKQQHHTDKY--YNPK-LIVRRGQPFQIQIDFS---RP-YDPEK---
DQIWLEYLIG--RYPQPNKGTYIPILIGD-VLKPGEWGAKITHRENN--SIRLSIMSSA-
TCIIGKFRLYIAIWTPYG--
IIRTHRNSATDTYILFNPWCQLDAVYLDDEKEREEYVLNDVGIVFHGDVNEVKLRSWSYGQFEENILD
ACLFLMDKA-------ELELSGRGNPIKICRVASAMINSKD-DNGVLAGSWD--
NLYDYGVPPSAWTGSVDILLEYYSSKQ--PVRYGQCWVFAGVFNTFLRCLGI-
PARLITNYSSAHDNN-ANLQLDFFL-DDEGQVDNRLTKDSVWNYHCWNEAWMTRPDLPVG--
FGGWQAVDGTPQET--SDGM--YRCGPASVQAIKHGHVCFQFDAPFVYAEVNSDIIYSR---MG-K--
NGS---QVIEKIDTTHIGKLIVTKGVGNDDMVDITENYKFQEGSAEERLALETA-VMYGVTYQPQK--
-IEMDLQVQ-KAVLGSDFKVTIILRNKSRNSY-TATTYLSGNIVFYTGVTKS-EFK-KHS-
FSAKLEPLLSNTFDVMITSAEYL-NDLL---DQA-SFHFFVTARI----NETGK-
LAMQKAVVLEIPT-LKIKTKG-QMVVDREMSVVVEFTNPLKQTLENATLRLEGPGVL-RTM-KKEF-
RQIPAMST-LIWDVKCIPKRPGL-RKLIASLNCDAL-RHV-YGELNIQV
>lizard_F13A1
LEVLEVRTFNRE---NKKEHHTDKY--SNHK-LIVRRGQPYHIQISFN---RP-YNPEK---
DTFWIEYLIG--RYPQQAKGTYIKVQLVE-KLQSKEWGAKITHSDGQ--FVALNIMPAA-
NCVVGKFRMYIAISTPIG--
ILRTRRNPATDTYILFNPWCTEDLVYLDDEKQRQEYVLNDLGVIFYGDPENIRSRSWNYGQFEEGVLD
ACLYVMDRA-------ELDLSGRWNPLKISRVGSAMINAKD-DEGVIVGSWS--
NVYDYGVAPSAWTGSVDILLEYHSSRE--PVRYGQCWVFAGVFNTFLRCLGI-
PARVVTNFCSAHDND-ANLQMDVFV-DENGKVDSKLTKDSIWNYHCWNEAWMSRPELPVG--
FGGWQAVDSTPQEN--SDGM--YRCGPASVQAIKHGHVCFPFDTPFVFAEVNSDVVYSK---AM-Q--
NGV---KLVQHVDKTQIGRLIVTKEEGTDKMKIITEQYKFQEGTEEERLALETA-LMYGVTSTL----
-VNMDFVVE-DPTLGSDFNVTITFQNNTQSRY-TATSYLSGNIVFYTGVSKN-EFK-NHS-
FNVTLEPMKAQTVEVLIKSSEYM-SQLL---EQA-SLHFFVTARV----NETQK-
LAQQKSVALKIPQ-LLLKVPG-EKVVGKDMAVIVEFTNPLKEDLNNVWVRLDGPGLL-KPT-SKLF-
REVPRNST-LTWEEKCLPKRAGL-RKLIASLNCDAL-RHV-YGELDINI
>human_F13A1
LNVTSVHLFKER---NKVDHHTDKY--ENNK-LIVRRGQSFYVQIDFS---RP-YDPRR---
DLFRVEYVIG--RYPQENKGTYIPVPIVS-ELQSGKWGAKIVMREDR--SVRLSIQSSP-
KCIVGKFRMYVAVWTPYG--
VLRTSRNPETDTYILFNPWCEDDAVYLDNEKEREEYVLNDIGVIFYGEVNDIKTRSWSYGQFEDGILD
TCLYVMDRA-------QMDLSGRGNPIKVSRVGSAMVNAKD-DEGVLVGSWD--
NIYAYGVPPSAWTGSVDILLEYRSSEN--PVRYGQCWVFAGVFNTFLRCLGI-
PARIVTNYFSAHDND-ANLQMDIFL-EEDGNVNSKLTKDSVWNYHCWNEAWMTRPDLPVG--
FGGWQAVDSTPQEN--SDGM--YRCGPASVQAIKHGHVCFQFDAPFVFAEVNSDLIYIT---AK-K--
DGT---HVVENVDATHIGKLIVTKQIGGDGMMDITDTYKFQEGQEEERLALETA-LMYGAVMKSRS--
-VDMDFEVE-NAVLGKDFKLSITFRNNSHNRY-TITAYLSANITFYTGVPKA-EFK-KET-
FDVTLEPLSFKKEAVLIQAGEYM-GQLL---EQA-SLHFFVTARI----NETRD-
LAKQKSTVLTIPE-IIIKVRG-TQVVGSDMTVTVEFTNPLKETLRNVWVHLDGPGVT-RPM-KKMF-
REIRPNST-VQWEEVCRPWVSGH-RKLIASMSSDSL-RHV-YGELDVQI
>platypus_F13A1
LNVTDIHLFKDS---NKQEHHTDKF--INNS-LIVRRGQPFCIQIDFN---RP-YIPSR---
DLFRLEFVIG--RYPQENKGTYIPVPLVS-ELQSEKWGAKIILKEDR--SLRLSIQSSP-
HCIVGKFRLYVAIWTPYG--
IIRTQRNPERDVYILFNPWCEEDAVYLEDEKEREEYVLNDLGVIFHGEVNNVKSRSWNFGQFEEGILD
ACLYLMDKA-------KMELSGRGNPVKISRVGAAMINAKD-DEGVLVGSWD--
NVYAYGVPPSTWTGSVDILLEYHSTGN--PVQYGQCWVFAGVFNTFLRCIGI-

PARVVTNYFSAHDNN-ANLQVDIFL-NEDGKTNTRISKDSVWNYHCWNEAWMTRPDLPVG--
FGGWQAVDGTPQEN--SDGM--YRCGPASVQAIKHGHVCFPFDAPFVFAEVNSDIVYIR---VK-Q--
DGT---HVVESVDATHVGKFILTKQIGGDGQQDITDQYKFQEGQEEERLALETA-LMYGALIALRD--
-TDMNFEVE-NVVLGRDFKVTITFHNRSPRRY-SATAYLSGNIVFYTGVAQT-EFK-NHT-
FEVTLEPLSLKKVEVAVKAGEYM-TQLL---EQA-CLHFFLTARI----NETKK-
LAKQKSVVLQIPK-VNIKVRG-EKMIGSDLVVEAEFVNPLKQTLQNVSVHMGGPGVM-RTA-KRTF-
REILPNST-LKWEEICRPWTPGY-RKIIASLNCDAI-RHV-YGELELEI
>coelacanth_F13A1
LEVWRVNVLKNP---NKKEHRTDKF--ICRN-LVVRRGQPFQMKITFN---RA-YNPET---
DVVWLDILIG--RYPDVRKSTWIQVRLNQ-ELEKGKWGTKVVESIDN--NVTLSVTSSS-
DCIVGKFRMYIAVYTSFG--IRRTARDRDTDIYVLFNPWSKE--------------------------
---VINSRD-
DDGVIVGNWT--DNYSYGLAPTAWTGSGDILLQYLSSGA--SVCYGQCWVFAAVLNTFLRCLGI-
PGRVVTNFYSAHDND-GNLVMDVVL-DEDGKTNKKLTKDTVWNFHCWNECWMARSDLPAG--
FGGWQVIDATPQET--SEGT--YRCGPASVHAVKHGHVFFPYDAPFVFAEVNSDIVYWK---RM-K--
DGT---RTVVKIDSCHIGKMILTKEVGSDERKDITELYKFPEGSNEERLALESA-MKYGAF--PVD--
-VYMEVYTEKNVTISNDVKLTLEFRNNSQEGR-AINVFIVGYIVFYTGVKKD-AFM-DQT-
LSVVVEPGYFKNVDVHVKAKDYM-SHLV---EQA-SLHFIVTARV----NETTQ-
LTAQCVVPLRSFK-LNLKIDG-KPELGNQMSVTVEFQNPLKKPLENVSVRLEGVGLM-RTK-IKEY-
STIPMNES-IKWTEMFVPAKPGL-RKMIASLDCVAL-RQV-YGEIQIMI
>lungfish_F13A1
LEVLSVDLLRHP---NKQQHHTDKY--DYLN-LIVRRGQNFTMKIKFN---RE-YISNK---
DQFWVEYLIG--RSPRIDNGTYIVAPLTE-ELKKDKWGAKILSIRDE--TLTLAIQSAP-
DCIVGKFRMYIAVMTPFG--
IRRTPRDPGTDTYIIFNPWCKDDTVYMENDAEKEECVLNDVGIIYHGEYNNIQHRNWNFGQFESQILD
SCLSLMDRA-------EMPLSSRGNPVKISRVASAMINAKD-DDGVILGNWT--
GEYLYGLSPSAWTGSQDILLEYYNSHR--SVRYGQCWVFAGVFNTFMRCLGI-
PGRVVTNYVSSHDND-GNLITDIIL-DEEGKVDKAQAQDSYWNYHCWNECWMARSDLPEG--
FGGWQVVDATPQET--SDGM--YRCGPASVKAIKHGFVYLPFDSPFVYAEVNSDIVYWK---RW-R--
NGR---KEIVDIVGNQIGKLVLTKENGKNAQMDITDQYKFPEGSEEEAIALKAA-LMYGTA--SKD--
-CTLQVVVDSKVPFGSDFNIKMDFGNNSRQTL-NIKAYVSGNIVYYTGVTKR-EFK-SST-
FEFNLDSGNYKKETVLIKSKDYM-NHIV---EQA-FLQFITTASI----EPSGQ-
VTSLNVVSLIVPK-LSIKVLG-EPVTRNEITILVEFTNPVKRPLENVNLRIEMPGNM-RSK-TTKY-
SYISPNET-VSWAVKFVPWRPGL-HLCFASLDCDAL-RQV-YGETEINV
>lamprey_F13A1L
LAVLSVDQLIAA---NRARHRTSDY--EVDN-LVLRRGEPFDLQLKLN---RP-YDPIR---
DKIRLELQIG--RFPLESRGSLVVAEVSG-WNSESRFGATVSRCSGN--ELTISMNLAP-
NCIVGRFEMYVVTESDGQ--
PNRTRRDPNTDTYVLFNPWCREDQVFMSDESWRREYVLNDTGCLYLGTSQQIVTQHWNFGQFERGILD
VCLSLLDKG-------DLGMSFRGSPIAVSRTTSAILNAND-DXGVLMGRWS--
GNYRNGVPPTAWNGSVDILLRYGRVG--RPVRYGQCWVFSGLLTTVLRCLGI-
PCRSISNFSSMHDQN-ANLTMDTYL-DQDMWPIDSLNHDSIWNFHVWNEAWMARPDLPGD--
NGGWQIVDATPQEQ--SNGL--MQCGPAPQVAVKNGHVHIKHDTPFVFAEVNGDRVYWV---WG-K--
DWR---FHKVYTDTSVIGKDISTKAVGSMRREDITLQYKFPEGSREERDTMALA-LAGGIASPPAS--
-LALSFAIGDAA-VGSDFAVRVRVANGKAATA-TVTVHVQGDFASYTGASRQ-RFK-RDR-
ATAVLEPGETGELEFPVLVSDYL-LHLS---EHG-TMNFTASARV----EETEN-
VTKVRAVSLRAPT-IDIAAKG-EARVDTEMHAELTFKNPLPVCLTDVSFNMEGPSLL-IPT-IRTF-
RNINAGES-VSLSQSFTPTKAGE-RTLMASLQCKEL-YIV-TGTLELAV
>lancelet_TGM1L2
LKVTNIDFREIG---NRLAHHTDEF--EKQV-LIVRRGQLFDIGLDLN---RP-YSQAK---
DKIILEFRIG--KYPKPSKGTLIKVTLGK-ELTTDKWGAKVQQTKGN--FVGISVMAPA-
NAIVGKFDFYVQTEHEGT--
KFRTAKDDKNAVIVLFNPWCRDDQVYIDDKAKLNEYVMNETGFIYVGNKRKIRGRPWNFGQFDDPVLD
ASLYLLDRA-------RMAYTSRWNPINITRVLTAQINSLD-DDGVLVGNWS--
GDYDDGVEPWVWNGSVKILEQFLKTK--ESVQYGQCWVFSGVTTSVLRCLGI-
PARSVTNFGSAHDTD-SNLTVDYHF-DDKDEPIEDLNNDSIWNFHVWNECWMARPDLPAG--
YGGWQAFDSTPQET--SEGQ--YCCGPASLNAIKNGHVYYGYDTKFIFAEVNADRIYWR---VP-E--

DGD--DWEQLRVEKYSVGCHISTKMVGSDEREDITHLYKHPEGTELERVAVREA-VEHGNSIPED---
-VTFSIDTDDDVDIGEDFSVRVKLTNQSDQER-YVTMYLTAQAMYYTGVSLG-KF-WNQR-
FTVRLTPNGDGEVKGRIDADLYV-PRLV---DQG-GIKFFLMGHV----RETQH-
FAGQHDFRLGVPD-LTLKVSD-KINIGQEVHVNVSFKNPINTALTKSAFYFEGPGLTADDK-VIHH-
KDIGPRET-ATAVVKLKPKKKGK-RKLMVSFTSKQL-QQV-CGECVLDI
>lancelet_TGM1L6
LQVSDVYFRLSY---NRRAHHTEEY--ESFQ-LIVRRGQPFQLSLAFD---KP-YTEEN---
SQVTLEFHTG--DSPKISDGTLARVPVDS-PLGGTSWGAKVTDKGLK--TIDLLVQAPP-
NAIVGKYSIVVETILDGH--
KYRTERDPYSHIYILFNPWCKDDSVYLNDEEELQEYIMNESGIIYVGNKRQVMERPWNYGQFEMPVLD
ASLELLDKA-------KFPPRARWDPIRLVRVVSKMVNSQD-EDGVLVGNWS--
GEYEDGIRPSAWNGSVEILQQYHVRK--EPVCYGQCWVFAGVTTSVLRSLGI-
PSRSVTNFASAHDTD-GNLVVDIHY-DEEFTPIDYLNTDSTWNFHVWNEAWMTRPDLPAG--
YGGWQAFDATPQET--SEGH--YKCGPASVHAIKNGHVYYGYDAKFIFAEVNADSISWV---CP-E--
DGP---MYQVWQQEKGIGVKISTKAVGKDEREDITDHYKYSEGTEEERLAVEKA-VSHGSPEKA-D--
-VEFSINAPKEVIVGHDFTASVTLRNHSNQER-SVSMFLTAHTMYYTGVPVT-QFH-KEK-
FVVTLQPNGQMTLHAQIEPAQYM-SYLV---DQG-AIKLNLLGRV----KENKC-
FAGQTDFRFSVPK-LNIHIPA-RERVGEEFVVELSFKNPLAVKLTNTEFHVEGPGIQ-KPK-LISY-
RDVRPLEA-ARTLVTMKPVRPGS-RKIIANFQSKQL-HDV-QGHLEIQV
>lancelet_F13A1L
LRINKVDFLREQ---NAPLHHTDEY--DSPL-VVVRRGAPFLLKLGFD---HG-FDRLK---
DNIVLEFRTGNTDKPFANKGTEVIVRVGD-GPGDHGWEAELSSVKEGEPWAVVSVTPPA-
QCPVAKWRMSVETISEGL----
SYETRYDSELVILFNPWCEDDEVYLEGDDKRGEYVLREFGKVYQSWKKVFNGKPWNYGQFEPEILEAC
LGLLDRS-------GVSVMSRGSPVAVTRVVSQMVNSQD-DNGVLEGNWS--
GDYSGGVAPWIWNGSVRILQQYHRTK--QPVSYGQCWVFSAVTTTVLRCLGI-
PSRSVTNFSSAHDTD-GSLTIDKVV-DEYGNLLED--SDSVWNFHVWNEAWMARTDLPKG--
YGGWQALDATPQEK--SSGI--FCCGPASVNAIKHGEVQYNFDARFIFAEVNADKVHWC---QQ-R--
DGS---CYVAGMEKGTIGRFISTKAVGINDREDITENYKFPEGSEEERVAVQRA-AQYGSYQSATS--
-VEFSLKDDGSVPINEDADCTITLKNTSSEIR-NVTLLYVANIVKYTGVQHR-KLR-KEK-
IEVELKPKSTESVNVAVNPMESM-DHME---DKC-ALKFLFMAHV----QETGQ-
YATERVVDIDFPD-LDVKVLG-KTDLGQQVAAAITFTNPLDRVLTNCEFNIEGPGLQ-KPK-TVFF-
RNIRPGET-VKIEERFTPKRAGL-KEIVANFSSSEL-CDI-TGEAEVQV
>lancelet_TGM1L1
LKLTNIDFLNDE---NGRAHHTDEF--EEHN--VVRRGQPFTVELRFE---KT-YDEGE---
DKLKVELHFG--DNPLPNKGTLLRMPVGK-SLEDGKFSAALDSSEGG--YAKVKITTPP-
DAIVGKYHVVIETMSDGK--
TFRSRKTDDNTVVVLFNPWVKDDMTYLDDDAQLGEYVLNEHGYQYYGTSRRIGKRPWNFGQFEPKILD
VCLRLLDRS-------QIKAESRGDAVKVSRIVSKMVNSAD-DGGVLTGNWS--
GNYAGGRSPTAWNGSVEILRQYYERN--RPVCYGQCWVFSGVMTTVLRCLGI-
PARSVTNYDSAHDTD-VSMTIDNYM-DHNLREIDG--GDSVWNFHVWNEAWMARPDLPEG--
YGGWQAVDATPQET--SDGV--YCCGPCPISAVKNGHVYLPYDTKFVFAEVNADKVYWL---VD-R--
HKN---LRKLRTRKAAIGNKMSTKAVGSNARHDLTENYKYPEGSDQERVAVRTA-VSHGLIEDE-D--
-VEFDVHADDEIYIGQNIHVTLSMKNTSSEPR-KVTTHLTARAMYYTGVPAH-DIG-ELE-
KDVFIPPNGEASVEMTFTPREYL-DMLV---DQA-IVKIHAMAHV----DDTNQ-
YSGQDDFRLMSPD-LTVKAPT-KMTLGEQVTAEIEFTNPLDVTLSMVEFHIEGPGLQ-KPK-KISH-
TPIKPGET-VKVTERMTPRKVGK-KTIMASFTSNKL-TQV-TGELDVVV
>lungfish_TGM1L
LSVKSINFLKKP---NRLSHHTEKY--VTED-LPVRRGNNFQIKIECN---LS-FDVKT---
DHFQLEFVHE-SKNPALKTERPIVVYIKE-QLDKKNWGAMVVGSSGN--YVTLSVYSPP-
NAPIGKFILRVKSKRSPG--
DFSTYSDPKTHICILFNPWCPDDTVFMNNEQWRQEYVLNEFGIIYRGSVNQNSSMKWSYAQFAKSIMD
ACYYTVDRA-------GLTLSERKDPVVVARKMSAMVNALD-DEGILVGNWS--
SDYSGGTPPTLWIGSEAILLQYYKTG--RPVRYAQCWVFAAVVTTILRSLGI-
LCRTITNFPSAHDTE-KNLTIDVYL-DEEFKPLSEMNNDSIWNFHVWNEIWICRPDLPAG--
FNEWQAIDATPQEK--SDGI--YCCGPAPVKAIKEGKIDILYDSDFICSEVNIDKVYKQ---KQ-K--
NGT---FSSVYVERNVAGIKILTKAVGSNGTQDLTNQYKYPKDSKEGMSAIASA--THRLVTIPNS--

-VEITACTDGTTDIGSPISFHVKLHNKSQEQY-TGILHIHASMMQYNGAIQD-PFRIDEN-
KEVVLEASQETDIEMAIEYEEYR-DYLT---EQA-CMMFTVGGQV----LETRQ-
LVLQSTLSLRKPG-LVIKTVG-VVAVGKESKAEFIFKNNFPFCLTDVLIRIAGPGLF-LPI-VLNV-
GTIGEESN-SVTGYTFIPTQAGP-RKLLASLHSNEL-TDV-TGSVDIDV
>lamprey_TGM1L1
LAVLRVESAGKE---AAREHRTGGF--AQPQ-LVLRRGAPFRLRLKLS---RA-VQRHH---
DSIVLEFSTG--LSPQLRKGTLIQVPLLP-PQRDGGWSAVVEREEER--ALHVLVRSCP-
RSVVARYEVAACAHSETAVEEVRSKREPLS-
VYILFNPWCREDTVYMESEEERQEYVLRETGLIYYGTKEQISARPWVFGQFGKGILDTCLHLLDRA--
-----NMPLYGRNDPVNVARVVCGIVNSQD-DSGVLEGNWS--
GDYTEGTSPAAWTGSAEILLGYHKGARPAPVRFGQCWVFSGVTTTVMRCLGI-
PCRSVTNFCSAHDTD-VSLTTDVYV-DEKMKPIDDLNTDSIWNFHVWNECWMARPDLPMG--
YGGWQVIDTTPQET--SLGF--YRCGPASVNAVRDGQVQHKYDAPFVFAEVNSDRVYWM---KQ-P--
GGE---FSVLSVDKQAVGHCISTKAVGSHEREDITGLYKHPEGSKEERIAVETA-CSQGSGTAQDV--
-LLVKPPVD-EVALGRDFAVTLELANLDADETRTVVLFCAAHGMHYTGVLRG-RVK-QHS-
WEVQLAPGEECTLSLPVSASEYL-QSLV---DQC-AMLFTVTGRV----GETGQ-
LATQRSFRVNFPA-LGVTVDD-RVRVGNKALVKVSFTNPLPQPLRAVTLRLEAAGLL-EPT-LIQH-
GDVPGGGS-VYRTVAVVPRVAGV-ATLLATLDSHQL-ITV-HGELELLV
>lamprey_TGM1L4
LAVLRVESAGKE---AAREHRTSGF--AQPQ-LVLRRGAPFRLRLQLS---RA-VQRHH---
DSIVLEFSTE-------TAGPPVSVPVGP-RLREETWSATLSGRGGQGVEIEVTVQSPP-
HCPVGQYRLAVAVSPAGG-
RPVRTEPGVCPDVIILFNPWCRGDAVFMEPEEHKTEFVLNETGRLFYGTAQQIGTRAWNYGQFTSGVL
DASLHLLS---------SMAWEQRGDPVAVVRTVSALVNAND-
ESGVLEGNWSERNDYSGGTAPAAWTGSPDILRQYHRSG--RPVKYGQCWVFAAVTTTVLRALGI-
PCRTVTNFSSAHDTD-GNLVTDIFL-DEKMRPDRSHTKDSIWNFHSWSDAWMKRPDLPSG--
YDGWQVVDATPQEA--SAGV--FRCGPAPVMAVKKGDIDLKYDTAFVFAEVNSDRVFWR---RR-A--
GGE---RERISVETDSIGQKISTKAPGSDARLDITAEYKPSEGSEEERRVVTHA-LSLLKGDPPPP--
-VTLAVRVLEETALGRELTAEVRVASASARPRVHVRVRVRGHGATYTGANLG-GFE-EEE-
ALLELGPHEERRVLVRVPASEYL-HLAV---DHF-ALVLVACARV----LDTGA-
VARVHVARVRSPR-LRVHVPE-GAHVGRDLISELCFTNPVARALHRVRVRVEGAGVH-----------
--TPGTI---------------------------L-----VGYGAVVT
>lamprey_TGM1L2
LGVWAVELHGEQ---TRLEHRTERY--ETPL-PVLRRGLPFPVTITFS---RP-FEPRS---
DRLQLELYIG--KYPRIDRGTYIPIPLGE-EVEPGTWKAKVTGSEGA--RLALLVHTSP-
KCIVGKYRFHVATLSAGG--
LYRSSRDPNTDLYFIFNPWCPEDSVYMESEAEREEYVLNDIGRIFHGTRDQIVSRVWNFGQYEYGVLD
AALSVLDCG-------RLPLPGRDNPVTVARVASAAVNSQD-DNGVVEGCWV--
DNYTGGVAPTAWNGSAEILLDFKMSR--RPVKYGQCWVFAGVATSVLRCLGI-
PSRPVTNYCSAHDRD-ASMSYDVYL-DESLAPRDDLNKDSIWNYHVWSECWMARPDLPLG--
YGGWQIVDATPQEN--SEGI--FRCGPSPVNAVKSGIVYLPYDTKFVFAEVNSDKVYWK---VG-L--
DGE---LSPVDVERRAVGHCISTKAIGSDNREDITHAYKYPEGSDEERVSVEMA-CRYGTGSRPNH--
-LEVTAPLDGALTMGRDVALGVRLKNHSGEER-HVSLLLHCDALFYTGVVRT-AVK-RQR-
FDLDLPPGAEHQVVLQVRRGEYL-GMLV---DQG-GLMLTATGRV----LETSQ-
LVAQRAFHLTLAP-LRITVLG-EVRSGWDFMAEVSFTNPLPTVLQGVTFRLEATGLQ-QNK-VIRH-
GDIRVGET-VTVRERLTPTRPGL-RKLAGSMVCRQL-TQV-LGDTDLNV
>lamprey_TGM1L3
LEALWVDAGGRG---NRARHRSAEY--AAGH-VVTRRGGPVLLHAAFS---RT-PTDGV---
DRVSVELEIG--SHPQLNKGTHLIFPVAA-AASSSSWASGAGGANGR--NRALWLRVRA-
DCLVGKWRLFVSTQGPAG--
SHRSARREESDIYIIFNAWCPEDAVFMEVEAWRREYVLADTGRIYYGTEQQIGSRPWNYGQFEKDVLE
ASVMIMDRA-------SLPLAGRGCPVKVVRIVSAMVNSLD-DDGVLEGNWS--
GDYGGGTAPTAWNGSVDILQQFARSRS--PVRYGQCWVFSGVTTTVLRSVGI-
PTRSVTNFASAHDTD-TSLTTDVYL-DDDLQPIAELNTDSVWNFHVWNDCWMTRPDLPEG--
LGGWQAVDATPQET--SSGV--FCCGPASVAAVRNGLVYLTHDTPFVFAEVNSDRIFWL---RS-A--
DGT---VKRLDVERRAIGHCISTKAVGSDTREDITHLYKFPEDTEEERLAVETA-CLHGSPSSPD---
-VTLAISTHERVVMGHDFDVSVTVSSTAVSER-QVSVFVRGAVMHYTGVTRG-EAIKQLK-

LDVTLQPGEERVLTVTVRQGEYL-ERLV---DQS-AVMFLVSARV----NPGNA-
LTRQHCFRLCTPK-LLVTCAD-ECRVGEPLTVEVLFENPLPHLLRDVSFRLEGPGLQ-SPR-VIKH-
GNVSRGGV-VRLRETLLPERPGQ-RKLLVSLHCTQL-SQV-HGETDVLV
>chicken_TGM1
LVPRGLVVGSRA---DRIAHHTAEF--CSPQ-LVVRRGQPFHLRVLLP---RP-FDPED---
DSLCVELLLG--PTPQVAKGTHVLIPLGE--TSATGWTAEEAASGSP--ALRLRLSAPA-
DAPIGRYRLSVKTRTGAG--
EFGAPFDDRNDVIVLFNPWCEEDGVYMEQTNDLNEYVLNETGRIFYGTEEQIAERSWNYGQFDAGVLD
ACLAILDRR-------RMPHSARGDPVMVTRVVSAMVNSLD-DNGVLVGNWT--
GDYTQGTNPSAWAGSVDILRSYHRGGA--PVRYGQCWVFAGVVTTVLRCLGV-
PTRTVTNYNSAHDTD-VSLTTDIYF-DENMKPLERLNTDSVWNFHVWNDCWMRRPDLPAG--
YDGWQVVDATPQET--SSGL--FCCGPCSVTAVKNGEVFLKYDTAFVFAEVNSDKVYWQ---RK-G--
NGA---FAIVHVEEGAIGRRISTVGPQSAARIDITHLYKHPEGSEAERRAVSTA-TSHGSSRGEVR--
-LSLS---SGPAVAGAELELKVTAHNAAPQPR-TVRVRLSVCALRYTGVAAP-PFR-HEQ-
HRRVVAPGGEEQLCVAVPFSEYS-PHVG---SQD-ALRLTAAAAV----EETGE-
VAKELRVRLAAPD-LSMTLLG-PPVVGQEVSVQVLFRNPLPQKLTGAELRMEGAGLS-CPA-SISV-
GTVAPEQT-LRLRQPVVPLRAGR-RRLVAAMESAQL-GPV-HGELQFDA
>coelacanth_TGM1L3
LTVKSINLLKGK---NKKSHHTDEF--EYDE-LIVRRGQSFHIKLELS---RP-FNPEA---
DKLLLEMHLG--PKPQFGKGTLAIVRLVQ-ELDPKSWSMKIVKVSDR--VLTLKVNSSP-
EAPIGRYQLIVKSLTLNE--
EFRTKHNPDDDIYILFNPWCKADTVYMEDEERRKEYVLNDTGKLYRGMQRWIGASDWNFGQFDQGILE
ACFFLLDKG-------KMPHAGRGDPISMVRVVSAMINSAD-DDGVLVGNWS--
GDYSDGITPMAWGGSVEILLGYYSTGG-EPVSFGQCWVFSGLTTTVLRCFGI-
PTRSVTNFDSAHDTD-VSLSMDTYY-DEDMEEIRDLNSDSVWQ----------------P--
VATYLYVHTNHFLF--SKGT--YCCGPASLRAIKNGLVFLKYDAPFIFAEVNCDRIYWQ---RK-A--
DGT---FEKFDVVKNAVGHKISTKAVGSDEREDITHLYKYQEGSEEERISVQTA-CQYGTD-AAND--
-VTADIKAEDKIQIGSDVMLKISLGNRSMDKR-TVSLFVQSSAILYTGVNKG-TFK-REQ-
EEIVLGPCEVKEMVKMLKFEEYD-DHLV---EHG-SFMFTILGRV----KETKQ-
FVKQDHACLSSSS-LKIMILG-QAEVDREVQAKFEFTNSQTRRIRNVQLRIEGPGLQ-KPK-TIYI-
GDIRGNQT-MSHTEMIIPSKPGA-RKLIATLDSHQLITEV-QGFANVYV
>clawed_frog_TGM1
LQVQNVDLLKQR---TRRAHHTDEF--EYDE-LIVRRGQPFKICVTFQ---RA-FNPKS---
DRICVVLQMA--------NAQPITLSQVN-EFDESGWACQVAEVNGC--KSNIWLNTSA-
QAPIGLYQMIVKTNSGEG--------
PSNLRVYILFNAWCRMDSVFMDNEAWRQEYVLNEIGRIYYGTQNQIGERSWNYGQFDKGVLDACLYLL
DCG-------RIAPGSRGDPINVTRVISAMVNSMD-DNGVVAGSWS--
GDYADGVNPVAWVGSVDILLRYHQSGS--SVKYGQCWVFGGVTTTVLRCLGI-
PGRTITNFASAHDAD-GNLTFDIYF-DENMKPIEDKNRDSVWNYHVWNDCWMTRPDLPAG--
YSGWQAIDSTPQET--SNGI--YCCGPCPLLAIKNGLTNIKYDAPFIFAEVNSDKVCHQ---RM-P--
NGQ---FKRVLVEERAVGHCISTKAVGSFARDDITYLYKHPEGSTEERNSVHTA-ARYSNE-ETSD--
-VSMSVESQDGVITGSDINVRVLLKNNSNSRR-CVSLSLTVGVMYYNGICKE-SFK-NET-
RDTTLNPGEGKAVGMLISYAEYS-KHLV---DQG-AMMLTVSGIV----NETGQ-
LAKQHTFRTRTPD-LVIKVRG-DAIVGQQIVAEVIFKNPLNTTLHNAVFHVEGPGLQ-RPK-VIKY-
GNIGPLQT-VCVSERFTPRRPGP-RQFIASLESNEL-SQV-HGTTEVVV
>lizard_TGM1
LVLSGIDLMCSP---NRRSHHTNEY--EYEN-LIIRRGQPFDMKLQFR---QP-YDPDD---
HRICLEFLVG--PNPQVAKKTHILVPLGS-PLSDLSWSAELRDTGTN--TMTIRVNTSP-
EAVIGKYQFSVKTRSKAG--
EYQAPFDPRYEIYILFNPWCPDDPVYLDKTSSLDEYVLNESGRIYYGTETQIGERTWNYAQFDHGILD
ACLFMLDQR-------GMPHASRGDPIMVSRVVSAMVNSLD-DNGVLVGNWN--
GDYSRGTNPSAWVGSRDILLKYLKTGY--PVLYGQCWVFAGVVTTVLRCLGI-
ATRTVTNYNSAHDTD-VSLTMDIYF-DENMKPLEHLNADSVWNFHVWNDCWMKRPDLPSG--
FDGWQVVDATPQES--SSGI--FCCGPCSVEAIKNGLVYMKYDASFCFAEVNSDKVYWQ---RQ-S--
DGS---FKIVYVEEKAIGHLISTKSVGSNQRQDITAIYKHPEGSPEERKAVETA-AKHGTELGQDI--
-VTVD---TDEAYTGQDVSLRVTLKNRSSMPR-NVSLNLFVAVMYYTGVTGK-RFK-EER-
RQVQVPAGGNQDVPMVISYPEYK-KHLV---DQG-AMKLSISGKV----AETGQ-

IAKEHTFRLRTPD-LTLTLLG-PAIIGCETQVQIVFKNPLGVTLTNAIFHMEGSGLS-TPN-TMTV-
GNIGPHQT-VTLRQTFTPLRAGQ-RQLVASLDSPQL-SQV-HGVLTVNV
>human_TGM1
LVVNGVDLLSSR---NRREHHTDEY--EYDE-LIVRRGQPFHMLLLLS---RT-YESS----
DRITLELLIG--NNPEVGKGTHVIIPVGK--GGSGGWKAQVVKASGQ--NLNLRVHTSP-
NAIIGKFQFTVRTQSDAG--
EFQLPFDPRNEIYILFNPWCPEDIVYVDHEDWRQEYVLNESGRIYYGTEAQIGERTWNYGQFDHGVLD
ACLYILDRR-------GMPYGGRGDPVNVSRVISAMVNSLD-DNGVLIGNWS--
GDYSRGTNPSAWVGSVEILLSYLRTGY--SVPYGQCWVFAGVTTTVLRCLGL-
ATRTVTNFNSAHDTD-TSLTMDIYF-DENMKPLEHLNHDSVWNFHVWNDCWMKRPDLPSG--
FDGWQVVDATPQET--SSGI--FCCGPCSVESIKNGLVYMKYDTPFIFAEVNSDKVYWQ---RQ-D--
DGS---FKIVYVEEKAIGTLIVTKAISSNMREDITYLYKHPEGSDAERKAVETA-AAHGSGSAEDV--
-MQVE---AQDAVMGQDLMVSVMLINHSSSRR-TVKLHLYLSVTFYTGVSGT-IFK-ETK-
KEVELAPGASDRVTMPVAYKEYR-PHLV---DQG-AMLLNVSGHV----KESGQ-
LAKQHTFRLRTPD-LSLTLLG-AAVVGQECEVQIVFKNPLPVTLTNVVFRLEGSGLQ-RPK-ILNV-
GDIGGNET-VTLRQSFVPVRPGP-RQLIASLDSPQL-SQV-HGVIQVDV
>platypus_TGM1
LVVTGVDLLSGR---NRRDHHTDEF--EYDE-LVLRRGQPFHLSLSLA---RP-YQSS----
DRITLELLIG--STPEVGKGTHIIIPVGR--GGGGGWSAQVTKASGQ--HVNLRVNTSP-
SAIVGKFRFTVRTQSDAG--
EFQLPFDPRNEIYILFNPWCSDDPVFVNREDWRQEYVLNEVGRIYYGTEAQIGERTWNYGQFDHGVLD
ACLYILDRR-------GMPYAGRGDPITVSRVVSAMVNSLD-DNGVLIGNWS--
GDYSRGTNPSAWVGSVEILLSYLRTGY--SVPYGQCWVFAGVTTTVLRCLGI-
ATRTVTNYNSAHDTD-TSLTMDIYF-DENMKPLEHLNRDSVWNFHVWNDCWMRRPDLPSG--
FDGWQVVDATPQET--SSGI--FCCGPCSVESIKNGLVYMKYDTPFIFAEVNSDKVYWQ---RQ-S--
DGS---FKIVYVEEKAIGTLISTKAIGSNMREDITHIYKHPEGSDAERKAVETA-TAHGSDAAEDV--
-VQVE---AEDAVMGQDLAVSVVLTNRGGSQR-TVKLHLYLSVTFYTGVTGP-VFK-DSK-
KEVALAPGASDRVVLPVAYSEYR-PHLV---DQG-AMLLNVSGRV----LENGQ-
LAKQHTFRLRTPD-LSLTVLG-AAVVGQETEVQIVFKNPLPITLTNVVFRLEGSGLQ-RSK-VLNV-
GDIGGNET-VTLRQTFVPVRPGP-RQLVASLDSPQL-SQV-HGVIQVDV
>zebrafish_TGM1L4
LAVRSVDLLRLR---NRRQHHTDGF--SSER-LIVRRGQSFQITVELS---RA-FKPRA---
DSLQLQLKLD--SAVSNSSGLLISVPLVE-DLEDRRWEMKIVEQKEN--RVRLVVNTLP-
SASIGCYKLTVVSFSPRG---
KLLFPCTPDDVYLLFNPWCEDDPVYLDNEAERKEYVLNTMGRIYYGTEQQIGTRTWNFAQFEQNILEA
CLFLLERG-------RVAVTEWRDPVIISRMVSALVNSND-DRGVLMGNWS--
ESFEGGTAPTAWSGSGDILRQYYSSKG-SPVRFAQCWVYAGVTCTVLRCLGI-
PTRCVTNFSSAHDTD-LSLTTDIYL-DEKLEMLKDKTSDSIWNFHVWNESWMRRGDLPAG--
YGGWQVVDATPQEQ--SQGS--YRCGPTPVSAVRSGQVNLRFDTPFVFAEVNSDKIYWQ---RN-A--
DGS---FRQVSVEKNSIGQKTSTKAVGSDTRVDITHLYKYPEGSEEERIAVESA-SRFGS--SGTD--
-VLEVQMSGAGPRIGEDVQLSIVLKNSSSAQR-SASLLYEALVMYYTGVLKQ-SLK-KDR-
ITLELQPRETKTIPWTLQYKEYK-EQLV---DQG-ALMLTLTGRV----SQTKQ-
LATQFNFRLRTPD-LVLTPLQ-DAVVGKEMSVRISFQNPLSQVLKNVLFRIEGLGMQ-SVR-KISY-
GDVARLGT-VSLIEKFTPTVSGS-QKLLASMDCRQL-TQV-HGVADITV
>zebrafish_TGM1L5
LQVRSVDLIKTR---NRQEHHTDAF--FSNH-LIIRRGQCFQMTIELS---RP-LIPNK---
DQLYLELRLG--NVVPAHRDSFVSVPIVS-EFKKNAWEAKIIEQAKT--TIKLSVYSLP-
TACIGQYKLTVVTNCPAG--
KATSPYTPDNDIYMLFNPWCKDDPVYLKDEAERNEYVLNDMGKMYYGTEQQIGTRTWNFGQFDEGVLE
ACFFVLEKS-------GSPCSGWGDPINVVRVISALVNSND-DQGVLIGNWQ--
NSYEGGLSPTAWSGSSAILKQYHKSGG-TPVKFGQCWVFAGVTNTMLRCFGV-
PTRPVSNFSSAHDTD-VSLTTDVYL-DEKLEEIKDLNRDSIWNFHVWNESWMTRPDLPAG--
FGGWQVVDATPQET--SQGV--FRCGPTSVAAVRSGQVYLKYDTPFVFAEVNSDKVFWQ---RQ-S--
NGS---FTVIKVDENAVGHCISTKAVGSDQRVDITHLYKHPEGSSEERSAVEAA-CSFGS--
SSSNTDVTVDVVMEDSGACLGQDAVLFIVLKNRSSSAR-TVDLQSRVEAVDYTEHHKA-FLR-KDQ-
TRAQLKPHEIQSLEWILQYEEYK-EELE---GQT-SLLLSLSGRI----TETKQ-

```
LVKHFTFRLRTPD-LVLTPVG-DAVVGQELKVKLKFQNPLVSVLRNVIFRMEGLGLQ-HVK-TIHY-
GDITGGAT-VRLTEIFVPKRSGP-QKLLATLDCPQL-TQV-HGVANILV
>zebrafish_TGM1L3
LQVRCVDLMKCW---NRQEHHTNGF--RGDH-LIVRRGQCFQMWVELS---RP-FNPKC---
DQLHLELKLG--NVPSIPNGTLVIVPLVE-EFKKNRWEAKIVEKCQN--RIKLSVYSLP-
TACIGRYSLTIVTCGPKG--
RATSSCNPSNDIYMLFNPWCKDDAVYLDEDAQRTEYVLNDTGKIFYGTKHQIASRTWHFGQFDEGVLA
ACLFVLEKC-------GGACSGWGDPVNVARVVSAMVNAND-DSGVLMGNWS--
NCYADGTAPTAWCGSSAILKQYHKCGG-VPVKYGQSLAFAGVTNTLLRCFGI-
PARPVTNFCSAHDTD-VSMTVDIYL-DENYDLIDSLNRDSIWTFHVWNEAWMARSDLPAG--
FGGWQAIDATPQET--SQGV--FRCGPTSVAAIRSGQVFLKYDAPFIFAEVNSDVVFWQ---RK-A--
CGT---FAVVHVDKNAVGHCISTKAVGSDKRVDITNHYKHPEGSEEERRAVETA-LRHGS--CAED--
-VCDITMKGDGVCVGKDAVLCIALKNKCSSTR-SVTLHSQLSAAYYTGIHKS-LVK-KDQ-
TCFELKATETKVLEWSLKYEDYK-NHLV---DHS-TMMVTVAGRV----TQTQQ-
VAKRFNFRLPTPG-LAISPGC-DCVVGKEVPVKITFQNPLPCVLKNAIFRIEGLGLK-HCR-SINY-
GDIAGLAT-VNLTEKFIPKCHGP-HKLLASLDCPQL-TQV-HGFTDVVV
>zebrafish_TGM1L2
LRVSKVDLLSCR---NRMEHRTHFF--HEEK-LIVRRGQCFNMWIDLC---RP-FNPTC---
DKLHLELRLG--HIPSIRDGTYVIVPIVD-EFKKDCWGARIVERCQN--RLKLCVNSLP-
TSCVGRYQLSVVTHCSAG--
RFCLPYVPENDIYMLFNPWCKEDCVYLHEETERAEYVLNDIGKIYYGTKHQIGCKSWNFGQFEEGILP
ACFYVLEKS-------CAPCSGWGNPINISRVVSDMVIAKK-DCGVLMGNWS--
NCYTDGIAPTSWCGSSAILRQYYKCGG-APVRYGQSLAFAGVTNTMLRCLGI-
PTRPVSNFCSAHDTD-MCLTSDVYL-DEKFQLIDHMNANPIWNYHVWNEAWMTRPDLPTG--
FGGWQAIDSTPQLT--HQGF--FRCGPTSVAAIRSGQTFLKHDVPFLFAEVNNDKVYWQ---RK-C--
DGT---FGVVHVEKDVVGHCISTKAVGSDQRLDITNLYKHPCGSEDRCTALETA-LRHGC--CAED--
-VCEVNLKGDGPCVGRDAVVCINLRNKCNQPR-SVTLYSHAAAMYYTGVRRT-YLK-RDQ-
TCIELKPSECKPLEWTLSYDEYK-EHLV---DHA-PLMLNLFGHV----AQTKQ-
LATQYNFRLRTPD-LVLAPAC-DAVVGQEVAVKVTFQNPLSCVLRNAAFRFVGLGLQ-HPR-IINY-
GDIAGHAT-VSLTEKFIPMCHGP-QKLLASFDCPQL-TQV-HGFTNMVV
>zebrafish_TGM1L6
LRVIKIDLLKCK---NRQEHHTHFF--HDEQ-LIVRRGQSFNMWVDLC---RP-FNPAS---
DKLHLELRLG--HIPSIRDGTYVIVPIVE-EFKKDCWGAKIVEHGQN--RIKLCVNSLS-
TACVGRYQLSVVTQCTAG--
KFTLPYVPEHDIYMLFNPWCKEDSVYLSDQAERTEYVLNDMGKIYYGTKQQIGCKTWNFGQFEDGILP
ACFYVLEKS-------GTPCSGWGNPINISRVVSEMINANK-DRGVLIANWS--
NYYVDGTAPTLWSSSSAILKQYHKCGG-VPVKYGQSLAFAGVTNTMLRCLGI-
PARPITNFCSAHDTD-VSLTTDVYL-DEKFQLIDQMNRDSIWNYHVWNEAWMTRPDLPTG--
FGGWQAIDSTPQQT--HQGF--FRCGPTSVAAIRSGQTFLKHDVPFLFAEVNSDKVYWQ---RK-N--
NGT---FGVVHVEKDVVGHCISTKAVGSDQRVDITNLYKHPCGSEDRLTALETA-LRHGC--IPED--
-VCEVNLKGDGPLVGKDAVLSINLKNKCNQPR-SITLYSHAAAMYYTGVRRT-YLK-RDQ-
TSIELKASESKLLEWTLSYDEYK-EHLV---DHA-PLMLNLFGHV----AQTKQ-
LATQYNFRLRTPD-LVLAPVC-DAVVGQEVAVKVTFQNPLSCVLKNSVFRFVGLGLK-QAR-VINY-
GDIAGHAT-VSLTEKFIPMCHGP-QKLLASFDCPQL-TQV-HGFTNMVV
>coelacanth_TGM1L2
LKVKNIDLLKKP---NKKSHHTDEY--EYDE-LIIRRGQPFQVKLEFS---RP-FNPET---
DKLFLELKLG--SQPQVGKGTHVIVKLVE-KHIPSEWGAKIIEASGN--TLTLSINSSP-
QTMIGRFELTVKTVTEGG--
AFKMKYNRDNDIYFLFNPWCEADTVYMEDEEWRKEYVLNDTGRLYYGTENSIGSRTWCFAQFEKGILE
ACFYLLDKG-------GMPDSGRGDPISVVRVISAMVNSQD-DDGVLVGSWS--
GVYWDGRRPTSWTGSSEILLHYHDS-Y-NPVRYGQCWVFSGVATTVLRCLGI-
PGRSVTNFSSAHDSD-TSLEIDYYI-DEDHCALDHLNNDSIWNFHVWNECWMARPDLPQG--
YGGWQVVDATPQET--SAGT--YCCGPASVRAIKNGLVYLKYDAPFIFAEVNSDKVYWI---RD-S--
NGD---FKRYYSARSVVGHNISTKAVGSEERLDITNLYKYPEGSEEERLSVKTA-CKFGNE-VEED--
-VVVEVEITGNDLIGSNIIARATVTNRGNNVC-NICLFMRAAMTFYTGVYKN-EIK-NMK-
EEVTLEPAEVKEVKMTIKYDEYD-DYLE---DQA-CIMFTLMGLV----SETKQ-
```

ITKQRDYRLRTPD-LMLKVHG-EVVKGKESKVVISFVNPLPKVLKNVVIHLEGPGLQ-KRK-TISV-
GEVAKQGL-MTITEIFIPSKAGC-RKLIANLDCHQL-TQV-HGVVEVEV
>zebrafish_TGM1
LEVRSVNLLKSS---NRLEHHTERY--RSEN-LIIRRGQTFQMQIELS---RA-FDPKT---
DKLHLDLKLG--DLPDVSKGTHVVVPLVE-ELQDNCWEAKIVEQKGR--LIKLSVNSLP-
TAPIGKYKLGVATSCPVG--
ESMSPYNPDNDIYMLFNPWCEEDSVYMDSEKERKEYVLNDMGIIYYGTDSQIGYRTWNFGQFDKSILP
ACLFLLERS-------GAPASGWGDPVNVVRLLSAMINAPD-DNGVLVGNWS--
GTYDDGTAPTFWSGSSDILRQYYNNGG-TPVRYGQCWVFSGVTTSVMRCLGI-
PTRSITNFESAHDTD-ASLTTDVYL-DENHELIEELCSDSVWNFHVWNDCWMARPDLPAG--
LGGWQVVDATPQET--SQGA--FRCGPASVAAVRNGLVYLKHDTPFVFAEVNSDKVYWQ---RT-A--
DGS---FTPVQIKKKAVGHCISTKAVGSDEREDVTHLYKYPEDSEEERIAVETA-VQHGS--SVND--
-VIEISMDGEDPQLGSDANLAITVKNSSSEMR-TFQLSAQVAVTYYTGVYKG-TVR-KDQ-
ISIELKPNEAETVEWTLLYDHYK-DHLV---DQA-MLMLILTGRV----NETKQ-
LVNQFHFRLRTPD-LAIKTEG-DAVVGKELKASITFRNPLKQTLKNVKFRIEGLGLQ-HVR-EISH-
GNIESLAT-VTLTETFIPLEAGQ-HKLLAALDSRQL-PQV-HGVANITV
>zebrafish_TGM1L1
LVVRSVDLLSRK---NKKEHHTDQY--SGDQ-LIIRRGQTFQIELELS---RP-FNPNT---
DKLHLELKTG--ALPLVSKGTHIIIPLVE-ELQDERWEAKIVEQNLN--RAKLSINSSV-
NAVIGKYTLTVVTQCLKT--
NESTTHDPEKDIYMLFNPWCEDDAVFMEGERELNEYVLNDTGRIYYGTEKQIGARTWNFGQFDEGILE
ACLFVLDNS-------EVPPSGRGDPVNVVRVISAMINSPD-DRGVLEGNWS--
GNYTGGTSPTAWSGSVEILKQYHREGG-TPVKYGQCWVFSGVTTTVLRCLGI-
PARSVTNFQSAHDTD-VSLTTDVYF-DEDMEPIHHLNSDSVWNFHVWNDCWLARPDLPPG--
FGGWQAVDSTPQET--SHGT--FRCGPASLAAVRSGQVYLKYDVPFVFAEVNSDKIYWQ---RN-L--
DGT---FSQIHSEKKAVGHCISTKAVGSDERVDITDVYKYPEGSEEERIAVETA-CRYGS--MAED--
-VVEVRMEGEGPRMGGDAQLKIVVKNMSSQPR-RTTLHSQAAVMYYTGVLKD-TVK-KDK-
LSVELMPQEEKVIEWTLPYTQYQ-NQLV---DQA-ALMLTLSGRV----SETQQ-
LANQTSFRLRTPD-LQIEPIG-EAYVGKEASAKISFTNPLPCTLRNVVVRVEGLGLRDLHP--IKV-
GDVGKHGK-VMVTEHFIPSIAGE-RKLVASLDCKQL-TQV-HGVADIIV
>coelacanth_TGM1L4
LEVRNINLLKKIHENNKIFHHTDEF--ESDY-LIVRRGQPFEIKVELS---RP-FDAQT---
DMLLLELQIG--PKPRLKKGTLVIVQVVK-EHNPKEWGMKTFWETEQ--TLTLSVNSSP-
EAVIGRYQLTVRTLTRKE--
EFRTKHNPDNDIYILFNPWCEADIVYMENEEWRKEYVLNEVGTLYYGTSEAIKSKMWNFGQFEKGILE
ACFNLLEKG-------RLPQYVWGNPITLVRFISAMVNSQD-DDGVVIGNWT--
GDYSEGTAPTAWAGSVDILHQYHQTG--KPVKFGQCWVFSAITTTVLRCLGI-
PTRSLTNFDSAHDTD-VSLTMDIYI-DEALEPIEEMNLDSTWNFHVWNECWMARPDLPPG--
YGGWQAVDATPQEM--SVGK--FCCGPASVRAIKNGLVYLNWDSPFIFAEVNCDIVYWK---KK-K--
DGS-GIFRPIFKEESAIGHFISTKAVGSNKREDITHLYKYPEGSEEERISVETA-CQHGTKPKTKD--
-VVINVQTEKNLQMGSDFTVWLRVQNCSTEQR-GISLFTQVAAIYYTGVYKS-CFK-KKR-
EEVQLSASEVRELELVIKYSEYD-PHLE---SQD-NMLFTVLGRV----
AETRQVIAKQHKFCLCTPD-LQIRVLG-EAIVGKEMKAEIVFINPLPKILKNVQLHIEGPGLQ-TPK-
IVTI-GDVGSHAK-VTHTESLVPVRPGM-RTLIANLDCPQL-SQV-HGVAEILV
>coelacanth_TGM1L1
LEVRNINLLKKI---NKIFHHTDEF--ESDY-LIVRRGQPFEIKVELS---RP-FDAQT---
DMLLLELQIG--PKPRLKKGTLVIVQVVK-EHNPKEWGMKTFWETEQ--TLTLSVNSSP-
EAVIGRYQLTVRTLTRKE--
EFRTKHNPDNDIYILFNPWCEADIVYMENEEWRKEYVLNEVGTLYYGTSEAIKSKMWNFGQFEKGILE
ACFNLLEKG-------RLPQYVWGNPITLVRFISAMVNSQD-DDGVVIGNWT--
GDYSEGTAPTAWAGSVDILHQYHQTG--KPVKFGQCWVFSAITTTVLRCLGI-
PTRSLTNFDSAHDTD-VSLTMDIYI-DEALEPIEEMNLDSTWNFHVWNECWMARPDLPPG--
YGGWQAVDATPQEM--SVGK--FCCGPASVRAIKNGLVYLNWDSPFIFAEVNCDIVYWK---KK-K--
DGS-GIFRPIFKEESAIGHFISTKAVGSNKREDITHLYKYPEGSEEERISVETA-CQHGTE-KVKD--
-VVINVQTEKNLQMGSDFTVWLRVQNCSTEQR-GISLFTQVAAIYYTGVYKS-CFK-KKR-
EEVQLSASEVRELELVIKYSEYD-PHLE---SQD-NMLFTVLGRV----AETRQ-

```
IAKQHKFCLCTPD-LQIRVLG-EAIVGKEMKAEIVFINPLPKILKNVQLHIEGPGLQ-TPK-IVTI-
GDVGSHAK-VTHTESLVPVRPGM-RTLIANLDCPQL-SQV-HGVAEILV
>shark_TGM1
LEVTNLDLLKGSQEVNQQRHHTAEF--ECTE-LVVRRGQPFEIRLRFN---RP-YDPQS---
DTVRLELRTG--DNPQPAKGTHVTLAPVE-QPERGQWGAEVLDRTGGGRGLSLSVHSPP-
GCPVGRYRLSARTQDPAG-----
PGKWLEEAVYILFNPWCPDDVVFVDEEKMRNEYVLNETGRIYYGTEKQIGARTWNFGQFSKGILEACL
FMLDRS-------KMPVAGRGDAVSIVRVISAMINSQD-DCGVLVGNWS--
GQYTGGTAPTAWIGSGDILLQYHRTG--EPVQYGQCWVFSGVVTTVLRCLGI-
AARSVTNFASAHDTD-VSLTTDVFL-DEEMKPLDHMNYDSIWNFHVWNDCWMARPDLPAG--
FGGWQAIDATPQET--SSGQ--YCCGPASVEAIRNGMVYLKYDAPFIFAEVNSDKVFWQ---RQ-T--
DGT---FQKVLVLKKAVGHQMSTKAVGSDERDDITESYKYTEGTDEERIAVETA-CRHGSRYTASD--
-VEVTVHTDDGIIMGSDFTVTVGVANTGSECR-SLTLFVQAIVMYYTGVAKG-SLK-KDK-
RDVLLEPHEKKEVKLVFHNEDYL-EFLV---DQA-AMMFTVTGRV----
KETGQAIVNQHTFRLRTPD-LMITPLG-DAQVGKPMKVEIALTNPLPKSLKNVTLRIEGPGLQ-NPG-
KVNI-GDVPRHAS-ITVTETLVPLKPGC-RKLIASLDCQQL-TQV-HGVAEVLV
>lungfish_TGM1
LIVKKIDLMKKK---NKITHHTNEY--DYDE-LIVRRGQPFDMKLQFN---RP-YNPET---
DQIFLELQTG-KHYPQVTKGTLIIVPLVE-EHNRMEWGAKVTQASGN--HVTLSVNSSP-
EAVIGKFQLSVRTRGPGG--
ELKTPSAPENSIYILFNPWCEKDSVFLDNEEWRKEYVLNETGMIYYGTEFQIGARTWNFAQFSKGVLD
ACLYVLEKG-------GMPASGSKDSVSTARVISAMVNSPD-DDGVLEGNWS--
GDYSDGTSPTSWVGSEDILLQYHKTR--KPVCYGQCWVFSGVTTTVLRCLGI-
PSRSVTNFSSAHDTD-VSLTMDIFF-DENMKSLEDMNYDSVWNFHVWNDCWMARPDLPPG--
YGGWQAVDATPQET--SSGT--YCCGPASILAIRNGLVFLKHDTPFIFAEVNSDKIYWQ---RQ-A--
DGT---FKKLFIEKKAIGHKISTKAVASNEREDITSLYKHPEGSEEERIAVETA-SKYGND-MPSD--
-VGIEVQTDEKTTMGSDVVLCVGLTNKSNEQR-FVTLFINTAMMYYTGVLKE-SFR-KEE-
KAVQLSPYEEARVEMVIKYAEYH-EELI---DQA-AMMFTISGRV----QETGQ-
LATQQTFRLRTPD-LEIIPIG-GAVVGQEMRAKISFVNPLPKTLTGVYFRVEGPGLH-KPN-IVQV-
GDVARGAT-VTITQTFVPVKPGP-RKLIASLDSRQL-TQV-HGVAEVDV
>coelacanth_TGM1
LTVKNVNLVRGR---NKKSHHTDEF--EYDE-LIVRRGQPFQIKLELS---RP-FNLET---
DKLFLELQTG--LLPQVSKGTLVIIPVVE-ELEYNEWGVKIVEATDN--VLSLLVNSSP-
QAVIGRFELTVKLQPAGE-
RENRTEHKPANDIYILFNPWCEADAVFVEDEEWRKEYVLNETGRIYYGTKNQIGARTWNFGQFDKGVL
DACLYLLEKG-------KMPHSGRGDPISMVRVVSAMVNSQD-DRGVLVGNWS--
GDYTGGSAPTEWVGSVDILLQFHRTG--EPVNYGQCWVFSGVTTTVLRCLGI-
PGRSVTNFASAHDTD-VSLTTDVYL-DENLEPLEHMNFDSVWNFHVWNDCWMARLDLPPG--
YGGWQAIDATPQET--SAGT--YCCGPASLQAIKSGLVYLKCDAPFIFAEVNSDRIYWQ---RQ-H--
NGT---FQKVLVQKNAVGHQISTKAVGLDEREDITHLYKYPEDSEEERIAVETA-CQYGSA-VMED--
-VALDIQTQEDIQMGSDVTVRVLVENCSSEHR-SISLFLKAAVVYYTGVYKN-SFK-QDR-
EEVLLSPAEGKELLVVLSYSQYQ-EYLV---DQA-AMMLTVSGRV----VETGQ-
LAKQHNFRLRTPD-LQIMPYG-EAIVGGQMKAEIVFLNPLPKILKNVTFRIEGPGLQ-KPK-KVQV-
GDVGRHAT-VTLKETFVPTKPGP-RKLIASLHCREL-TQV-HGVAEVIV
```

Figure S10. Multiple sequence alignment of vertebrate TGMs used for the phylogenetic analysis. The trimmed aligment contains the Transglut_N domain, Transglut_core domain and the two Transglut_C domains. Sequences are provided in the fast_aln format. Species: Human (*Homo sapiens*), platypus (*Ornithorhynchus anatinus*), chicken (*Gallus gallus*), lizard (*Anolis carolinensis*), clawed frog (*Xenopus tropicalis*), lungfish (*Protopterus annectens*), coelacanth (*Latimeria chalumnae*), zebrafish (*Danio rerio*), shark (*Carcharodon carcharias*), lamprey (*Petromyzon marinus*), tunicate (*Ciona intestinalis*), lancelet (*Branchiostoma floridae*), fruit fly (*Drosophila melanogaster*).

Supplementary Table S1. Accession numbers of transglutaminases investigated in this study

Species	short	Gene	NCBI GenBank accession number	Notes
Homo sapiens	human	TGM1	NP_000350	
Homo sapiens	human	TGM2	NP_004604	
Homo sapiens	human	TGM3	NP_003236	
Homo sapiens	human	TGM4	NP_003232	
Homo sapiens	human	TGM5	NP_963925	
Homo sapiens	human	TGM6	NP_945345	
Homo sapiens	human	TGM7	NP_443187	
Homo sapiens	human	F13A1	NP_000120	
Homo sapiens	human	EPB42	NP_000110	
Ornithorhynchus anatinus	platypus	TGM1	XP_028933142	
Ornithorhynchus anatinus	platypus	TGM2	XP_028904586	
Ornithorhynchus anatinus	platypus	TGM3	XP_028904555	
Ornithorhynchus anatinus	platypus	TGM4	XP_039768747	
Ornithorhynchus anatinus	platypus	TGM5	XP_028934239	
Ornithorhynchus anatinus	platypus	TGM6	XP_039766001	
Ornithorhynchus anatinus	platypus	TGM7	XP_028934236	
Ornithorhynchus anatinus	platypus	TGM9	XP_028921818	corrected*
Ornithorhynchus anatinus	platypus	F13A1	XP_028909882	
Ornithorhynchus anatinus	platypus	EPB42	XP_039769781	
Gallus gallus	chicken	TGM1	BK059845	see Figure S1*
Gallus gallus	chicken	TGM2	NP_001378925	
Gallus gallus	chicken	TGM3	XP_040544622	
Gallus gallus	chicken	TGM4	NP_001006368	
Gallus gallus	chicken	TGM6	XP_040544619	
Gallus gallus	chicken	TGM6L	XP_015152172	
Gallus gallus	chicken	TGM9	XP_015137927	
Gallus gallus	chicken	F13A1	NP_990016	
Gallus gallus	chicken	EPB42	NP_001308488	
Anolis carolinensis	lizard	TGM1	XP_016852481	first half of sequence*
Anolis carolinensis	lizard	TGM1L	XP_016852469	second half of sequence*
Anolis carolinensis	lizard	TGM2	XP_008119776	
Anolis carolinensis	lizard	TGM2L	XP_016853249	
Anolis carolinensis	lizard	TGM4	XP_008113745	
Anolis carolinensis	lizard	TGM4L1	XP_008113741	
Anolis carolinensis	lizard	TGM4L2	XP_008113740	
Anolis carolinensis	lizard	TGM3L1	XP_016854018	
Anolis carolinensis	lizard	TGM3L2	XP_003230298	
Anolis carolinensis	lizard	TGM9	XP_003219770	corrected*
Anolis carolinensis	lizard	F13A1	XP_016852585	
Anolis carolinensis	lizard	EPB42	XP_008121298	
Xenopus tropicalis	clawed frog	TGM1	XP_012826927	
Xenopus tropicalis	clawed frog	TGM2	NP_001123852	
Xenopus tropicalis	clawed frog	TGM3L1	XP_031750711	
Xenopus tropicalis	clawed frog	TGM3L2	XP_004918837	
Xenopus tropicalis	clawed frog	TGM3L4	XP_031750708	
Xenopus tropicalis	clawed frog	TGM3L5	NP_001361172	
Xenopus tropicalis	clawed frog	TGM3L7	XP_031750709	
Xenopus tropicalis	clawed frog	TGM3L8	XP_002935578	
Xenopus tropicalis	clawed frog	TGM3L9	XP_031750472	
Xenopus tropicalis	clawed frog	TGM4	XP_002937794	
Xenopus tropicalis	clawed frog	TGM4L	XP_002937793	
Xenopus tropicalis	clawed frog	TGM5	XP_031750630	

Xenopus tropicalis	clawed frog	TGM3L	NP_001120399	
Xenopus tropicalis	clawed frog	F13A1	XP_031759894	
Xenopus tropicalis	clawed frog	EPB42	XP_002935587	
Protopterus annectens	lungfish	TGM1	XP_043914271	
Protopterus annectens	lungfish	TGM1L	XP_043914432	
Protopterus annectens	lungfish	TGM2	XP_043946131	
Protopterus annectens	lungfish	TGM10	XP_043935473	
Protopterus annectens	lungfish	TGM5L	XP_043946061	
Protopterus annectens	lungfish	F13A1	XP_043920796	
Latimeria chalumnae	coelacanth	TGM1	XP_014350256	corrected with SRR391920*
Latimeria chalumnae	coelacanth	TGM1L1	XP_006014051	
Latimeria chalumnae	coelacanth	TGM1L2	XP_006008136	
Latimeria chalumnae	coelacanth	TGM1L3	XP_006006388	
Latimeria chalumnae	coelacanth	TGM1L4	XP_006008135	corrected with SRR391920*
Latimeria chalumnae	coelacanth	TGM10	XP_005997256	
Latimeria chalumnae	coelacanth	TGM2	XP_006007736	
Latimeria chalumnae	coelacanth	TGM4	XP_005999060	
Latimeria chalumnae	coelacanth	F13A1	XP_014352948	
Danio rerio	zebrafish	TGM1	XP_009294851	
Danio rerio	zebrafish	TGM1L1	XP_694950	
Danio rerio	zebrafish	TGM1L2	XP_009295369	
Danio rerio	zebrafish	TGM1L3	XP_001331914	
Danio rerio	zebrafish	TGM1L4	NP_001025267	
Danio rerio	zebrafish	TGM1L5	XP_009295379	
Danio rerio	zebrafish	TGM1L6	XP_021325715	
Danio rerio	zebrafish	TGM2a	NP_001004647	
Danio rerio	zebrafish	TGM2b	NP_997821	
Danio rerio	zebrafish	TGM5L	NP_001313420	
Danio rerio	zebrafish	TGM8	XP_009301053	
Danio rerio	zebrafish	TGM10	XP_687398	
Danio rerio	zebrafish	F13A	XP_686649.5	
Danio rerio	zebrafish	F13A1a.1	NP_001070179	
Danio rerio	zebrafish	F13A1b	XP_021333236	
Carcharodon carcharias	shark	TGM1	XP_041031332	
Carcharodon carcharias	shark	TGM2L	XP_041060189	
Carcharodon carcharias	shark	TGM2L2	XP_041060188	
Carcharodon carcharias	shark	TGM2L3	XP_041060568	
Carcharodon carcharias	shark	TGM2L4	XP_041060955	
Carcharodon carcharias	shark	TGM10	XP_041047102	
Carcharodon carcharias	shark	F13A1	XP_041040322	
Petromyzon marinus	lamprey	TGM1L1	XP_032816217	
Petromyzon marinus	lamprey	TGM1L2	XP_032827092	
Petromyzon marinus	lamprey	TGM1L3	XP_032834804	
Petromyzon marinus	lamprey	TGM1L4	XP_032816216	
Petromyzon marinus	lamprey	F13A1L	XP_032833350	
Petromyzon marinus	lamprey	TGM10	XP_032816667	
Petromyzon marinus	lamprey	TGM10L	XP_032833357	
Branchiostoma floridae	lancelet	TGM1L1	XP_035678619	
Branchiostoma floridae	lancelet	TGM1L2	XP_035678971	
Branchiostoma floridae	lancelet	TGM1L6	XP_035691780	
Branchiostoma floridae	lancelet	F13A1L	XP_035674033	
Ciona intestinalis	vase tunicate	TGML1	XP_002119887	
Ciona intestinalis	vase tunicate	TGML2	XP_002128434	
Ciona intestinalis	vase tunicate	TGML3	XP_009861590	
Drosophila melanogaster	fruit fly	Tg	NP_609174	

* The amino acid sequence is shown in Figure S1.

Table S2. GenBank accession number of genes located close to TGM1 (locus shown in Figure S3)

Species	name	Gene	chromosome/scaffold	NCBI GenBank accession	notes
Homo sapiens	human	*FEN1*	chromosome 11	NP_004102	
Homo sapiens	human	*CIDEB*	chromosome 14	NP_001380268	
Homo sapiens	human	*RABGGTA*	chromosome 14	NP_878256	
Homo sapiens	human	*TGM1*	chromosome 14	NP_000350	
Homo sapiens	human	*TINF2*	chromosome 14	NP_001350597	
Homo sapiens	human	*GMPR2*	chromosome 14	NP_001269951	
Homo sapiens	human	*NEDD8*	chromosome 14	NP_001186752	
Homo sapiens	human	*MDP1*	chromosome 14	NP_001186750	
Gallus gallus	chicken	*FEN1*	chromosome 5	NP_001072959	
Gallus gallus	chicken	*TGM1*	chromosome 35	BK059845	see Figure S1*
Gallus gallus	chicken	*NEDD8*	chromosome 35	XP_046761005	
Anolis carolinensis	lizard	*FEN1*	scaffold NW_003338782.1	XP_016851055	
Anolis carolinensis	lizard	*CIDEB*	scaffold NW_003338947.1	XP_003226697	
Anolis carolinensis	lizard	*RABGGTA*	scaffold NW_003338947.1	XP_016852482	
Anolis carolinensis	lizard	*RABGGTAL*	scaffold NW_003338947.1	XP_016852470	
Anolis carolinensis	lizard	*TGM1*	scaffold NW_003338947.1	XP_016852481	first part of TGM1 sequence*
Anolis carolinensis	lizard	*TGM1*	scaffold NW_003338947.1	XP_016852469	second part of TGM1 sequence*
Anolis carolinensis	lizard	*TINF2*	scaffold NW_003338947.1	XP_008117769	
Anolis carolinensis	lizard	*GMPR2*	scaffold NW_003338947.1	XP_003226714	
Anolis carolinensis	lizard	*NEDD8*	scaffold NW_003338947.1	XP_008117761	
Anolis carolinensis	lizard	*MDP1*	scaffold NW_003338947.1	XP_008117762	
Xenopus tropicalis	clawed frog	*FEN1*	chromosome 4	NP_001017005	
Xenopus tropicalis	clawed frog	*CIDEB*	chromosome 2	NP_001011434	
Xenopus tropicalis	clawed frog	*RABGGTA*	chromosome 1	NP_001006728	
Xenopus tropicalis	clawed frog	*TGM1*	chromosome 1	XP_012826927	
Xenopus tropicalis	clawed frog	*TINF2*	chromosome 4	NP_001137397	
Xenopus tropicalis	clawed frog	*GMPR2*	chromosome 1	NP_001025529	
Xenopus tropicalis	clawed frog	*NEDD8*	chromosome 1	NP_001016973	
Protopterus annectens	lungfish	*FEN1*	chromosome 16.part0	XP_043914536	
Protopterus annectens	lungfish	*TGM1L*	chromosome 16.part0	XP_043914432	
Protopterus annectens	lungfish	*CIDEB*	chromosome 16.part0	XP_043913793	
Protopterus annectens	lungfish	*RABGGTA*	chromosome 16.part0	XP_043913984	
Protopterus annectens	lungfish	*TGM1*	chromosome 16.part0	XP_043914271	
Protopterus annectens	lungfish	*TINF2*	chromosome 16.part0	XP_043914337	
Protopterus annectens	lungfish	*GMPR2*	chromosome 16.part0	XP_043914045	
Protopterus annectens	lungfish	*NEDD8*	chromosome 6.part0	XP_043930133	
Latimeria chalumnae	coelacanth	*FEN1*	scaffold NW_005824665.1	XP_006014031	
Latimeria chalumnae	coelacanth	*CIDEB*	scaffold NW_005819494.1	XP_005998952	
Latimeria chalumnae	coelacanth	*RABGGTA*	NW_005820340.1	XP_014350255	
Latimeria chalumnae	coelacanth	*TGM1*	NW_005820340.1	XP_014350256	corrected with SRR391920*
Latimeria chalumnae	coelacanth	*TGM1L3*	NW_005820340.1	XP_006006388	
Latimeria chalumnae	coelacanth	*TGM1L1*	scaffold NW_005824701.1	XP_006014051	
Latimeria chalumnae	coelacanth	*TGM1L4*	scaffold NW_005820704.1	XP_006008135	corrected with SRR391920*
Latimeria chalumnae	coelacanth	*TGM1L2*	scaffold NW_005820704.1	XP_006008136	
Latimeria chalumnae	coelacanth	*TINF2*	scaffold NW_005820704.1	XP_006008144	
Latimeria chalumnae	coelacanth	*GMPR2*	scaffold NW_005820704.1	XP_006008141	
Latimeria chalumnae	coelacanth	*NEDD8*	scaffold NW_005820704.1	XP_006008142	
Latimeria chalumnae	coelacanth	*MDP1*	scaffold NW_005822223.1	XP_006012405.1	
Danio rerio	zebrafish	*FEN1*	chromosome 24	NP_001315436	
Danio rerio	zebrafish	*CIDEB*	chromosome 7	NP_001243186	
Danio rerio	zebrafish	*RABGGTA*	chromosome 23	NP_001070131	
Danio rerio	zebrafish	*TGM1*	chromosome 23	XP_009294851	
Danio rerio	zebrafish	*TGM1L2*	chromosome 23	XP_009295369	
Danio rerio	zebrafish	*TGM1L6*	chromosome 23	XP_021325715	
Danio rerio	zebrafish	*TGM1L3*	chromosome 23	XP_001331914	
Danio rerio	zebrafish	*TGM1L5*	chromosome 23	XP_009295379	
Danio rerio	zebrafish	*TGM1L4*	chromosome 23	NP_001025267	
Danio rerio	zebrafish	*TINF2*	chromosome 7	XP_005172357	
Danio rerio	zebrafish	*GMPR2*	chromosome 7	NP_001035394	
Danio rerio	zebrafish	*NEDD8*	chromosome 7	NP_958478	
Danio rerio	zebrafish	*MDP1*	chromosome 23	XP_685841	
Danio rerio	zebrafish	TGM1L1	chromosome 2	XP_694950	
Carcharodon carcharias	great white shark	*FEN1*	chromosome 3	XP_041039310	
Carcharodon carcharias	great white shark	*CIDEB*	chr 27 scaffold NW_024470624.1	XP_041036706	
Carcharodon carcharias	great white shark	*RABGGTA*	chromosome 27	XP_041031372	
Carcharodon carcharias	great white shark	*TGM1*	chromosome 27	XP_041031332	
Carcharodon carcharias	great white shark	*TINF2*	chromosome 7	XP_041048560	
Carcharodon carcharias	great white shark	*NEDD8*	chromosome 1	XP_041049449	

Carcharodon carcharias	great white shark	*MDP1*	chr 24 scaffold NW_024470615.1	XP_041036657	
Petromyzon marinus	sea lamprey	*FEN1*	chromosome 63	XP_032833433	
Petromyzon marinus	sea lamprey	*CIDEB*	chromosoem 69	XP_032834929	
Petromyzon marinus	sea lamprey	*TGM1L3*	chromosome 69	XP_032834804	
Petromyzon marinus	sea lamprey	*RABGGTA*	scaffold NW_022638414.1	XP_032800045	
Petromyzon marinus	sea lamprey	*TGM1L4*	chromosome 25	XP_032816216	
Petromyzon marinus	sea lamprey	*TGM1L1*	chromosome 25	XP_032816217	
Petromyzon marinus	sea lamprey	*GMPR2*	chromosome 25	XP_032816349	
Petromyzon marinus	sea lamprey	*NEDD8*	chromosome 54	XP_032830533	
Ciona intestinalis	vase tunicate	*FEN1*	scaffold NW_004190443.2	XP_009861628	
Ciona intestinalis	vase tunicate	*MDP1*	scaffold NW_004190441.2	XP_002130277	
Ciona intestinalis	vase tunicate	*TGML3*	scaffold NW_004190441.2	XP_009861590	annotated as tgm2 in GenBank
Branchiostoma floridae	lancelet	*FEN1*	chromosome 6	XP_035680338	
Branchiostoma floridae	lancelet	*RABGGTA*	chromosome 6	XP_035678477	
Branchiostoma floridae	lancelet	*TGM1L3*	chromosome 6	XP_035678632	
Branchiostoma floridae	lancelet	*TGM1L5*	chromosome 6	XP_035678634	
Branchiostoma floridae	lancelet	*TGM1L4*	chromosome 6	XP_035678628	
Branchiostoma floridae	lancelet	*TGM1L7*	chromosome 6	XP_035678631	
Branchiostoma floridae	lancelet	*TGM1L1*	chromosome 6	XP_035678619	
Branchiostoma floridae	lancelet	*TGM1L2*	chromosome 6	XP_035678971	
Branchiostoma floridae	lancelet	*MDP1*	chromosome 6	XP_035678775	
Branchiostoma floridae	lancelet	*TGM1L6*	chromosome 11	XP_035691780	

* The amino acid sequence is shown in Figure S1.

Table S3. GenBank accession numbers for genes close to the F13A1 gene (locus shown in Figure S5)

Species	name	Gene	chromosome/scaffold	NCBI GenBank accession number
Homo sapiens	human	*NRN1*	chromosome 6	NP_001265639
Homo sapiens	human	*F13A1*	chromosome 6	NP_000120
Homo sapiens	human	*LY86*	chromosome 6	NP_004262
Gallus gallus	chicken	*FARS2*	chromosome 2	XP_040552223
Gallus gallus	chicken	*NRN1*	chromosome 2	XP_046780218
Gallus gallus	chicken	*F13A1*	chromosome 2	NP_990016
Gallus gallus	chicken	*LY86*	chromosome 2	NP_001004399
Gallus gallus	chicken	*RREB1*	chromosome 2	XP_015131325
Anolis carolinensis	lizard	*FARS2*	scaffold NW_003338968.1	XP_003226906
Anolis carolinensis	lizard	*NRN1*	scaffold NW_003338968.1	XP_003226908
Anolis carolinensis	lizard	*F13A1*	scaffold NW_003338968.1	XP_016852585
Xenopus tropicalis	clawed frog	*FARS2*	chromosome 6	XP_031759688
Xenopus tropicalis	clawed frog	*NRN1*	chromosome 6	NP_001120071
Xenopus tropicalis	clawed frog	*F13A1*	chromosome 6	XP_031759894
Xenopus tropicalis	clawed frog	*RREB1*	chromosome 6	XP_002932722
Protopterus annectens	lungfish	*FARS2*	chromosome 3.part0	XP_043920793
Protopterus annectens	lungfish	*NRN1*	chromosome 3.part0	XP_043920795
Protopterus annectens	lungfish	*F13A1*	chromosome 3.part0	XP_043920796
Protopterus annectens	lungfish	*LY86*	chromosome 3.part0	XP_043920797
Latimeria chalumnae	coelacanth	*NRN1*	scaffold NW_005821405.1	XP_006010651
Latimeria chalumnae	coelacanth	*F13A1*	scaffold NW_005821405.1	XP_014352948
Danio rerio	zebrafish	*FARS2*	chromosome 24	NP_001099064
Danio rerio	zebrafish	*NRN1*	chromosome 24	NP_001002507
Danio rerio	zebrafish	*F13A1a.1*	chromosome 24	NP_001070179
Danio rerio	zebrafish	*F13A*	chromosome 24	XP_686649
Danio rerio	zebrafish	*LY86*	chromosome 24	XP_009295493
Danio rerio	zebrafish	*RREB1*	chromosome 24	XP_002666628
Danio rerio	zebrafish	*F13A1b*	chromosome 7	XP_021333236
Carcharodon carcharias	shark	*FARS2*	chromosome 3	XP_041039422
Carcharodon carcharias	shark	*NRN1*	chromosome 3	XP_041040407
Carcharodon carcharias	shark	F13A1	chromosome 3	XP_041040322
Carcharodon carcharias	shark	*RREB1*	chromosome 3	XP_041039672
Petromyzon marinus	sea lamprey	*FARS2*	chromosome 45	XP_032827167
Petromyzon marinus	sea lamprey	*NRN1*	chromosome 45	XP_032827071
Petromyzon marinus	sea lamprey	*TGM1L2*	chromosome 45	XP_032827092
Petromyzon marinus	sea lamprey	*F13A1L*	chromosome 63	XP_032833350
Branchiostoma floridae	lancelet	*FARS2*	chromosome 6	XP_035679726
Branchiostoma floridae	lancelet	*F13A1L*	chromosome 4	XP_035674033

Table S4. Proteomic analysis of cornified teeth of the sea lamprey (*Petromyzon marinus*)

Rank	Accession	Annotation	Protein name (GenBank)	# AAs	MW [kDa]	calc. pI	Coverage [%]	# Peptides	# Unique Peptides	# PSMs	Score
1	XP_032832261.1	Keratin	keratin, type I cytoskeletal 19-like	458	50,3	5,27	99	66	66	984	1974,9
2	XP_032836208.1	Keratin	keratin, type II cytoskeletal cochleal-like	521	57,8	6,25	74	65	61	927	1798,8
3	XP_032812944.1	Keratin	keratin, type II cytoskeletal 8-like	495	53,5	6,42	81	70	34	431	867,4
4	XP_032814531.1	Keratin	keratin, type 1 cytoskeletal 11-like	448	48,6	5,87	71	57	48	430	818,6
5	XP_032812945.1	Keratin	keratin, type II cytoskeletal 8-like	484	52,6	5,71	76	55	12	233	383,1
6	XP_032812943.1	Keratin	keratin, type II cytoskeletal 8-like	510	55,7	6,25	75	50	22	210	372,5
7	XP_032834074.1		glycine-rich cell wall structural protein 2-like	220	21,4	8,68	19	4	4	98	184,0
8	XP_032808690.1	MACF1-like	microtubule-actin cross-linking factor 1-like	2636	303,3	6,43	27	59	59	113	167,7
9	XP_032820218.1		cadherin-2-like	1008	106,3	6,11	21	15	15	46	101,6
10	XP_032816031.1		plakophilin-3-like	942	103,4	9,23	33	28	28	48	96,2
11	XP_032806953.1		catenin beta-1-like	780	83,8	5,94	25	16	14	43	81,0
12	XP_032817362.1		fatty acid-binding protein, heart-like	133	14,9	7,09	50	8	8	38	63,6
13	XP_032809983.1		filamin-A-like	2528	266,4	5,69	11	18	18	30	62,9
14	XP_032817193.1		arachidonate 5-lipoxygenase-like	668	75,5	6,05	31	18	18	41	62,6
15	XP_032837300.1	Keratin	keratin, type 1 cytoskeletal 11-like	387	42,7	5,16	18	9	1	43	61,6
16	XP_032800492.1	Keratin	keratin, type 1 cytoskeletal 11-like, partial	409	44,3	5,35	20	12	2	44	61,0
17	AJG41731.1		beta-actin	376	41,8	5,48	49	13	3	30	50,2
18	XP_032816216.1	**TGM1L4**	protein-glutamine gamma-glutamyltransferase 4-like	787	84,8	6,83	31	18	18	30	45,5
19	XP_032816807.1		uncharacterized protein LOC116946080	217	24,4	9,54	46	9	9	22	43,5
20	XP_032801611.1		intermediate filament protein ON3-like	476	50,9	6,07	13	7	1	38	43,3
21	XP_032817105.1		actin, cytoplasmic 2-like	376	41,8	5,48	31	10	1	23	40,8
22	XP_032804442.1		actin, alpha cardiac muscle 2	377	42	5,39	24	8	2	18	32,7
23	XP_032801610.1	Keratin	keratin, type II cytoskeletal 8-like	475	53,8	8,06	6	3	1	23	25,5
24	XP_032802783.1		glyceraldehyde-3-phosphate dehydrogenase	333	36	7,2	12	3	3	10	25,4
25	XP_032832439.1		tubulin beta-4 chain-like	445	49,8	4,89	16	6	6	16	23,4
26	XP_032822326.1		uncharacterized protein LOC116949299 isoform X1	920	100,4	5,55	7	4	2	12	23,1
27	XP_032834033.1		abscisic acid and environmental stress-inducible protein-like	199	20,2	8,16	34	7	1	12	22,9
28	XP_032823532.1		clustered mitochondria protein homolog isoform X1	2053	220,4	6,38	5	8	8	11	22,4
29	XP_032806848.1		lamin-B1-like isoform X1	783	84,8	6,39	17	11	11	15	21,9
30	XP_032803858.1		annexin A2-like	332	37	7,18	14	5	5	8	21,0
31	XP_032834035.1		cold and drought-regulated protein CORA-like	188	18,9	8,19	32	7	1	12	20,7
32	XP_032824491.1		elongation factor 2	858	95,4	6,74	8	4	4	8	19,4
33	XP_032825520.1		protein-arginine deiminase type-2-like	992	110,1	7,08	10	9	9	12	19,1
34	XP_032803022.1		14-3-3 protein gamma-A-like	248	28,4	4,84	19	5	4	9	18,7
35	XP_032800944.1		serum albumin SDS-1	1423	159,1	6,51	8	9	9	10	18,1
36	XP_032834085.1	not FLG2-like	filaggrin-2-like	1141	117	7,72	5	5	5	9	17,5
37	XP_032823604.1		14-3-3 protein epsilon-like	258	29,4	4,82	16	4	3	8	17,2
38	XP_032819215.1		tubulin alpha chain, testis-specific-like	450	50,2	5,1	13	4	2	10	15,6
39	XP_032833891.1		cold and drought-regulated protein CORA-like	173	17,4	8,4	36	5	1	8	14,6
40	XP_032815474.1		catenin beta-1	777	85,3	5,87	5	3	1	8	14,6
41	AAN73382.1		ribosomal protein L18, partial	188	21,5	11,77	23	4	4	6	13,8
42	XP_032811538.1		40S ribosomal protein S3	249	27,3	9,58	16	4	4	5	13,8
43	XP_032819572.1		histone H4	103	11,4	11,36	51	6	6	8	13,3

44	XP_032829724.1		pyruvate kinase PKM	529	57,6	6,29	12	5	5	6	12,9
45	XP_032811593.1		calpain-1 catalytic subunit-like	878	97	8,41	8	6	6	7	12,8
46	XP_032820277.1		heat shock cognate 71 kDa protein isoform X1	651	71,3	5,66	7	5	5	6	12,5
47	AAA49267.1		lactate dehydrogenase	334	36,4	7,31	15	4	4	5	11,8
48	XP_032833791.1		profilin-1-like	139	15,1	7,05	27	2	2	6	11,8
49	XP_032817266.1	plectin	LOW QUALITY PROTEIN: plectin-like	2752	308,3	5,74	12	7	2	7	11,0
50	XP_032811511.1	plectin	plectin-like	897	97,3	8,7	5	6	6	6	10,7
51	XP_032803290.1		annexin A2 isoform X1	396	44	8,15	9	3	3	4	10,2
52	XP_032833379.1		hydroxyacylglutathione hydrolase, mitochondrial isoform X1	324	35,1	7,83	8	2	2	3	9,9
53	XP_032817272.1	plectin	LOW QUALITY PROTEIN: plectin-like	2038	227,5	5,44	11	7	2	7	9,8
54	P07096.1		RecName: Full=Blood plasma apolipoprotein LAL2; Flags: Precursor	191	20,5	6,4	34	5	5	8	9,7
55	XP_032801167.1		nucleoside diphosphate kinase-like isoform X1	149	16,7	7,12	22	4	4	4	9,6
56	XP_032809885.1		transitional endoplasmic reticulum ATPase-like	811	89	5,31	4	3	3	4	8,5
57	XP_032799905.1		T-complex protein 1 subunit zeta	531	57,5	7,02	6	3	3	5	8,1
58	XP_032805864.1		retinal dehydrogenase 2-like	520	56,1	5,49	8	3	3	4	7,5
59	XP_032800408.1		T-complex protein 1 subunit alpha isoform X3	559	59,7	6,55	9	5	5	6	7,4
60	XP_032830674.1		40S ribosomal protein SA	298	33	4,87	13	3	3	4	7,1
61	XP_032824816.1		ATP synthase subunit alpha, mitochondrial isoform X1	553	60	9,25	7	3	3	4	6,9
62	XP_032831352.1		ATP synthase subunit beta, mitochondrial	524	56,1	5,27	8	3	3	4	6,7
63	XP_032815375.1		apolipoprotein B-100	5361	586,4	6,98	1	4	4	4	6,7
64	XP_032806935.1		myosin light polypeptide 6-like isoform X1	151	16,9	4,5	13	2	2	4	6,5
65	XP_032817138.1		myosin-10-like isoform X1	1981	228,5	5,63	1	3	3	4	6,3
66	XP_032807738.1		vimentin-like	446	50,9	5,34	4	2	2	11	6,3
67	XP_032816217.1	**TGM1L1**	protein-glutamine gamma-glutamyltransferase K-like	811	88,8	6,13	3	3	3	3	6,2
68	XP_032812044.1		alpha-actinin-1-like isoform X1	890	102,4	5,53	6	4	4	4	5,9
69	XP_032828431.1		40S ribosomal protein S4, X isoform	263	29,5	10,17	11	3	3	4	5,8
70	XP_032813838.1		T-complex protein 1 subunit beta	535	57,9	6,44	5	2	2	2	5,6
71	XP_032817596.1		elongation factor 1-alpha, somatic form	463	50,2	9	8	2	2	3	5,5
72	XP_032815154.1		CD109 antigen-like	1410	154,1	6,74	4	4	4	4	5,5
73	AAZ83742.1		carbonic anhydrase	262	28,8	5,76	9	3	3	3	5,5
74	XP_032819329.1		filamin-A-like isoform X1	2608	276,7	6,38	2	3	3	4	5,3
75	XP_032819899.1		cytoplasmic dynein 1 heavy chain 1-like isoform X1	4637	529,7	6,48	1	3	3	4	5,3
76	XP_032833892.1		keratin-associated protein 5-1-like	468	47,9	8,28	18	2	2	5	5,3
77	XP_032820625.1		calpain-2 catalytic subunit-like	944	105	7,81	3	2	2	2	5,1
78	XP_032831572.1		pyruvate kinase PKM-like isoform X1	532	58,2	7,94	4	2	2	2	5,1
79	XP_032818217.1		enolase	434	47,5	7,44	7	2	2	3	5,0
80	XP_032804478.1		calpain-1 catalytic subunit-like	706	79,6	4,98	4	3	3	3	5,0
81	XP_032810903.1	plectin	plectin-like	745	84,5	6,43	8	3	3	3	4,9
82	AAM88904.1		guanine nucleotide-binding protein	317	35,1	7,44	7	2	2	3	4,5
83	XP_032806574.1		eukaryotic translation initiation factor 3 subunit A isoform X1	1373	163,1	7,5	3	3	3	3	4,5
84	XP_032818000.1		proteasome subunit beta type-6	233	25,1	6,2	5	2	2	2	4,5
85	XP_032834739.1		prosaposin-like	372	39,4	6,87	7	2	2	2	4,3
86	XP_032834804.1	**TGM1L3**	protein-glutamine gamma-glutamyltransferase K-like isoform X1	856	91,7	6,14	3	2	2	2	4,3
87	XP_032801469.1		alkaline phosphatase, tissue-nonspecific isozyme, partial	347	38,1	6,24	8	2	2	2	4,3
88	XP_032814482.1		40S ribosomal protein S26	118	13,3	11	16	2	2	2	4,1
89	XP_032831750.1		T-complex protein 1 subunit gamma	560	61,2	7,8	5	2	1	3	4,0
90	XP_032836002.1		eIF-2-alpha kinase activator GCN1	2694	288,3	6,89	2	3	3	3	4,0

91	XP_032830511.1		gigaxonin	618	68,8	5,95	4	2	2	2	4,0
92	XP_032837319.1	Keratin	keratin, type I cytoskeletal 13-like	829	84,8	8,44	3	2	2	2	3,8
93	XP_032826392.1		cuticle collagen 6-like	191	18,6	7,42	12	2	2	2	3,8
94	XP_032834723.1		aerolysin-like protein	313	34,1	6,57	10	2	2	2	3,6
95	XP_032813046.1		uncharacterized protein LOC116943873	1706	182,2	5,68	1	2	2	2	3,6
96	XP_032834312.1		transmembrane protein 47-like	180	19,6	8,66	11	2	2	2	3,5
97	XP_032815156.1		CD109 antigen-like isoform X1	1443	154	5,4	2	2	2	2	3,0
98	XP_032832495.1		cystatin-B-like	99	11,3	8,18	19	2	2	2	2,2
99	XP_032814865.1		glutathione peroxidase 6-like	145	16,8	6,54	8	2	2	2	2,2
100	XP_032833350.1	**F13A1L**	LOW QUALITY PROTEIN: coagulation factor XIII A chain-like	804	88	6,38	3	2	2	2	2,2
101	XP_032831821.1		perilipin-3-like	402	42,5	7,03	4	2	2	2	2,1
102	XP_032822285.1		actin-related protein 2/3 complex subunit 4	168	19,7	8,43	11	2	2	2	2,0
103	XP_032803479.1		copper chaperone for superoxide dismutase	279	28,7	6,84	14	3	3	3	1,9
104	XP_032802310.1		aldo-keto reductase family 1 member B7-like	335	37	7,18	5	2	2	2	1,8
105	XP_032817391.1		myosin regulatory light chain 12A	174	20	4,81	23	2	2	3	1,7
106	XP_032829378.1		annexin A4-like	524	55,9	8,13	3	2	2	2	0,0
107	XP_032830825.1		T-complex protein 1 subunit eta	539	58,3	5,87	4	2	1	2	0,0

Notes: AA, amino acid residue; PSM, peptide spectrum match. Transglutaminases are highlighted by red fonts.

Table S5. GenBank accession numbers for genes close to the TGM4 gene (locus shown in Figure S6)

Species	name	Gene	chromosome/scaffold	NCBI GenBank accession	notes
Homo sapiens	human	*KIFF15*	chromosome 3	XP_016862373	
Homo sapiens	human	*TMEM42*	chromosome 3	NP_653239	
Homo sapiens	human	*TGM4*	chromosome 3	NP_003232	
Homo sapiens	human	*ZDHHC3*	chromosome 3	NP_001336306	
Homo sapiens	human	*EXOSC7*	chromosome 3	NP_055819	
Talpa occidentalis	Iberian mole	*KIFF15*	scaffold NW_023600993.1	XP_037383430	
Talpa occidentalis	Iberian mole	*TMEM42*	scaffold NW_023600993.1	XP_037383484	
Talpa occidentalis	Iberian mole	*ZDHHC3*	scaffold NW_023600993.1	XP_037383438	
Talpa occidentalis	Iberian mole	*EXOSC7*	scaffold NW_023600993.1	XP_037383516	
Elephas maximus indicus	indian elephant	*KIFF15*	chromosome 20	XP_049718971	
Elephas maximus indicus	indian elephant	*TMEM42*	chromosome 20	XP_049718656	
Elephas maximus indicus	indian elephant	*TGM4*	chromosome 20	XP_049718014	premature stop codon
Elephas maximus indicus	indian elephant	*ZDHHC3*	chromosome 20	XP_049717968	
Elephas maximus indicus	indian elephant	*EXOSC7*	chromosome 20	XP_049718405	
Sarcophilus harrisii	Tasmanian devil	*KIFF15*	chromosome 5	XP_023360919	
Sarcophilus harrisii	Tasmanian devil	*TMEM42*	chromosome 5	XP_031795931	
Sarcophilus harrisii	Tasmanian devil	*ZDHHC3*	chromosome 5	XP_031795939	
Sarcophilus harrisii	Tasmanian devil	*EXOSC7*	chromosome 5	XP_031795933	
Gallus gallus	chicken	*KIFF15*	chromosome 2	XP_015137220	
Gallus gallus	chicken	*TMEM42*	chromosome 2	XP_046794130	
Gallus gallus	chicken	*TGM4*	chromosome 2	NP_001006368	
Gallus gallus	chicken	*ZDHHC3*	chromosome 2	XP_046794123	
Gallus gallus	chicken	*EXOSC7*	chromosome 2	NP_001239083	
Anolis carolinensis	lizard	*KIFF15*	scaffold NW_003338771.1	XP_008113746	
Anolis carolinensis	lizard	*TGM4*	scaffold NW_003338771.1	XP_008113745	
Anolis carolinensis	lizard	*TGM4L1*	scaffold NW_003338771.1	XP_008113741	
Anolis carolinensis	lizard	*TGM4L2*	scaffold NW_003338771.1	XP_008113740	
Anolis carolinensis	lizard	*EXOSC7*	scaffold NW_003338771.1	XP_008113738	
Xenopus tropicalis	clawed frog	*KIFF15*	chromosome 6	XP_012820972	
Xenopus tropicalis	clawed frog	*TMEM42*	chromosome 6	XP_031760415	
Xenopus tropicalis	clawed frog	*TGM4*	chromosome 6	XP_002937794	
Xenopus tropicalis	clawed frog	*TGM4L*	chromosome 6	XP_002937793	
Protopterus annectens	lungfish	*KIFF15*	chromosome 3.part0	XP_043921457	
Protopterus annectens	lungfish	*TMEM42*	chromosome 3.part0	XP_043920603	
Protopterus annectens	lungfish	*ZDHHC3*	chromosome 3.part0	XP_043920605	
Protopterus annectens	lungfish	*EXOSC7*	chromosome 3.part0	XP_043920604	
Latimeria chalumnae	coelacanth	*KIFF15*	scaffold NW_005819503.1	XP_014345707	
Latimeria chalumnae	coelacanth	*TMEM42*	scaffold NW_005819503.1	XP_014345717	
Latimeria chalumnae	coelacanth	*TGM4*	scaffold NW_005819503.1	XP_005999060	
Latimeria chalumnae	coelacanth	*ZDHHC3*	scaffold NW_005819503.1	XP_005999059	
Latimeria chalumnae	coelacanth	*EXOSC7*	scaffold NW_005819503.1	XP_005999056	
Danio rerio	zebrafish	*TMEM42*	chromosome 16	XP_002665001	
Danio rerio	zebrafish	*ZDHHC3*	chromosome 16	NP_001002725	
Scleropages formosus	asian bonytongue	*TMEM42*	chromosome 8	XP_018610022	
Scleropages formosus	asian bonytongue	*TGM4*	chromosome 8	XP_018610188	
Scleropages formosus	asian bonytongue	*EXOSC7*	chromosome 8	XP_018610063	
Polypterus senegalus	grey bichir	*KIFF15*	chromosome 15	XP_039592109	
Polypterus senegalus	grey bichir	*TMEM42*	chromosome 15	XP_039592112	
Polypterus senegalus	grey bichir	*TGM4*	chromosome 15	XP_039592984	
Polypterus senegalus	grey bichir	*TGM4L*	chromosome 15	XP_039593036	
Polypterus senegalus	grey bichir	*ZDHHC3*	chromosome 15	XP_039593279	
Polypterus senegalus	grey bichir	*EXOSC7*	chromosome 15	XP_039593563	
Carcharodon carcharias	shark	*KIFF15*	chromosome 3	XP_041040885	
Carcharodon carcharias	shark	*TMEM42*	chromosome 3	XP_041039598	
Carcharodon carcharias	shark	*ZDHHC3*	chromosome 3	XP_041039594	
Carcharodon carcharias	shark	*EXOSC7*	chromosome 3	XP_041040774	
Petromyzon marinus	lamprey	*TMEM42*	chromosome 20	XP_032813716	
Petromyzon marinus	lamprey	*ZDHHC3*	chromosome 20	XP_032813706	
Petromyzon marinus	lamprey	*EXOSC7*	chromosome 20	XP_032813704	

Table S6. GenBank accession numbers for genes close to the TGM2-7 and EPB42 genes (locus shown in Figure 3)

Species	name	Gene	chromosome/scaffold	NCBI GenBank accession number
Homo sapiens	human	*CCNDBP1*	chromosome 15	NP_036274
Homo sapiens	human	*EPB42*	chromosome 15	NP_000110
Homo sapiens	human	*TGM5*	chromosome 15	NP_963925
Homo sapiens	human	*TGM7*	chromosome 15	NP_443187
Homo sapiens	human	*TGM3*	chromosome 20	NP_003236
Homo sapiens	human	*TGM6*	chromosome 20	NP_945345
Homo sapiens	human	*TTI1*	chromosome 20	XP_047296562
Homo sapiens	human	*TGM2*	chromosome 20	NP_004604
Gallus gallus	chicken	*ZNFX1*	chromosome 20	XP_015152165
Gallus gallus	chicken	*CCNDBP1*	chromosome 20	XP_015152166
Gallus gallus	chicken	*EPB42*	chromosome 20	NP_001308488
Gallus gallus	chicken	*TGM3*	chromosome 20	XP_040544622
Gallus gallus	chicken	*TGM6*	chromosome 20	XP_040544619
Gallus gallus	chicken	*TGM6L*	chromosome 20	XP_015152172
Gallus gallus	chicken	*TTI1*	chromosome 20	XP_001234099
Gallus gallus	chicken	*TGM2*	chromosome 20	NP_001378925
Anolis carolinensis	lizard	*ZNFX1*	scaffold NW_003339367.1	XP_008121304
Anolis carolinensis	lizard	*CCNDBP1*	scaffold NW_003339367.1	XP_003229186
Anolis carolinensis	lizard	*EPB42*	scaffold NW_003339367.1	XP_008121298
Anolis carolinensis	lizard	*TGM5*	scaffold NW_003339367.1	XP_008121297
Anolis carolinensis	lizard	*TGM3L1*	scaffold NW_003339418.1	XP_016854018
Anolis carolinensis	lizard	*TGM3L2*	scaffold NW_003340348.1	XP_003230298
Anolis carolinensis	lizard	*TGM3L3*	scaffold NW_003339429.1	XP_003229383
Anolis carolinensis	lizard	*TTI1*	scaffold NW_003339119.1	XP_008119774
Anolis carolinensis	lizard	*TGM2*	scaffold NW_003339119.1	XP_008119776
Anolis carolinensis	lizard	*TGM2L*	scaffold NW_003339119.1	XP_016853249
Xenopus tropicalis	clawed frog	*ZNFX1*	chromosome 10	XP_012808186
Xenopus tropicalis	clawed frog	*CCNDBP1*	chromosome 10	XP_002935599
Xenopus tropicalis	clawed frog	*EPB42*	chromosome 10	XP_002935587
Xenopus tropicalis	clawed frog	*TGM5*	chromosome 10	XP_031750630
Xenopus tropicalis	clawed frog	*TGM7*	chromosome 10	XP_031750714
Xenopus tropicalis	clawed frog	*TGM3L8*	chromosome 10	XP_002935578
Xenopus tropicalis	clawed frog	*TGM3L7*	chromosome 10	XP_031750709
Xenopus tropicalis	clawed frog	*TGM3L9*	chromosome 10	XP_031750472
Xenopus tropicalis	clawed frog	*TGM3L1*	chromosome 10	XP_031750711
Xenopus tropicalis	clawed frog	*TGM3L2*	chromosome 10	XP_004918837
Xenopus tropicalis	clawed frog	*TGM3L*	chromosome 10	NP_001120399
Xenopus tropicalis	clawed frog	*TGM3L4*	chromosome 10	XP_031750708
Xenopus tropicalis	clawed frog	*TGM3L5*	chromosome 10	NP_001361172
Xenopus tropicalis	clawed frog	*TTI1*	chromosome 10	XP_031749805
Xenopus tropicalis	clawed frog	*TGM2*	chromosome 10	NP_001123852
Protopterus annectens	lungfish	*ZNFX1*	chromosome 12.part0	XP_043946062
Protopterus annectens	lungfish	*TGM5L*	chromosome 12.part0	XP_043946061
Protopterus annectens	lungfish	*TTI1*	chromosome 12.part0	XP_043946132
Protopterus annectens	lungfish	*TGM2*	chromosome 12.part0	XP_043946131
Latimeria chalumnae	coelacanth	*ZNFX1*	NW_005819908.1	XP_006003202
Latimeria chalumnae	coelacanth	*CCNDBP1*	NW_005819908.1	XP_006003203
Latimeria chalumnae	coelacanth	*TTI1*	scaffold NW_005820602.1	XP_014351079
Latimeria chalumnae	coelacanth	*TGM2*	scaffold NW_005820602.1	XP_006007736
Danio rerio	zebrafish	*CCNDBP1*	chromosome 6	NP_001082834
Danio rerio	zebrafish	*TGM8*	chromosome 6	XP_009301053
Danio rerio	zebrafish	*TGM5L*	chromosome 6	NP_001313420
Danio rerio	zebrafish	*TGM2b*	chromosome 6	NP_997821
Danio rerio	zebrafish	*TTI1*	chromosome 23	XP_698602
Danio rerio	zebrafish	*TGM2a*	chromosome 23	NP_001004647
Carcharodon carcharias	shark	*ZNFX1*	chromosome 14	XP_041059921
Carcharodon carcharias	shark	*CCNDBP1*	chromosome 14	XP_041060851
Carcharodon carcharias	shark	*TGM2L*	chromosome 14	XP_041060189
Carcharodon carcharias	shark	*TGM2L2*	chromosome 14	XP_041060188
Carcharodon carcharias	shark	*TGM2L3*	chromosome 14	XP_041060568
Carcharodon carcharias	shark	*TTI1*	chromosome 14	XP_041060292
Carcharodon carcharias	shark	*TGM2L4*	chromosome 14	XP_041060955
Petromyzon marinus	lamprey	*CCNDBP1*	chromosome 45	XP_032827172
Petromyzon marinus	lamprey	*TTI1*	chromosome 63	XP_032833264
Ciona intestinalis	vase tunicate	*ZNFX1*	chromosome 10	XP_009860073
Ciona intestinalis	vase tunicate	*TTI1*	chromosome 11	XP_002130662
Branchiostoma floridae	lancelet	*ZNFX1*	chromosome 6	XP_035678891
Branchiostoma floridae	lancelet	*CCNDBP1*	chromosome 6	XP_035680453
Branchiostoma floridae	lancelet	*TTI1*	chromosome 14	XP_035697320

Table S7. GenBank accessions for genes close to the TGM10 gene (Locus shown in Figure 3)

Species	name	Gene	chromosome/scaffold	NCBI GenBank accession number
Homo sapiens	human	*PLLP*	chromosome 16	NP_057077
Homo sapiens	human	*SLC9A5*	chromosome 16	NP_004585
Gallus gallus	chicken	*PLLP*	chromosome 11	XP_046755361
Gallus gallus	chicken	*SLC9A5*	chromosome 11	XP_003641949
Anolis carolinensis	lizard	*PLLP*	scaffold NW_003339224.1	XP_003228650
Xenopus tropicalis	clawed frog	*PLLP*	chromosome 4	XP_002931714
Xenopus tropicalis	clawed frog	*SLC9A5*	chromosome 4	XP_002931737
Protopterus annectens	lungfish	*PLLP*	chromosome 8.part0	XP_043937332
Protopterus annectens	lungfish	*TMPPE*	chromosome 8.part0	XP_043937331
Protopterus annectens	lungfish	*TGM10*	chromosome 8.part0	XP_043935473
Protopterus annectens	lungfish	*SLC9A5*	chromosome 8.part0	XP_043937321
Latimeria chalumnae	coelacanth	*PLLP*	scaffold NW_005819374.1	XP_005997249
Latimeria chalumnae	coelacanth	*TMPPE*	scaffold NW_005819374.1	XP_005997248
Latimeria chalumnae	coelacanth	*TGM10*	scaffold NW_005819374.1	XP_005997256
Latimeria chalumnae	coelacanth	*SLC9A5*	scaffold NW_005819374.1	XP_005997255
Danio rerio	zebrafish	*PLLP*	chromosome 18	NP_001116171
Danio rerio	zebrafish	*TGM10*	chromosome 18	XP_687398
Danio rerio	zebrafish	*SLC9A5*	chromosome 7	NP_001106943
Carcharodon carcharias	shark	*PLLP*	chromosome 7	XP_041048331
Carcharodon carcharias	shark	*TMPPE*	chromosome 7	XP_041047103
Carcharodon carcharias	shark	*TGM10*	chromosome 7	XP_041047102
Carcharodon carcharias	shark	*SLC9A5*	chromosome 7	XP_041047607
Petromyzon marinus	sea lamprey	*TMPPE*	chromosome 25	XP_032816663
Petromyzon marinus	sea lamprey	*TGM10*	chromosome 25	XP_032816667
Ciona intestinalis	vase tunicate	*TMPPE*	scaffold NW_004190356.2	XP_004226985

Table S8. Tissue transcriptomes of the zebrafish (*Danio rerio*), reference genome GCF_000002035.6, that were analyzed in this study.

Tissue type	SRA GenBank accession number	SRA GenBank run accession number	Transcriptome size [Gb]
adult female skin 1	SRX15614020	SRR19561930	8.8
adult female skin 2	SRX15614013	SRR19561937	7.4
adult female skin 3	SRX15614007	SRR19561943	7.0
adult female liver 1	SRX15614024	SRR19561926	6.8
adult female liver 2	SRX15614018	SRR19561932	7.5
adult female liver 3	SRX15614011	SRR19561939	5.9
adult female gill 1	SRX15614015	SRR19561935	7.2
adult female gill 2	SRX15614022	SRR19561928	15.5
adult female gill 3	SRX15614009	SRR19561941	7.7
adult female muscle 1	SRX15614025	SRR19561925	7.1
adult female muscle 2	SRX15614019	SRR19561931	10.1
adult female muscle 3	SRX15614012	SRR19561938	7.6
adult female ovary 1	SRX15614023	SRR19561927	7.0
adult female ovary 2	SRX15614016	SRR19561934	8.3
adult female ovary 3	SRX15614010	SRR19561940	10.7
adult female brain 1	SRX15614021	SRR19561929	8.5
adult female brain 2	SRX15614017	SRR19561933	7.0
adult female brain 3	SRX15614014	SRR19561936	7.1

Notes: Gb, gigabases; SRA, sequence read archive.

Table S9. Expression levels of TGMs and keratin markers in mouse hair follicles, as determined by scRNA-seq (Joost et al. 2020)

Gene	Medulla 1 (MED1)	Medulla 2 (MED2)	Medulla 3 (MED3)	Cortex / cuticle 1 (CX1)	Cortex / cuticle 2 (CX2)	Cortex / cuticle 3 (CX3)	Cortex / cuticle 4 (CX4)	Cortex / cuticle 5 (CX5)	Inner root sheath 1 (IRS1)	Inner root sheath 2 (IRS2)	Inner root sheath 3 (IRS3)	Inner root sheath 4 (IRS4)	Inner root sheath 5 (IRS5)	Inner root sheath 6 (IRS6)	Middle companion layer (mCP)	Upper companion layer (uCP)	Suprabasal outer root sheath (ORS SB)	Basal outer root sheath 1 (ORS B1)	Basal outer root sheath 2 (ORS B2)	Lower proximal cup (LPC)	Germin. layer 1 (GL1)	Germin. layer 2 (GL2)	Germin. layer 3 (GL3)	Germin. layer 4 (GL4)
Transglutaminases																								
Tgm1	0,000	0,047	0,000	0,000	0,007	0,001	0,006	0,000	0,008	0,000	0,007	0,323	0,043	0,287	0,000	0,000	0,021	0,003	0,014	0,000	0,000	0,003	0,000	0,000
Tgm2	0,000	0,001	0,000	0,000	0,000	0,000	0,000	0,009	0,007	0,000	0,000	0,000	0,000	0,000	0,000	0,117	0,000	0,102	0,000	0,248	0,000	0,000	0,005	0,000
Tgm3	0,200	0,374	0,248	0,142	0,023	0,012	0,102	0,464	0,025	0,174	0,093	0,122	0,153	0,232	0,000	0,120	0,000	0,000	0,019	0,128	0,023	0,015	0,019	0,015
Tgm4	0,000	0,000	0,000	0,000	0,000	0,000	0,000	0,000	0,000	0,000	0,000	0,000	0,000	0,000	0,000	0,000	0,000	0,000	0,000	0,000	0,000	0,000	0,000	0,000
Tgm5	0,000	0,000	0,000	0,000	0,000	0,006	0,000	0,000	0,000	0,000	0,013	0,083	0,000	0,015	0,000	0,083	0,000	0,000	0,000	0,000	0,000	0,000	0,000	0,000
Tgm6	0,039	0,384	0,390	0,008	0,005	0,000	0,000	0,000	0,004	0,000	0,000	0,000	0,013	0,181	0,000	0,000	0,000	0,000	0,000	0,000	0,000	0,005	0,000	0,004
Tgm7	0,000	0,000	0,000	0,000	0,000	0,000	0,000	0,000	0,000	0,000	0,000	0,000	0,000	0,000	0,000	0,000	0,000	0,000	0,000	0,000	0,000	0,000	0,000	0,000
Keratins																								
Krt5	1,027	0,733	1,160	0,884	0,371	0,465	0,300	0,313	0,683	0,594	0,567	0,316	0,378	0,089	18,927	10,013	13,276	6,735	5,168	4,012	1,384	1,271	1,045	1,217
Krt14	0,943	0,736	0,807	0,823	0,615	0,441	0,206	0,511	0,391	0,525	0,255	0,561	0,322	0,390	68,109	37,252	47,246	13,970	8,119	2,252	0,599	1,210	0,795	1,319
Krt15	0,296	0,412	0,275	0,255	0,407	0,289	0,403	0,631	0,328	0,490	1,938	0,409	0,131	0,000	1,250	2,445	0,730	5,541	13,248	33,265	1,627	0,748	0,716	0,505
Krt17	4,779	8,326	8,196	3,628	0,835	0,969	0,416	0,495	0,461	0,655	0,898	0,796	0,244	0,020	57,991	90,720	61,224	27,410	23,654	5,709	1,098	1,987	0,542	3,088
Krt25	1,021	0,537	0,354	1,338	1,064	1,144	0,789	0,154	4,976	6,523	12,289	39,305	6,776	4,880	0,205	0,162	1,116	0,126	0,101	0,132	2,310	1,720	1,698	1,090
Krt28	0,837	0,476	1,082	2,578	1,096	1,345	0,906	0,212	6,103	9,386	10,458	9,490	16,683	55,021	0,025	0,213	0,106	0,057	0,163	0,215	2,361	1,783	2,169	1,645
Krt31	0,006	0,044	0,027	0,010	0,006	0,039	0,024	15,454	0,014	0,010	0,014	0,050	0,030	0,090	0,000	0,097	0,068	0,041	0,017	0,000	0,037	0,026	0,025	0,020
Krt35	0,280	0,211	0,013	1,636	5,178	9,206	27,673	31,629	0,185	0,194	0,385	0,427	2,872	0,528	0,235	0,060	0,110	0,095	0,152	0,000	0,127	0,312	0,226	0,418

Notes: Colors highlight expression levels in cell clusters separately for transglutaminases and keratins, whereby dark red is assigned to the highest level observed. Boxes highlight P<0.01 (Mann-Whitney U / Wilcoxon rank sum test against average over all other cell populations, Benjamini Hochberg-corrected)
Data available (open access) at https://kasperlab.org/mouseskin (last accessed on 31 January 2023).

Table S10. Expression levels of TGMs and keratin markers in mouse epidermis and upper hair follicle, as determined by scRNA-seq (Joost et al. 2020)

Gene	Interfollicular epidermis basal, cycling (IFE C)	Interfollicular epidermis basal (IFE B)	Interfollicular epidermis suprabasal 1 (IFE SB1)	Interfollicular epidermis suprabasal 2 (IFE SB2)	Upper hair follicle basal (uHF B)	Upper hair follicle suprabasal (uHF SB)	Outer bulge of hair follicle (OB)	Hair germ (HG)	Sebaceous gland (SG)
Transglutaminases									
Tgm1	0,000	0,000	0,008	0,066	0,002	0,021	0,000	0,000	0,000
Tgm2	0,012	0,004	0,008	0,010	0,002	0,000	0,025	0,023	0,084
Tgm3	0,018	0,014	0,016	0,020	0,002	0,000	0,002	0,000	0,000
Tgm4	0,000	0,000	0,000	0,000	0,010	0,000	0,008	0,000	0,000
Tgm5	0,006	0,015	0,021	0,044	0,019	0,026	1,668	0,178	0,095
Tgm6	0,009	0,000	0,008	0,001	0,007	0,000	0,004	0,000	0,000
Tgm7	0,000	0,004	0,003	0,002	0,000	0,000	0,012	0,000	0,000
Keratins									
Krt10	1,034	2,403	46,642	92,874	2,243	14,599	1,439	1,500	0,253
Krt14	67,296	63,476	15,009	3,981	31,532	11,931	15,710	16,801	0,000
Krt15	42,654	60,674	37,967	13,781	43,818	20,644	110,068	16,806	0,000
Krt17	1,627	2,005	0,653	3,692	9,400	81,975	17,442	13,368	0,000
Krt79	0,042	0,058	0,121	0,175	2,071	30,410	0,087	0,050	0,000

Notes: Colors highlight expression levels in cell clusters separately for transglutaminases and keratins, whereby dark red is assigned to the highest level observed.
Boxes highlight P<0.01 (Mann-Whitney U / Wilcoxon rank sum test against average over all other cell populations, Benjamini Hochberg-corrected).
Data available (open access) at https://kasperlab.org/mouseskin (last accessed on 31 January 2023).

Table S11. Transglutaminases detected by mass spectrometry-based proteomics of mouse hair shafts (data from: Sukseree et al. 2024, Table S2)

Accession nr.	Protein	Coverage (%)	#Peptides	#Unique	Protein abundance (abundance units according to PEAKS Studio Q module)													Mean abundance	Mean abundance
					WT1	WT2	WT3	WT4	WT5	WT6	EKO1	EKO2	EKO3	EKO4	EKO5	EKO6	EKO7	WT	EKO
A0A0R4J293	TGM1	29	16	16	1,8E+07	1,5E+07	1,5E+07	2,2E+07	1,6E+07	1,8E+07	1,6E+07	1,5E+07	1,1E+07	1,7E+07	1,8E+07	1,7E+07	1,8E+07	1,7E+07	1,6E+07
Q08189	TGM3	50	42	42	8,7E+07	8,5E+07	7,6E+07	3,3E+07	9,5E+07	7,3E+07	1,2E+08	1,1E+08	1,1E+08	1,1E+08	1,3E+08	9,8E+07	1,2E+08	7,5E+07	1,1E+08
Q8BM11	TGM6	48	25	25	2,7E+07	2,5E+07	3,1E+07	1,8E+07	3,2E+07	3,1E+07	2,2E+07	2,3E+07	2,6E+07	2,3E+07	2,0E+07	2,4E+07	2,9E+07	2,7E+07	2,4E+07

Notes: Protein abundance was determined with PEAKS Studio Q module (Sukseree et al. 2024)

Abbreviations: WT, wildtype (Atg7f/f); EKO, epithelial knockout (Atg7f/f K14-Cre)

Individual measurements in 6 and 7 biological replicates of WT and EKO mice are shown to demonstrates consistency of expression. The protein abundance levels in WT and Atg7 EKO hair shafts were compared previously (Sukseree et al. 2024).

Table S12. GenBank accessions for genes at the TGM9 locus of different species (Figure 5A)

Species	Species name	Gene	chromosome/scaffold	NCBI GenBank accession	Notes
Homo sapiens	human	*DSEL*	chromosome 18	NP_115536	
Homo sapiens	human	*TMX3*	chromosome 18	NP_001337443	
Sarcophilus harrisii	tasmanian devil	*DSEL*	chromosome 1	XP_003760137	
Sarcophilus harrisii	tasmanian devil	*TMX3*	chromosome 1	XP_012399569	
Ornithorhynchus anatinus	platypus	*DSEL*	chromosome 5	XP_028921649	
Ornithorhynchus anatinus	platypus	*TGM9*	chromosome 5	XP_028921818	
Ornithorhynchus anatinus	platypus	*TMX3*	chromosome 5	XP_028921009	
Gallus gallus	chicken	*DSEL*	chromosome 2	XP_040551582	
Gallus gallus	chicken	*TGM9*	chromosome 2	XP_015137927	
Gallus gallus	chicken	*TMX3*	chromosome 2	NP_001308535	
Alligator sinensis	alligator	*TGM9*	scaffold NW_005842697.1	XP_006034123	
Alligator sinensis	alligator	*TMX3*	scaffold NW_005842697.1	XP_006034122	
Mauremys reevesii	turtle	*DSEL*	linkage group 2 NC_052624.1	XP_039381881	
Mauremys reevesii	turtle	*TGM9*	linkage group 2 NC_052624.1	XP_039378944	
Mauremys reevesii	turtle	*TMX3*	linkage group 2 NC_052624.1	XP_039379300	
Rhineura floridana	worm lizard	*DSEL*	chromosome 1	XP_061450672	
Rhineura floridana	worm lizard	*TGM9*	chromosome 1	n.a.	premature stop codon
Rhineura floridana	worm lizard	*TMX3*	chromosome 1	XP_061450648	
Podarcis muralis	wall lizard	*DSEL*	chromosome 7	XP_028593273	
Podarcis muralis	wall lizard	*TGM9*	chromosome 7	XP_028593274	annotation incomplete (NCBI)
Podarcis muralis	wall lizard	*TGM9*	chromosome 7	XP_028594402	annotation incomplete (NCBI)
Podarcis muralis	wall lizard	*TMX3*	chromosome 7	XP_028593275	
Pantherophis guttatus	snake	*DSEL*	scaffold NW_026844077.1	XP_034285274	
Pantherophis guttatus	snake	*TMX3*	scaffold NW_026844077.1	XP_034285272	
Anolis carolinensis	anole lizard	*DSEL*	chromosome 4	XP_003219769	
Anolis carolinensis	anole lizard	*TGM9*	chromosome 4	XP_003219770	corrected, fused with TMX3 in GenBank*
Anolis carolinensis	anole lizard	*TMX3*	chromosome 4	XP_016848401	fused with TGM9 in GenBank
Gekko japonicus	gecko	*TGM9*	NW_015164209.1	XP_015263918	
Gekko japonicus	gecko	*TMX3*	NW_015164209.1	XP_015263919	
Xenopus tropicalis	clawed frog	*DSEL*	chromosome 6	XP_002939660	
Xenopus tropicalis	clawed frog	*TMX3*	chromosome 6	XP_002939661	

* The amino acid sequence is shown in Figure S1.
